# Synthesis of Sulfated Glycomimetics with Micromolar Affinity for Midkine

**DOI:** 10.1021/acs.joc.6c00064

**Published:** 2026-03-27

**Authors:** Rocío Pereira-Jaramillo, José L. de Paz, Pedro M. Nieto

**Affiliations:** Glycosystems Laboratory, Instituto de Investigaciones Químicas (IIQ), cicCartuja, 16379 CSIC and Universidad de Sevilla, Americo Vespucio, 49, Sevilla 41092, Spain

## Abstract

A series of sulfated oligosaccharides (one tetra- and ten disaccharides) have been synthesized to study their interactions with midkine, a heparin-binding growth factor involved in cancer and inflammation. These compounds were prepared as glycosaminoglycan (GAG) mimetics and displayed hydrophobic groups at specific positions to enhance midkine binding. For the synthesis of the tetrasaccharide, a fluoro-assisted strategy was adopted. The use of an *N*-phenyltrifluoroacetimidate donor, instead of the analogous trichloroacetimidate, proved to be crucial in obtaining the desired tetramer with good yield. On the other hand, the synthesized disaccharides differed in the number of sulfates present and in the substituent at position 2 of the glucosamine unit. Fluorescence polarization competition experiments provided relative binding affinities for each glycomimetic expressed as IC_50_ values. Our results indicated that all the mimetics interacted with midkine in the micromolar range, highlighting the affinity of the 4,6-di-*O*-sulfated disaccharides with aromatic rings on the 2-amide group (IC_50_’s from 3.4 ± 0.8 to 4.3 ± 1.1 μM). Overall, this study offers valuable data for the design and synthesis of high-affinity midkine ligands with potential biological applications.

## Graphical abstract

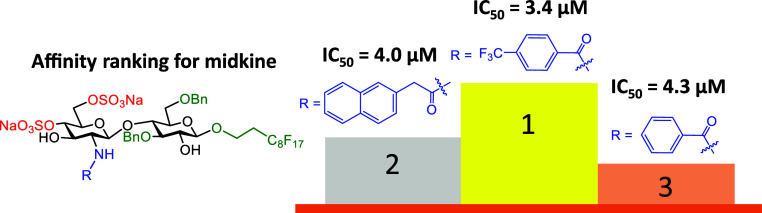

## Introduction

Glycosaminoglycans (GAGs) are a family of negatively charged polysaccharides, including heparin, heparan sulfate, chondroitin sulfate (CS), and hyaluronic acid, that control multiple biological processes by interaction with their protein receptors.^1^ One of these proteins is midkine, a heparin-binding growth factor involved in cancer, inflammation, and cardiovascular diseases.^2–4^

The design and synthesis of mimetics that replicate the structure and function of naturally occurring GAGs is an attractive approach toward the development of therapeutics.^5,6^ These analogues offer improved structural definition in contrast to the typical heterogeneity of native GAGs and include multivalent systems decorated with sulfated carbohydrate ligands^7–14^ and noncarbohydrate, sulfated compounds possessing an aromatic scaffold.^15–18^ In addition, unnatural sulfated oligosaccharides are often prepared and employed as GAG mimetics,^19–33^ including Pixatimod (PG545), a persulfated glucose (Glc) tetrasaccharide with anticancer and antiviral activities.^34,35^ Their structures are usually simpler and more accessible synthetically than those of natural GAG oligosaccharides.

In this context, we previously reported the synthesis of tetrasaccharides **1** and **2**, following the sequence GlcN­(4,6-di-OSO_3_)-β­(1 → 4)-Glc-β(1 → 3), as mimetics of biologically active chondroitin sulfate E (CS-E) (Figure 1).^36^ These compounds also share some structural similarities with other GAGs: they contain 6-*O*-sulfated glucosamine units, as in heparin, and β-linked, 3-*O*-glycosylated glucosamine residues, as in hyaluronic acid (Figure 1). We demonstrated that these compounds were able to interact with midkine, showing IC_50_ values of 32 ± 15 (**1**) and 4.2 ± 0.5 (**2**) μM, and to block the midkine-stimulating effect on cell growth. Their binding affinities for midkine were much higher than that corresponding to a CS-E tetrasaccharide (IC_50_ = 254 μM).^37^ Derivatives **1** and **2** contain benzyl groups at position 3 of the Glc units and a fluorinated C_8_F_17_ tag at the reducing end anomeric position. Our previous results^27^ indicated that the presence of aromatic substituents enhanced the binding to midkine by establishing additional interactions with the amino acids of the protein binding site. This argument could explain the greater affinity of **2**, which has a 2-benzamide group, compared to that of **1**. In addition, we hypothesized that the fluorous tag can contribute to the interaction by making additional contacts with the protein surface. On the other hand, increasing the overall hydrophobicity of our glycomimetics can improve protein binding due to the hydrophobic effect.

**Figure 1:**
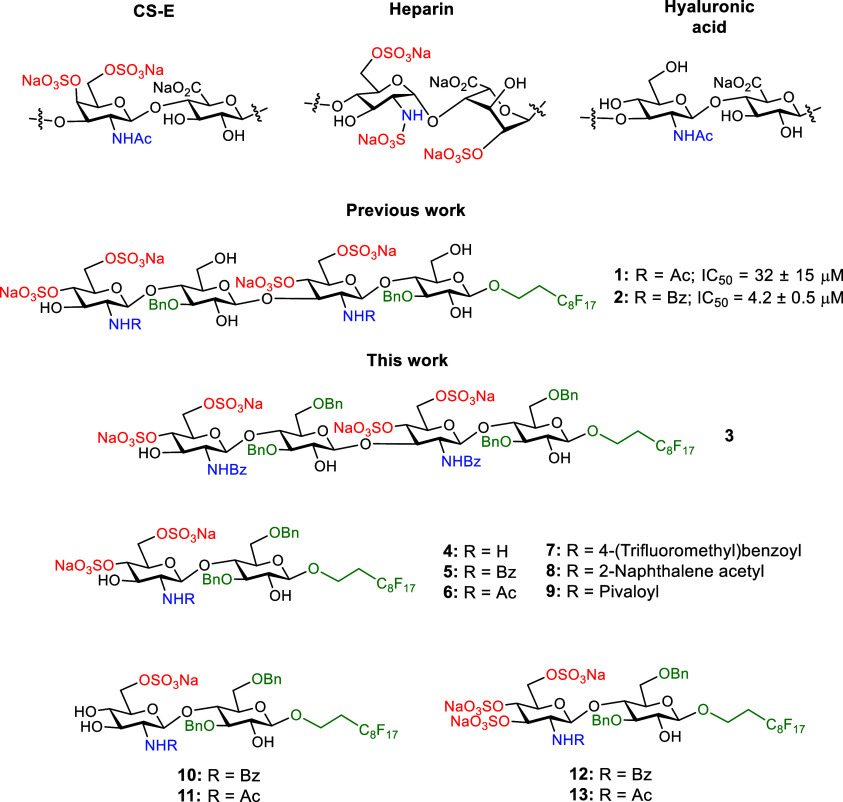
Structures of glycomimetics **1**–**13**. Native disaccharide repeating units (sodium salts) of CS-E, heparin (regular region), and hyaluronic acid are also shown for comparison purposes.

For all of this, we present here the synthesis of tetrasaccharide **3** displaying additional benzyl rings at position 6 of the Glc moieties. We hypothesized that the presence of these groups should increase the affinity of **3** for midkine. High-affinity ligands are required to potentially modulate the biological functions of this protein.

On the other hand, we also describe here the preparation of disaccharides **4–13** in which we have modified the sulfation pattern and the nature of the substituent at position 2 of the glucosamine (GlcN) unit, with the aim of studying how these structural variations affect the interaction with midkine, at the disaccharide level. The study of the interaction between the synthesized glycomimetics **3**–**13** and midkine, using fluorescence polarization (FP) experiments, provided useful data for establishing structure–affinity relationships and for designing high-affinity ligands with possible biological relevance.

## Results and Discussion

### Synthesis of Tetrasaccharide 3

For the synthesis of tetrasaccharide **3**, we first prepared trichloroacetimidate **17** (Scheme 1). Known derivative **14** ^38^ was treated with triethylsilane and trifluoroacetic acid to give 6-*O*-benzylated monosaccharide **15** in excellent yield. Levulinoyl (Lev) group was then installed at position 4 using levulinic anhydride and 4-dimethylamino pyridine (DMAP) as a catalyst. Oxidative removal of the 4-methoxyphenyl (MP) group with cerium­(IV) ammonium nitrate (CAN) at 0 °C afforded hemiacetal **16** in good yield. Treatment with trichloroacetonitrile and potassium carbonate afforded glycosyl donor **17** as a mixture of α/β anomers.

**Scheme 1:**
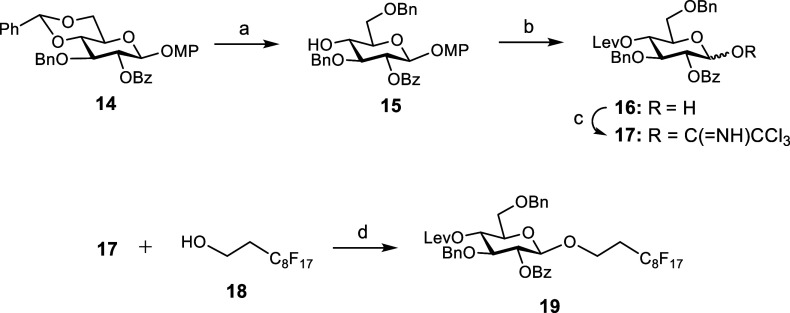
Reagents and Conditions: (a) Et_3_SiH, CF_3_COOH, CH_2_Cl_2_, 80%; (b) Lev_2_O, DMAP, CH_2_Cl_2_, 90%; CAN, CH_2_Cl_2_/CH_3_CN/H_2_O, 0 °C, 84%; and (c) Cl_3_CCN, K_2_CO_3_, CH_2_Cl_2_, 80%; (d) TBSOTf, CH_3_CN, Room Temperature, 64%

Next, we accomplished the glycosylation reaction between **17** and fluorinated decanol **18** at room temperature, employing *tert*-butyldimethylsilyl trifluoromethanesulfonate (TBSOTf) as the catalyst and CH_3_CN as the solvent. The crude product was purified by fluorous solid-phase extraction (F-SPE).^39,40^ NMR analysis of the fluorophilic fraction indicated the formation of the desired β glycoside as the major product, together with a small amount of α anomer (1:10 α/β mixture). This is in good agreement with our previous results in the glycosylations between perfluorinated alcohols and trichloroacetimidate donors.^36^ Pure β anomer **19** was obtained by silica gel column chromatography in a 64% yield.

Starting from **19**, an iterative sequence of Lev deprotection and glycosylation reactions was then carried out to build the tetrasaccharidic chain (Scheme 2). The presence of the fluorinated tail enabled the purification of the reaction intermediates by simple F-SPE. Thus, treatment with hydrazine monohydrate in a pyridine/acetic acid buffer afforded glycosyl acceptor **20**. Glycosylation between **20** and trichloroacetimidate **21** ^41^ was performed at room temperature using TBSOTf in CH_2_Cl_2_ to provide the desired disaccharide **22**. The *N*-phthaloyl (*N*-Phth) participating group at position 2 of the donor led to the exclusive formation of the 1,2-*trans* glycosidic bond. Next, the Lev group was selectively removed to obtain disaccharide acceptor **23**. Synthesis of trisaccharide **24** proved to be challenging. Compound **23** was first reacted with 3 equiv of trichloroacetimidate **17**, using the reaction conditions optimized in our group for this type of couplings (room temperature, 20 mol % of TBSOTf with respect to the donor and CH_2_Cl_2_ as the solvent). The crude mixture was purified by F-SPE, and the TLC and NMR analysis of the fluorinated fraction showed unreacted **23** as the major product along with a small amount of the desired trisaccharide. Disappointingly, after two more glycosylation cycles with additional donor **17** (3 + 3 equiv), trisaccharide **24** was isolated in a poor 33% yield.

**Scheme 2:**
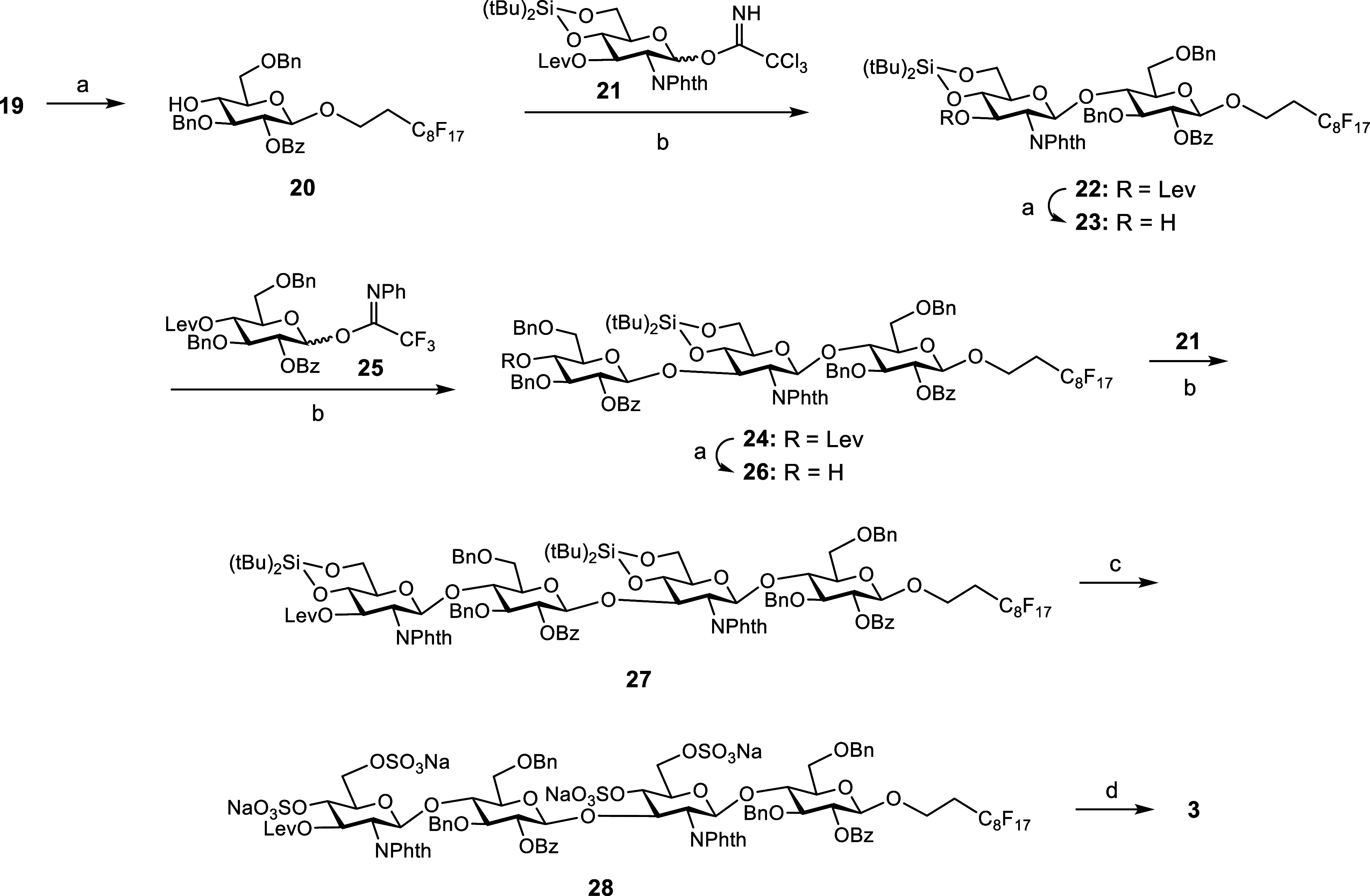
Reagents and Conditions: (a) NH_2_NH_2_·H_2_O (2 equiv), Py/AcOH, CH_2_Cl_2_; (b) TBSOTf, CH_2_Cl_2_, Room Temperature, 36% (**27**) from **19**, 6 Steps; (c) (HF)_*n*_·Py, THF, 0 °C; SO_3_·Me_3_N, DMF, 100 °C, MW Heating, 63%; and (d) NH_2_(CH_2_)_2_NH_2_, *n*-BuOH/MeOH/CF_3_CH_2_OH, 120 °C, MW Heating; NaOH, MeOH/CF_3_CH_2_OH; Bz_2_O, Et_3_N, MeOH, 87%

*N*-Phenyltrifluoroacetimidates have been extensively used in oligosaccharide synthesis as alternative donors to trichloroacetimidates.^42–44^ Glycosyl donor **25** ^45,46^ proved to be more efficient than **17** in the preparation of trisaccharide **24**. After two reaction cycles, glycosylation between disaccharide **23** and *N*-phenyltrifluoroacetimidate **25** afforded **24** as the main product. Subsequent Lev deprotection (→**26**) and condensation with trichloroacetimidate **21** led to the target tetrasaccharide **27** in an excellent 36% yield from **19** (6 steps, 85% average yield per step). Selective removal of the cyclic silylene groups was performed with the (HF)_*n*_·Py complex in THF at 0 °C. The released hydroxyl groups were then sulfated using the SO_3_·NMe_3_ complex in DMF under microwave irradiation at 100 °C (→**28**). Finally, *N*-Phth, Bz, and Lev groups were deprotected with ethylenediamine at 120 °C under microwave heating, followed by treatment with an aqueous solution of NaOH (4 M). Selective *N*-benzoylation was performed using benzoic anhydride and Et_3_N in MeOH to afford tetrasaccharide **3** in an 87% yield.

### Synthesis of Disaccharides 4–13

Taking advantage of the preparation of disaccharide **22**, we also decided to prepare derivatives **4–13** in order to study the influence of the number of sulfate groups and the nature of the substituent at position 2 of the GlcN unit on the binding affinity for midkine at the disaccharide level (Scheme 3). In this way, for the preparation of the disaccharides with sulfate groups at positions 4 and 6 of the GlcN unit, compound **22** was treated with the (HF)_*n*_·Py complex in THF at 0 °C and then sulfated with SO_3_·Me_3_N (10 equiv. per hydroxyl group) in DMF at 100 °C to yield derivative **29**. Treatment with ethylene diamine in a *n*-BuOH/MeOH/CF_3_CH_2_OH mixture at 120 °C (microwave heating) and then with 4 M NaOH in MeOH/CF_3_CH_2_OH afforded disaccharide **4**, displaying a free amino group. *N*-Acylation was performed using the corresponding anhydrides and Et_3_N in MeOH. After silica gel chromatography and treatment with Dowex Na^+^ ion-exchange resin, disaccharides **5–8** were obtained in excellent yields as sodium salts. Similarly, *N*-pivaloylation was accomplished by using pivaloyl (Piv) chloride (→**9**).

**Scheme 3:**
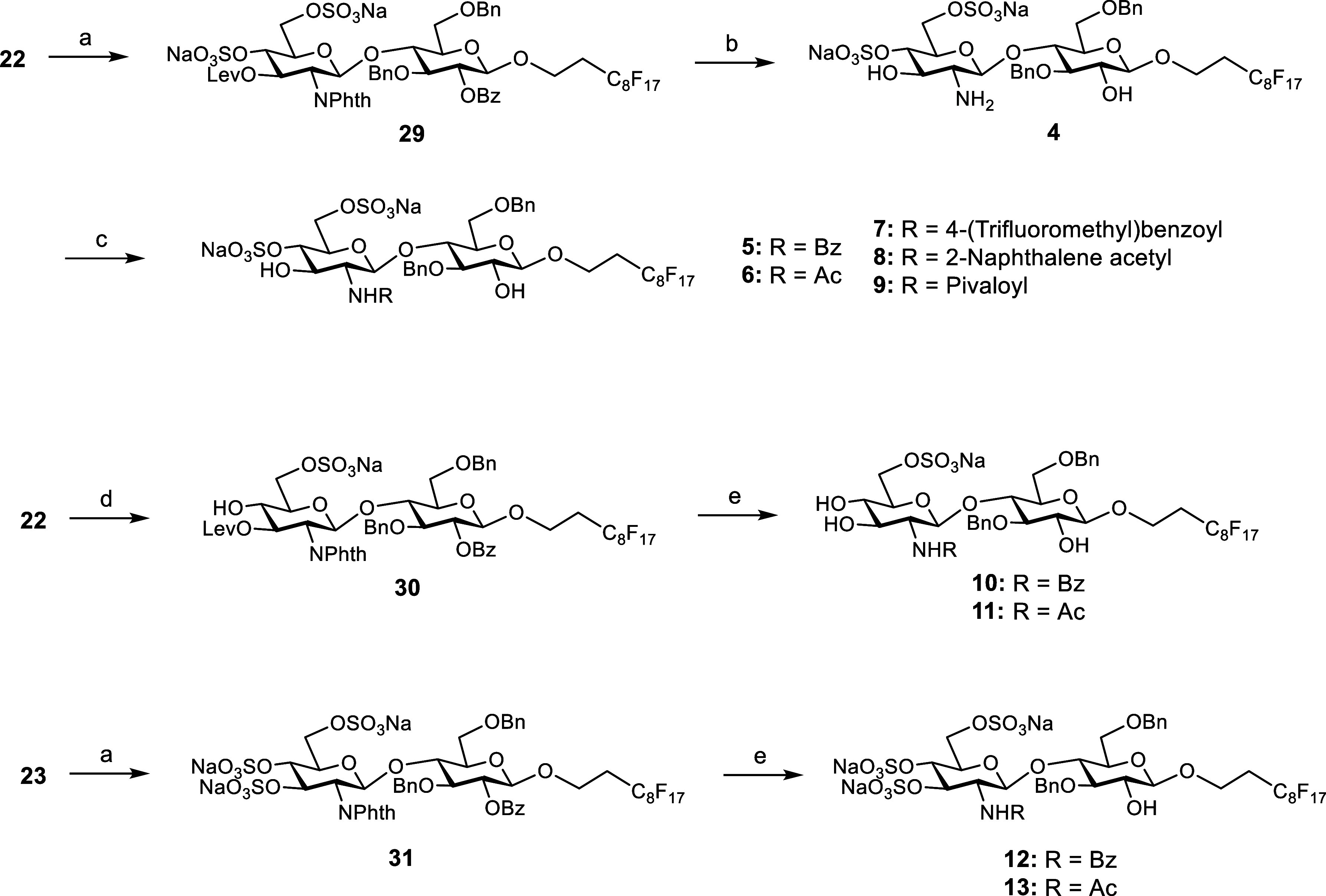
Reagents and Conditions: (a) (HF)_*n*_·Py, THF, 0 °C; SO_3_·Me_3_N (10 equiv. per OH), DMF, 100 °C, MW Heating, 83% (**29**), 62% (**31**); (b) NH_2_(CH_2_)_2_NH_2_, *n*-BuOH/MeOH/CF_3_CH_2_OH, 120 °C, MW Heating; NaOH, MeOH/CF_3_CH_2_OH, 86%; (c) The Corresponding Acid Anhydride (Benzoic, Acetic, 4-(Trifluoromethyl)­benzoic or 2-Naphthaleneacetic Anhydride) or Acyl Chloride (Pivaloyl Chloride), Et_3_N, MeOH, 85% (**5**), 81% (**6**), 79% (**7**), 88% (**8**), 87% (**9**); (d) (HF)_*n*_·Py, THF, 0 °C; SO_3_·Me_3_N (3 equiv), DMF, 45 °C, MW Heating, 74%; and (e) NH_2_(CH_2_)_2_NH_2_, *n*-BuOH/MeOH/CF_3_CH_2_OH, 120 °C, MW Heating; NaOH, MeOH/CF_3_CH_2_OH; Bz_2_O or Ac_2_O, Et_3_N, MeOH, 73% (**10**), 55% (**11**), 80% (**12**), 79% (**13**)

Mono 6-*O*-sulfated intermediate **30** was prepared by using 3 equiv of SO_3_·Me_3_N at 45 °C. Deprotection of the *N*-Phth and acyl groups followed by *N*-benzoylation/acetylation provided disaccharides **10** and **11**. On the other hand, for the synthesis of the 3,4,6-tri-*O*-sulfated derivatives **12** and **13**, the cyclic silylene group of disaccharide **23** was first deprotected and the resulting triol was then extensively sulfated using 10 equiv of SO_3_·Me_3_N per hydroxyl group at 100 °C to give compound **31**. Finally, treatment with benzoic and acetic anhydrides led to the formation of disaccharides **12** and **13**, respectively.

### Interaction Studies between Glycomimetics and Midkine

FP competition experiments^47^ were carried out to estimate the relative binding affinities of the synthesized glycomimetics **3–13** for midkine. We followed an FP experimental protocol previously developed in our group.^48,49^ In this assay, the ability of the different glycomimetics to block the formation of the complex between the protein and the fluorescent probe (compound **32**, Figure S1, Supporting Information) was measured. To this end, FP of samples containing fixed amounts of protein and fluorescent probe and increasing concentrations of the mimetics were recorded. The resulting inhibition curves were mathematically fitted, and an IC_50_ value for each glycomimetic (defined as the concentration required for 50% inhibition) was obtained (Figure 2). The reported IC_50_s represent the average from three independent experiments. All of the inhibition curves are shown in the Supporting Information.

**Figure 2:**
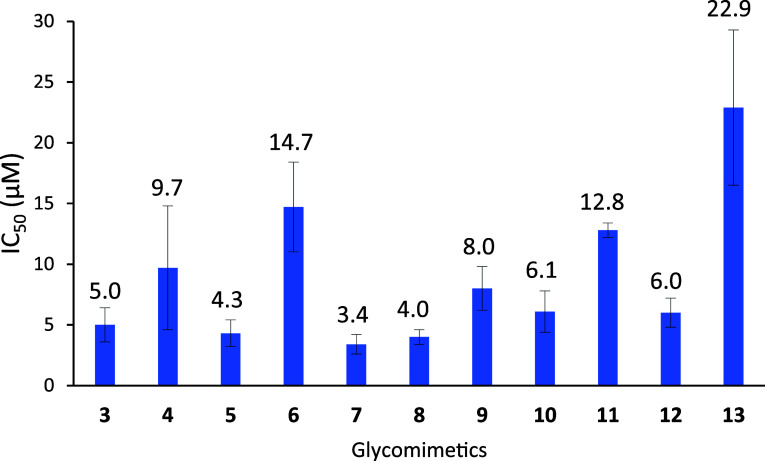
Bar chart showing the binding affinity of the synthesized glycomimetics for midkine, as determined by FP competition assays. The IC_50_ values (μM, shown above bars) and the error bars represent, respectively, the average and the standard deviation from three independent experiments.

The binding affinity of tetrasaccharide **3** (IC_50_ = 5.0 ± 1.4 μM) was very similar to that of **2** (see Figure 1; IC_50_ = 4.2 ± 0.5 μM), indicating that the presence of the benzyl rings at position 6 of the Glc moieties did not enhance the interaction with midkine. In contrast to our previous findings, this result suggests that an increase in the hydrophobicity of the molecule (from **2** to **3**) does not always imply an increase in midkine affinity. On the other hand, disaccharides **4–13** also interacted with midkine in the micromolar range, with IC_50_’s ranging from 3.4 ± 0.8 to 22.9 ± 6.4 μM. The highest affinities were shown by the 4,6-di-*O*-sulfated disaccharides containing aromatic rings on the amide group (**5**, **7**, **8**). In particular, disaccharide **7** presenting a 4-(trifluoromethyl)­benzamide gave the lowest IC_50_ value (IC_50_ = 3.4 ± 0.8 μM) followed by 2-naphthaleneacetamide-containing **8** (IC_50_ = 4.0 ± 0.6 μM) and benzamide derivative **5** (IC_50_ = 4.3 ± 1.1 μM). Based on our previous results,^27^ in addition to increasing overall hydrophobicity, aromatic substituents can establish hydrophobic and π–π stacking interactions with aromatic amino acids of the midkine binding side. Compounds **4**, **6,** and **9** do not have aromatic rings at position 2 of the glucosamine unit and therefore cannot establish these additional interactions with the protein. Thus, the presence of a free amino group decreased the level of interaction (compound **4**, IC_50_ = 9.7 ± 5.1 μM). Disaccharides **6** and **9**, displaying *N*-acetyl and *N*-pivaloyl groups, also showed lower affinity values (IC_50_ = 14.7 ± 3.7 and 8.0 ± 1.8 μM, respectively). The same trend was observed for the trisulfated and monosulfated compounds: the *N*-benzoylated derivatives gave higher affinities than the *N*-acetylated ones (compound **10**, IC_50_ = 6.1 ± 1.7 μM against compound **11**, IC_50_ = 12.8 ± 0.6 μM; compound **12**, IC_50_ = 6.0 ± 1.2 μM against compound **13**, IC_50_ = 22.9 ± 6.4 μM).

Electrostatic interactions between sulfate groups and basic amino acids are the main driving force behind GAG-protein binding. Regarding the number of sulfates, 4,6-di-*O*-sulfated disaccharides generally interacted more strongly than 3,4,6-tri-*O*-sulfated and mono-6-*O*-sulfated derivatives (compare IC_50_ values for *N*-benzoylated disaccharides **5**, **10,** and **12** and for *N*-acetylated compounds **6** and **13**). However, in the case of *N*-acetylated derivatives, monosulfated **11** did not follow the same trend and interacted slightly more strongly than disulfated **6** (IC_50_ = 12.8 ± 0.6 μM versus IC_50_ = 14.7 ± 3.7 μM).

The natural ligands of midkine are CS-E and heparin oligosaccharides.^50–52^ Interestingly, the synthesized glycomimetics **3**–**13** exhibited higher affinities than CS-E tetra and hexasaccharides^37,53^ but lower than a heparin hexasaccharide.^54^ In particular, a CS-E tetrasaccharide (compound **33**; Figure S2) provided an IC_50_ of 254 μM in our FP competition assay, while a heparin hexasaccharide (compound **34**, Figure S2) gave an IC_50_ of 1.1 μM.^37,54^ Our results highlight that compounds as small as disaccharides can be designed to bind midkine, with affinities superior or comparable to those of longer natural ligands, by strategically introducing hydrophobic groups at certain positions.

## Conclusions

The search for low-molecular-weight compounds that mimic GAG oligosaccharides, binding with high affinity to their protein receptors, is a promising strategy for modulating important biological processes. In this work, we described the synthesis of a series of sulfated glycomimetics that show micromolar affinity for midkine, a protein with a key role in cancer cell growth. These compounds were prepared to investigate the influence of certain structural variations, such as the presence of additional aromatic rings and the number of sulfate groups, on their interaction with this protein. It is important to note that our glycomimetics are synthetically more accessible than natural GAG oligosaccharides and, at the same time, exhibit affinities comparable to or even stronger than them. In particular, 4,6-di-*O*-sulfated disaccharides containing aromatic rings on the amide group were identified as the best midkine ligands. Overall, our results provide useful insights into the discovery of GAG mimetics with potential biological relevance.

## Experimental Section

### General Synthetic Procedures

Thin-layer chromatography (TLC) analyses were performed on silica gel 60F-254-precoated aluminum plates from Merck. The compounds were detected by UV visualization (λ = 254 nm) and by staining with 5% v/v anisaldehyde-5% v/v H_2_SO_4_-0.2% v/v AcOH in EtOH or 0.2% w/v cerium­(IV) sulfate-5% w/v ammonium molybdate tetrahydrate in 1 M H_2_SO_4_, followed by heating at over 200 °C. Column chromatography was carried out on silica gel 60 (0.2–0.063 mm or 0.040–0.015 mm; Merck). F-SPE was performed using FluoroFlash silica gel from Sigma-Aldrich. Optical rotations were determined with a PerkinElmer 341 polarimeter using a sodium lamp (λ = 589 nm) at 25 °C in 1 dm tubes. ^1^H, ^19^F, and ^13^C NMR spectra were acquired on a Bruker 400 spectrometer. 1-D TOCSY, 2-D COSY, and HSQC experiments were routinely carried out to assist in signal assignment. Unit A refers to the reducing end monosaccharide in the NMR data. Electrospray mass spectra (ESI MS) were carried out with an amaZon SL mass spectrometer from Bruker. High-resolution mass spectra (HR MS) were carried out by CITIUS (Universidad de Sevilla) using an Orbitrap Elite spectrometer from Thermo Scientific (mass analyzer type: ion trap/orbitrap). Microwave-based reactions were performed using a Biotage Initiator Eight synthesizer in sealed reaction vessels.

### General Procedure for F-SPE

As described previously,^49^ a glass chromatography column was packed with 5 g of FluoroFlash silica gel. Precondition was performed with MeOH/H_2_O 80:20 (15 mL). The crude sample (50–300 mg) was loaded on the column using DMF/H_2_O 9:1 (1 mL) and the fluorophobic and fluorophilic elutions were carried out with 15 mL of MeOH/H_2_O 80:20 and 20 mL of acetone, respectively. To regenerate the F-SPE column, we washed it with additional acetone (20 mL).

#### 4-Methoxyphenyl 2-*O*-Benzoyl-3,6-di-*O*-benzyl-β-d-glucopyranoside (**15**)

Compound **14** (2.72 g, 4.78 mmol) was coevaporated with toluene, dissolved in dry CH_2_Cl_2_ (27 mL) and further dried by stirring over activated 4 Å molecular sieves (2 g) for 15 min under an argon atmosphere. Triethylsilane (7.7 mL, 47.8 mmol) was then added at room temperature, and the mixture was stirred for 15 min. Finally, trifluoroacetic acid (3.9 mL, 50.2 mmol) was added dropwise at 0 °C. After stirring for 10 min at room temperature, the reaction was filtered and diluted with CH_2_Cl_2_. The reaction mixture was washed with saturated NaHCO_3_ and water, dried over Mg_2_SO_4_, filtered, and concentrated. The residue was purified by column chromatography (toluene-EtOAc 8:1) to afford **15** as a white amorphous solid (2.21 g, 80%). Spectroscopic data are in good agreement with those reported in the literature for this compound.^55^

#### 2-*O*-Benzoyl-3,6-di-*O*-benzyl-4-*O*-levulinoyl-α,β-d-glucopyranose (**16**)

1,3-Dicyclohexylcarbodiimide (1.38 g, 6.61 mmol) was dissolved in CH_2_Cl_2_ (23 mL). Levulinic acid (1.38 mL, 13.2 mmol) was then added at 0 °C, and the mixture was stirred for 5 min at room temperature and filtered. The resulting Lev_2_O solution (6.6 mmol) was added at room temperature (rt) to a mixture of **15** (1.16 g, 2.20 mmol) and DMAP (40 mg, 0.33 mmol). After stirring for 1 h 30 min at rt under dry argon, the reaction mixture was diluted with CH_2_Cl_2_ and washed with saturated aqueous NaHCO_3_ and H_2_O. The organic phase was dried (MgSO_4_), filtered, and concentrated to dryness. The residue was purified by column chromatography (toluene-EtOAc 8:1) to give the corresponding 4-*O*-levulinated compound (1.22 g, 90%) as a white, amorphous solid. TLC (toluene-EtOAc 8:1) Rf 0.23; ^1^H NMR (400 MHz, CDCl_3_): δ 8.02 (m, 2H, Ar), 7.63–7.14 (m, 13H, Ar), 6.92, 6.71 (2d, 4H, Ar), 5.54 (t, 1H, *J*_2,3_ = 8.6 Hz, H-2), 5.20 (t, 1H, *J*_3,4_ = *J*_4,5_ = 9.4 Hz, H-4), 5.00 (d, 1H, *J*_1,2_ = 7.8 Hz, H-1), 4.65–4.57 (m, 4H, CH_2_(Bn)), 3.92 (t, 1H, H-3), 3.78 (m, 1H, H-5), 3.72 (s, 3H, CH_3_(OMP)), 3.69 (m, 2H, H-6), 2.70–2.32 (m, 4H, CH_2_(Lev)), 2.14 (s, 3H, CH_3_(Lev)).

CAN (3.7 mL of a 1.08 M solution in H_2_O) was added to a solution of the 4-*O*-levulinated compound (665 mg, 0.99 mmol) in CH_2_Cl_2_/MeCN (1:2; 33.5 mL). After stirring for 2 h at 0 °C, the reaction mixture was diluted with EtOAc, washed with H_2_O, saturated aqueous NaHCO_3_, and H_2_O. The organic phase was dried (MgSO_4_), filtered, and concentrated to dryness. The residue was purified by column chromatography (toluene-EtOAc 6:1 → 4:1) to afford **16** as a white, amorphous solid (469 mg, 84%). Spectroscopic data are in good agreement with those reported in the literature for this compound.^46^

#### *O*-(2-*O*-Benzoyl-3,6-di-*O*-benzyl-4-*O*-levulinoyl-α,β-d-glucopyranosyl) Trichloroacetimidate (**17**)

Trichloroacetonitrile (100 μL, 0.94 mmol) and K_2_CO_3_ (15 mg, 0.113 mmol) were added to **16** (53 mg, 0.094 mmol) in dry CH_2_Cl_2_ (1 mL) under an argon atmosphere. After being stirred at room temperature for 4 h, the mixture was filtered and concentrated to dryness. The residue was purified by column chromatography (toluene-EtOAc 4:1 + 1% Et_3_N) to give **17** as a white amorphous solid (53 mg, 80%, mixture of α/β anomers). TLC (toluene-EtOAc 4:1) Rf 0.57 and 0.37; ^1^H NMR (400 MHz, CDCl_3_) (data for β anomer): δ 8.65 (s, 1H, NH), 7.99–7.13 (m, 15H, Ar), 6.00 (d, 1H, *J*_1,2_ = 7.7 Hz, H-1), 5.61 (t, 1H, *J*_2,3_ = 7.7 Hz, H-2), 5.32 (m, 1H, H-4), 4.75–4.51 (m, 4H, CH_2_(Bn)), 3.98 (t, 1H, *J*_3,4_ = 8.6 Hz, H-3), 3.92 (m, 1H, H-5), 3.77–3.56 (m, 2H, H-6a, H-6b), 2.64 (m, 2H, CH_2_(Lev)), 2.42 (m, 2H, CH_2_(Lev)), 2.15 (s, 3H, CH_3_(Lev)); ^13^C­{^1^H} NMR (100 MHz, CDCl_3_) (data for β anomer): δ 206.3, 171.4, 165.0 (3x CO), 160.8 (C = NH), 138.1–127.4 (Ar), 96.1 (C-1), 90.4 (CCl_3_), 79.3 (C-3), 74.7 (C-5), 73.7 (2x CH_2_(Bn)), 71.8 (C-2), 70.1 (C-4), 69.0 (C-6), 37.7 (CH_2_(Lev)), 29.8 (CH_3_(Lev)), 27.9 (CH_2_(Lev)). This compound proved to be too unstable to obtain MS data.

#### 1H,1H,2H,2H-Perfluorodecyl 2-*O*-Benzoyl-3,6-di-*O*-benzyl-4-*O*-levulinoyl-β-d-glucopyranoside (**19**)

**17** (100 mg, 0.141 mmol) was coevaporated with toluene and dried under high vacuum. Fluorinated decanol **18** (135 mg, 0.28 mmol) was added, and the mixture of solids was dissolved in dry CH_3_CN (2 mL) in the presence of activated 4 Å molecular sieves (150 mg). The reaction mixture was stirred for 10 min at room temperature under dry argon. TBSOTf (250 μL of a 0.17 M solution in dry CH_3_CN) was added, and the mixture was stirred for 30 min at rt. The reaction mixture was quenched with triethylamine (0.2 mL), filtered, and concentrated under reduced pressure. The residue was purified by F-SPE to give **19** as a 1:10 α/β mixture. Finally, standard silica gel column chromatography (toluene-EtOAc 16:1) afforded pure β anomer **19** as a white, amorphous solid (84 mg, 64%). TLC (toluene-EtOAc 8:1) Rf 0.3; [α][Bibr ref20]_D_ +10° (*c* 1.0, CHCl_3_); ^1^H NMR (400 MHz, CDCl_3_) (data for β anomer): δ 8.08–7.14 (m, 15H, Ar), 5.35 (t, 1H, *J*_1,2_ = *J*_2,3_ = 8.5 Hz, H-2), 5.19 (t, 1H, *J*_3,4_ = *J*_4,5_ = 9.5 Hz, H-4), 4.65–4.59 (m, 5H, CH_2_(Bn), H-1), 4.16 (m, 1H, CH_2_CH_2_C_8_F_17_), 3.92 (t, 1H, H-3), 3.83 (m, 1H, CH_2_CH_2_C_8_F_17_), 3.73–3.63 (m, 3H, H-5, H-6), 2.74–2.31 (m, 6H, CH_2_(Lev), CH_2_CH_2_C_8_F_17_), 2.17 (s, 3H, CH_3_(Lev)); ^13^C­{^1^H} NMR (100 MHz, CDCl_3_) (data for β anomer): δ 206.2, 171.6, 165.1 (3x CO), 138.2–127.4 (Ar), 101.2 (C-1), 79.8 (C-3), 73.86, 73.81, 73.66 (CH_2_(Bn), C-5), 73.0 (C-2), 70.9 (C-4), 69.5 (C-6), 61.8 (CH_2_CH_2_C_8_F_17_), 37.7 (CH_2_(Lev)), 31.5 (CH_2_CH_2_C_8_F_17_), 29.7 (CH_3_(Lev)), 27.9 (CH_2_(Lev)); ^19^F NMR (376 MHz, CDCl_3_): δ −80.8 (t, 3F), −113.3 (m, 2F), −122.0 (m, 6F), −122,7 (m, 2F), −123.6 (m, 2F), −126.2 (m, 2F); HR MS, *m*/*z*: calcd for C_42_H_37_F_17_O_9_Na: 1031.2058; found, 1031.2033 [*M* + Na]^+^.

#### 1H,1H,2H,2H-Perfluorodecyl *O*-(2-Deoxy-3-*O*-levulinoyl-2-phthalimido-4,6-*O*-di-*tert*-butylsilylene-β-d-glucopyranosyl)-(1 → 4)-*O*-(2-*O*-Benzoyl-3,6-di-*O*-benzyl-β-d-glucopyranosyl)-(1 → 3)-*O*-(2-Deoxy-2-phthalimido-4,6-*O*-di-*tert*-butylsilylene-β-d-glucopyranosyl)-(1 → 4)-2-*O*-Benzoyl-3,6-di-*O*-benzyl-β-d-glucopyranoside (**27**)

Monosaccharide **19** (84 mg, 0.083 mmol) was dissolved in dry CH_2_Cl_2_ (1.5 mL). A solution of hydrazine monohydrate in Py/AcOH 3:2 (0.33 mL of a 0.5 M solution) was added. After stirring at room temperature for 1.5 h, the reaction mixture was quenched with acetone (0.2 mL), diluted with CH_2_Cl_2_, and washed with 1 M HCl, saturated aqueous NaHCO_3_ solution, and H_2_O. The organic phase was dried (MgSO_4_), filtered, and concentrated to dryness. The residue was purified by F-SPE to give compound **20** (71 mg, 94%). TLC (toluene-EtOAc 8:1) Rf 0.3; ^1^H NMR (400 MHz, CDCl_3_): δ 8.08–7.16 (m, 15H, Ar), 5.30 (t, 1H, H-2), 4.79–4.57 (m, 5H, CH_2_(Bn), H-1), 4.17 (m, 1H, CH_2_CH_2_C_8_F_17_), 3.87–3.76 (m, 4H, CH_2_CH_2_C_8_F_17_, H-4, H-6), 3.71 (t, 1H, H-3), 3.59 (m, 1H, H-5), 2.38 (m, 2H, CH_2_CH_2_C_8_F_17_); ESI MS; *m*/*z*: calcd for C_37_H_31_F_17_O_7_Na: 933.2; found, 933.3 [*M* + Na]^+^.

Acceptor **20** (70 mg, 0.076 mmol) and donor **21** (105 mg, 0.152 mmol) were coevaporated with toluene and dried under a high vacuum. This mixture was dissolved in dry CH_2_Cl_2_ (2.5 mL) containing 4 Å molecular sieves (200 mg) and stirred under an argon atmosphere for 10 min. TBSOTf (100 μL of a 0.11 M solution in dry CH_2_Cl_2_) was added, and the mixture was stirred for 45 min at rt. Then, the reaction mixture was quenched with triethylamine (0.2 mL), filtered, and concentrated under reduced pressure. The residue was purified by F-SPE to give disaccharide **22** as a white, amorphous solid (100 mg, 91%). TLC (toluene-EtOAc 8:1) Rf 0.45; [α][Bibr ref20]_D_ −2° (*c* 1.0, CHCl_3_); ^1^H NMR (400 MHz, CDCl_3_): δ 7.99–7.18 (m, 19H, Ar), 5.67 (dd, 1H, *J*_3,4_ = 9.0 Hz, *J*_2,3_ = 10.7 Hz, H-3′), 5.61 (d, 1H, *J*_1,2_ = 8.2 Hz, H-1′), 5.21 (t, 1H, *J*_1,2_ = *J*_2,3_ = 8.3 Hz, H-2), 4.85–4.71 (2d, 2H, CH_2_(Bn)), 4.50–4.35 (m, 3H, CH_2_(Bn), H-1), 4.25 (dd, 1H, H-2′), 4.19 (t, 1H, *J*_3,4_ = *J*_4,5_ = 8.6 Hz, H-4), 4.06–3.95 (m, 2H, H-6′, CH_2_CH_2_C_8_F_17_), 3.85 (t, 1H, *J*_4,5_ = 9.2 Hz, H-4′), 3.77 (t, 1H, H-3), 3.71–3.61 (m, 2H, H-6′, CH_2_CH_2_C_8_F_17_), 3.57–3.47 (m, 2H, H-6), 3.39 (m, 2H, H-5, H-5′), 2.65–2.25 (m, 6H, CH_2_(Lev), CH_2_CH_2_C_8_F_17_), 1.96 (s, 3H, CH_3_(Lev)), 1.03, 0.91 (2s, 18H, C­(CH_3_)_3_); ^13^C­{^1^H} NMR (100 MHz, CDCl_3_): δ 205.6, 171.9, 168.3, 167.7, 165.1 (CO), 138.2–123.4 (Ar), 101.0 (C-1), 97.7 (C-1′), 80.3 (C-3), 75.6 (C-4′), 75.3 (C-4), 74.9 (C-5), 73.9 (CH_2_(Bn)), 73.0 (C-2), 72.7 (CH_2_(Bn)), 72.3 (C-3′), 70.4 (C-5′), 68.0 (C-6), 66.0 (C-6′), 61.3 (CH_2_CH_2_C_8_F_17_), 55.3 (C-2′), 37.8 (CH_2_(Lev)), 31.5 (t, CH_2_CH_2_C_8_F_17_), 29.4 (CH_3_(Lev)), 27.9 (CH_2_(Lev)), 27.3, 26.8 (Si­(C­(CH_3_)_3_)_2_), 22.6, 19.9 (Si­(C­(CH_3_)_3_)_2_); HR MS; *m*/*z*: calcd for C_64_H_66_F_17_O_15_NSiNa: 1462.3822; found, 1462.3804 [*M* + Na]^+^.

Compound **22** (100 mg, 0.069 mmol) was dissolved in CH_2_Cl_2_ (1.5 mL), and hydrazine monohydrate (276 μL of a 0.5 M solution in Py/AcOH 3:2) was added. After being stirred at room temperature for 1 h, the reaction mixture was quenched with acetone (0.2 mL). The mixture was diluted with CH_2_Cl_2_ and washed with 1 M HCl aqueous solution, saturated NaHCO_3_ aqueous solution, and H_2_O. The organic layer was dried (MgSO_4_), filtered, and concentrated in vacuo. The crude product was purified by F-SPE to give disaccharide **23** as a white foam (79 mg, 85%). TLC (toluene-EtOAc 12:1) Rf 0.25; ^1^H NMR (400 MHz, CDCl_3_): δ 7.98–7.17 (m, 19H, Ar), 5.49 (d, 1H, *J*_1,2_ = 8.3 Hz, H-1′), 5.20 (t, 1H, *J*_1,2_ = *J*_2,3_ = 8.2 Hz, H-2), 4.83–4.69 (2d, 2H, *J* = 11.3 Hz, CH_2_(Bn)), 4.46–4.30 (m, 4H, 2x CH_2_(Bn), H-1, H-3′), 4.18 (m, 2H, H-2′, H-4), 3.99 (m, 2H, H-6′, CH_2_CH_2_C_8_F_17_), 3.77 (t, 1H, H-3), 3.70–3.59 (m, 3H, H-4′, H-6′, CH_2_CH_2_C_8_F_17_), 3.57–3.47 (m, 2H, 2x H-6), 3.39–3.28 (m, 2H, H-5, H-5′), 2.32 (m, 2H, CH_2_CH_2_C_8_F_17_), 1.04, 0.93 (2s, 18H, Si­(C­(CH_3_)_3_)_2_); ^13^C­{^1^H} NMR (100 MHz, CDCl_3_, selected data from HSQC experiment): δ 100.9 (C-1), 97.8 (C-1′), 80.4 (C-3), 78.1 (C-4′), 75.4 (C-4), 75.0 (C-5), 73.9 (CH_2_(Bn)), 72.9 (C-2), 72.7 (CH_2_(Bn)), 71.1 (C-3′), 70.4 (C-5′), 68.0 (C-6), 66.1 (C-6′), 61.2 (CH_2_CH_2_C_8_F_17_), 56.5 (C-2′); ESI MS; *m*/*z*: calcd for C_59_H_64_F_17_N_2_O_13_Si: 1359.4; found, 1359.5 [*M* + NH_4_]^+^.

Donor **25** (57 mg, 0.078 mmol) and acceptor **23** (31 mg, 0.023 mmol) were dissolved in dry CH_2_Cl_2_ (1.5 mL) in the presence of activated 4Å MS (100 mg). The reaction mixture was stirred, under an argon atmosphere, for 10 min at rt, and TBSOTf (101 μL of a 0.16 M solution in dry CH_2_Cl_2_) was added. After stirring for 1 h at rt, the reaction mixture was quenched with triethylamine (0.2 mL), filtered, and then concentrated under reduced pressure. The crude product was purified by F-SPE. A second glycosylation cycle with additional donor **25** (55 mg, 0.075 mmol) was run to complete the reaction. F-SPE purification gave trisaccharide **24** (43 mg, 98%). TLC (toluene-EtOAc 12:1) Rf 0.09; ^1^H NMR (400 MHz, CDCl_3_): δ 7.94–6.95 (m, 34H, Ar), 5.25 (d, 1H, *J*_1,2_ = 8.4 Hz, H-1B), 5.20 (t, 1H, *J*_3,4_ = *J*_4,5_ = 9.1 Hz, H-4C), 5.13 (dd, 1H, *J*_1,2_ = 7.8 Hz, *J*_2,3_ = 8.8 Hz, H-2A), 5.09–5.02 (m, 2H, H-1C, H-2C), 4.75 (d, 1H, *J* = 11.8 Hz, CH_2_(Bn)), 4.63 (d, 1H, *J* = 11.8 Hz, CH_2_(Bn)), 4.56 (dd, 1H, *J* = 8.5 Hz, *J* = 10.4 Hz, H-3B), 4.46–4.26 (m, 8H, 6x CH_2_(Bn), H-1A, H-2B), 4.07–3.93 (m, 4H, H-4A, H-6B, H-4B, CH_2_CH_2_C_8_F_17_), 3.68–3.55 (m, 7H, 2x H-6C, H-3A, H-3C, H-5C, H-6B, CH_2_CH_2_C_8_F_17_), 3.41–3.20 (m, 4H, 2x H-6A, H-5A, H-5B), 2.52–2–23 (m, 6H, CH_2_(Lev), CH_2_CH_2_C_8_F_17_), 2.08 (s, 3H, CH_3_(Lev)), 1.05, 0.98 (2s, 18H, Si­(C­(CH_3_)_3_)_2_); ^13^C­{^1^H} NMR (100 MHz, CDCl_3_, selected data from the HSQC experiment): δ 101.1 (C-1A), 99.3 (C-1C), 97.7 (C-1B), 80.0 (C-3C, C-3A), 77.7 (C-3B), 77.3 (C-4B), 75.4 (C-4A), 74.8 (C-5A), 74.2 (C-2C, CH_2_(Bn)), 73.6 (CH_2_(Bn)), 72.9 (C-2A, CH_2_(Bn), C-5C), 71.7 (C-4C), 70.4 (C-6C), 70.3 (C-5B), 68.2 (C-6A), 66.1 (C-6B), 61.3 (CH_2_CH_2_C_8_F_17_), 56.0 (C-2B); ESI MS; *m*/*z*: calcd for C_91_H_96_F_17_N_2_O_21_Si: 1903.6; found, 1903.7 [*M* + NH_4_]^+^.

Compound **24** (43 mg, 0.023 mmol) was dissolved in CH_2_Cl_2_ (1.0 mL), and hydrazine monohydrate (91 μL of a 0.5 M solution in Py/AcOH 3:2) was added. After stirring at room temperature for 1 h, the reaction mixture was quenched with acetone (0.2 mL). The mixture was diluted with CH_2_Cl_2_ and washed with a 1 M HCl aqueous solution, saturated NaHCO_3_ aqueous solution, and H_2_O. The organic layer was dried (MgSO_4_), filtered, and concentrated in vacuo. The crude product was purified by F-SPE to give trisaccharide **26** (41 mg, quantitative). TLC (toluene-EtOAc 6:1) Rf 0.48; ^1^H NMR (400 MHz, CDCl_3_): δ 7.94–6.97 (m, 34H, Ar), 5.25 (d, 1H, *J*_1,2_ = 8.5 Hz, H-1B), 5.13 (dd, 1H, *J*_1,2_ = 7.9 Hz, *J*_2,3_ = 8.8 Hz, H-2A), 5.02–4.96 (m, 2H, H-1C, H-2C), 4.76 (d, 1H, *J* = 11.7 Hz, CH_2_(Bn)), 4.65–4.23 (m, 10H, 7x CH_2_(Bn), H-1A, H-2B, H-3B), 4.05 (t, 1H, *J*_3,4_ = *J*_4,5_ = 8.8 Hz, H-4A), 4.00–3.92 (m, 3H, H-6B, H-4B, CH_2_CH_2_C_8_F_17_), 3.89–3.83 (m, 2H, H-4C, H-6C), 3.71 (dd, 1H, *J*_5,6_ = 6.6 Hz, *J*_6,6_ = 9.6 Hz, H-6C), 3.67–3.55 (m, 3H, H-3A, H-6B, CH_2_CH_2_C_8_F_17_), 3.50–3.45 (m, 2H, H-3C, H-5C), 3.40–3.19 (m, 4H, 2x H-6A, H-5A, H-5B), 2.28 (m, 2H, CH_2_CH_2_C_8_F_17_), 1.03, 0.97 (2s, 18H, Si­(C­(CH_3_)_3_)_2_); ^13^C­{^1^H} NMR (100 MHz, CDCl_3_, selected data from the HSQC experiment): δ 101.0 (C-1A), 99.5 (C-1C), 97.7 (C-1B), 82.1 (C-3C), 79.9 (C-3A), 77.9 (C-3B), 77.5 (C-4B), 75.2 (C-4A), 74.9 (C-5A), 74.0 (C-2C, CH_2_(Bn)), 73.6 (C-4C), 73.1 (C-5C), 72.7 (C-2A, CH_2_(Bn)), 71.5 (C-6C), 70.1 (C-5B), 68.1 (C-6A), 66.0 (C-6B), 61.2 (CH_2_CH_2_C_8_F_17_), 56.0 (C-2B); ESI MS; *m*/*z*: calcd for C_87_H_87_F_17_NO_21_Si: 1832.5; found, 1832.9 [*M* + HCO_2_]^−^.

Donor **21** (56 mg, 0.080 mmol) and acceptor **26** (41 mg, 0.023 mmol) were dissolved in dry CH_2_Cl_2_ (1.5 mL) in the presence of activated 4Å MS (120 mg). The reaction mixture was stirred, under an argon atmosphere, for 10 min at rt, and TBSOTf (50 μL of a 0.11 M solution in dry CH_2_Cl_2_) was added. After stirring for 30 min at rt, the reaction mixture was quenched with triethylamine (0.2 mL), filtered, and then concentrated under reduced pressure. The crude product was first purified by F-SPE. The fluorous fraction was then purified by standard silica gel column chromatography (toluene-EtOAc 14:1) to afford pure **27** (27 mg, 51%; 36% from **19**, 6 steps, 85% average yield per step) as a white, amorphous solid. TLC (toluene-EtOAc 14:1) Rf 0.15; [α][Bibr ref20]_D_ +4° (*c* 1, CHCl_3_); ^1^H NMR (400 MHz, CDCl_3_): δ 7.93–7.01 (m, 38H, Ar), 5.59 (dd, 1H, *J*_2,3_ = 10.7 Hz, *J*_3,4_ = 8.9 Hz, H-3D), 5.42 (d, 1H, *J*_1,2_ = 8.2 Hz, H-1D), 5.20 (d, 1H, *J*_1,2_ = 8.2 Hz, H-1B), 5.11 (t, 1H, *J*_1,2_ = *J*_2,3_ = 8.2 Hz, H-2A), 4.92 (t, 1H, *J*_1,2_ = *J*_2,3_ = 8.0 Hz, H-2C), 4.79 (d, 1H, H-1C), 4.74–4.25 (m, 10H, 8x CH_2_(Bn), H-1A, H-3B), 4.21–4.17 (m, 2H, H-2B, H-4C), 4.11 (dd, 1H, H-2D), 4.01 (t, 1H, *J*_3,4_ = *J*_4,5_ = 8.7 Hz, H-4A), 3.97–3.85 (m, 3H, H-6B, H-4B, CH_2_CH_2_C_8_F_17_), 3.80 (dd, 1H, *J*_5,6_ = 5.1 Hz, *J*_6,6_ = 10.2 Hz, H-6D), 3.70 (t, 1H, H-4D), 3.65–3.49 (m, 5H, H-3A, H-3C, H-6B, H-6C, CH_2_CH_2_C_8_F_17_), 3.41–3.36 (m, 3H, H-6A, H-6C, H-6D), 3.28–3.16 (m, 5H, H-6A, H-5A, H-5B, H-5C, H-5D), 2.60–2.37 (m, 4H, CH_2_(Lev)), 2.27 (m, 2H, CH_2_CH_2_C_8_F_17_), 1.94 (s, 3H, CH_3_(Lev)), 0.97–0.85 (4s, 36H, Si­(C­(CH_3_)_3_)_2_); ^13^C­{^1^H} NMR (100 MHz, CDCl_3_): δ 205.7, 171.8, 168.2, 167.7, 165.1, 164.8 (CO), 138.2–123.4 (Ar), 100.9 (C-1A), 99.7 (C-1C), 97.6, 97.3 (C-1B, C-1D), 80.3 (C-3C), 79.9 (C-3A), 77.8 (C-3B), 77.2 (C-4B), 75.6 (C-4D), 75.3 (C-4A), 75.1, 74.8, 74.6 (C-5A, C-5C, C-4C), 74.4 (C-2C), 74.0, 73.6 (2x CH_2_(Bn)), 72.8, 72.6, 72.3 (C-2A, C-3D, 2x CH_2_(Bn)), 70.3, 70.1 (C-5B, C-5D), 68.1, 68.0 (C-6A, C-6C), 65.9, 65.8 (C-6B, C-6D), 61.2 (CH_2_CH_2_C_8_F_17_), 56.0 (C-2B), 55.3 (C-2D), 37.9 (CH_2_(Lev)), 31.4 (t, CH_2_CH_2_C_8_F_17_), 29.7 (CH_3_(Lev)), 27.9 (CH_2_(Lev)), 27.3, 27.2, 27.0, 26.8 (Si­(C­(CH_3_)_3_)_2_), 22.5, 22.4, 19.8 (Si­(C­(CH_3_)_3_)_2_); ^19^F NMR (376 MHz, CDCl_3_): δ −80.7 (t, 3F), −113.4 (m, 2F), −122.0 (m, 6F), −122.7 (m, 2F), −123.7 (m, 2F), −126.1 (m, 2F); HR MS; *m*/*z*: calcd for C_113_H_121_F_17_O_27_N_2_Si_2_Na: 2339.7316; found, 2339.7287 [*M* + Na]^+^.

#### 1H,1H,2H,2H-Perfluorodecyl *O*-(2-Deoxy-3-*O*-levulinoyl-2-phthalimido-4,6-di-*O*-sulfo-β-d-glucopyranosyl)-(1 → 4)-*O*-(2-*O*-Benzoyl-3,6-di-*O*-benzyl-β-d-glucopyranosyl)-(1 → 3)-*O*-(2-Deoxy-2-phthalimido-4,6-di-*O*-sulfo-β-d-glucopyranosyl)-(1 → 4)-2-*O*-Benzoyl-3,6-di-*O*-benzyl-β-d-glucopyranoside (**28**)

An excess of (HF)_*n*_·Py (0.063 mL, 2.4 mmol) was added at 0 °C under an argon atmosphere to a solution of **27** (28 mg, 12.1 μmol) in dry THF (1.5 mL). After 18 h at 4 °C, the mixture was diluted with CH_2_Cl_2_ and washed with H_2_O, saturated NaHCO_3_ solution, and H_2_O. The organic layers were dried (MgSO_4_), filtered, and concentrated in vacuo to give the corresponding tetraol (25 mg) as a white amorphous solid that was directly submitted to the sulfation reaction. TLC (toluene/acetone 2:1) Rf 0.39.

The tetraol (25 mg, 12.1 μmol) and sulfur trioxide–trimethylamine complex (68 mg, 0.49 mmol) were dissolved in dry DMF (1.5 mL) and heated at 100 °C for 30 min using microwave radiation (20 W average power). The reaction vessel was cooled, and Et_3_N (250 μL) was added. The crude solution was purified by Sephadex LH 20 chromatography (CH_2_Cl_2_-MeOH 1:1) and silica gel column chromatography (EtOAc-MeOH-H_2_O 34:5:3 → 24:5:3). Finally, the residue was eluted from a Dowex 50WX2-Na^+^ column (MeOH as eluent) to obtain **28** as a sodium salt (19 mg, 63%, 2 steps, white amorphous solid). TLC (EtOAc-MeOH-H_2_O 24:5:3) Rf 0.31; ^1^H NMR (400 MHz, CD_3_OD): δ 7.95–7.02 (m, 38H, Ar), 5.80 (dd, 1H, *J* = 10.8 Hz, *J* = 9.0 Hz, H-3D), 5.49 (d, 1H, *J*_1,2_ = 8.4 Hz, H-1D), 5.32 (d, 1H, *J*_1,2_ = 8.2 Hz, H-1B), 5.03–4.96 (m, 2H, H-2A, H-2C), 4.86 (m, 1H, H-3B), 4.81–4.79 (m, 2H, 2x CH_2_(Bn)), 4.72–4.67 (m, 2H, H-6B, H-1A or C), 4.62–4.52 (m, 5H, 4x CH_2_(Bn), H-1A or C), 4.48–4.27 (m, 5H, 2x CH_2_(Bn), H-4B, H-4D, H-6D), 4.22–4.11 (m, 4H, H-2B, H-2D, H-6B, H-4A or C), 4.06–3.95 (m, 5H, H-6D, H-5B, H-4A or C, H-3A or C, CH_2_CH_2_C_8_F_17_), 3.76–3.46 (m, 8H, H-5A or C, H-3A or C, H-5D, CH_2_CH_2_C_8_F_17_, 2x H-6A, 2x H-6C), 3.33 (m, 1H, H-5A or C), 2.58–2.26 (m, 6H, CH_2_(Lev), CH_2_CH_2_C_8_F_17_), 1.88 (s, 3H, CH_3_(Lev)); ^13^C­{^1^H} NMR (100 MHz, CD_3_OD): δ 207.4, 172.5, 165.8, 165.6 (CO), 138.3–123.1 (Ar), 100.5, 99.8 (C-1A, C-1C), 96.6 (C-1D), 95.6 (C-1B), 79.8, 78.0 (C-3A, C-3C), 76.5 (C-4B), 75.4 (C-3B), 74.7, 74.6 (3C: C-4D, C-4A or C, C-5A or C), 74.3–73.2 (6C: C-2A, C-2C, C-5A or C, C-5B, C-4A or C, C-5D), 72.7, 72.3 (4x CH_2_(Bn)), 70.5 (C-3D), 68.2, 67.5 (C-6A, C-6C), 67.3, 66.5 (C-6B, C-6D), 61.1 (CH_2_CH_2_C_8_F_17_), 56.3, 55.0 (C-2B, C-2D), 37.1 (CH_2_(Lev)), 30.7 (t, *J*_C,F_ = 21.4 Hz, CH_2_CH_2_C_8_F_17_), 27.83, 27.75 (CH_3_(Lev), CH_2_(Lev)); ^19^F NMR (376 MHz, CD_3_OD): δ −82.4 (t, 3F), −114.3 (m, 2F), −123.0 (m, 6F), −123.8 (m, 2F), −124.6 (m, 2F), −127.3 (m, 2F); HR MS; *m*/*z*: calcd for C_97_H_85_F_17_O_39_N_2_S_4_Na_3_: 2421.3039; found, 2421.3009 [*M* – Na]^−^.

#### 1H,1H,2H,2H-Perfluorodecyl *O*-(2-Benzamido-2-deoxy-4,6-di-*O*-sulfo-β-d-glucopyranosyl)-(1 → 4)-*O*-(3,6-di-*O*-Benzyl-β-d-glucopyranosyl)-(1 → 3)-*O*-(2-Benzamido-2-deoxy-4,6-di-*O*-sulfo-β-d-glucopyranosyl)-(1 → 4)-3,6-di-*O*-Benzyl-β-d-glucopyranoside (**3**)

Ethylene diamine (148 μL, 2.21 mmol) was added, under an argon atmosphere, to a solution of **28** (18 mg, 7.4 μmol) in a mixture of *n*-BuOH (1.5 mL), MeOH (0.6 mL), and 2,2,2-trifluoroethanol (0.2 mL). Then, the reaction mixture was subjected to microwave irradiation (25 W average power) for 90 min at 120 °C (3 cycles, 30 min each). The reaction vessel was cooled, and the mixture was concentrated to dryness. The residue was dissolved in MeOH/CF_3_CH_2_OH (2.0 mL/0.1 mL), and an aqueous solution of NaOH (4 M, 370 μL) was added. After stirring for 24 h at room temperature, the reaction mixture was neutralized with Amberlite IR-120 (H^+^) resin, filtered, and concentrated to give the desired amine intermediate. Triethylamine (27 μL, 0.19 mmol) and benzoic anhydride (67 mg, 0.29 mmol) were added to a cooled (0 °C) solution of this amine derivative in MeOH (2 mL). After the mixture was stirred for 4 h at room temperature, additional triethylamine (27 μL, 0.19 mmol) and benzoic anhydride (67 mg, 0.29 mmol) were added at 0 °C. After further stirring for 3 h at room temperature, Et_3_N (0.25 mL) was added, and the mixture was concentrated to dryness. The residue was then purified by silica gel column chromatography (EtOAc-MeOH-H_2_O 32:5:3 → 24:5:3) and finally eluted from a Dowex 50WX2-Na^+^ column (MeOH) to obtain **3** as a sodium salt (13 mg, 87%, white amorphous solid). TLC (EtOAc-MeOH-H_2_O 24:5:3) Rf 0.24; ^1^H NMR (400 MHz, CD_3_OD): δ 7.86–7.19 (m, 30H, Ar), 5.08 (d, 1H, *J*_1,2_ = 7.8 Hz, H-1B), 4.98 (d, 1H, *J* = 11.4 Hz, CH_2_(Bn)), 4.88 (m, 3H, CH_2_(Bn)), 4.83 (d, 1H, *J*_1,2_ = 8.0 Hz, H-1D), 4.61 (m, 2H, H-1A or C, H-6B), 4.45 (m, 5H, H-3B, H-6D, 3x CH_2_(Bn)), 4.35 (m, 3H, CH_2_(Bn), H-1A or C, H-4B), 4.18–4.03 (m, 4H, CH_2_CH_2_C_8_F_17,_ H-6B, H-4D, H-6D), 3.98–3.83 (m, 6H, CH_2_CH_2_C_8_F_17,_ H-4A, H-4C, H-2B, H-2D, H-3D), 3.76–3.58 (m, 8H, 2x H-6A, 2x H-6C, H-5B, H-3A, H-3C, H-5A or C), 3.52 (m, 3H, H-2A or C, H-5A or C, H-5D), 3.40 (t, 1H, *J*_1,2_ = *J*_2,3_ = 8.4 Hz, H-2A or C), 2.56 (m, 2H, CH_2_CH_2_C_8_F_17_); ^13^C­{^1^H} NMR (100 MHz, CD_3_OD): δ 169.0 (CO), 139.3–126.7 (Ar), 103.0, 100.9 (C-1A, C-1C), 99.2, 98.9 (C-1B, C-1D), 82.0, 81.4 (C-3A, C-3C), 77.1, 76.7 (C-4D, C-3B), 75.9, 75.4 (C-4A, C-4C), 74.8–72.4 (C-2A, C-2C, C-5A, C-5C, C-5B, C-5D, C-3D, C-4B, 4x CH_2_(Bn)), 69.4, 68.8 (C-6A, C-6C), 67.3, 66.9 (C-6B, C-6D), 61.2 (CH_2_CH_2_C_8_F_17_), 56.6, 56.2 (C-2B, C-2D), 31.2 (CH_2_CH_2_C_8_F_17_); ^19^F NMR (376 MHz, CD_3_OD): δ −82.3 (t, 3F), −114.4 (m, 2F), −122.9 (m, 6F), −123.7 (m, 2F), −124.6 (m, 2F), −127.2 (m, 2F); HR MS; *m*/*z*: calcd for C_76_H_75_F_17_O_33_N_2_S_4_Na_3_: 2063.2562; found, 2063.2597 [*M* – Na]^−^.

#### 1H,1H,2H,2H-Perfluorodecyl *O*-(2-Deoxy-3-*O*-levulinoyl-2-phthalimido-4,6-di-*O*-sulfo-β-d-glucopyranosyl)-(1 → 4)-2-*O*-Benzoyl-3,6-di-*O*-benzyl-β-d-glucopyranoside (**29**)

An excess of (HF)_*n*_·Py (0.296 mL, 11.4 mmol) was added at 0 °C under an argon atmosphere to a solution of **22** (82 mg, 56.9 μmol) in dry THF (2.5 mL). After 21 h at 4 °C, the mixture was diluted with CH_2_Cl_2_ and washed with H_2_O, saturated NaHCO_3_ solution, and H_2_O. The organic layers were dried (MgSO_4_), filtered, and concentrated in vacuo to give the corresponding diol (72 mg) as a white amorphous solid that was directly submitted to the sulfation reaction. TLC (toluene-acetone 2:1) Rf 0.48.

The diol (72 mg, 55.4 μmol) and sulfur trioxide–trimethylamine complex (154 mg, 1.11 mmol) were dissolved in dry DMF (3 mL) and heated at 100 °C for 30 min using microwave radiation (15 W average power). The reaction vessel was cooled, and Et_3_N (300 μL) was added. The crude solution was purified by Sephadex LH 20 chromatography (CH_2_Cl_2_-MeOH 1:1) and silica gel column chromatography (EtOAc-MeOH-H_2_O 56:5:3). Finally, the residue was eluted from a Dowex 50WX2-Na^+^ column (MeOH as eluent) to obtain **29** as a sodium salt (71 mg, 83%, 2 steps, white amorphous solid). TLC (EtOAc-MeOH-H_2_O 36:5:3) Rf 0.27; ^1^H NMR (400 MHz, CD_3_OD): δ 7.98–7.10 (m, 19H, Ar), 5.90 (dd, 1H, *J*_2,3_ = 11.0 Hz, *J*_3,4_ = 8.6 Hz, H-3′), 5.67 (d, 1H, *J*_1,2_ = 8.4 Hz, H-1′), 5.10 (dd, 1H, *J*_1,2_ = 8.0 Hz, *J*_2,3_ = 8.8 Hz, H-2), 4.92 (d, 1H, *J* = 12.0 Hz, CH_2_(Bn)), 4.67 (m, 2H, H-1, CH_2_(Bn)), 4.57 (dd, 1H, *J*_5,6_ = 2.0 Hz, *J*_6,6_ = 11.2 Hz, H-6′), 4.49 (2d, 2H, *J* = 12.2 Hz, CH_2_(Bn)), 4.38 (dd, 1H, H-4′), 4.25 (dd, 1H, H-2′), 4.20–4.10 (m, 2H, H-4, H-6′), 4.06–4.01 (m, 2H, H-3, CH_2_CH_2_C_8_F_17_), 3.79–3.76 (m, 2H, H-5′, CH_2_CH_2_C_8_F_17_), 3.71–3.64 (m, 3H, 2x H-6, H-5), 2.63–2.30 (m, 6H, CH_2_(Lev), CH_2_CH_2_C_8_F_17_), 1.90 (s, 3H, CH_3_(Lev)); ^13^C­{^1^H} NMR (100 MHz, CD_3_OD): δ 207.5, 172.6, 165.7 (CO), 138.2–126.8 (Ar), 100.6 (C-1), 96.0 (C-1′), 78.6 (C-3), 74.9 (C-4), 74.7 (C-4′), 74.3 (C-5), 73.3, 73.2 (C-2, C-5′), 72.7 72.6 (2x CH_2_(Bn)), 70.5 (C-3′), 68.1 (C-6), 66.7 (C-6′), 61.2 (CH_2_CH_2_C_8_F_17_), 55.1 (C-2′), 37.1 (CH_2_(Lev)), 30.9 (CH_2_CH_2_C_8_F_17_), 27.9 (CH_3_(Lev)), 27.8 (CH_2_(Lev)); ^19^F NMR (376 MHz, CD_3_OD): δ −82.4 (t, 3F), −114.2 (m, 2F), −123.0 (m, 6F), −123.7 (m, 2F), −124.6 (m, 2F), −127.3 (m, 2F); HR MS; *m*/*z*: calcd for C_56_H_48_F_17_O_21_NS_2_Na: 1480.1792; found, 1480.1797 [*M* – Na]^−^.

#### 1H,1H,2H,2H-Perfluorodecyl *O*-(2-Amino-2-deoxy-4,6-di-*O*-sulfo-β-d-glucopyranosyl)-(1 → 4)-3,6-Di-*O*-benzyl-β-d-glucopyranoside (**4**)

Ethylene diamine (473 μL, 7.08 mmol) was added, under an argon atmosphere, to a solution of **29** (71 mg, 47.2 μmol) in a mixture of *n*-BuOH (2.5 mL), MeOH (1 mL), and 2,2,2-trifluoroethanol (0.25 mL). Then, the reaction mixture was subjected to microwave irradiation (20 W average power) for 90 min at 120 °C (3 cycles, 30 min each). The reaction vessel was cooled, and the mixture was concentrated to dryness. The residue was dissolved in MeOH/CF_3_CH_2_OH (5.5 mL/0.3 mL), and an aqueous solution of NaOH (4 M, 1 mL) was added. After being stirred for 18 h at room temperature, the reaction mixture was neutralized with Amberlite IR-120 (H^+^) resin, filtered, and concentrated to give the crude amine disaccharide **4** (77 mg). A portion of this crude material (20 mg, 0.0123 mmol) was purified by silica gel column chromatography (EtOAc-MeOH-H_2_O 56:5:3 → 48:5:3) and finally eluted from a Dowex 50WX2-Na^+^ column (MeOH) to obtain **4** as a sodium salt (12 mg, 86%, white amorphous solid). TLC (EtOAc-MeOH-H_2_O 32:5:3) Rf 0.32; ^1^H NMR (400 MHz, CD_3_OD): δ 7.52–7.27 (m, 10H, Ar), 4.93 (2d, 2H, CH_2_(Bn)), 4.77 (d, 1H, *J*_1,2_ = 8.4 Hz, H-1′), 4.71–4.61 (2d, 2H, *J* = 12.0 Hz, CH_2_(Bn)), 4.50 (dd, 1H, *J*_5,6_ = 2.0 Hz, *J*_6,6_ = 11.4 Hz, H-6′), 4.46 (d, 1H, *J*_1,2_ = 7.6 Hz, H-1), 4.19–4.11 (m, 2H, H-4, CH_2_CH_2_C_8_F_17_), 4.09–3.92 (m, 3H, H-4′, H-6′, CH_2_CH_2_C_8_F_17_), 3.84–3.74 (m, 5H, 2x H-6, H-3′, H-3, H-5), 3.62 (m, 1H, H-5′), 3.52 (dd, 1H, *J*_2,3_ = 8.8 Hz, H-2), 2.91 (dd, 1H, *J*_2,3_ = 10.4 Hz, H-2′), 2.60 (m, 2H, CH_2_CH_2_C_8_F_17_); ^13^C­{^1^H} NMR (100 MHz, CD_3_OD): δ 138.9–127.0 (Ar), 103.1 (C-1), 95.9 (C-1′), 80.2 (C-3), 76.2 (C-4′), 74.3, 74.1 (C-4, C-5), 73.5, 73.2, 73.0 (C-2, C-5′, CH_2_(Bn)), 72.0 (CH_2_(Bn)), 71.5 (C-3′), 68.6 (C-6), 66.6 (C-6′), 61.3 (CH_2_CH_2_C_8_F_17_), 56.6 (C-2′), 31.3 (CH_2_CH_2_C_8_F_17_); ^19^F NMR (376 MHz, CD_3_OD): δ −82.4 (t, 3F), −114.3 (m, 2F), −122.9 (m, 6F), −123.7 (m, 2F), −124.5 (m, 2F), −127.3 (m, 2F); HR MS; *m*/*z*: calcd for C_36_H_37_F_17_O_16_NS_2_: 1126.1288; found, 1126.1254 [*M*-2Na + H]^−^.

#### 1H,1H,2H,2H-Perfluorodecyl *O*-(2-Benzamide-2-deoxy-4,6-di-*O*-sulfo-β-d-glucopyranosyl)-(1 → 4)-3,6-Di-*O*-benzyl-β-d-glucopyranoside (**5**)

Triethylamine (37 μL, 0.27 mmol) and benzoic anhydride (92 mg, 0.41 mmol) were added to a cooled (0 °C) solution of crude amine disaccharide **4** (20.4 μmol) in MeOH (2 mL). After stirring for 3.5 h at room temperature, Et_3_N (0.25 mL) was added, and the mixture was concentrated to dryness. The residue was then purified by silica gel column chromatography (EtOAc-MeOH-H_2_O 48:5:3 → 40:5:3) and finally eluted from a Dowex 50WX2-Na^+^ column (MeOH) to obtain **5** as a sodium salt (22 mg, 85%, white amorphous solid). TLC (EtOAc-MeOH-H_2_O 32:5:3) Rf 0.32; ^1^H NMR (400 MHz, CD_3_OD): δ 7.88–7.23 (m, 15H, Ar), 5.05 (d, 1H, *J* = 11.2 Hz, CH_2_(Bn)), 4.98–4.94 (m, 2H, H-1′, CH_2_(Bn)), 4.51–4.45 (m, 2H, H-6′, CH_2_(Bn)), 4.40–4–35 (m, 2H, H-1, CH_2_(Bn)), 4.18–3.96 (m, 5H, H-4′, CH_2_CH_2_C_8_F_17,_ H-6′, H-2′, H-3′), 3.90 (m, 2H, H-4, CH_2_CH_2_C_8_F_17_), 3.79 (m, 1H, H-6), 3.71 (t, 1H, H-3), 3.63 (m, 3H, H-6, H-5, H-5′), 3.43 (t, 1H, *J*_1,2_ = *J*_2,3_ = 8.2 Hz, H-2), 2.56 (m, 2H, CH_2_CH_2_C_8_F_17_); ^13^C­{^1^H} NMR (100 MHz, CD_3_OD): δ 169.2 (CO), 139.4–126.7 (Ar), 103.0 (C-1), 99.6 (C-1′), 82.1 (C-3), 77.1 (C-4′), 76.0 (C-4), 74.5 (C-5), 73.7 (C-2), 73.0–72.8 (C-3′, C-5′, 2x CH_2_(Bn)), 69.1 (C-6), 66.9 (C-6′), 61.2 (CH_2_CH_2_C_8_F_17_), 56.7 (C-2′), 31.2 (CH_2_CH_2_C_8_F_17_); ^19^F NMR (376 MHz, CD_3_OD): δ −82.4 (t, 3F), −114.4 (m, 2F), −122.7 (m, 6F), −123.7 (m, 2F), −124.6 (m, 2F), −127.3 (m, 2F); HR MS; *m*/*z*: calcd for C_43_H_40_F_17_O_17_NS_2_Na: 1252.1369; found, 1252.1364 [*M*-Na]^−^.

#### 1H,1H,2H,2H-Perfluorodecyl *O*-(2-Acetamide-2-deoxy-4,6-di-*O*-sulfo-β-d-glucopyranosyl)-(1 → 4)-3,6-Di-*O*-benzyl-β-d-glucopyranoside (**6**)

Triethylamine (25 μL, 0.18 mmol) and acetic anhydride (26 μL, 0.27 mmol) were added to a cooled (0 °C) solution of crude amine **4** (13.6 μmol) in MeOH (1.5 mL). After stirring for 3.5 h at room temperature, Et_3_N (0.25 mL) was added and the mixture was concentrated to dryness. The residue was then purified by silica gel column chromatography (EtOAc-MeOH-H_2_O 48:5:3 → 40:5:3 → 32:5:3) and finally eluted from a Dowex 50WX2-Na^+^ column (MeOH) to obtain **6** as a sodium salt (13 mg, 81%, white amorphous solid). TLC (EtOAc-MeOH-H_2_O 32:5:3) Rf 0.21; ^1^H NMR (400 MHz, CD_3_OD): δ 7.53–7.22 (m, 10H, Ar), 5.05–4.93 (2d, 2H, *J* = 11.6 Hz, CH_2_(Bn)), 4.76 (d, 1H, *J*_1,2_ = 8.4 Hz, H-1′), 4.69–4.60 (2d, 2H, *J* = 12.0 Hz, CH_2_(Bn)), 4.47 (dd, 1H, *J*_5,6_ = 2.0 Hz, *J*_6,6_ = 11.2 Hz, H-6′), 4.39 (d, 1H, *J*_1,2_ = 8.0 Hz, H-1), 4.15 (m, 1H, CH_2_CH_2_C_8_F_17_), 4.09–3.98 (m, 2H, H-4′, H-6′), 3.94–3.90 (m, 2H, CH_2_CH_2_C_8_F_17,_ H-4), 3.85–3.63 (m, 6H, 2x H-6, H-2′, H-3′, H-3, H-5), 3.52 (m, 1H, H-5′), 3.43 (t, 1H, *J*_2,3_ = 8.6 Hz, H-2), 2.60 (m, 2H, CH_2_CH_2_C_8_F_17_), 1.93 (s, 3H, NHAc); ^13^C­{^1^H} NMR (100 MHz, CD_3_OD): δ 172.2 (CO), 139.4–126.7 (Ar), 103.1 (C-1), 99.3 (C-1′), 81.9 (C-3), 76.9 (C-4′), 75.6 (C-4), 74.6 (C-5), 73.7 (C-2), 73.0–72.7 (C-3′, C-5′, 2 x CH_2_(Bn)), 68.8 (C-6), 66.9 (C-6′), 61.2 (CH_2_CH_2_C_8_F_17_), 56.2 (C-2′), 31.3 (CH_2_CH_2_C_8_F_17_), 21.7 (NHAc); ^19^F NMR (376 MHz, CD_3_OD): δ −82.4 (t, 3F), −114.4 (m, 2F), −122.9 (m, 6F), −123.7 (m, 2F), −124.6 (m, 2F), −127.3 (m, 2F); HR MS; *m*/*z*: calcd for C_38_H_38_F_17_O_17_NS_2_Na: 1190.1213; found, 1190.1213 [*M*-Na]^−^.

#### 1H,1H,2H,2H-Perfluorodecyl *O*-[2-Deoxy-4,6-di-*O*-sulfo-2-(4-trifluoromethylbenzamide)-β-d-glucopyranosyl]-(1 → 4)-3,6-Di-*O*-benzyl-β-d-glucopyranoside (**7**)

Triethylamine (19 μL, 0.14 mmol) and 4-(trifluoromethyl)­benzoic anhydride (75 mg, 0.21 mmol) were added to a cooled (0 °C) solution of crude disaccharide **4** (10.4 μmol) in MeOH (1.5 mL). After stirring for 3.5 h at room temperature, Et_3_N (0.25 mL) was added and the mixture was concentrated to dryness. The residue was purified by silica gel column chromatography (EtOAc-MeOH-H_2_O 56:5:3 → 48:5:3) and then eluted from a Dowex 50WX2-Na^+^ column (MeOH) to obtain **7** as a sodium salt (11 mg, 79%, white amorphous solid). TLC (EtOAc-MeOH-H_2_O 32:5:3) Rf 0.33; ^1^H NMR (400 MHz, CD_3_OD): δ 7.99–7.22 (m, 14H, Ar), 5.04 (d, 1H, *J* = 11.6 Hz, CH_2_(Bn)), 4.98–4.95 (m, 2H, H-1′, CH_2_(Bn)), 4.52–4.49 (m, 2H, H-6′, CH_2_(Bn)), 4.43–4–37 (m, 2H, H-1, CH_2_(Bn)), 4.17–4.10 (m, 2H, H-4′, CH_2_CH_2_C_8_F_17_), 4.08–3.87 (m, 5H, H-6′, H-2′, H-3′, H-4, CH_2_CH_2_C_8_F_17_), 3.80–3.60 (m, 5H, 2x H-6, H-3, H-5, H-5′), 3.44 (t, 1H, *J*_1,2_ = *J*_2,3_ = 8.2 Hz, H-2), 2.56 (m, 2H, CH_2_CH_2_C_8_F_17_); ^13^C­{^1^H} NMR (100 MHz, CD_3_OD): δ 167.8 (CO), 139.4–125.0 (Ar), 103.0 (C-1), 99.2 (C-1′), 81.9 (C-3), 77.1 (C-4′), 75.8 (C-4), 74.6 (C-5), 73.7 (C-2), 72.8–72.7 (C-3′, C-5′, 2x CH_2_(Bn)), 69.1 (C-6), 66.9 (C-6′), 61.2 (CH_2_CH_2_C_8_F_17_), 56.9 (C-2′), 31.3 (CH_2_CH_2_C_8_F_17_); ^19^F NMR (376 MHz, CD_3_OD): δ −64.4 (s, 3F), −82.4 (t, 3F), −114.4 (m, 2F), −122.9 (m, 6F), −123.7 (m, 2F), −124.6 (m, 2F), −127.3 (m, 2F); HR MS; *m*/*z*: calcd for C_44_H_40_F_20_O_17_NS_2_: 1298.1424; found, 1298.1399 [*M*-2Na + H]^−^.

#### 1H,1H,2H,2H-Perfluorodecyl *O*-[2-Deoxy-4,6-di-*O*-sulfo-2-(2-naphthalene Acetamide)-β-d-glucopyranosyl]-(1 → 4)-3,6-Di-*O*-benzyl-β-d-glucopyranoside (**8**)

1,3-Dicyclohexylcarbodiimide (60 mg, 0.29 mmol) was dissolved in CH_2_Cl_2_ (3 mL). 2-Naphthalene acetic acid (109 mg, 0.59 mmol) was then added at 0 °C, and the mixture was stirred for 5 min at room temperature, filtered, and concentrated to give 2-naphthalene acetic anhydride as a white solid (0.29 mmol). A solution containing triethylamine (22 μL, 0.15 mmol) and crude **4** (11.7 μmol) in MeOH (2.5 mL) was added at 0 °C to the anhydride (0.29 mmol). After the mixture was stirred for 4 h at room temperature, Et_3_N (0.25 mL) was added and the mixture was concentrated to dryness. The residue was purified by silica gel column chromatography (EtOAc-MeOH-H_2_O 56:5:3 → 48:5:3) and then eluted from a Dowex 50WX2-Na^+^ column (MeOH) to obtain **8** as a sodium salt (14 mg, 88%, white amorphous solid). TLC (EtOAc-MeOH-H_2_O 32:5:3) Rf 0.27; ^1^H NMR (400 MHz, CD_3_OD): δ 7.84–7.21 (m, 17H, Ar), 5.01–4.78 (2d, 2H, *J* = 11.6 Hz, CH_2_(Bn)), 4.57 (d, 1H, *J*_1,2_ = 8.0 Hz, H-1′), 4.50 (dd, 1H, *J*_5,6_ = 1.6 Hz, *J*_6,6_ = 11.2 Hz, H-6′), 4.40 (2d, 2H, *J* = 12.0 Hz, CH_2_(Bn)), 4.09–4.00 (m, 2H, H-6′, H-4′), 3.94–3.73 (m, 6H, H-4, 2x CH_2_CH_2_C_8_F_17,_ H-1, H-3′, H-2′), 3.72–3.64 (2d, 2H, *J* = 13.6 Hz, CH_2_C­(=O)­NH), 3.51 (m, 1H, H-5′), 3.31 (m, 1H, H-3), 3.25–3.19 (m, 2H, H-2, H-6), 3.13 (dd, 1H, *J*_5,6_ = 1.6 Hz, *J*_6,6_ = 11.2 Hz, H-6), 2.51 (m, 3H, H-5, CH_2_CH_2_C_8_F_17_); ^13^C­{^1^H} NMR (100 MHz, CD_3_OD): δ 172.7 (CO), 139.2–125.4 (Ar), 103.0 (C-1), 98.9 (C-1′), 82.0 (C-3), 77.2 (C-4′), 74.3 (C-4, C-5), 73.4 (CH_2_(Bn)), 73.0–72.7 (C-2, C-3′, C-5′, CH_2_(Bn)), 68.0 (C-6), 66.9 (C-6′), 61.3 (CH_2_CH_2_C_8_F_17_), 56.3 (C-2′), 43.3 (CH_2_C­(=O)­NH), 31.2 (CH_2_CH_2_C_8_F_17_); ^19^F NMR (376 MHz, CD_3_OD): δ −82.3 (t, 3F), −114.3 (m, 2F), −122.8 (m, 6F), −123.7 (m, 2F), −124.5 (m, 2F), −127.3 (m, 2F); HR MS; *m*/*z*: calcd for C_48_H_45_F_17_O_17_NS_2_: 1294.1863; found, 1294.1836 [*M*-2Na + H]^−^.

#### 1H,1H,2H,2H-Perfluorodecyl *O*-(2-Deoxy-4,6-di-*O*-sulfo-2-pivaloylamide-β-d-glucopyranosyl)-(1 → 4)-3,6-Di-*O*-benzyl-β-d-glucopyranoside (**9**)

Triethylamine (41 μL, 0.29 mmol) and pivaloyl chloride (29 μL, 0.23 mmol) were added to a cooled (0 °C) solution of crude **4** (11.7 μmol) in MeOH (1.5 mL). After stirring for 3.5 h at room temperature, Et_3_N (0.25 mL) was added and the mixture was concentrated to dryness. The residue was purified by silica gel column chromatography (EtOAc-MeOH-H_2_O 56:5:3 → 48:5:3) and then eluted from a Dowex 50WX2-Na^+^ column (MeOH) to obtain **9** as a sodium salt (13 mg, 87%, white amorphous solid). TLC (EtOAc-MeOH-H_2_O 32:5:3) Rf 0.31; ^1^H NMR (400 MHz, CD_3_OD): δ 7.54–7.23 (m, 10H, Ar), 5.06–4.88 (2d, 2H, *J* = 11.2 Hz, CH_2_(Bn)), 4.78 (d, 1H, *J*_1,2_ = 8.0 Hz, H-1′), 4.69–4.60 (2d, 2H, *J* = 12.0 Hz, CH_2_(Bn)), 4.53 (dd, 1H, *J*_5,6_ = 2.0 Hz, *J*_6,6_ = 11.2 Hz, H-6′), 4.39 (d, 1H, *J*_1,2_ = 7.6 Hz, H-1), 4.13 (m, 1H, CH_2_CH_2_C_8_F_17_), 4.08–3.99 (m, 2H, H-4′, H-6′), 3.95–3.77 (m, 5H, CH_2_CH_2_C_8_F_17,_ H-4, H-3′, H-6, H-2′), 3.73 (m, 1H, H-6), 3.66–3.62 (m, 3H, H-5, H-5′, H-3), 3.42 (dd, 1H, *J*_2,3_ = 8.8 Hz, H-2), 2.59 (m, 2H, CH_2_CH_2_C_8_F_17_), 1.20 (s, 9H, C­(CH_3_)_3_); ^13^C­{^1^H} NMR (100 MHz, CD_3_OD): δ 180.2 (CO), 139.4–126.7 (Ar), 103.1 (C-1), 99.1 (C-1′), 82.0 (C-3), 77.3 (C-4′), 75.2 (C-4), 75.0 (C-5), 73.3–72.9 (C-2, C-5′, 2x CH_2_(Bn)), 72.6 (C-3′), 69.1 (C-6), 67.1 (C-6′), 61.2 (CH_2_CH_2_C_8_F_17_), 56.4 (C-2′), 38.5 (C­(CH_3_)_3_), 31.3 (CH_2_CH_2_C_8_F_17_), 26.6 (C­(CH_3_)_3_); ^19^F NMR (376 MHz, CD_3_OD): δ −82.3 (t, 3F), −114.4 (m, 2F), −122.9 (m, 6F), −123.7 (m, 2F), −124.6 (m, 2F), −127.3 (m, 2F); HR MS; *m*/*z*: calcd for C_41_H_45_F_17_O_17_NS_2_: 1210.1863; found, 1210.1833 [*M*-2Na + H]^−^.

#### 1H,1H,2H,2H-Perfluorodecyl *O*-(2-Deoxy-3-*O*-levulinoyl-2-phthalimido-6-*O*-sulfo-β-d-glucopyranosyl)-(1 → 4)-2-*O*-Benzoyl-3,6-di-*O*-benzyl-β-d-glucopyranoside (**30**)

An excess of (HF)_*n*_·Py (0.18 mL, 6.94 mmol) was added at 0 °C under an argon atmosphere to a solution of **22** (50 mg, 34.7 μmol) in dry THF (2 mL). After 22 h at 4 °C, the mixture was diluted with CH_2_Cl_2_ and washed with H_2_O, saturated NaHCO_3_ solution, and H_2_O. The organic layers were dried (MgSO_4_), filtered, and concentrated in vacuo to give the corresponding diol (45 mg) that was directly submitted to the sulfation reaction.

The diol (25 mg, 19.2 μmol) and sulfur trioxide–trimethylamine complex (8 mg, 0.058 mmol) were dissolved in dry DMF (1.5 mL) and heated at 45 °C for 30 min using microwave radiation (12 W average power). The reaction vessel was cooled, and Et_3_N (250 μL) was added. The crude solution was purified by Sephadex LH 20 chromatography (CH_2_Cl_2_-MeOH 1:1) and silica gel column chromatography (EtOAc-MeOH-H_2_O 72:5:3). Finally, the residue was eluted from a Dowex 50WX2-Na^+^ column (MeOH as eluent) to obtain **30** as a sodium salt (20 mg, 74%, 2 steps, white amorphous solid). TLC (EtOAc-MeOH-H_2_O 36:5:3) Rf 0.49; ^1^H NMR (400 MHz, CD_3_OD): δ 7.96–7.09 (m, 19H, Ar), 5.67 (m, 2H, H-1′, H-3′), 5.08 (dd, 1H, *J*_2,3_ = 9.2 Hz, H-2), 4.97–4.67 (2d, 2H, *J* = 11.6 Hz, CH_2_(Bn)), 4.61 (d, 1H, *J*_1,2_ = 8.0 Hz, H-1), 4.44 (2d, 2H, *J* = 11.9 Hz, CH_2_(Bn)), 4.25 (dd, 1H, H-6′), 4.17 (m, 3H, H-2′, H-4, H-6′), 4.03 (m, 1H, CH_2_CH_2_C_8_F_17_), 3.93 (t, 1H, *J*_3,4_ = 9.0 Hz, H-3), 3.75 (m, 2H, H-4′, CH_2_CH_2_C_8_F_17_), 3.60–3.53 (m, 3H, 2x H-6, H-5), 3.45 (m, 1H, H-5′), 2.63–2.28 (m, 6H, CH_2_(Lev), CH_2_CH_2_C_8_F_17_), 1.90 (s, 3H, CH_3_(Lev)); ^13^C­{^1^H} NMR (100 MHz, CD_3_OD): δ 207.1, 172.6, 168.6, 167.9, 165.5 (CO), 138.3–126.8 (Ar), 100.6 (C-1), 96.8 (C-1′), 79.6 (C-3), 75.1 (C-4), 74.5, 74.4 (C-5, C-5′), 73.23, 73.17 (C-2, CH_2_(Bn)), 72.8 (C-3′), 72.6 (CH_2_(Bn)), 68.3 (C-4′), 68.0 (C-6), 65.9 (C-6′), 61.1 (CH_2_CH_2_C_8_F_17_), 55.4 (C-2′), 37.0 (CH_2_(Lev)), 30.9 (CH_2_CH_2_C_8_F_17_), 27.9 (CH_3_(Lev)), 27.5 (CH_2_(Lev)); ^19^F NMR (376 MHz, CD_3_OD): δ −82.4 (t, 3F), −114.2 (m, 2F), −123.0 (m, 6F), −123.7 (m, 2F), −124.6 (m, 2F), −127.3 (m, 2F); HR MS; *m*/*z*: calcd for C_56_H_49_F_17_O_18_NS: 1378.2404; found, 1378.2425 [*M*-Na]^−^.

#### 1H,1H,2H,2H-Perfluorodecyl *O*-(2-Benzamide-2-deoxy-6-*O*-sulfo-β-d-glucopyranosyl)-(1 → 4)-3,6-Di-*O*-benzyl-β-d-glucopyranoside (**10**)

Ethylene diamine (193 μL, 2.89 mmol) was added, under an argon atmosphere, to a solution of **30** (27 mg, 19.3 μmol) in a mixture of *n*-BuOH (1.4 mL), MeOH (0.6 mL), and 2,2,2-trifluoroethanol (0.15 mL). Then, the reaction mixture was subjected to microwave irradiation (40 W average power) for 90 min at 120 °C (3 cycles, 30 min each). The reaction vessel was cooled, and the mixture was concentrated to dryness. The residue was dissolved in MeOH/CF_3_CH_2_OH (4 mL/0.2 mL), and an aqueous solution of NaOH (4 M, 434 μL) was added. After being stirred for 24 h at room temperature, the reaction mixture was neutralized with Amberlite IR-120 (H^+^) resin, filtered, and concentrated to give the amine disaccharide intermediate. Triethylamine (18 μL, 0.13 mmol) and benzoic anhydride (44 mg, 0.19 mmol) were added to a cooled (0 °C) solution containing a part of this amine derivative (9.7 μmol) in MeOH (1.5 mL). After stirring for 3.5 h at room temperature, Et_3_N (0.25 mL) was added and the mixture was concentrated to dryness. The residue was purified by silica gel column chromatography (EtOAc-MeOH-H_2_O 72:5:3 → 64:5:3 → 56:5:3) and eluted from a Dowex 50WX2-Na^+^ column (MeOH as eluent) to obtain **10** as a sodium salt (8 mg, 73%, white amorphous solid). TLC (EtOAc-MeOH-H_2_O 36:5:3) Rf 0.38; ^1^H NMR (400 MHz, CD_3_OD): δ 7.89–7.22 (m, 15H, Ar), 5.07–4.95 (2d, 2H, *J* = 11.5 Hz, CH_2_(Bn)), 4.92 (d, 1H, *J*_1,2_ = 8.1 Hz, H-1′), 4.44 (d, 1H, *J* = 11.8 Hz, CH_2_(Bn)), 4.34 (m, 2H, H-1, CH_2_(Bn)), 4.23 (dd, 1H, H-6′), 4.13–4.07 (m, 2H, CH_2_CH_2_C_8_F_17,_ H-6′), 3.92–3.83 (m, 3H, H-2′, CH_2_CH_2_C_8_F_17,_ H-4), 3.77 (dd, 1H, H-6), 3.68–3.60 (m, 3H, H-6, H-3, H-3′), 3.57–3.47 (m, 2H, H-4′, H-5), 3.40 (t, 1H, *J*_1,2_ = *J*_2,3_ = 8.4 Hz, H-2), 3.28 (m, 1H, H-5′), 2.56 (m, 2H, CH_2_CH_2_C_8_F_17_); ^13^C­{^1^H} NMR (100 MHz, CD_3_OD): δ 169.2 (CO), 139.3–126.7 (Ar), 103.0 (C-1), 100.5 (C-1′), 82.9 (C-3), 76.3 (C-4), 74.7, 74.5 (C-5, C-5′), 73.8, 73.65, 73.60 (C-2, C-3′, CH_2_(Bn)), 72.7 (CH_2_(Bn)), 70.4 (C-4′), 69.1 (C-6), 66.6 (C-6′), 61.2 (CH_2_CH_2_C_8_F_17_), 57.4 (C-2′), 31.2 (CH_2_CH_2_C_8_F_17_); ^19^F NMR (376 MHz, CD_3_OD): δ −82.4 (t, 3F), −114.4 (m, 2F), −122.7 (m, 6F), −123.7 (m, 2F), −124.6 (m, 2F), −127.3 (m, 2F); HR MS; *m*/*z*: calcd for C_43_H_41_F_17_O_14_NS: 1150.1982; found, 1150.1991 [*M*-Na]^−^.

#### 1H,1H,2H,2H-Perfluorodecyl *O*-(2-Acetamide-2-deoxy-6-*O*-sulfo-β-d-glucopyranosyl)-(1 → 4)-3,6-Di-*O*-benzyl-β-d-glucopyranoside (**11**)

A solution of the previous free amine disaccharide (9.7 μmol, obtained from **30**) in MeOH (1.5 mL) was treated at 0 °C with triethylamine (18 μL, 0.13 mmol) and acetic anhydride (18 μL, 0.19 mmol). After stirring for 3.5 h at room temperature, Et_3_N (0.25 mL) was added and the mixture was concentrated to dryness. The residue was then purified by silica gel column chromatography (EtOAc-MeOH-H_2_O 72:5:3 → 56:5:3 → 48:5:3) and finally eluted from a Dowex 50WX2-Na^+^ column (MeOH) to obtain **11** as a sodium salt (6 mg, 55%, white amorphous solid). TLC (EtOAc-MeOH-H_2_O 36:5:3) Rf 0.25; ^1^H NMR (400 MHz, CD_3_OD): δ 7.52–7.21 (m, 10H, Ar), 5.04–4.92 (2d, 2H, *J* = 11.6 Hz, CH_2_(Bn)), 4.73 (d, 1H, *J*_1,2_ = 8.4 Hz, H-1′), 4.69–4.59 (2d, 2H, *J* = 11.8 Hz, CH_2_(Bn)), 4.36 (d, 1H, *J*_1,2_ = 7.6 Hz, H-1), 4.21–4.06 (m, 3H, CH_2_CH_2_C_8_F_17,_ 2x H-6′), 3.95–3.86 (m, 2H, CH_2_CH_2_C_8_F_17,_ H-4), 3.83 (dd, 1H, *J*_5,6_ = 2.0 Hz, *J*_6,6_ = 11.2 Hz, H-6), 3.76 (dd, 1H, *J*_5,6_ = 5.2 Hz, H-6), 3.64–3.55 (m, 3H, H-2′, H-3, H-5), 3.44–3.41 (m, 3H, H-2, H-3′, H-4′), 3.19 (m, 1H, H-5′), 2.59 (m, 2H, CH_2_CH_2_C_8_F_17_), 1.94 (s, 3H, NHAc); ^13^C­{^1^H} NMR (100 MHz, CD_3_OD): δ 172.3 (CO), 139.3–126.7 (Ar), 103.1 (C-1), 100.2 (C-1′), 82.8 (C-3), 75.8 (C-4), 74.7, 74.6 (C-5, C-5′), 73.9, 73.73, 73.66 (C-2, C-3′, CH_2_(Bn)), 73.0 (CH_2_(Bn)), 70.2 (C-4′), 68.8 (C-6), 66.5 (C-6′), 61.3 (CH_2_CH_2_C_8_F_17_), 56.8 (C-2′), 31.3 (CH_2_CH_2_C_8_F_17_), 21.8 (NHAc); ^19^F NMR (376 MHz, CD_3_OD): δ −82.4 (t, 3F), −114.4 (m, 2F), −122.9 (m, 6F), −123.7 (m, 2F), −124.6 (m, 2F), −127.3 (m, 2F); HR MS; *m*/*z*: calcd for C_38_H_39_F_17_O_14_NS: 1088.1825; found, 1088.1836 [*M*-Na]^−^.

#### 1H,1H,2H,2H-Perfluorodecyl *O*-(2-Deoxy-2-phthalimido-3,4,6-tri-*O*-sulfo-β-d-glucopyranosyl)-(1 → 4)-2-*O*-Benzoyl-3,6-di-*O*-benzyl-β-d-glucopyranoside (**31**)

An excess of (HF)_*n*_·Py (0.089 mL, 3.43 mmol) was added at 0 °C under an argon atmosphere to a solution of **23** (23 mg, 17.1 μmol) in dry THF (1.5 mL). After 20 h at 4 °C, the mixture was diluted with CH_2_Cl_2_ and washed with H_2_O, saturated NaHCO_3_ solution, and H_2_O. The organic layers were dried (MgSO_4_), filtered, and concentrated in vacuo to give the corresponding triol (21 mg) that was directly submitted to the sulfation reaction. TLC (toluene/acetone 2:1) Rf 0.30.

The triol (21 mg, 17.1 μmol) and sulfur trioxide–trimethylamine complex (73 mg, 0.53 mmol) were dissolved in dry DMF (1.5 mL) and heated at 100 °C for 2 h using microwave radiation (15 W average power). The reaction vessel was cooled, and Et_3_N (250 μL) was added. The crude solution was purified by Sephadex LH 20 chromatography (CH_2_Cl_2_-MeOH 1:1) and silica gel column chromatography (EtOAc-MeOH-H_2_O 36:5:3 → 24:5:3). Finally, the residue was eluted from a Dowex 50WX2-Na^+^ column (MeOH as eluent) to obtain **31** as a sodium salt (16 mg, 62%, 2 steps, white amorphous solid). TLC (EtOAc-MeOH-H_2_O 24:5:3) Rf 0.33; ^1^H NMR (400 MHz, CD_3_OD): δ 7.99–7.09 (m, 19H, Ar), 5.67 (d, 1H, *J*_1,2_ = 8.4 Hz, H-1′), 5.23 (t, 1H, *J*_2,3_ = *J*_3,4_ = 9.4 Hz, H-3′), 5.11 (t, 1H, *J*_1,2_ = *J*_2,3_ = 8.4 Hz, H-2), 4.93 (d, 1H, *J* = 11.2 Hz, CH_2_(Bn)), 4.76–4.66 (m, 3H, H-6′, H-1, CH_2_(Bn)), 4.49 (2d, 2H, *J* = 12.4 Hz, CH_2_(Bn)), 4.28 (m, 2H, H-2′, H-4′), 4.18–4.01 (m, 4H, H-4, H-3, H-6′, CH_2_CH_2_C_8_F_17_), 3.90 (m, 1H, H-5′), 3.77 (m, 2H, H-5, CH_2_CH_2_C_8_F_17_), 3.66 (m, 2H, H-6), 2.36 (m, 2H, CH_2_CH_2_C_8_F_17_); ^13^C­{^1^H} NMR (100 MHz, CD_3_OD): δ 165.6 (CO), 138.3–122.8 (Ar), 100.6 (C-1), 95.9 (C-1′), 78.5 (C-3), 75.3 (C-3′, C-4′), 74.9 (C-4), 74.4 (C-5), 73.31, 73.26 (C-2, C-5′), 72.7, 72.3 (2x CH_2_(Bn)), 68.3 (C-6), 67.2 (C-6′), 61.1 (CH_2_CH_2_C_8_F_17_), 56.0 (C-2′), 30.9 (CH_2_CH_2_C_8_F_17_); ^19^F NMR (376 MHz, CD_3_OD): δ −82.4 (t, 3F), −114.3 (m, 2F), −123.0 (m, 6F), −123.7 (m, 2F), −124.6 (m, 2F), −127.3 (m, 2F); ESI MS; *m*/*z*: calcd for C_51_H_42_F_17_O_22_NS_3_: 719.6; found, 720.3 [*M*-3Na + H]^2–^.

#### 1H,1H,2H,2H-Perfluorodecyl *O*-(2-Benzamide-2-deoxy-3,4,6-tri-*O*-sulfo-β-d-glucopyranosyl)-(1 → 4)-3,6-Di-*O*-benzyl-β-d-glucopyranoside (**12**)

Ethylene diamine (73 μL, 1.09 mmol) was added, under an argon atmosphere, to a solution of **31** (11 mg, 7.3 μmol) in a mixture of *n*-BuOH (1.2 mL), MeOH (0.6 mL), and 2,2,2-trifluoroethanol (0.16 mL). Then, the reaction mixture was subjected to microwave irradiation (30 W average power) for 90 min at 120 °C (3 cycles, 30 min each). The reaction vessel was cooled, and the mixture was concentrated to dryness. The residue was dissolved in MeOH/CF_3_CH_2_OH (2.0 mL/0.1 mL), and an aqueous solution of NaOH (4 M, 182 μL) was added. After stirring for 24 h at room temperature, the reaction mixture was neutralized with Amberlite IR-120 (H^+^) resin, filtered, and concentrated to give the desired amine intermediate. Triethylamine (13 μL, 0.096 mmol) and benzoic anhydride (33 mg, 0.146 mmol) were added to a cooled (0 °C) solution of this amine derivative in MeOH (2 mL). After stirring for 4 h at room temperature, Et_3_N (0.25 mL) was added and the mixture was concentrated to dryness. The residue was purified by silica gel column chromatography (EtOAc-MeOH-H_2_O 32:5:3 → 28:5:3 → 24:5:3) and eluted from a Dowex 50WX2-Na^+^ column (MeOH) to obtain **12** as a sodium salt (8 mg, 80%, white amorphous solid). TLC (EtOAc-MeOH-H_2_O 24:5:3) Rf 0.22; ^1^H NMR (400 MHz, CD_3_OD): δ 7.90–7.20 (m, 15H, Ar), 5.07 (d, 1H, *J* = 11.2 Hz, CH_2_(Bn)), 4.93–4.90 (m, 2H, H-1′, CH_2_(Bn)), 4.71–4–62 (m, 2H, H-6′, H-3′), 4.49 (d, 1H, *J* = 12.0 Hz, CH_2_(Bn)), 4.42–4–37 (m, 2H, H-1, CH_2_(Bn)), 4.30 (t, 1H, *J*_3,4_ = *J*_4,5_ = 8.6 Hz, H-4′), 4.22 (m, 1H, H-2′), 4.13–4.08 (m, 2H, H-6′, CH_2_CH_2_C_8_F_17_), 3.92–3.81 (m, 3H, H-4, H-5′, CH_2_CH_2_C_8_F_17_), 3.74 (m, 2H, H-6, H-3), 3.66 (m, 2H, H-6, H-5), 3.41 (dd, 1H, *J*_1,2_ = 7.8 Hz, *J*_2,3_ = 8.6 Hz, H-2), 2.56 (m, 2H, CH_2_CH_2_C_8_F_17_); ^13^C­{^1^H} NMR (100 MHz, CD_3_OD): δ 169.1 (CO), 139.3–126.7 (Ar), 103.0 (C-1), 99.8 (C-1′), 82.0 (C-3), 77.1 (C-3′), 76.0 (C-4), 74.46, 74.40 (C-5, C-4′), 73.76, 73.71 (C-2, C-5′), 73.2, 72.8 (2x CH_2_(Bn)), 68.9 (C-6), 67.5 (C-6′), 61.2 (CH_2_CH_2_C_8_F_17_), 55.3 (C-2′), 31.2 (CH_2_CH_2_C_8_F_17_); ^19^F NMR (376 MHz, CD_3_OD): δ −82.4 (t, 3F), −114.4 (m, 2F), −122.9 (m, 6F), −123.7 (m, 2F), −124.6 (m, 2F), −127.3 (m, 2F); HR MS; *m*/*z*: calcd for C_43_H_39_F_17_O_20_NS_3_Na_2_: 1354.0757; found, 1354.0745 [*M*-Na]^−^.

#### 1H,1H,2H,2H-Perfluorodecyl *O*-(2-Acetamide-2-deoxy-3,4,6-tri-*O*-sulfo-β-d-glucopyranosyl)-(1 → 4)-3,6-Di-*O*-benzyl-β-d-glucopyranoside (**13**)

Ethylene diamine (106 μL, 1.59 mmol) was added, under an argon atmosphere, to a solution of **31** (16 mg, 10.6 μmol) in a mixture of *n*-BuOH (1.2 mL), MeOH (0.6 mL), and 2,2,2-trifluoroethanol (0.16 mL). Then, the reaction mixture was subjected to microwave irradiation (25 W average power) for 90 min at 120 °C (3 cycles, 30 min each). The reaction vessel was cooled, and the mixture was concentrated to dryness. The residue was dissolved in MeOH/CF_3_CH_2_OH (2.0 mL/0.1 mL), and an aqueous solution of NaOH (4 M, 265 μL) was added. After stirring for 22 h at room temperature, the reaction mixture was neutralized with Amberlite IR-120 (H^+^) resin, filtered, and concentrated to give the desired amine intermediate. Triethylamine (20 μL, 0.14 mmol) and acetic anhydride (20 μL, 0.212 mmol) were added to a cooled (0 °C) solution of this amine derivative in MeOH (1.5 mL). After stirring for 3.5 h at room temperature, Et_3_N (0.25 mL) was added and the mixture was concentrated to dryness. The residue was purified by silica gel column chromatography (EtOAc-MeOH-H_2_O 32:5:3 → 28:5:3 → 24:5:3) and eluted from a Dowex 50WX2-Na^+^ column (MeOH) to obtain **13** as a sodium salt (11 mg, 79%, white amorphous solid). TLC (EtOAc-MeOH-H_2_O 24:5:3) Rf 0.25; ^1^H NMR (400 MHz, CD_3_OD): δ 7.53–7.22 (m, 10H, Ar), 5.06–4.92 (2d, 2H, *J* = 11.2 Hz, CH_2_(Bn)), 4.82 (d, 1H, *J*_1,2_ = 8.0 Hz, H-1′), 4.67–4.60 (m, 3H, 2 x CH_2_(Bn), H-6′), 4.46–4.39 (m, 2H, H-3′, H-1), 4.22–4.12 (m, 2H, H-4′, CH_2_CH_2_C_8_F_17_), 4.05 (dd, 1H, *J*_5,6_ = 8.0 Hz, *J*_6,6_ = 11.4 Hz, H-6′), 3.96–3.87 (m, 3H, CH_2_CH_2_C_8_F_17,_ H-4, H-2′), 3.82 (dd, 1H, *J*_5,6_ = 2.0 Hz, *J*_6,6_ = 11.1 Hz, H-6), 3.78–3.65 (m, 4H, H-6, H-5′, H-3, H-5), 3.43 (t, 1H, *J*_1,2_ = *J*_2,3_ = 8.3 Hz, H-2), 2.59 (m, 2H, CH_2_CH_2_C_8_F_17_), 1.91 (s, 3H, NHAc); ^13^C­{^1^H} NMR (100 MHz, CD_3_OD): δ 172.7 (CO), 139.4–126.8 (Ar), 103.1 (C-1), 99.2 (C-1′), 81.9 (C-3), 77.7 (C-3′), 75.7 (C-4), 74.5, 74.4 (C-5, C-4′), 73.8, 73.6 (C-2, C-5′), 73.1–73.0 (2x CH_2_(Bn)), 68.7 (C-6), 67.4 (C-6′), 61.3 (CH_2_CH_2_C_8_F_17_), 55.1 (C-2′), 31.3 (CH_2_CH_2_C_8_F_17_), 22.0 (NHAc); ^19^F NMR (376 MHz, CD_3_OD): δ −82.4 (t, 3F), −114.4 (m, 2F), −122.9 (m, 6F), −123.7 (m, 2F), −124.6 (m, 2F), −127.3 (m, 2F); HR MS; *m*/*z*: calcd for C_38_H_37_F_17_O_20_NS_3_Na_2_: 1292.0600; found, 1292.0591 [*M*-Na]^−^.

### Fluorescence Polarization Competition Assays

Fluorescence polarization measurements were performed in 384-well microplates (black polystyrene, nontreated, Corning), as previously reported.^49^ The fluorescence polarization was recorded using a TRIAD multimode microplate reader (from Dynex), with excitation and emission wavelengths of 485 and 535 nm, respectively. The fluorescent probe (a fluorescein labeled heparin-like hexasaccharide previously prepared in our lab)^48^ was dissolved in PBS buffer (10 mM, pH 7.4). Recombinant human midkine (Peprotech) was dissolved in a PBS buffer (10 mM, pH 7.4) containing 1% BSA (bovine serum albumin). One or 2 mM stock solutions of compounds **3**–**13** were prepared in PBS/DMSO 9:1 (v/v), and serial dilutions were then performed in PBS buffer (10 mM, pH 7.4).

For the determination of the IC_50_ values, we recorded the fluorescence polarization from wells containing 20 μL of a 125 nM protein solution and 10 μL of a 40 nM probe solution in the presence of 10 μL of inhibitor solution, with concentrations ranging from 1 mM to 0.1 μM. The microplate was shaken in the dark for 10 min before reading. The total sample volume in each well was 40 μL, and the final buffer composition was PBS +0.5% BSA. The final concentrations of the fluorescent probe and protein in each well were 10 and 63 nM, respectively, while the final inhibitor concentration ranged from 250 μM to 25 nM. The average polarization values of at least three replicates were plotted against the logarithm of inhibitor concentration. Two control samples were included in the competition experiment. The first one only contained fluorescent probe and afforded the expected minimum polarization value for 100% inhibition; the second one contained midkine and probe in the absence of inhibitor and gave the maximum polarization value corresponding to 0% inhibition. Blank wells contained 20 μL of protein solution and 20 μL of inhibitor solution, and their measurements were subtracted from all values. The curve was fitted to the equation for a one-site competition: *y* = A_2_ + (A_1_ – A_2_)/[1 + 10̂(*x*-logIC_50_)], where A_1_ and A_2_ are the maximal and minimal values of polarization, respectively, and IC_50_ is the inhibitor concentration that results in 50% inhibition. Three independent experiments were carried out for each IC_50_ calculation.

## Supplementary Material



## Data Availability

The data underlying this study are available in the published article and its online Supporting Information.

## Notes

Fluorescence polarization competition curves and NMR spectra for all compounds (Figures S1 and S2) (PDF)
