# Supplementary material for: Synthesis of Sulfated Glycomimetics with Micromolar Affinity for Midkine
Source: J Org Chem. 2026 Mar 27;91(14):5102–14. doi: 10.1021/acs.joc.6c00064 (PMC13077691; doi:10.1021/acs.joc.6c00064)

Supporting Information

Synthesis of sulfated glycomimetics with micromolar affinity for midkine

Rocío Pereira-Jaramillo,[a] José L. de Paz,*[a] Pedro M. Nieto*[a]

[a] Glycosystems Laboratory, Instituto de Investigaciones Químicas (IIQ), cicCartuja, CSIC and Universidad de Sevilla, Americo Vespucio, 49, 41092 Sevilla, Spain.

* Corresponding author. e-mail: pedro.nieto@iiq.csic.es; jlpaz@iiq.csic.es

Page

Figures S1, S2 S-2

Fluorescence polarization competition assays S-3

NMR spectra S-14

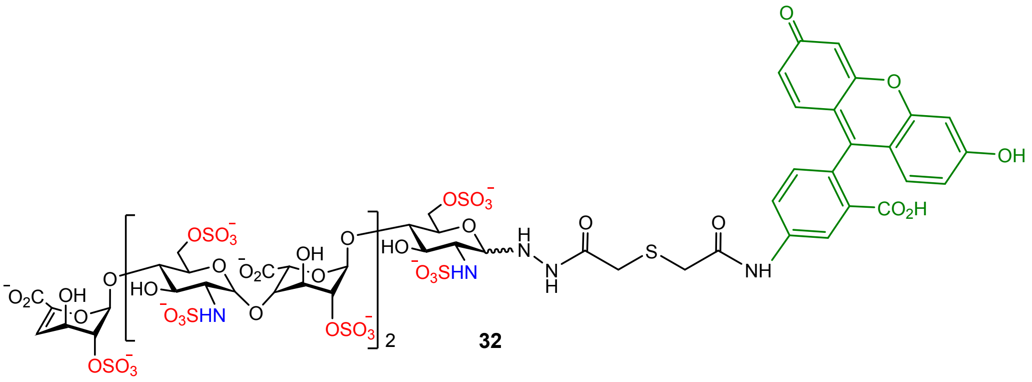

Figure S1. Structure of the fluorescently labelled heparin hexasaccharide **32** employed as probe in the FP competition assay.

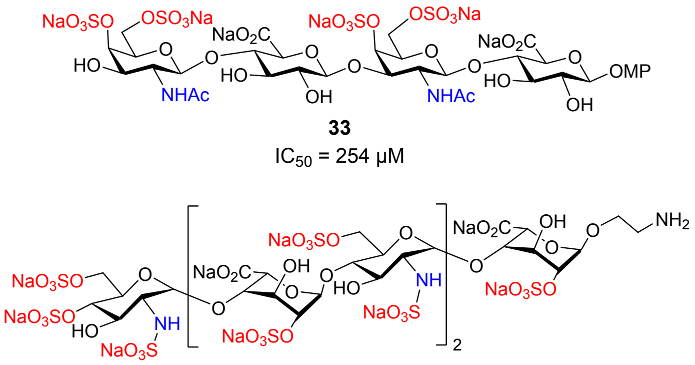

33
IC_{50} = 254 μM

34
IC_{50} = 1.1 μM

Figure S2. Structures of CS-E tetrasaccharide **33** and heparin hexasaccharide **34**. Relative binding affinities for midkine (IC_{50} values obtained with our FP competition assay) are also indicated.

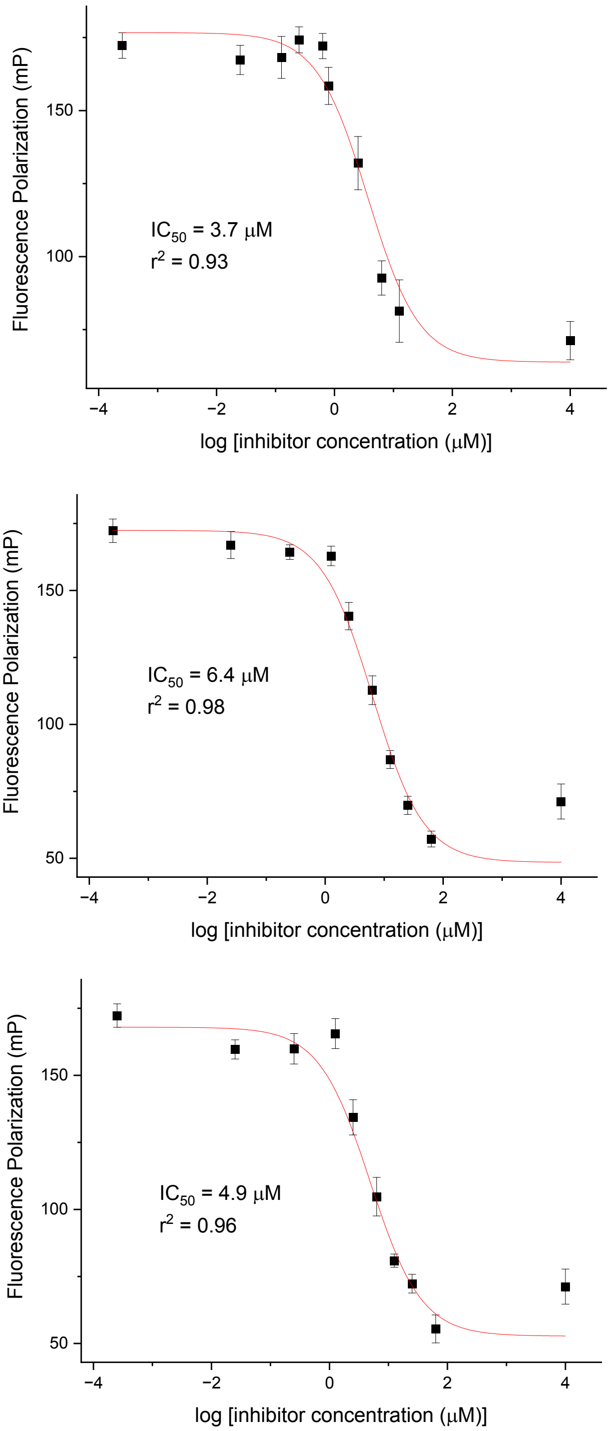

Figure S3. Inhibition curves showing the ability of compound **3** to inhibit the interaction between midkine (63 nM) and fluorescent probe (10 nM). All the FP values are the average of at least three replicate wells, with error bars showing the standard deviations for these measurements. The reported IC_{50} value and the error (5.0 ± 1.4 μM) represent the average and the standard deviation from these three independent experiments.

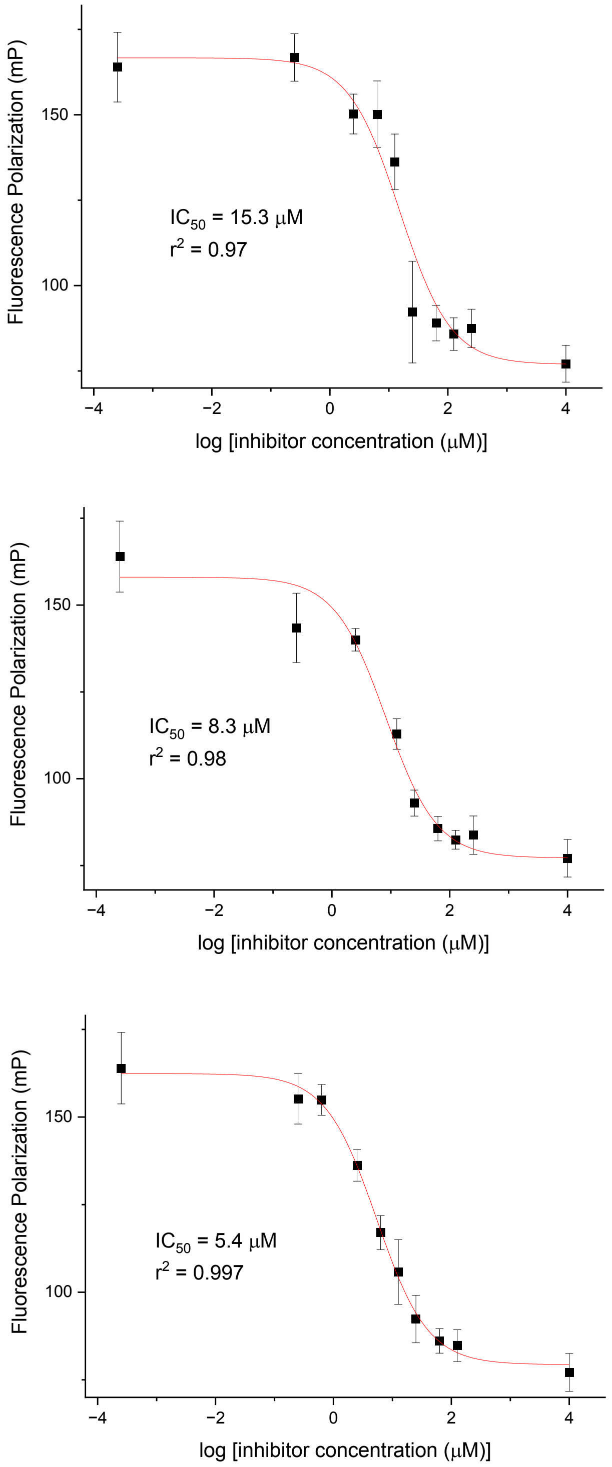

Figure S4. Inhibition curves showing the ability of compound **4** to inhibit the interaction between midkine (63 nM) and fluorescent probe (10 nM). All the FP values are the average of at least three replicate wells, with error bars showing the standard deviations for these measurements. The reported IC_{50} value and the error (9.7 ± 5.1 μM) represent the average and the standard deviation from these three independent experiments.

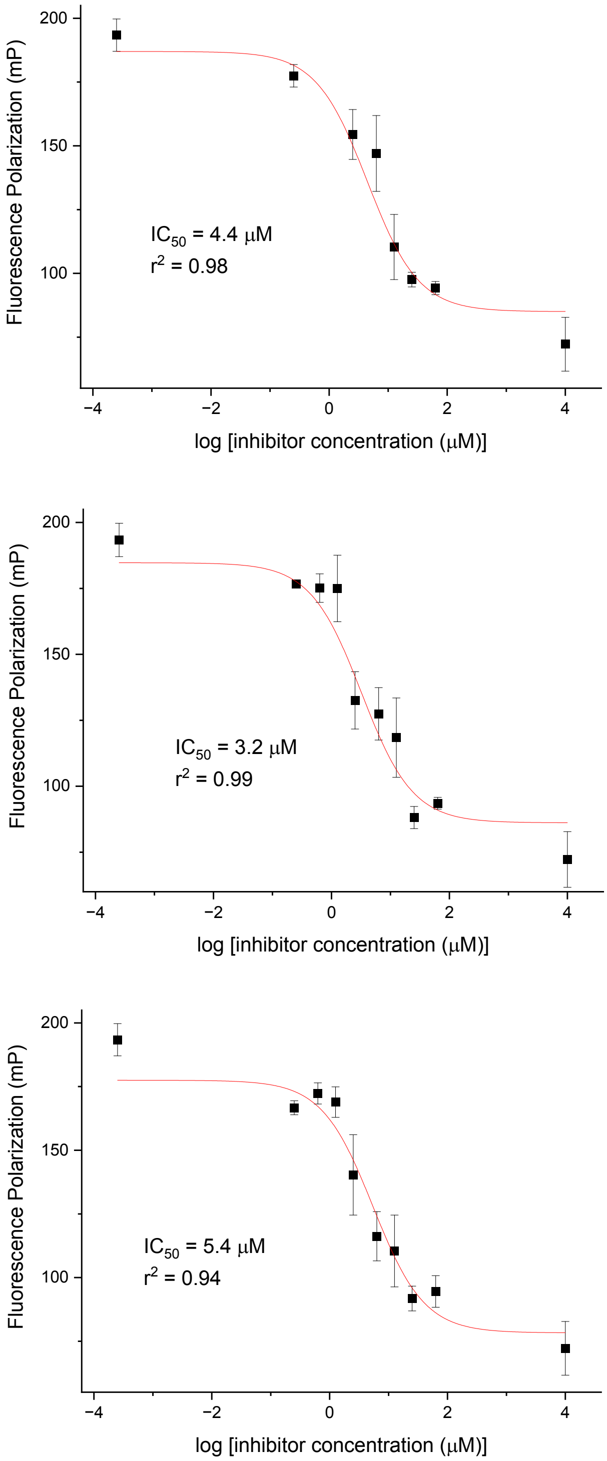

Figure S5. Inhibition curves showing the ability of compound **5** to inhibit the interaction between midkine (63 nM) and fluorescent probe (10 nM). All the FP values are the average of at least three replicate wells, with error bars showing the standard deviations for these measurements. The reported IC_{50} value and the error (4.3 ± 1.1 μM) represent the average and the standard deviation from these three independent experiments.

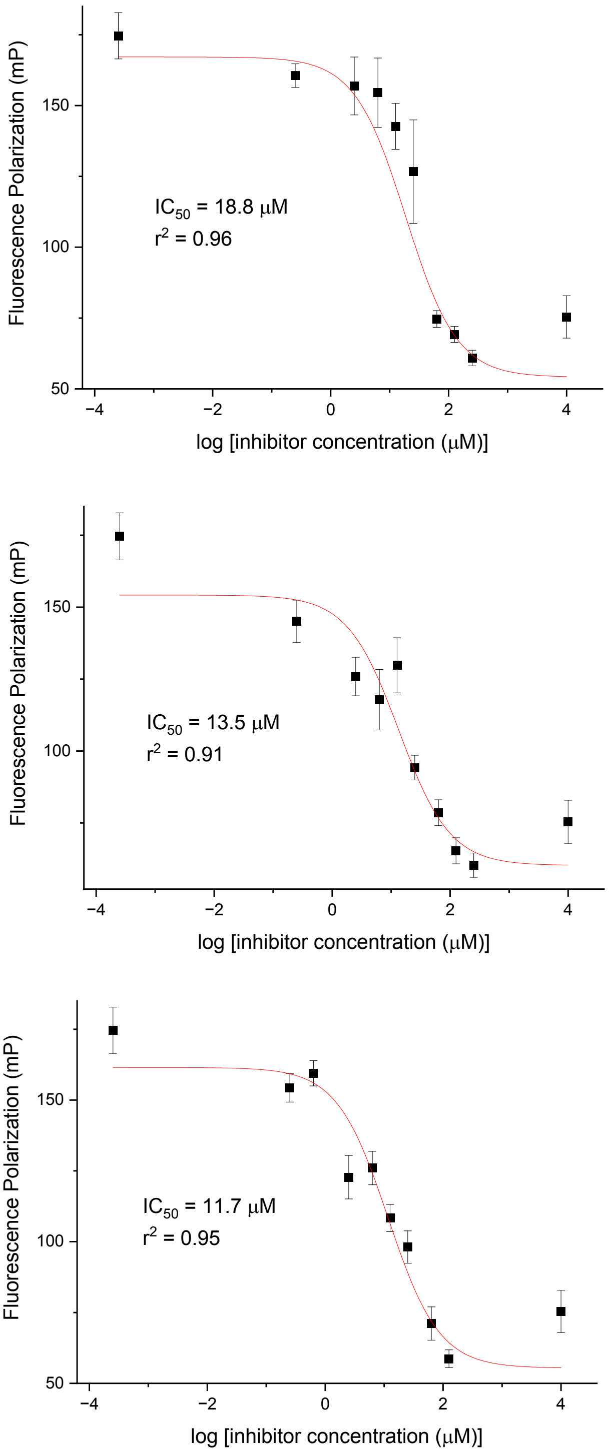

Figure S6. Inhibition curves showing the ability of compound **6** to inhibit the interaction between midkine (63 nM) and fluorescent probe (10 nM). All the FP values are the average of at least three replicate wells, with error bars showing the standard deviations for these measurements. The reported IC_{50} value and the error (14.7 ± 3.7 μM) represent the average and the standard deviation from these three independent experiments.

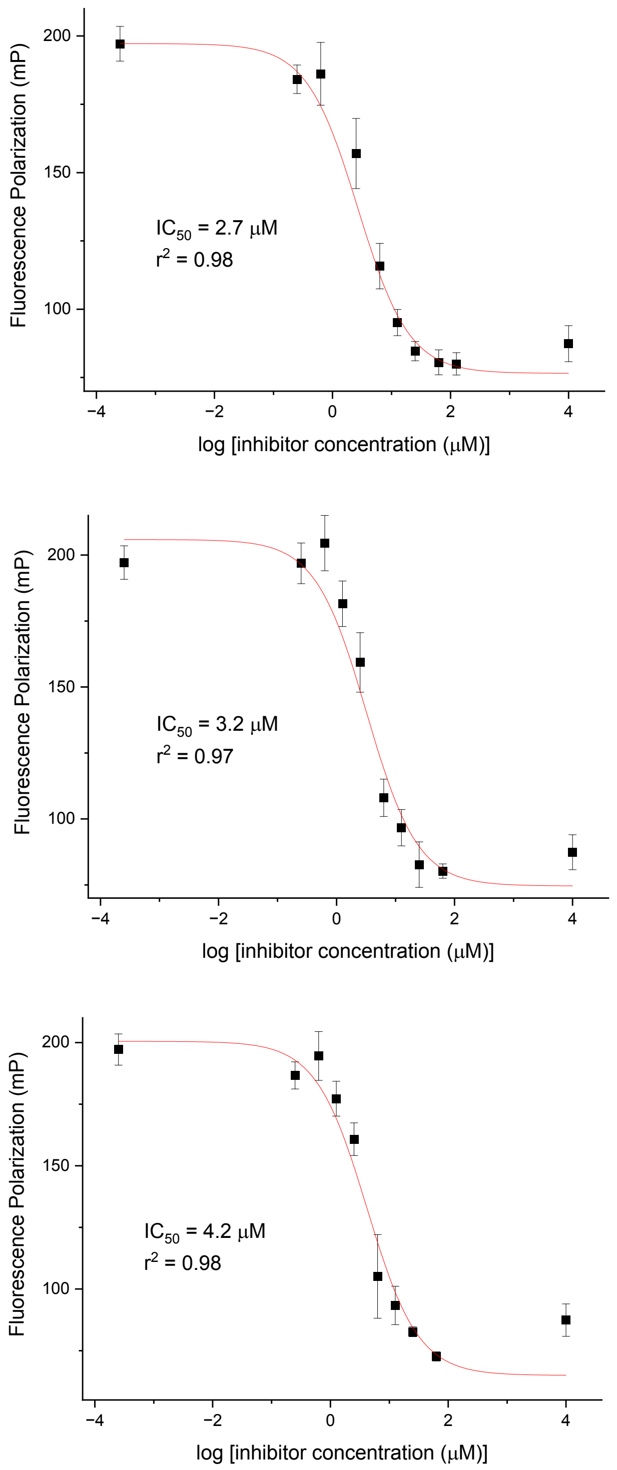

Figure S7. Inhibition curves showing the ability of compound **7** to inhibit the interaction between midkine (63 nM) and fluorescent probe (10 nM). All the FP values are the average of at least three replicate wells, with error bars showing the standard deviations for these measurements. The reported IC_{50} value and the error (3.4 ± 0.8 μM) represent the average and the standard deviation from these three independent experiments.

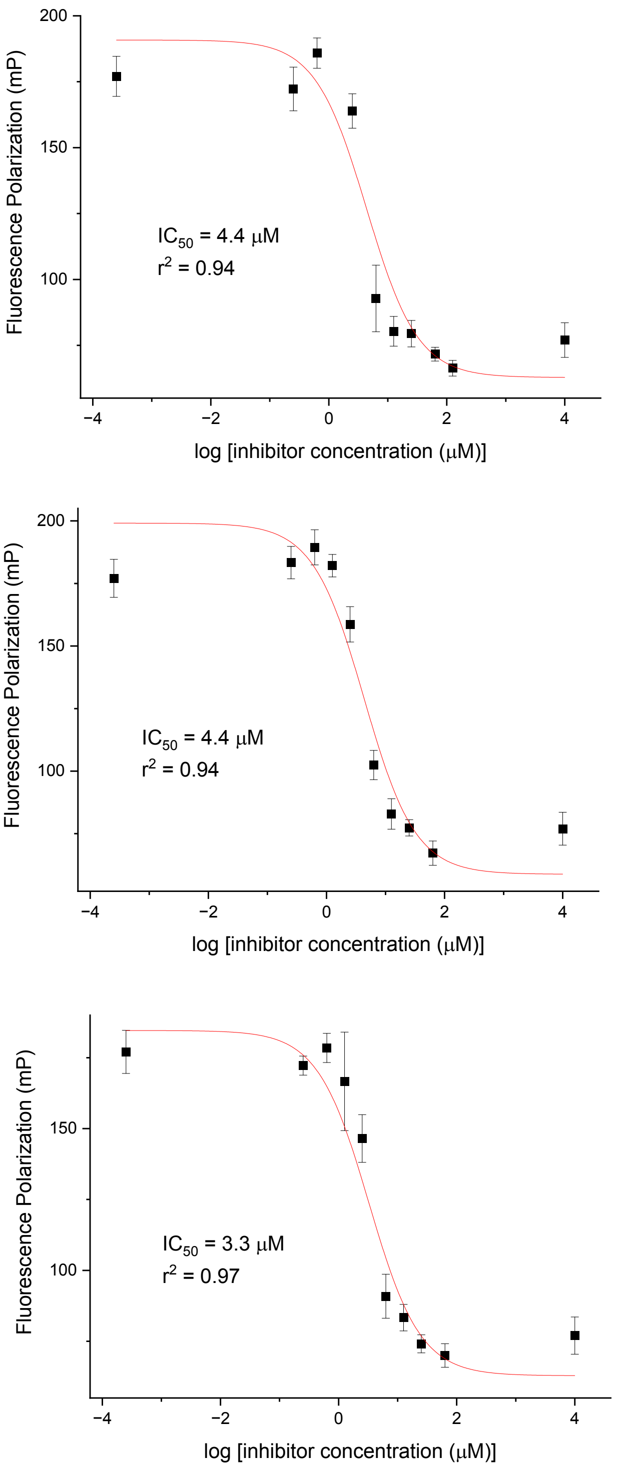

Figure S8. Inhibition curves showing the ability of compound **8** to inhibit the interaction between midkine (63 nM) and fluorescent probe (10 nM). All the FP values are the average of at least three replicate wells, with error bars showing the standard deviations for these measurements. The reported IC_{50} value and the error (4.0 ± 0.6 μM) represent the average and the standard deviation from these three independent experiments.

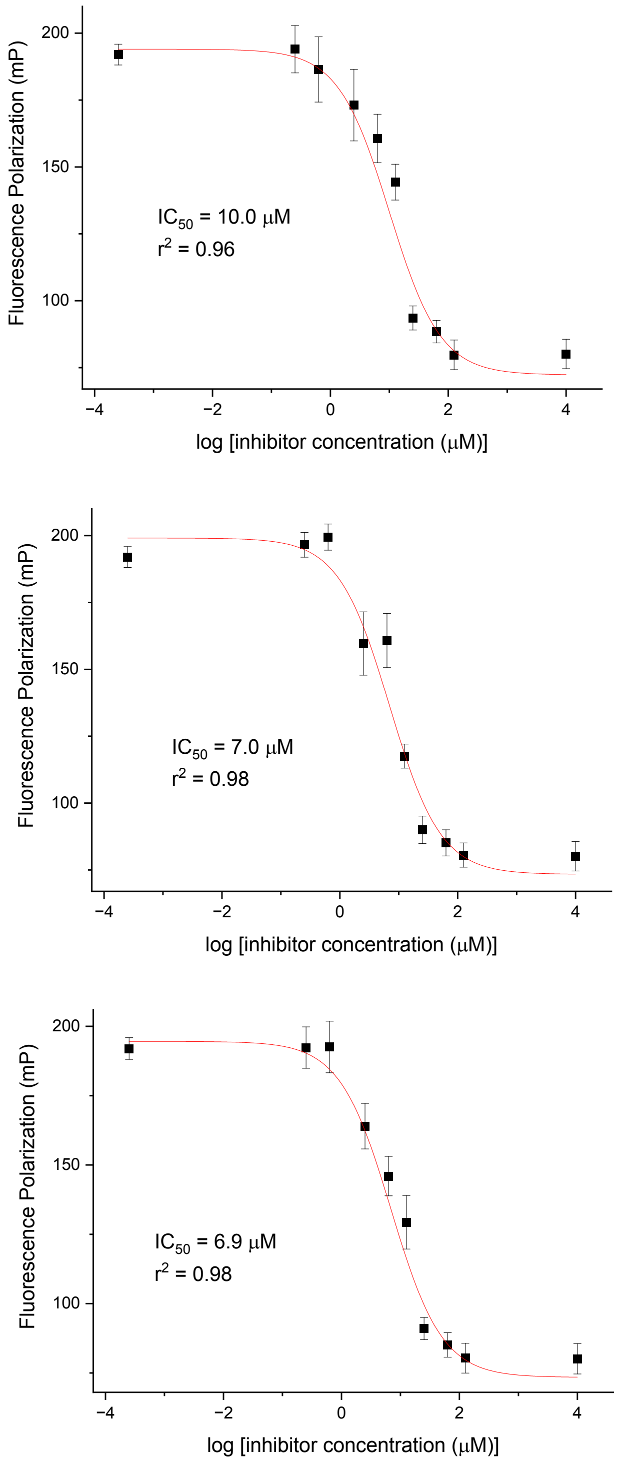

Figure S9. Inhibition curves showing the ability of compound **9** to inhibit the interaction between midkine (63 nM) and fluorescent probe (10 nM). All the FP values are the average of at least three replicate wells, with error bars showing the standard deviations for these measurements. The reported IC_{50} value and the error (8.0 ± 1.8 μM) represent the average and the standard deviation from these three independent experiments.

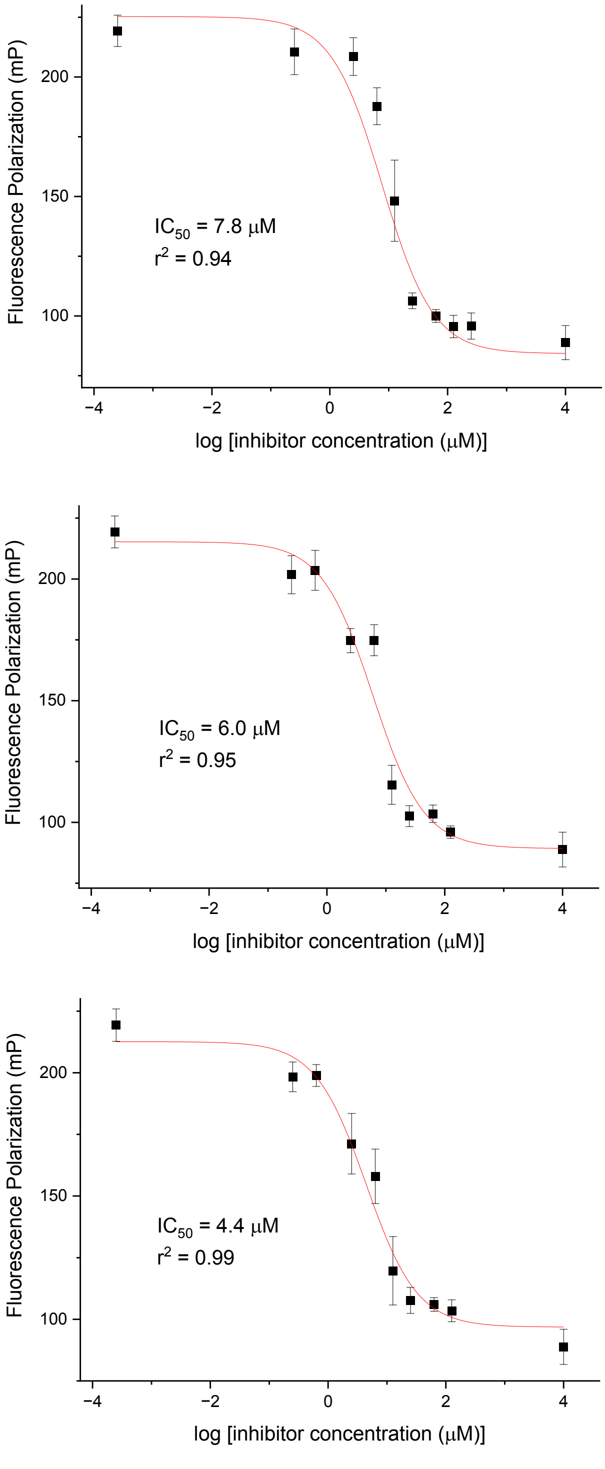

Figure S10. Inhibition curves showing the ability of compound **10** to inhibit the interaction between midkine (63 nM) and fluorescent probe (10 nM). All the FP values are the average of at least three replicate wells, with error bars showing the standard deviations for these measurements. The reported IC_{50} value and the error (6.1 ± 1.7 μM) represent the average and the standard deviation from these three independent experiments.

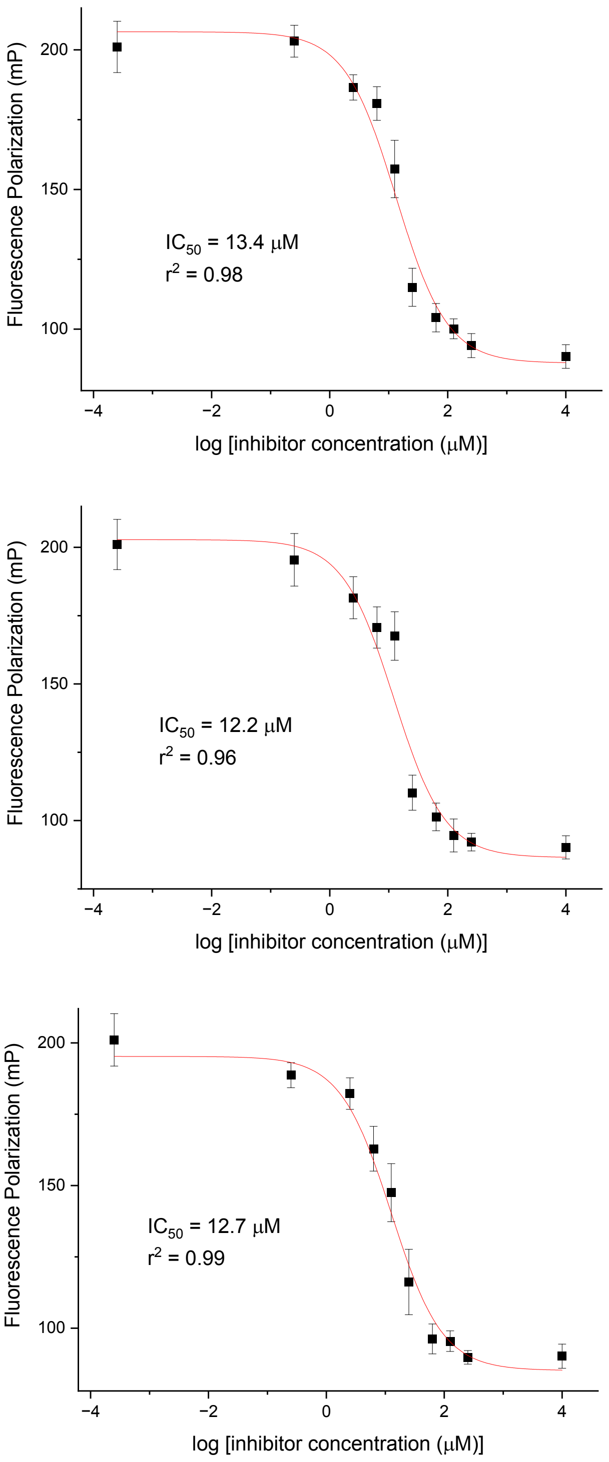

Figure S11. Inhibition curves showing the ability of compound **11** to inhibit the interaction between midkine (63 nM) and fluorescent probe (10 nM). All the FP values are the average of at least three replicate wells, with error bars showing the standard deviations for these measurements. The reported IC_{50} value and the error (12.8 ± 0.6 μM) represent the average and the standard deviation from these three independent experiments.

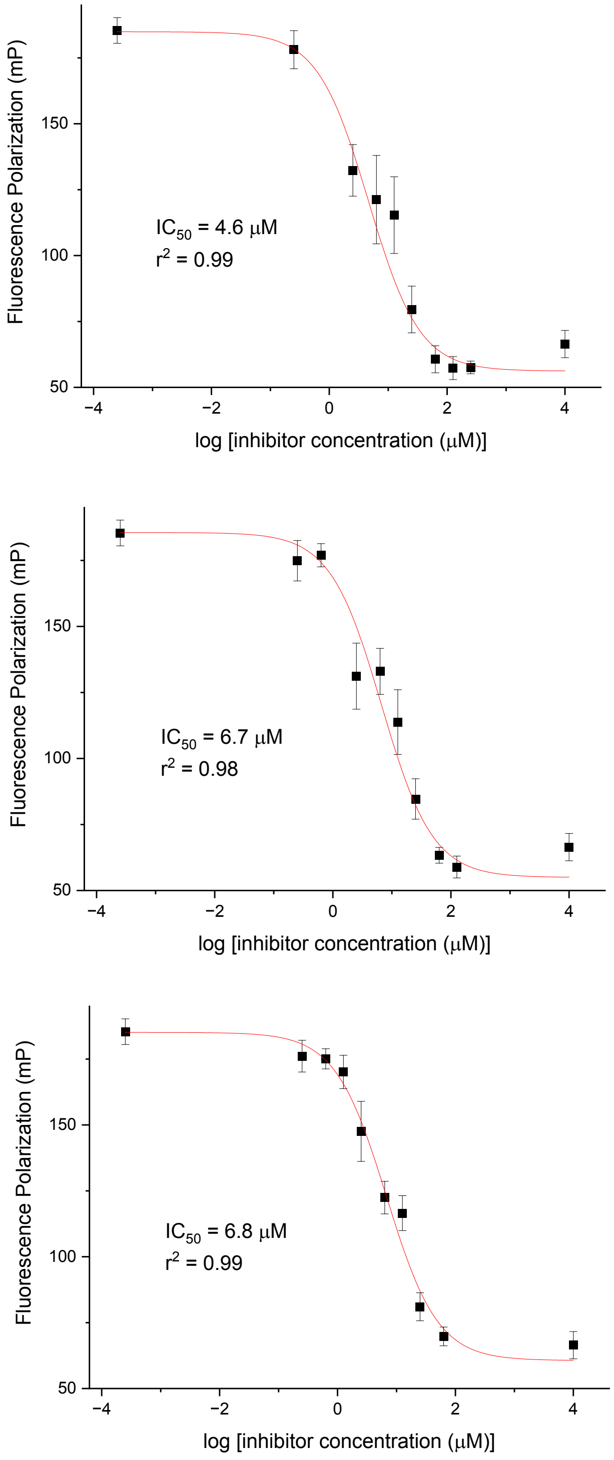

Figure S12. Inhibition curves showing the ability of compound **12** to inhibit the interaction between midkine (63 nM) and fluorescent probe (10 nM). All the FP values are the average of at least three replicate wells, with error bars showing the standard deviations for these measurements. The reported IC_{50} value and the error (6.0 ± 1.2 μM) represent the average and the standard deviation from these three independent experiments.

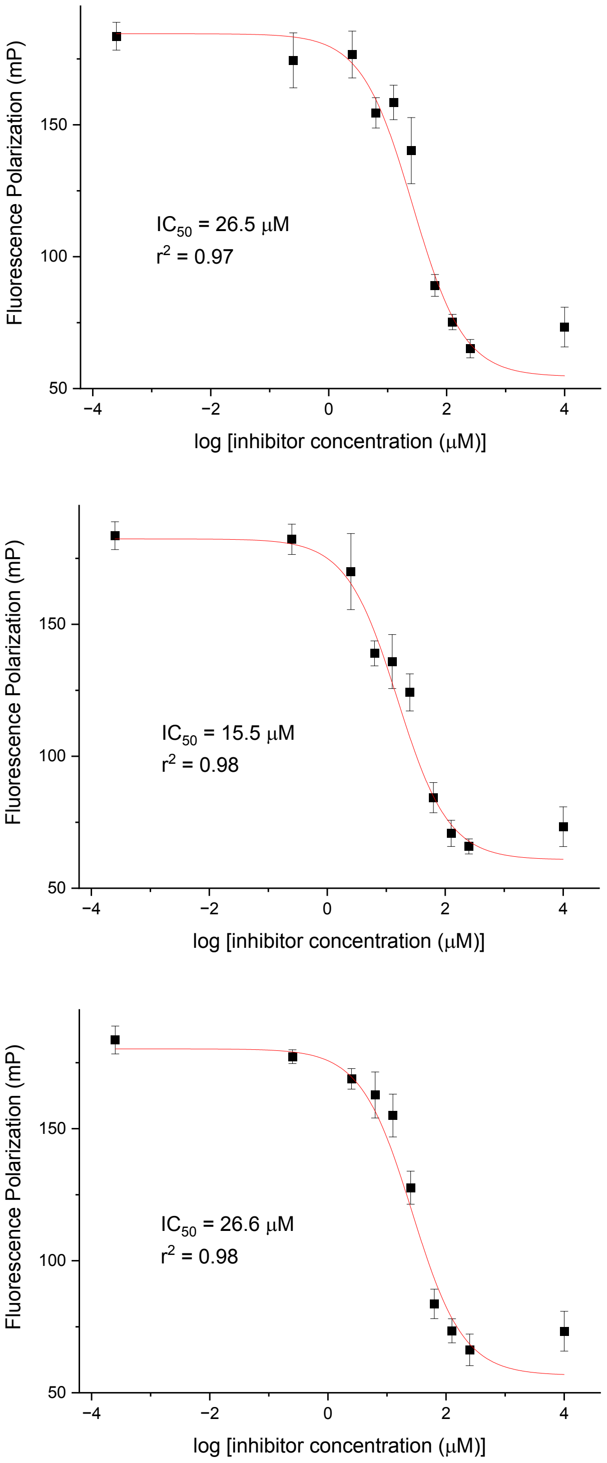

Figure S13. Inhibition curves showing the ability of compound **13** to inhibit the interaction between midkine (63 nM) and fluorescent probe (10 nM). All the FP values are the average of at least three replicate wells, with error bars showing the standard deviations for these measurements. The reported IC_{50} value and the error (22.9 ± 6.4 μM) represent the average and the standard deviation from these three independent experiments.

Compound 15 ^{1}H-NMR (400 MHz, $CDCl_3$)

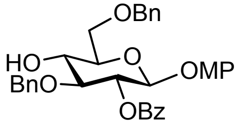

15

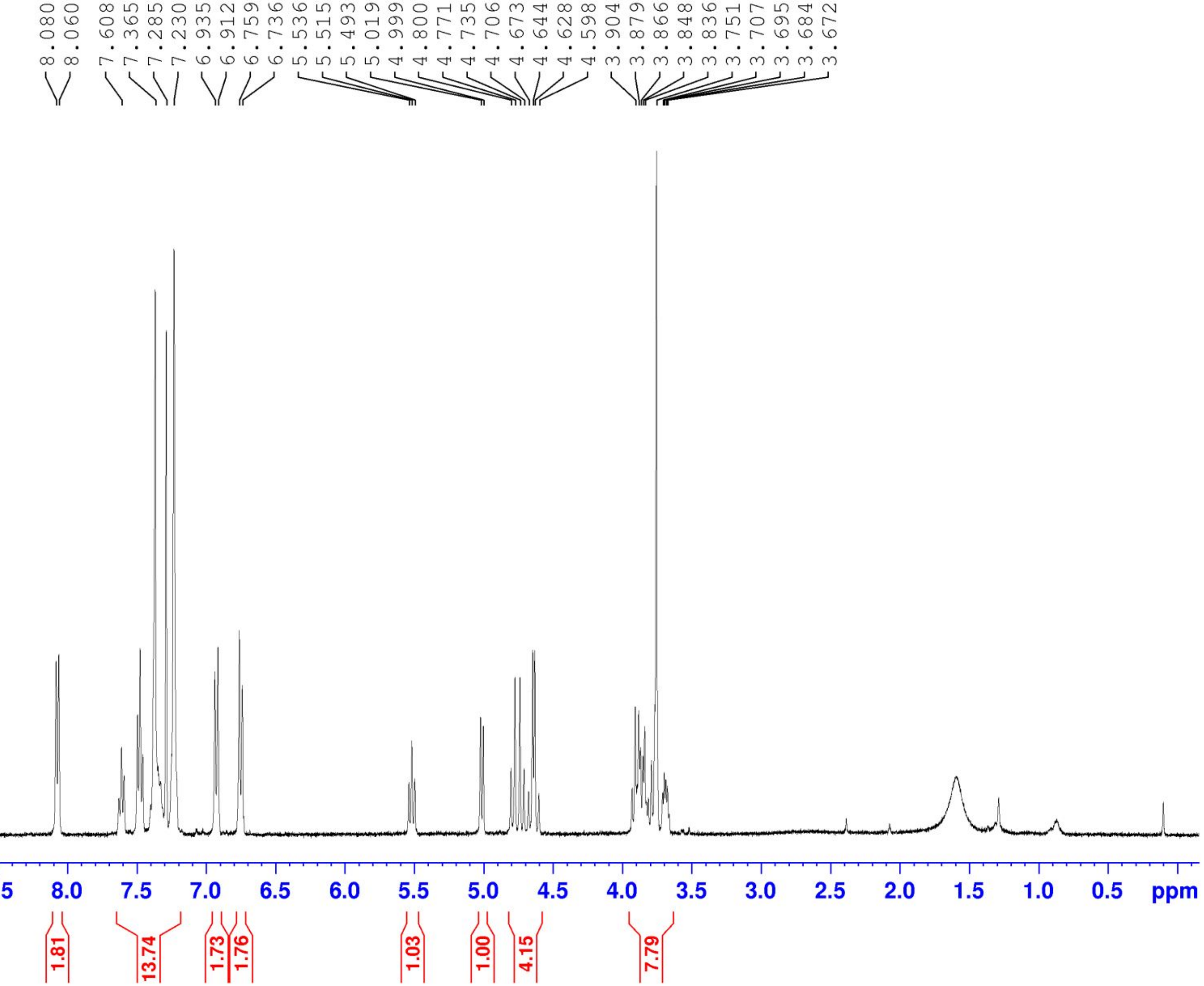

Compound 16 ^{1}H-NMR (400 MHz, $CDCl_3$)

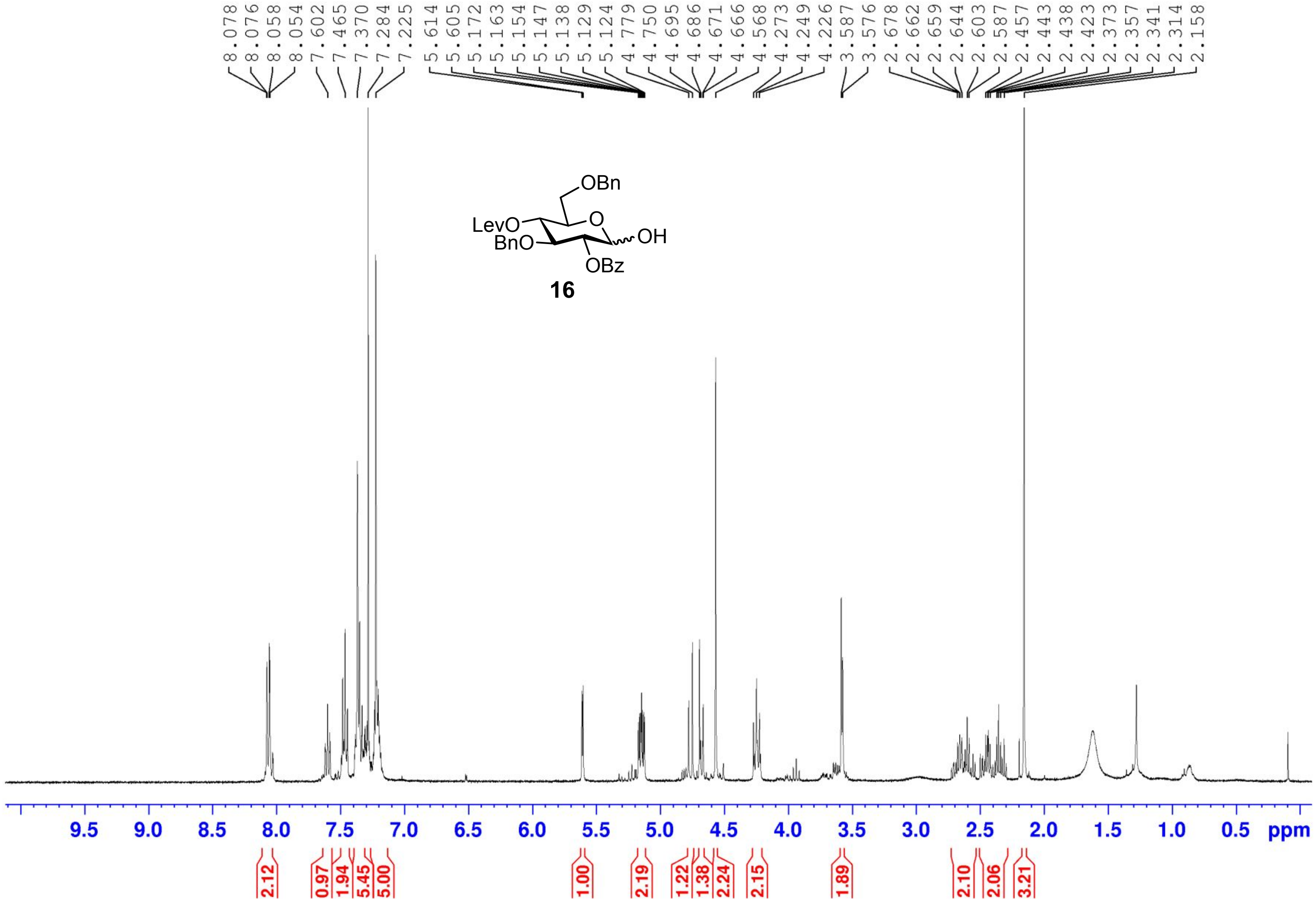

Compound 17 ^{1}H-NMR (400 MHz, $CDCl_3$)

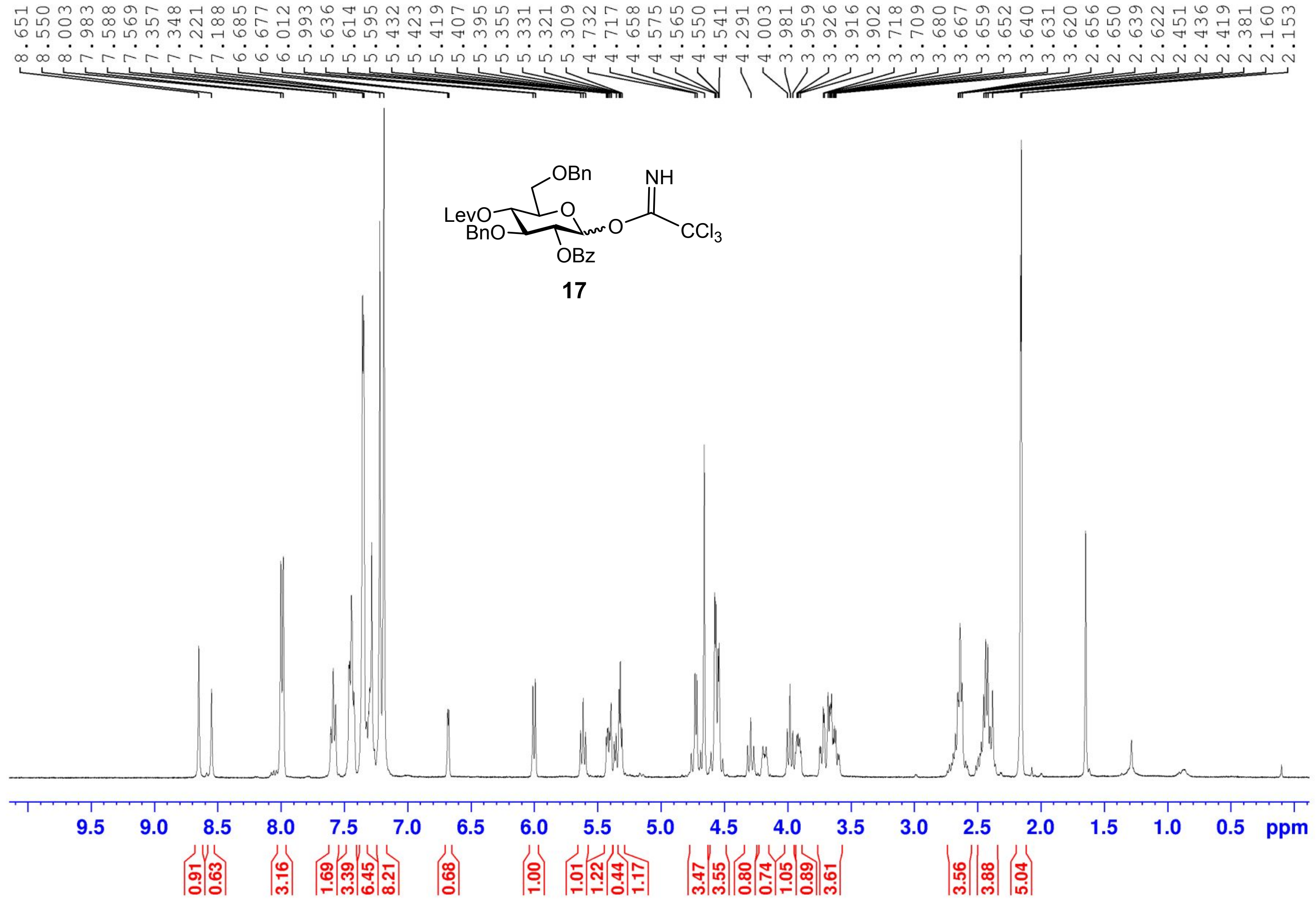

Compound 17 $^{13}C\{^{1}H\}$ NMR (100 MHz, $CDCl_3$)

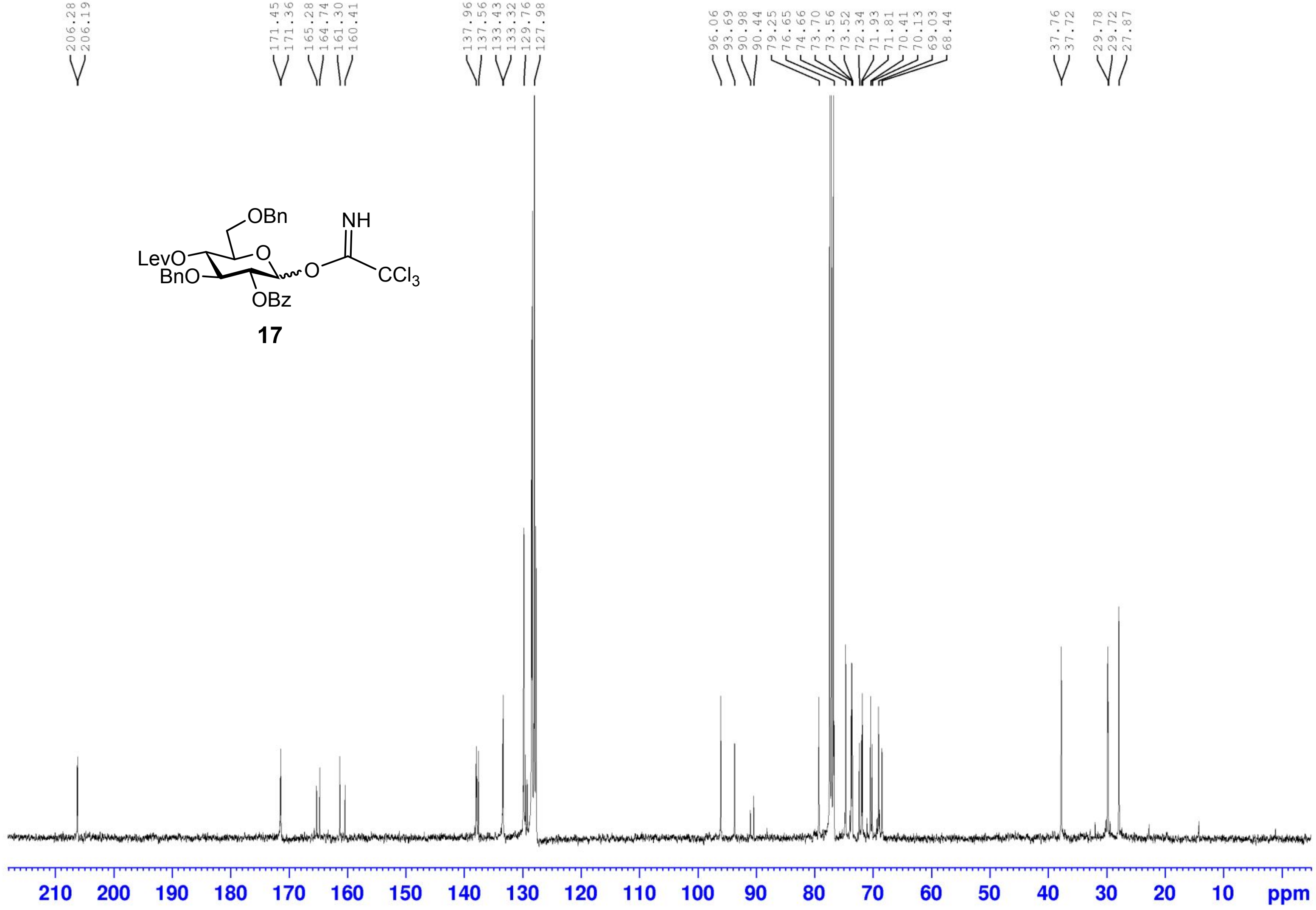

Compound 19 ^{1}H-NMR (400 MHz, $CDCl_3$)

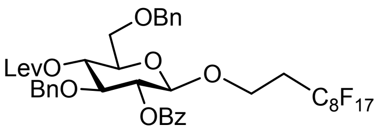

19

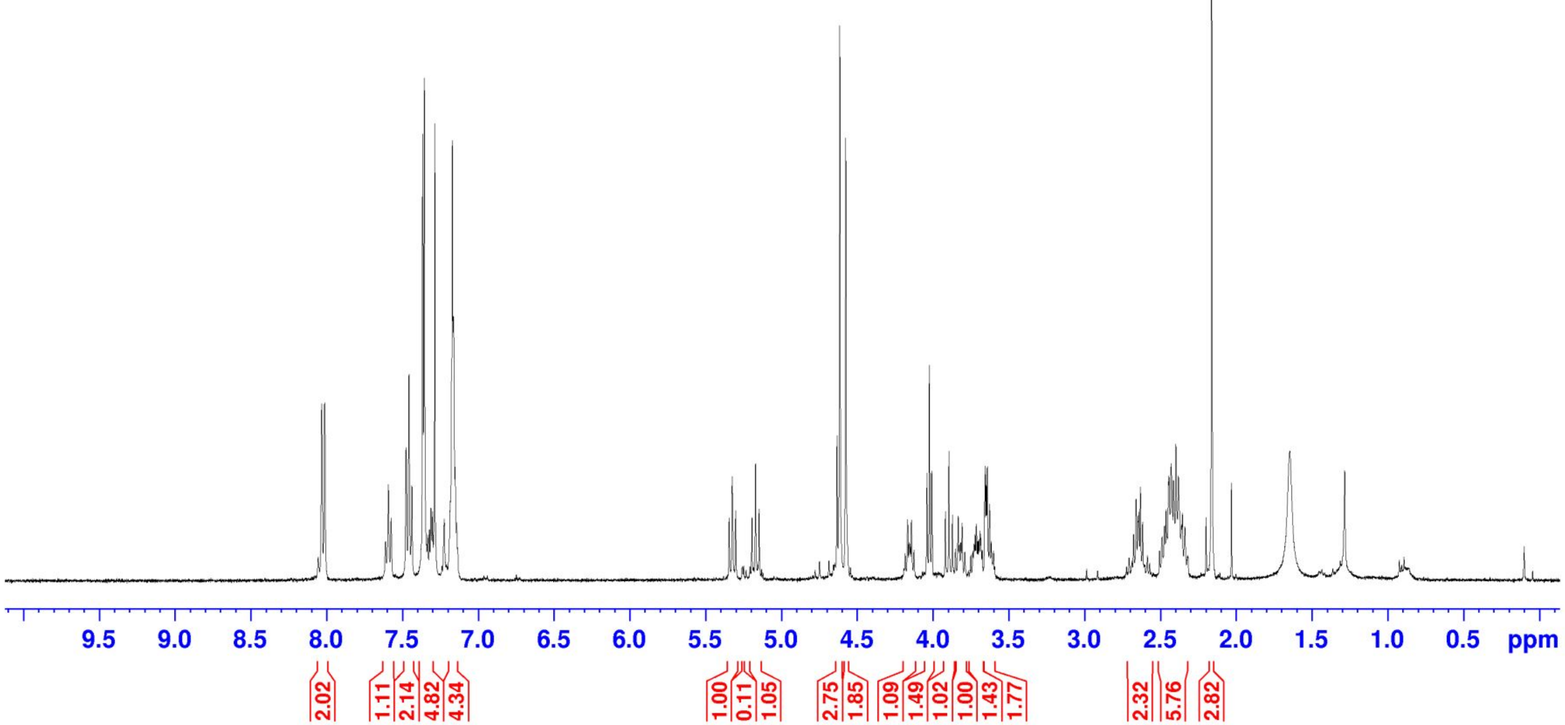

Compound 19 ^{1}H-NMR (400 MHz, $CDCl_3$) (Purified β anomer)

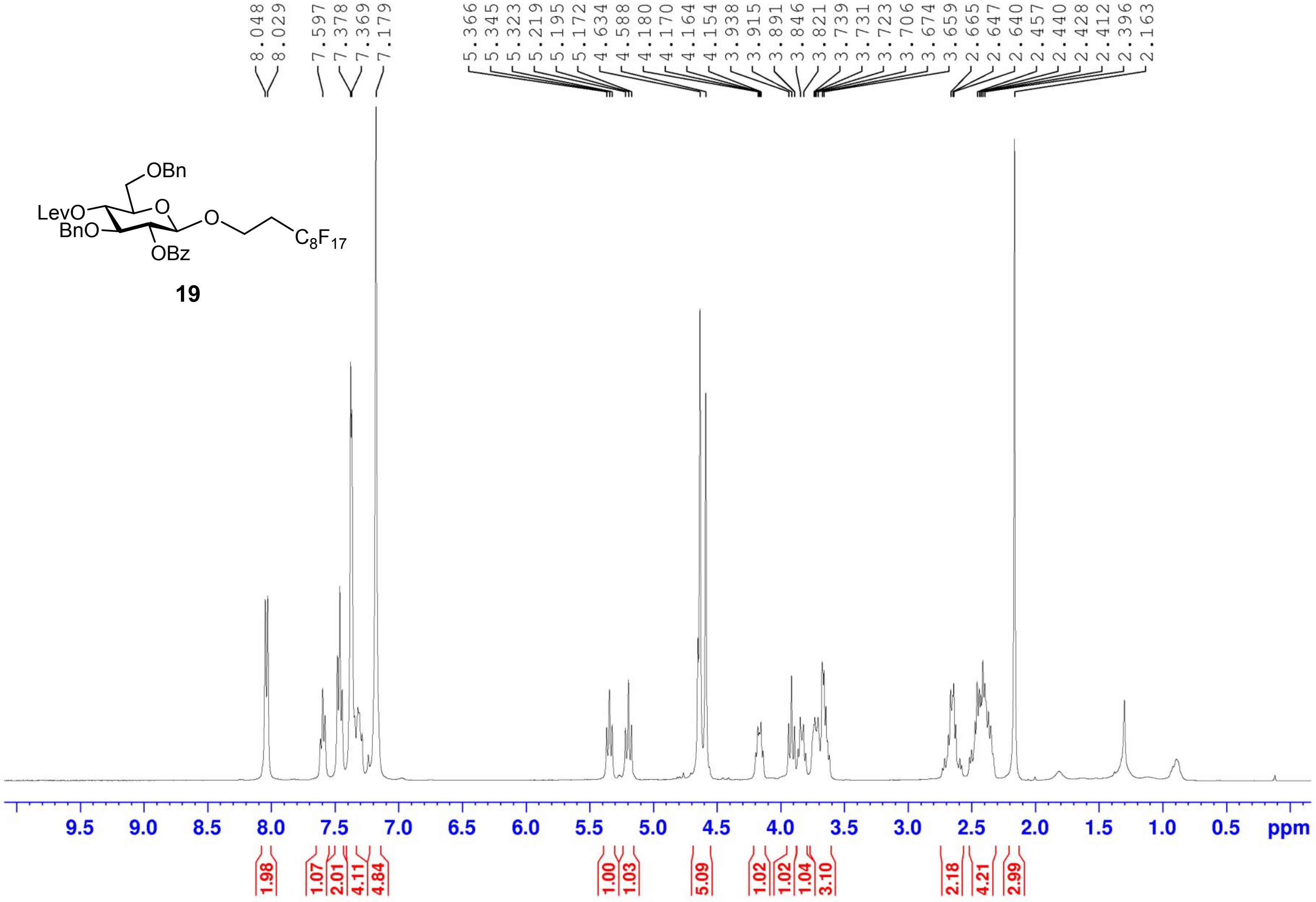

Compound 19 $^{13}C\{^{1}H\}$ NMR (100 MHz, $CDCl_3$)

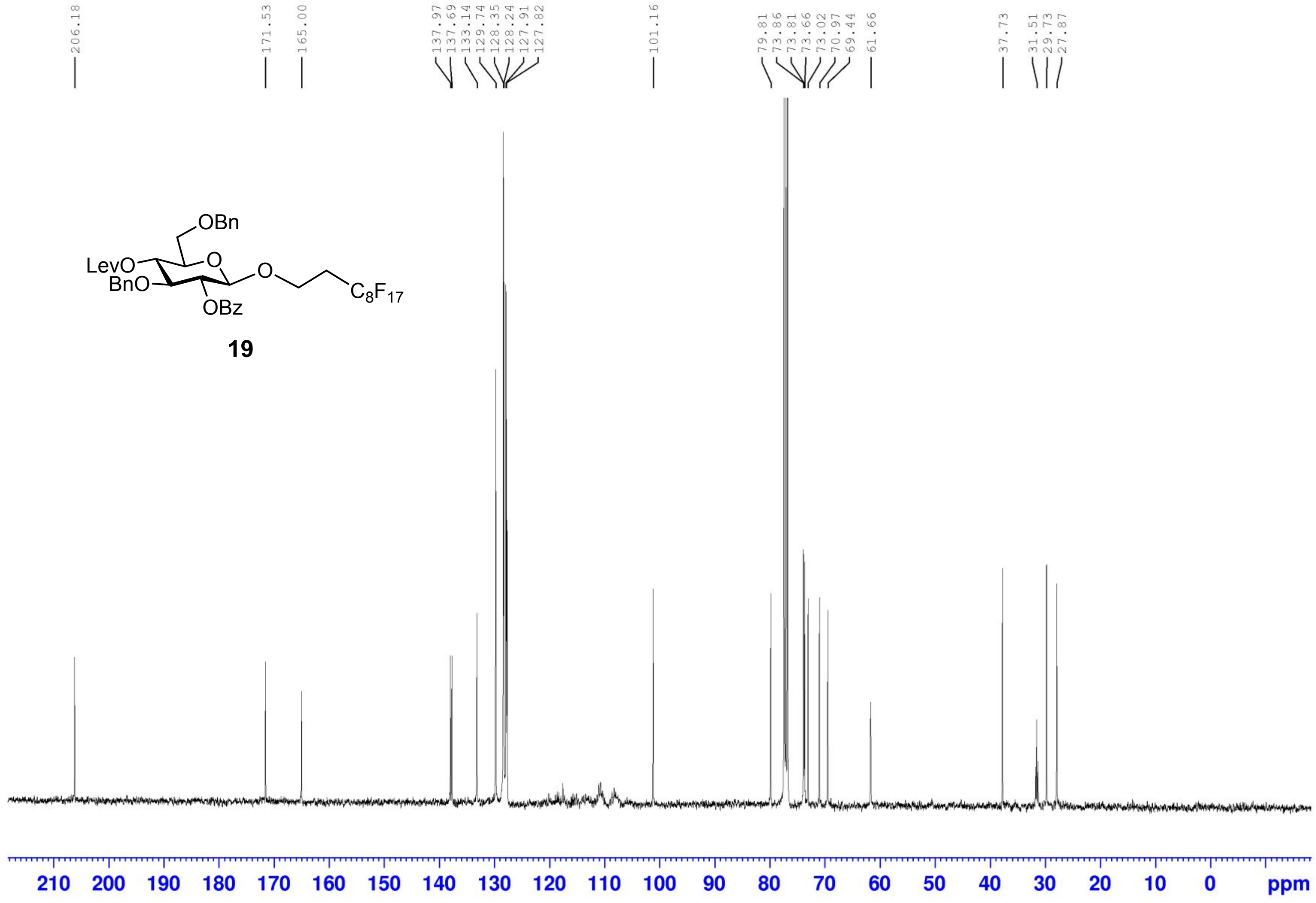

Compound 20 ^{1}H-NMR (400 MHz, $CDCl_3$)

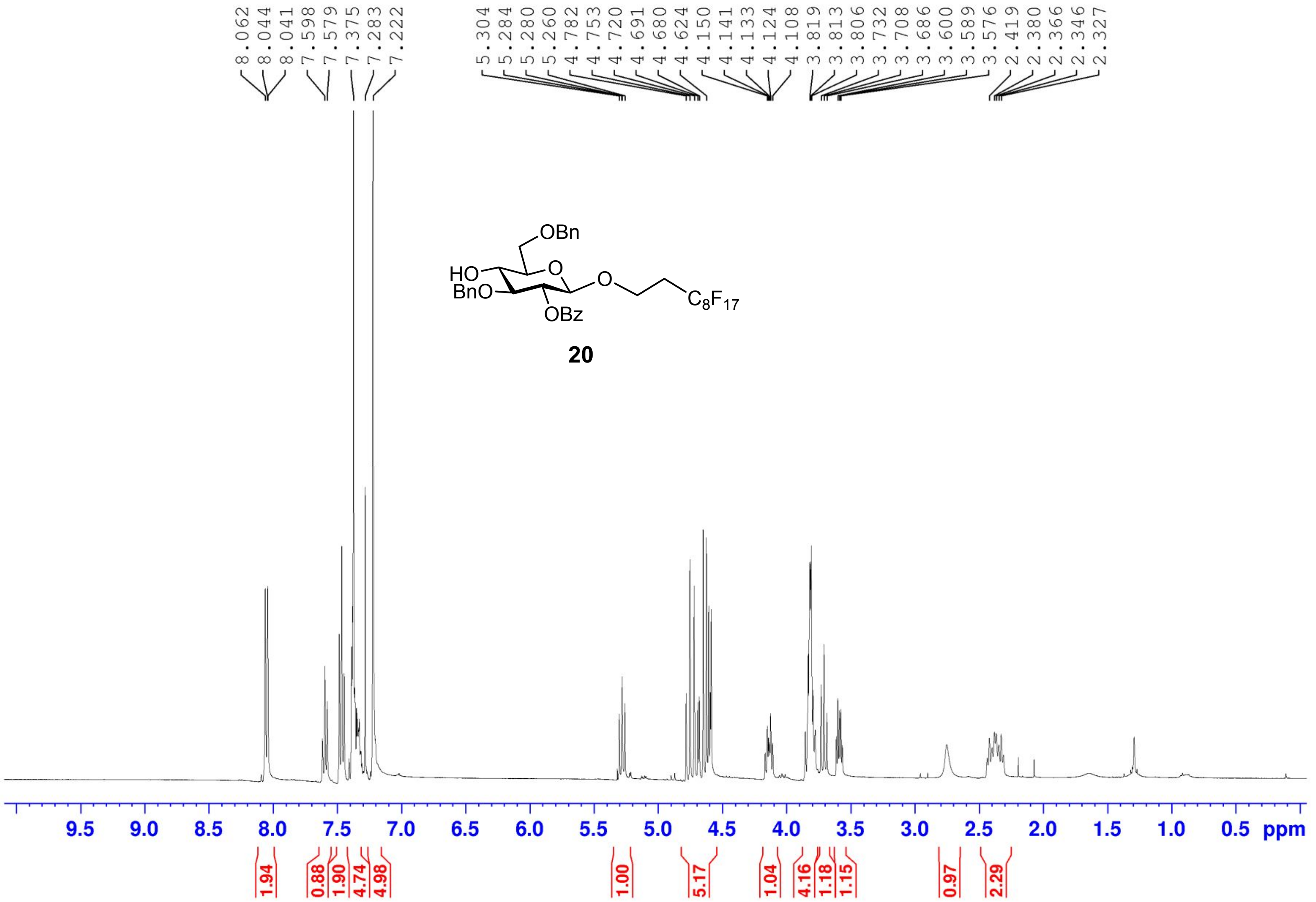

Compound 20 COSY (400 MHz, $CDCl_3$)

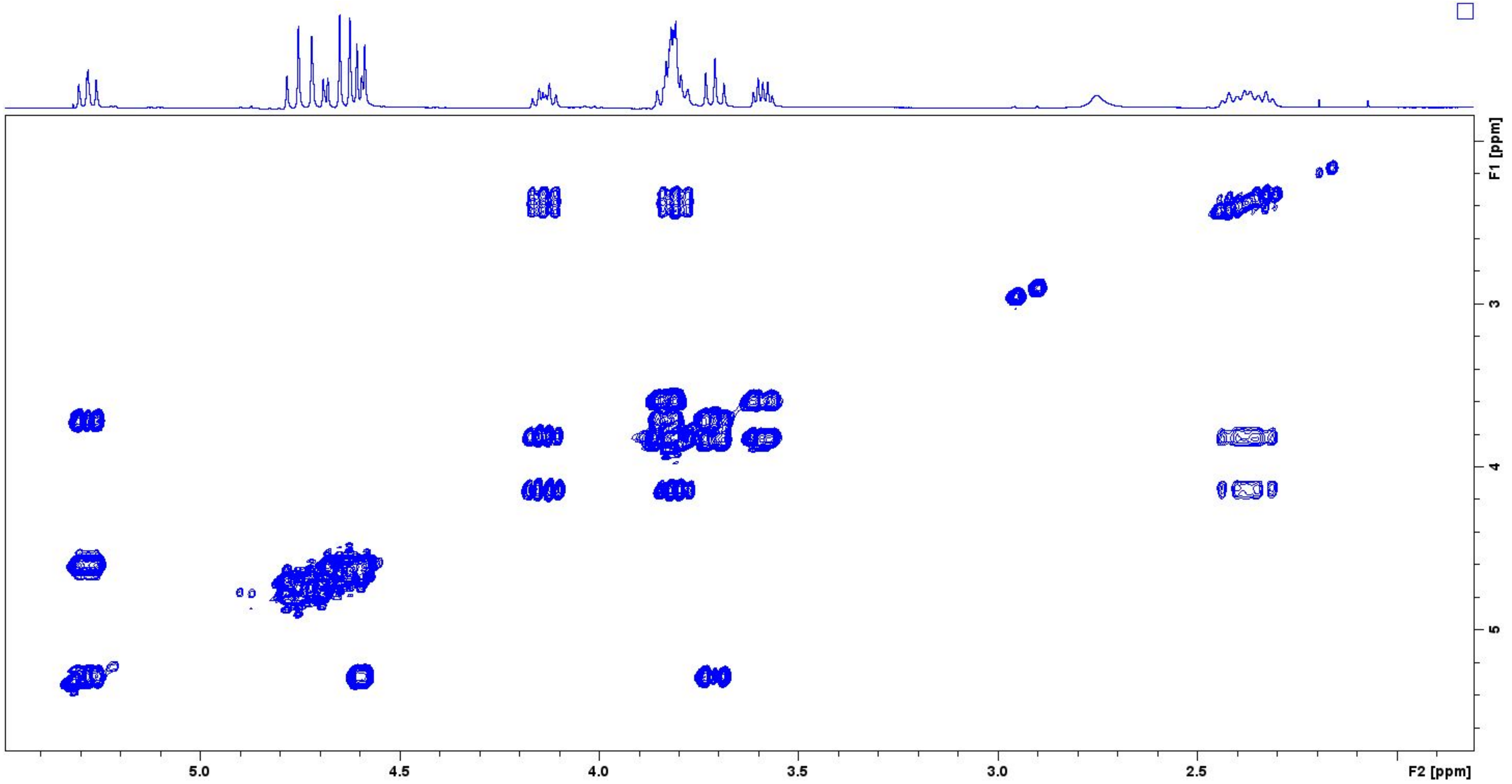

Compound 22 ^{1}H-NMR (400 MHz, $CDCl_3$)

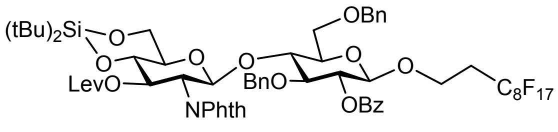

22

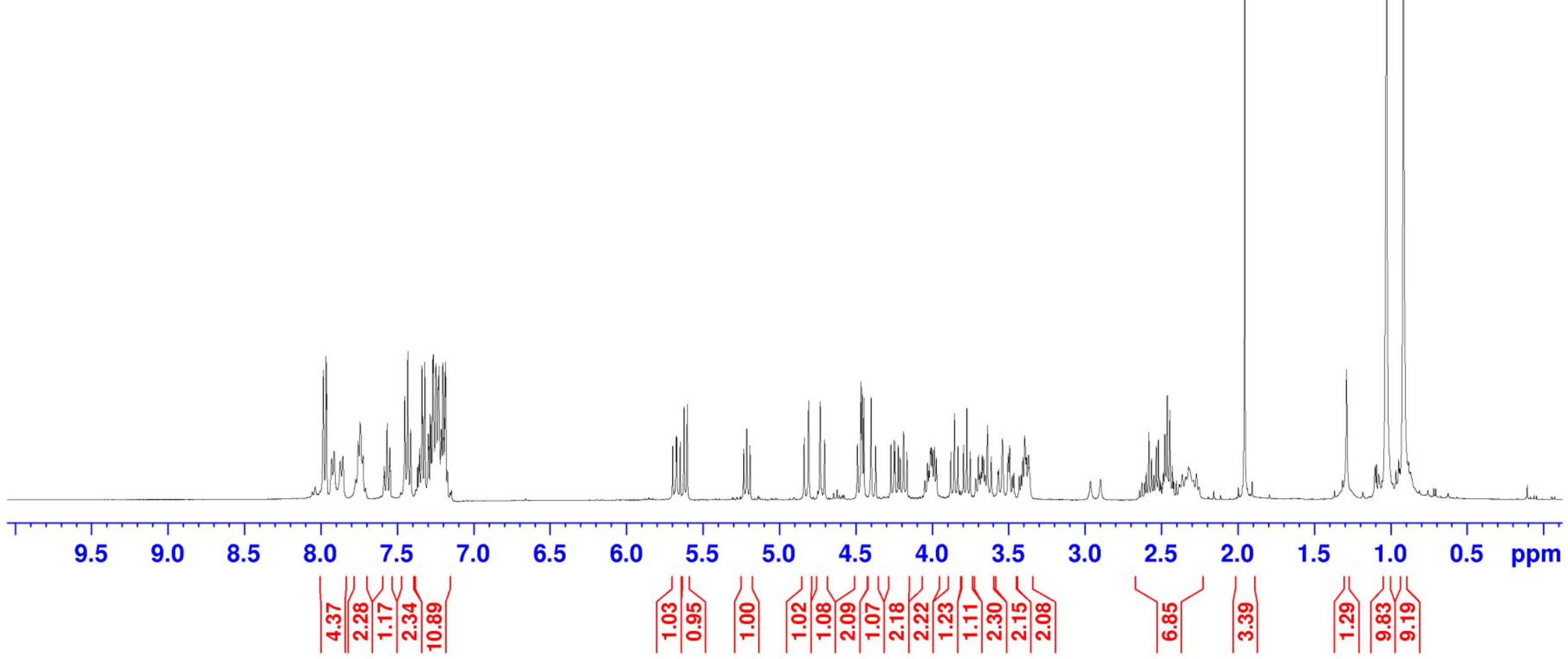

Compound 22 ^{1}H-NMR (400 MHz, $CDCl_3$)

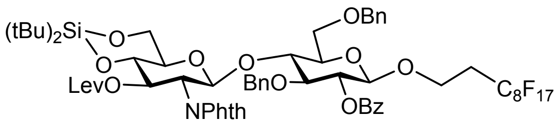

22

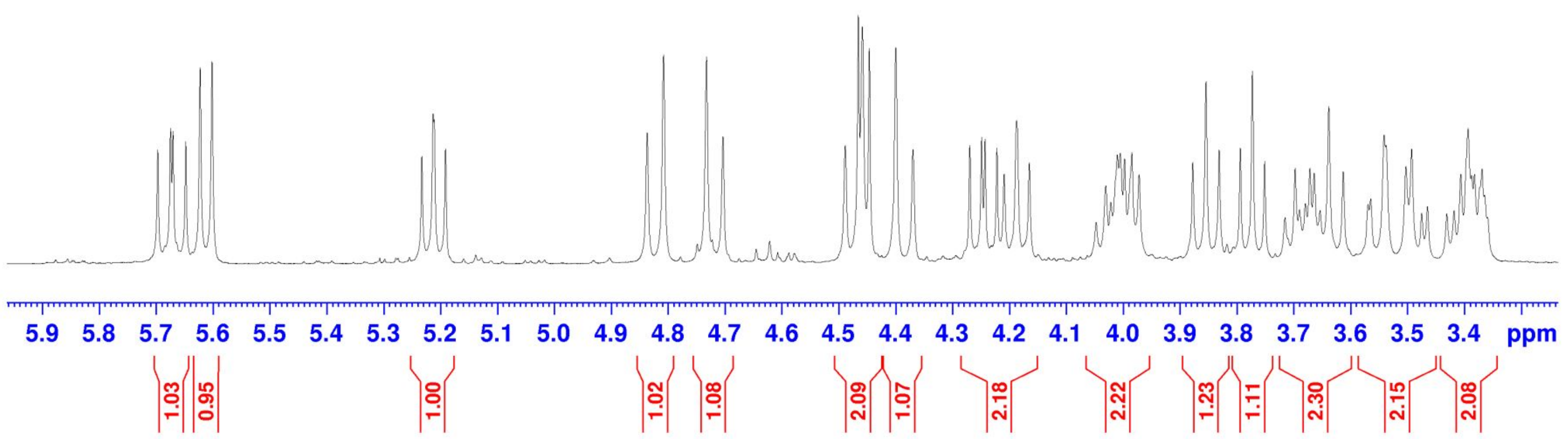

Compound 22 HSQC (400 MHz, $CDCl_3$)

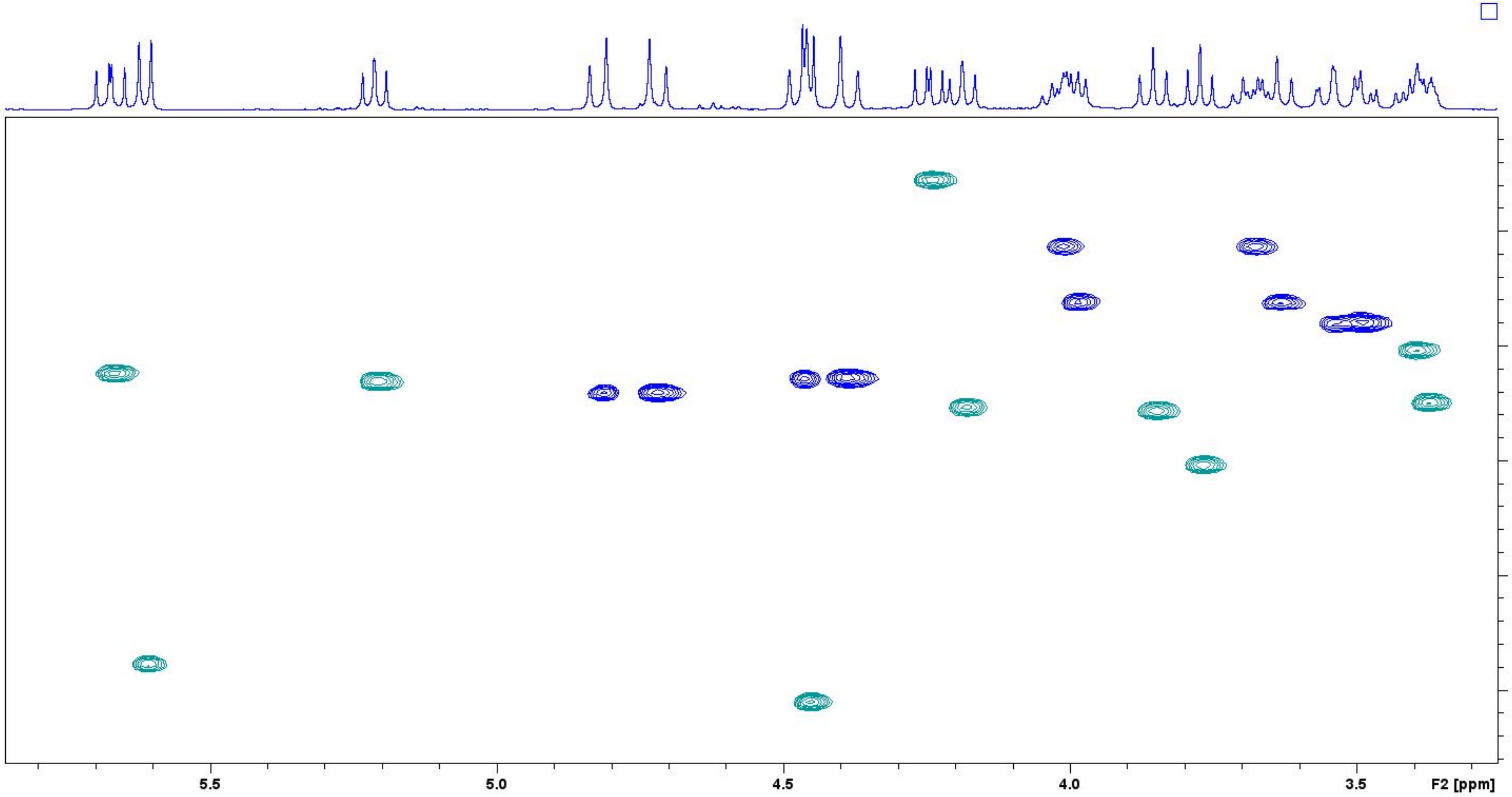

Compound 23 ^{1}H-NMR (400 MHz, $CDCl_3$)

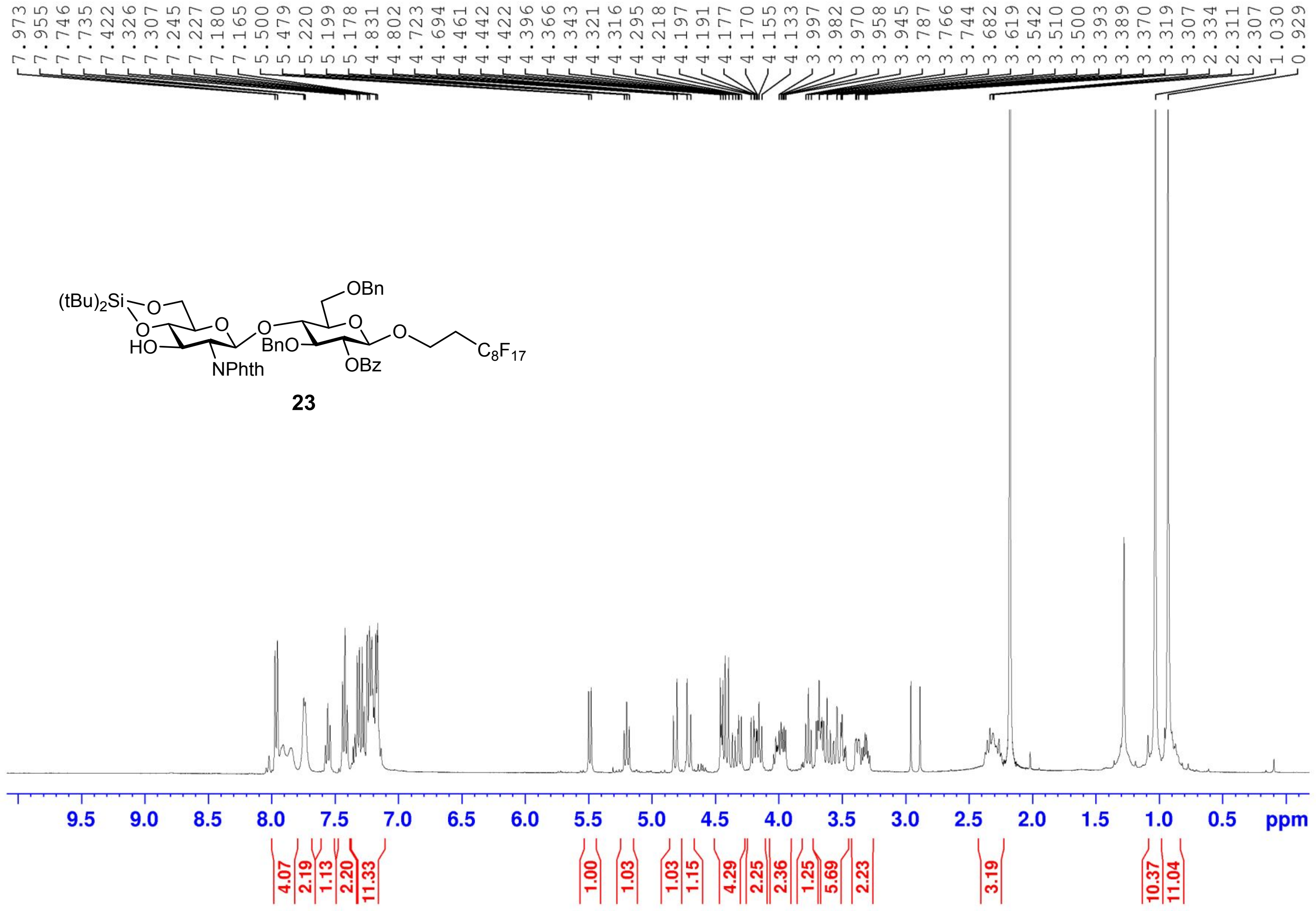

Compound 23 ^{1}H-NMR (400 MHz, $CDCl_3$)

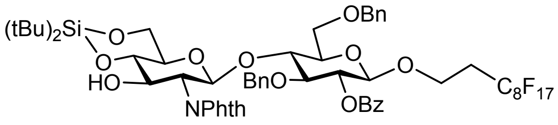

23

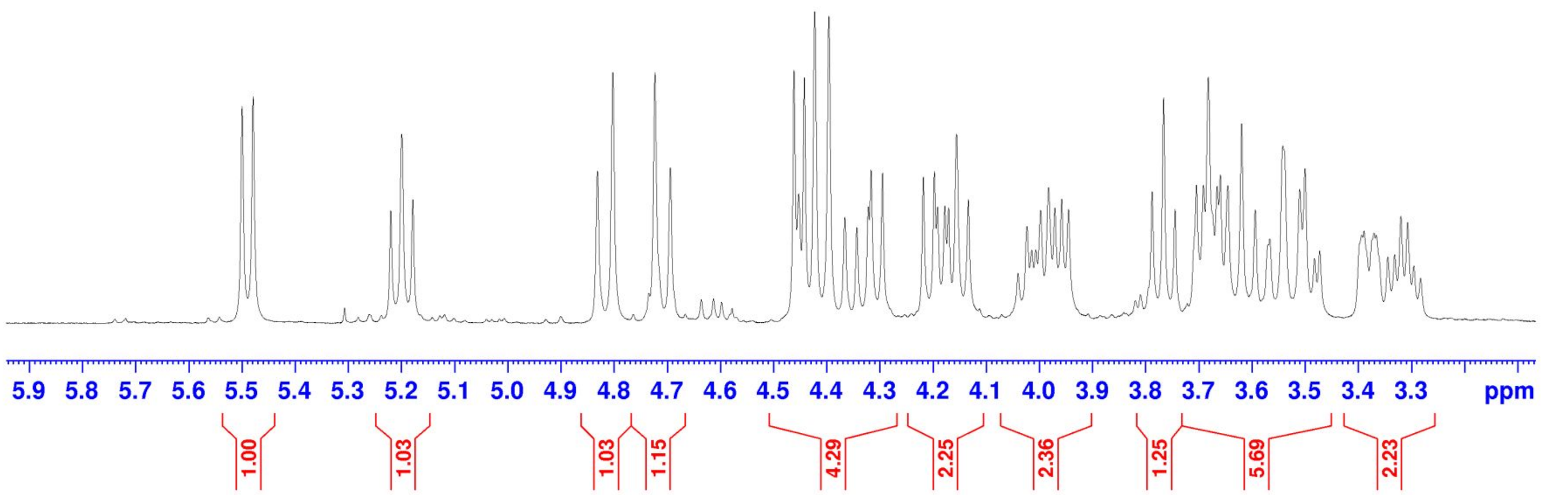

Compound 23 HSQC (400 MHz, $CDCl_3$)

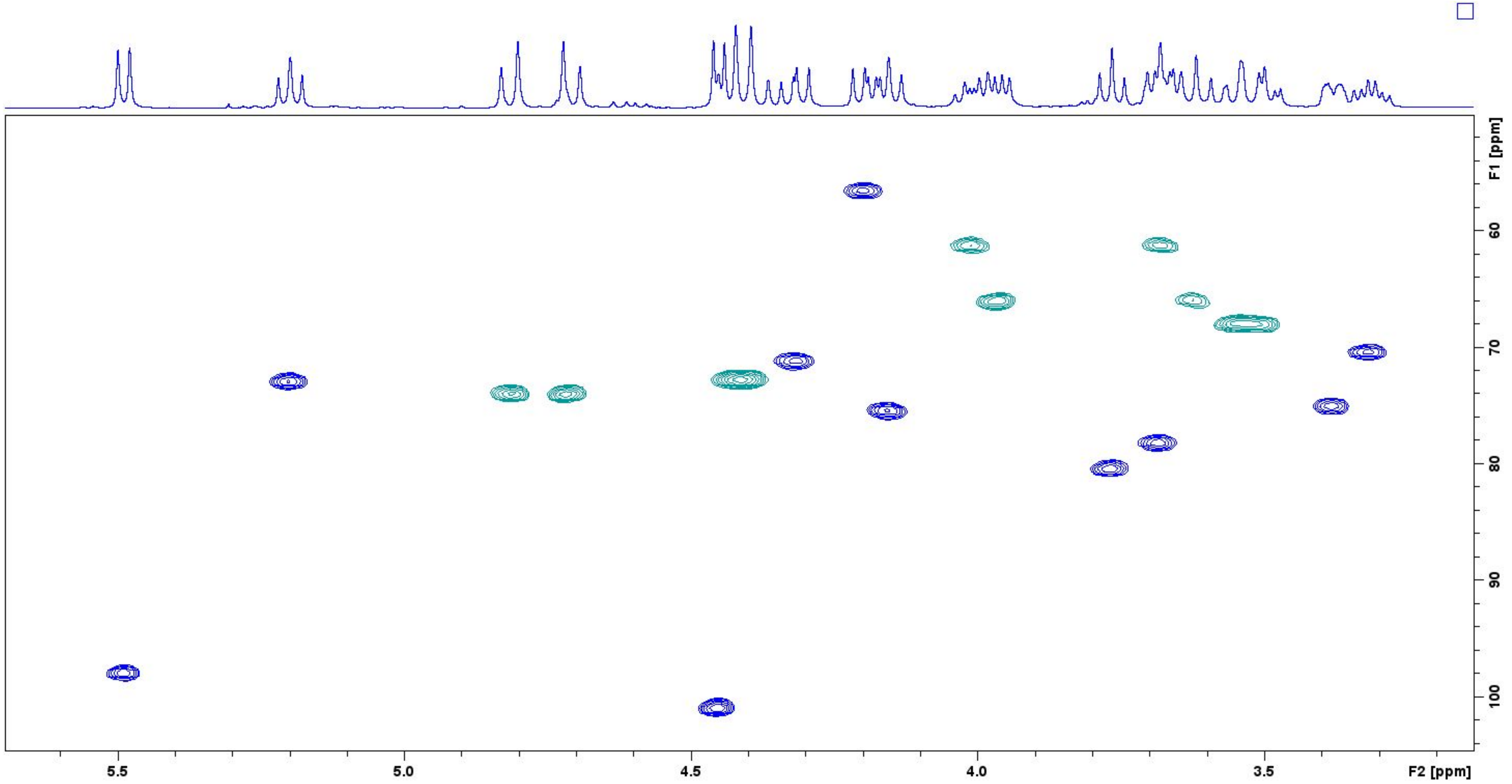

Compound 24 ^{1}H-NMR (400 MHz, $CDCl_3$)

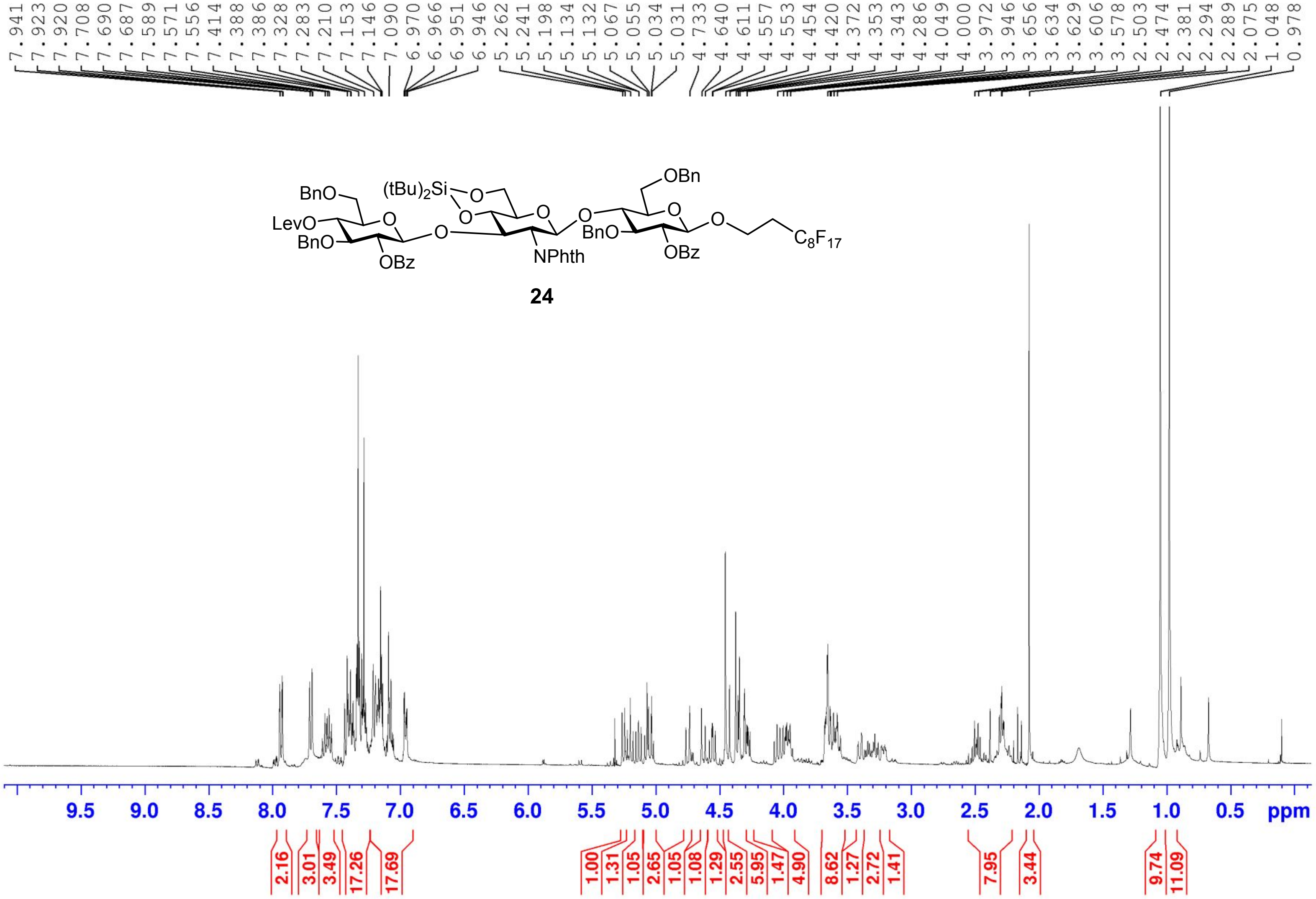

Compound 24 ^{1}H-NMR (400 MHz, $CDCl_3$)

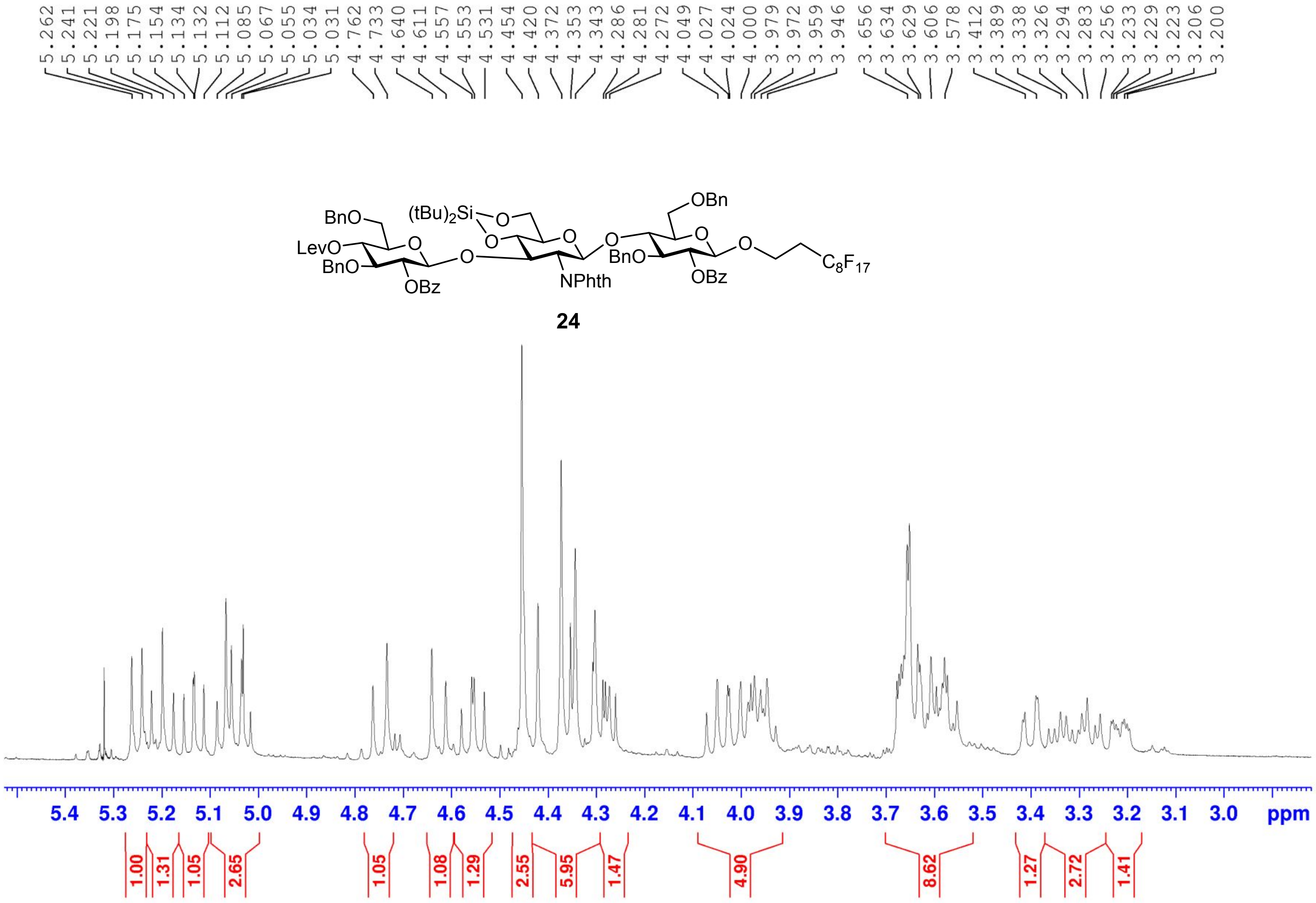

Compound 24 HSQC (400 MHz, $CDCl_3$)

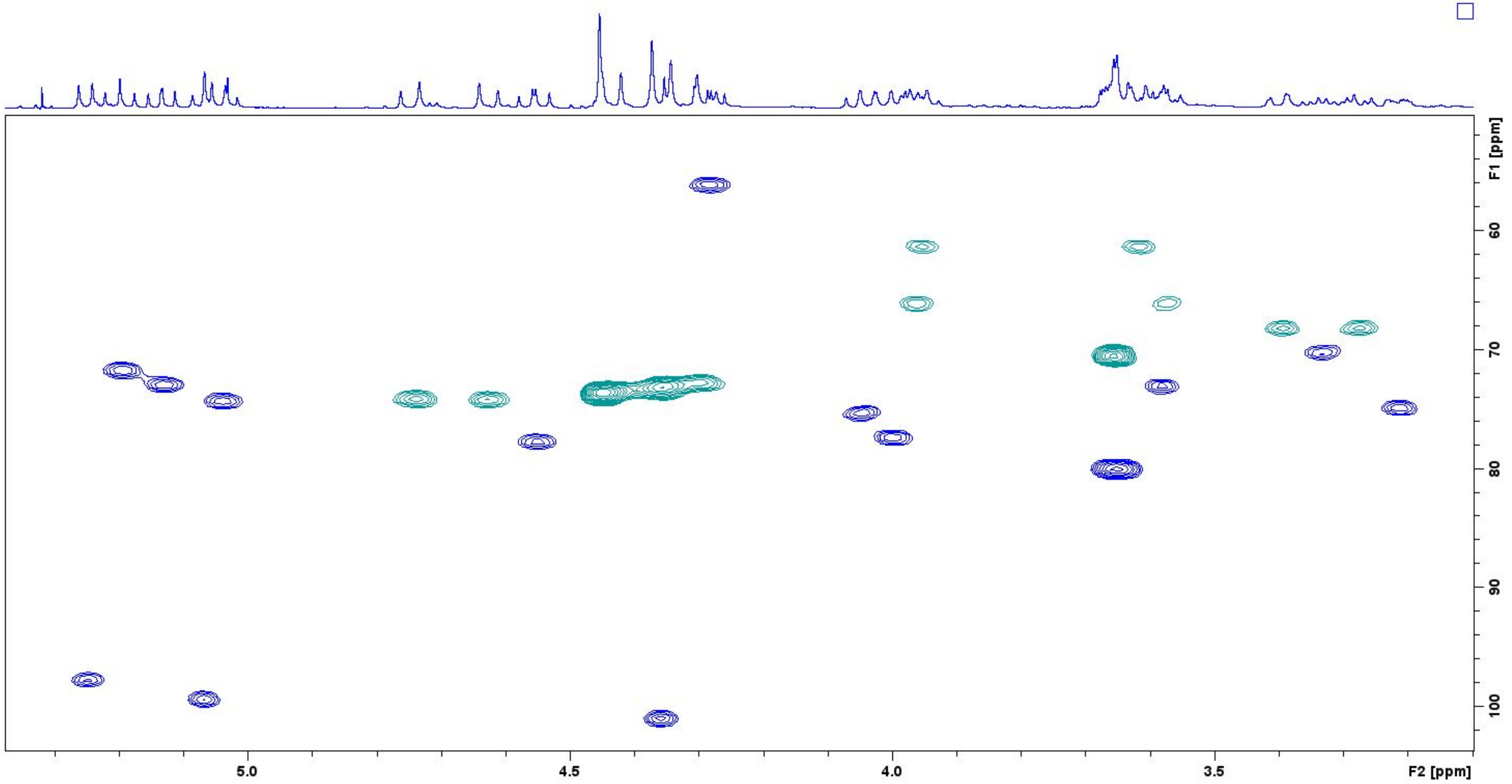

Compound 26 ¹H-NMR (400 MHz, $CDCl_3$)

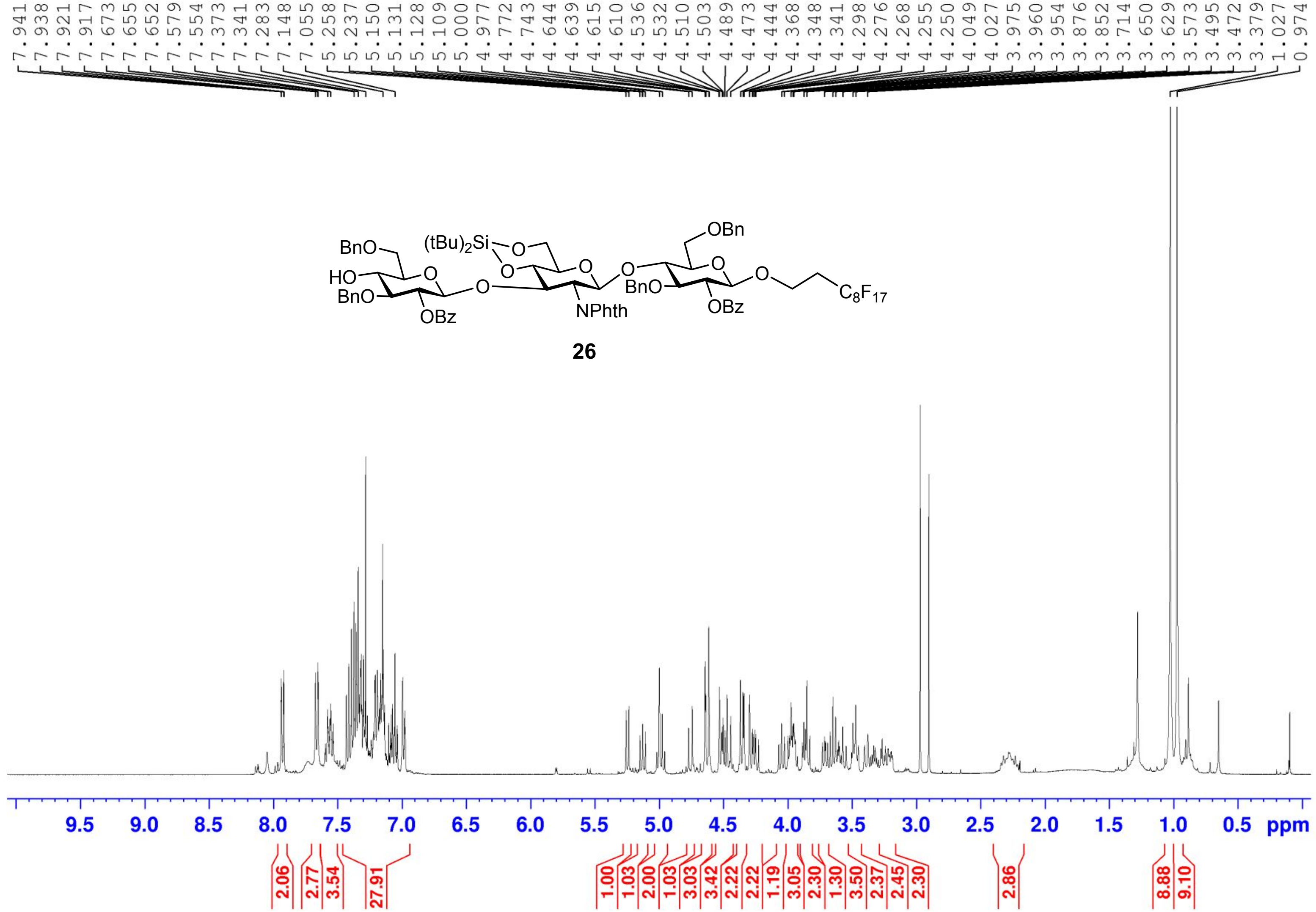

26

Compound 26 ^{1}H-NMR (400 MHz, $CDCl_3$)

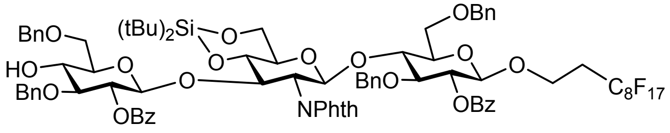

26

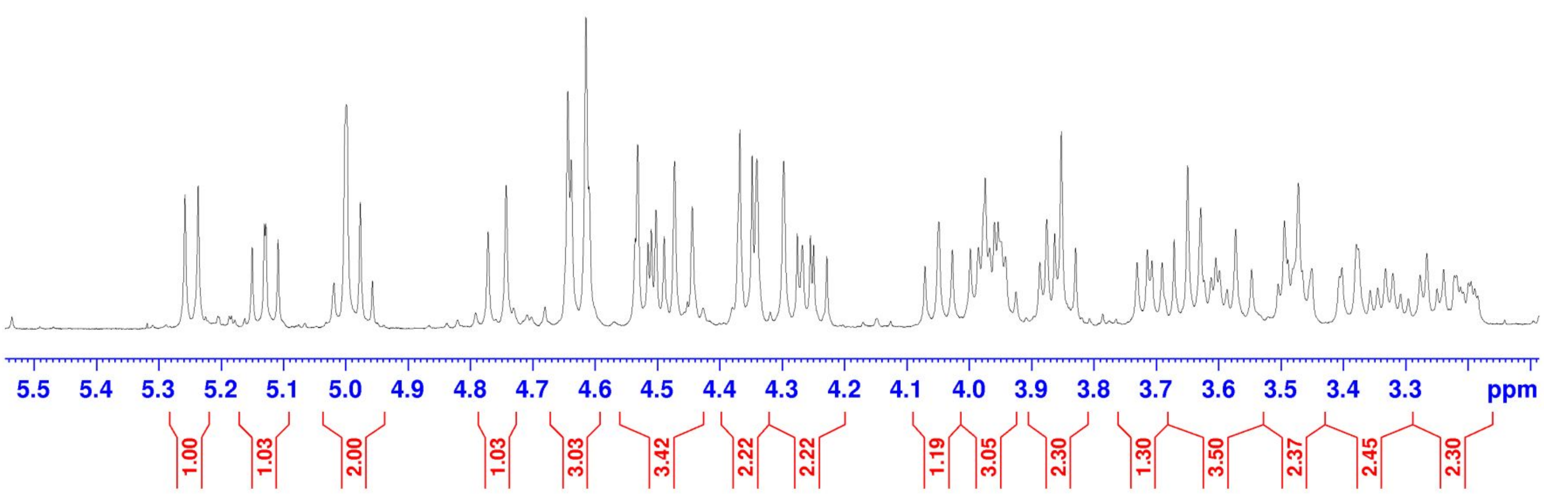

Compound 26 HSQC (400 MHz, $CDCl_3$)

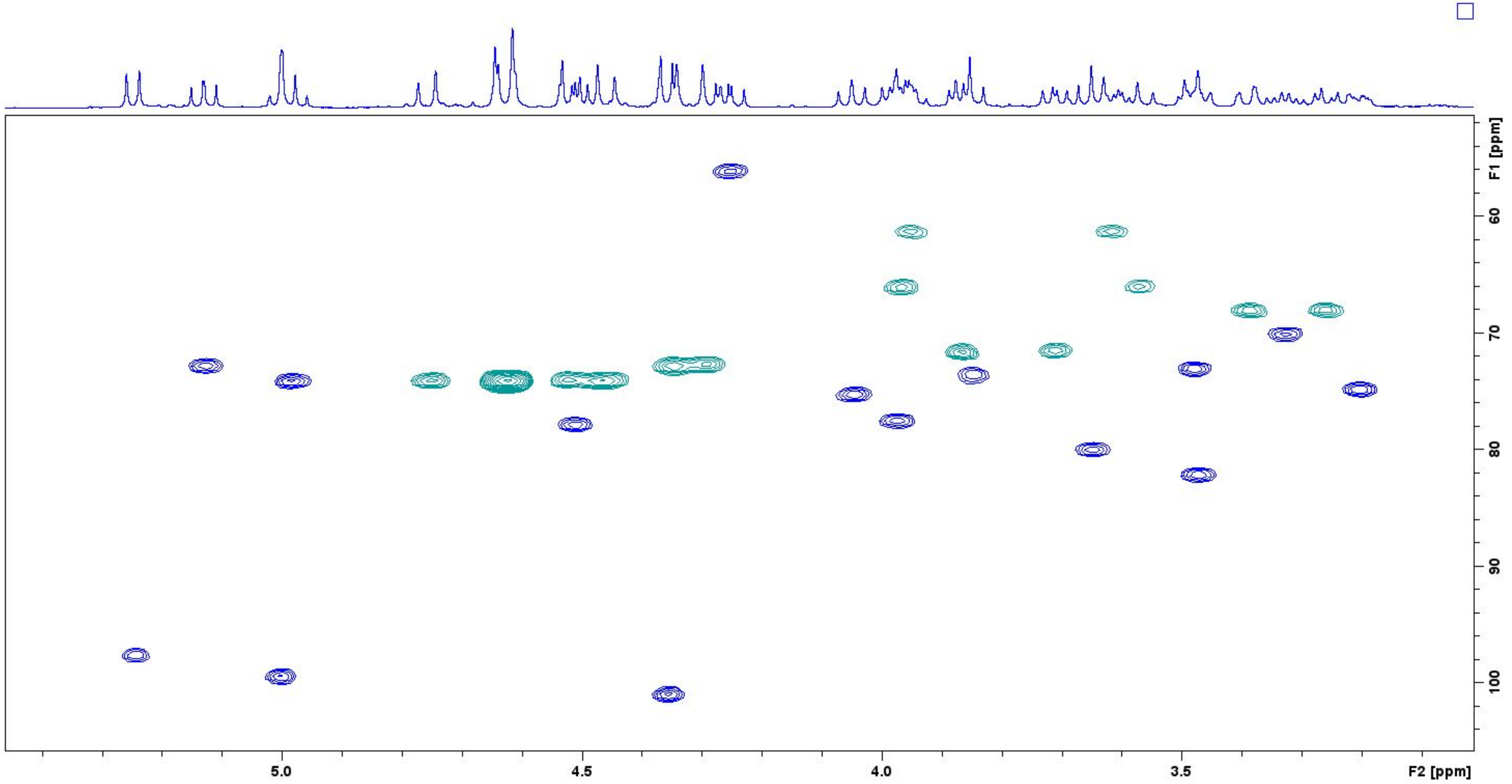

Compound 27 ^{1}H-NMR (400 MHz, $CDCl_3$)

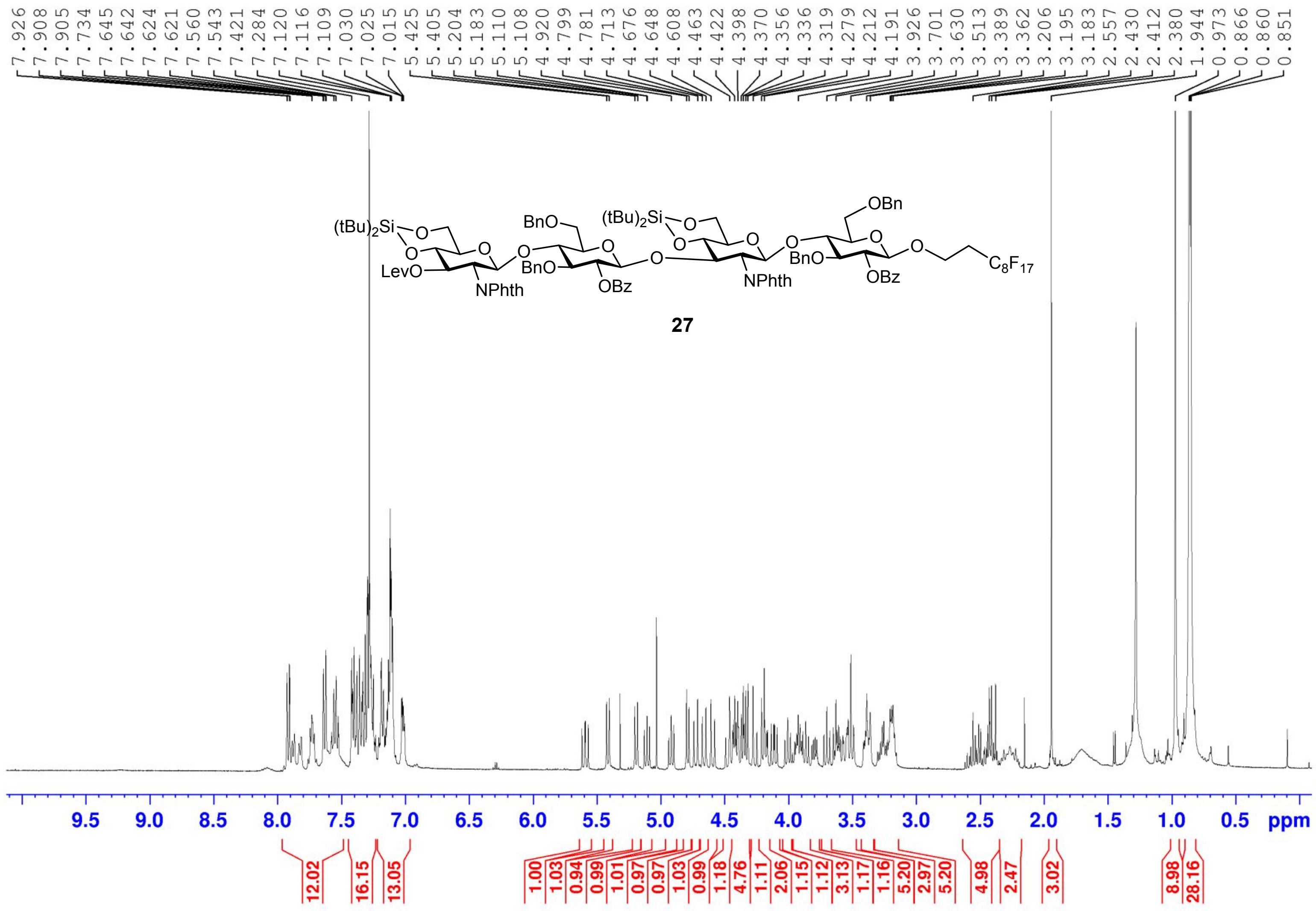

Compound 27 ^{1}H-NMR (400 MHz, $CDCl_3$)

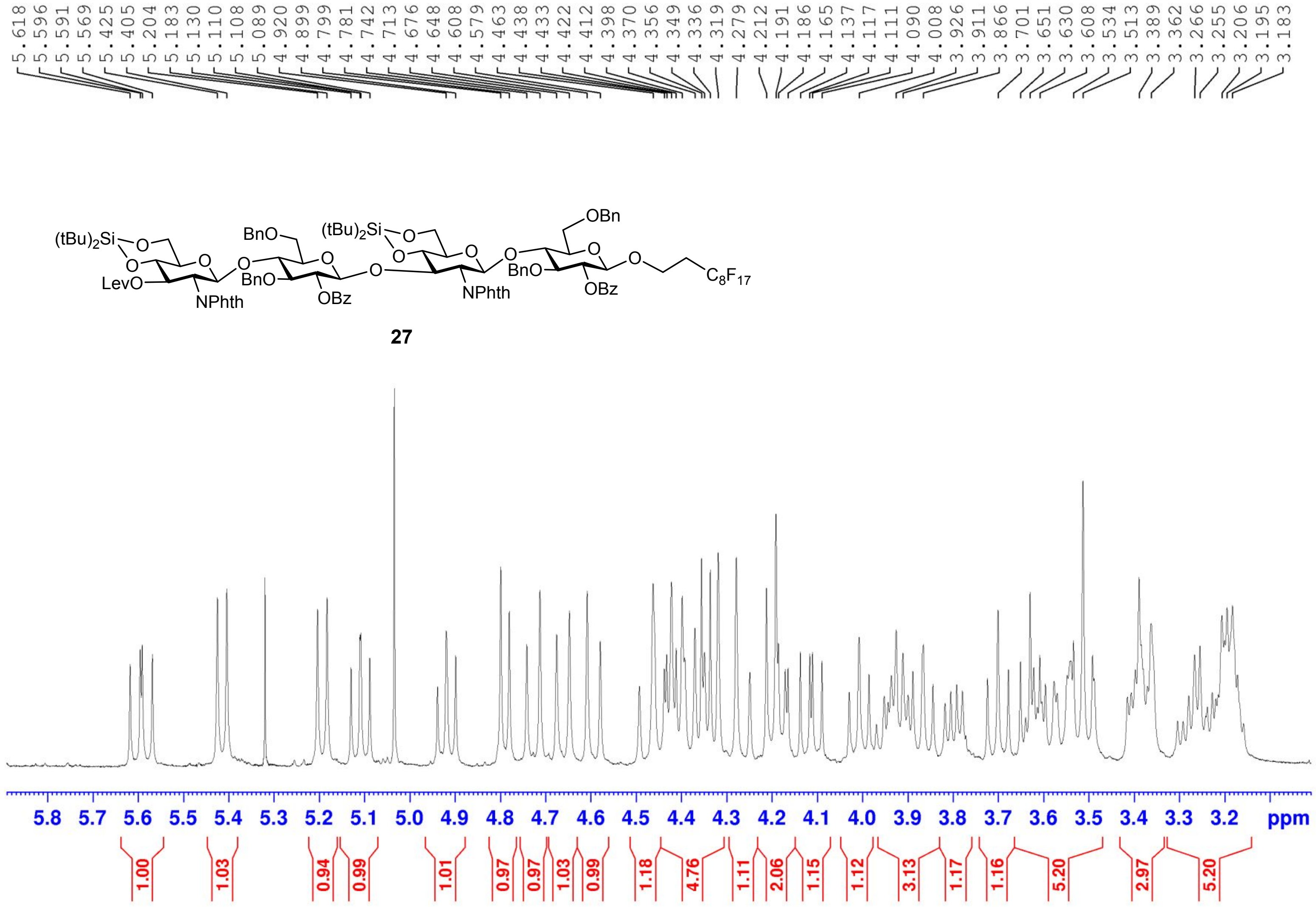

27

Compound 27 HSQC (400 MHz, $CDCl_3$)

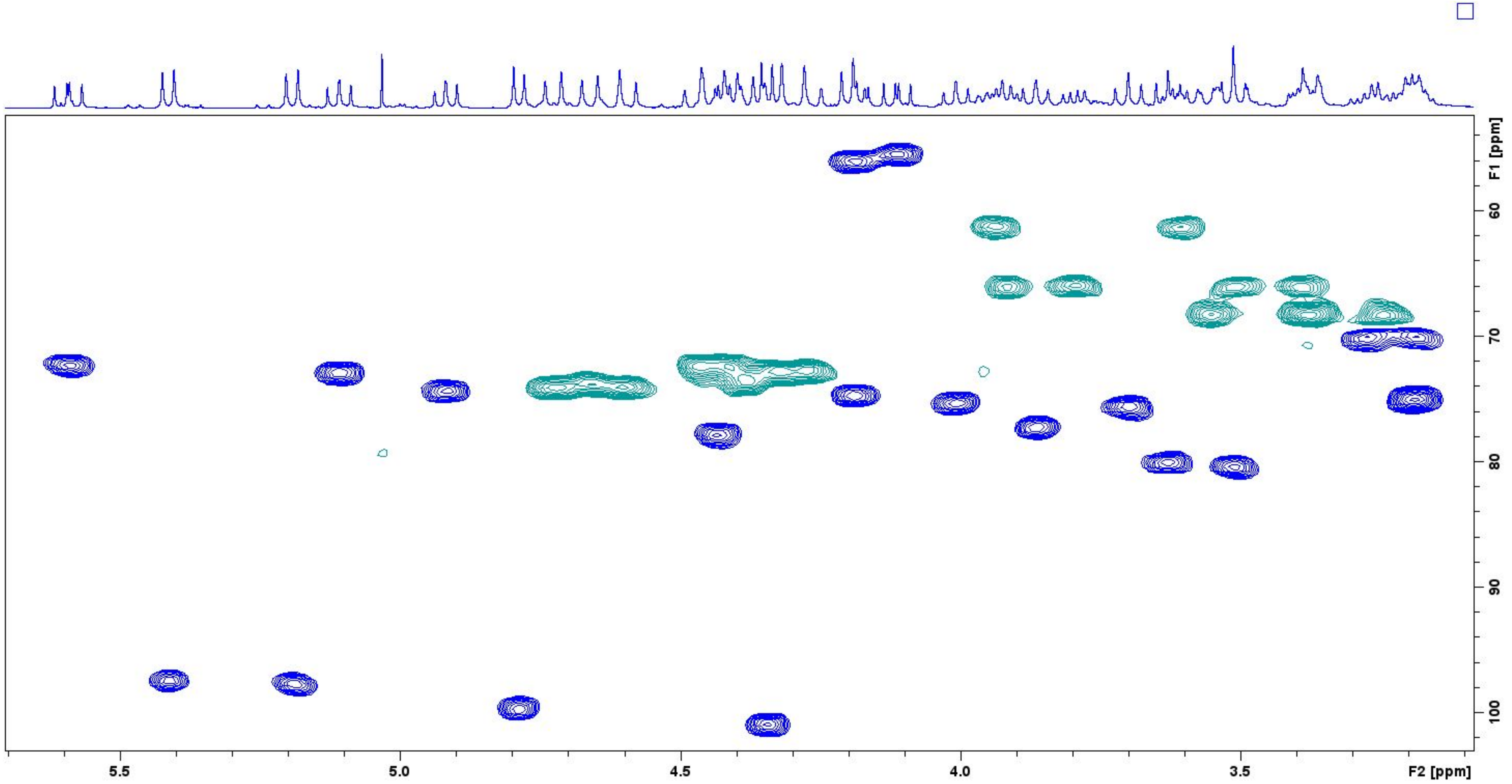

Compound 27 $^{13}C\{^{1}H\}$ NMR (100 MHz, $CDCl_3$)

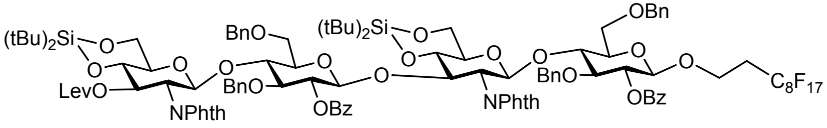

27

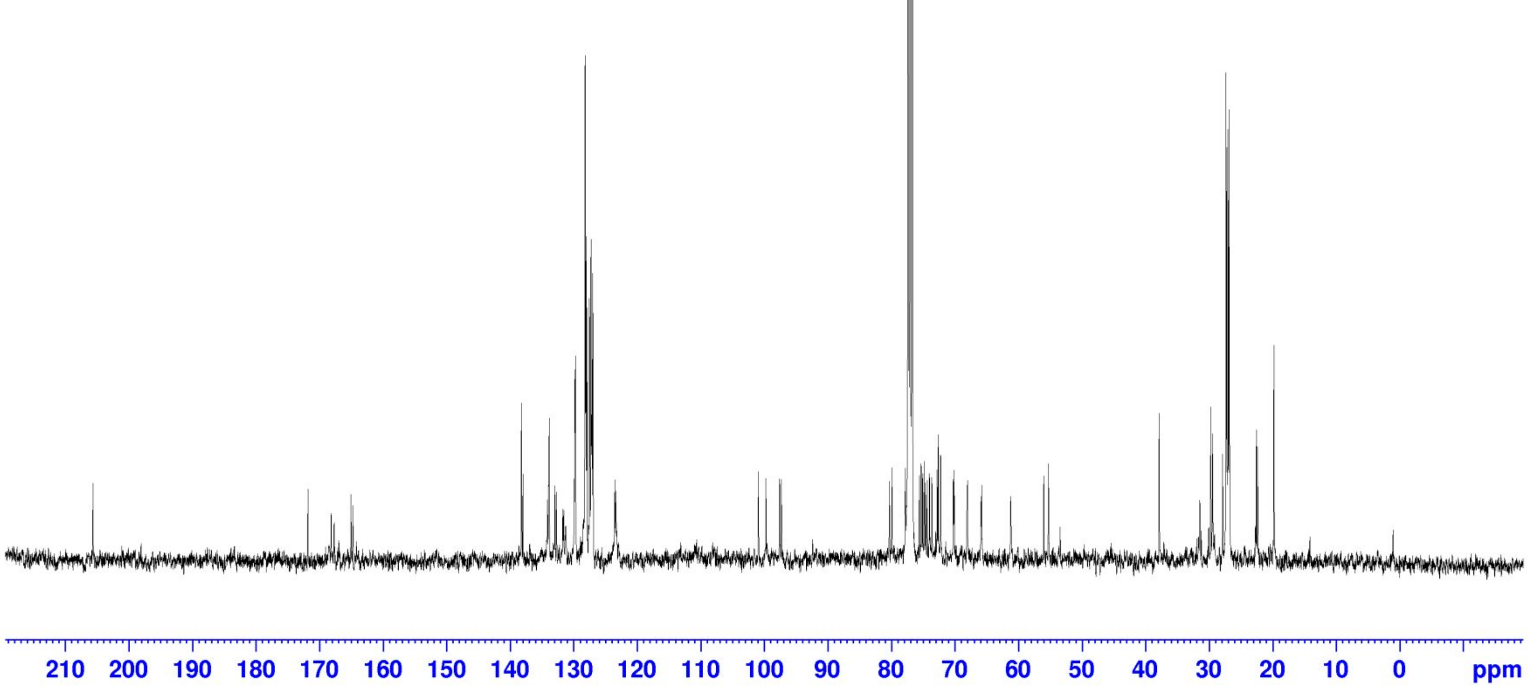

Compound 27 $^{13}C\{^1H\}$ NMR (100 MHz, $CDCl_3$)

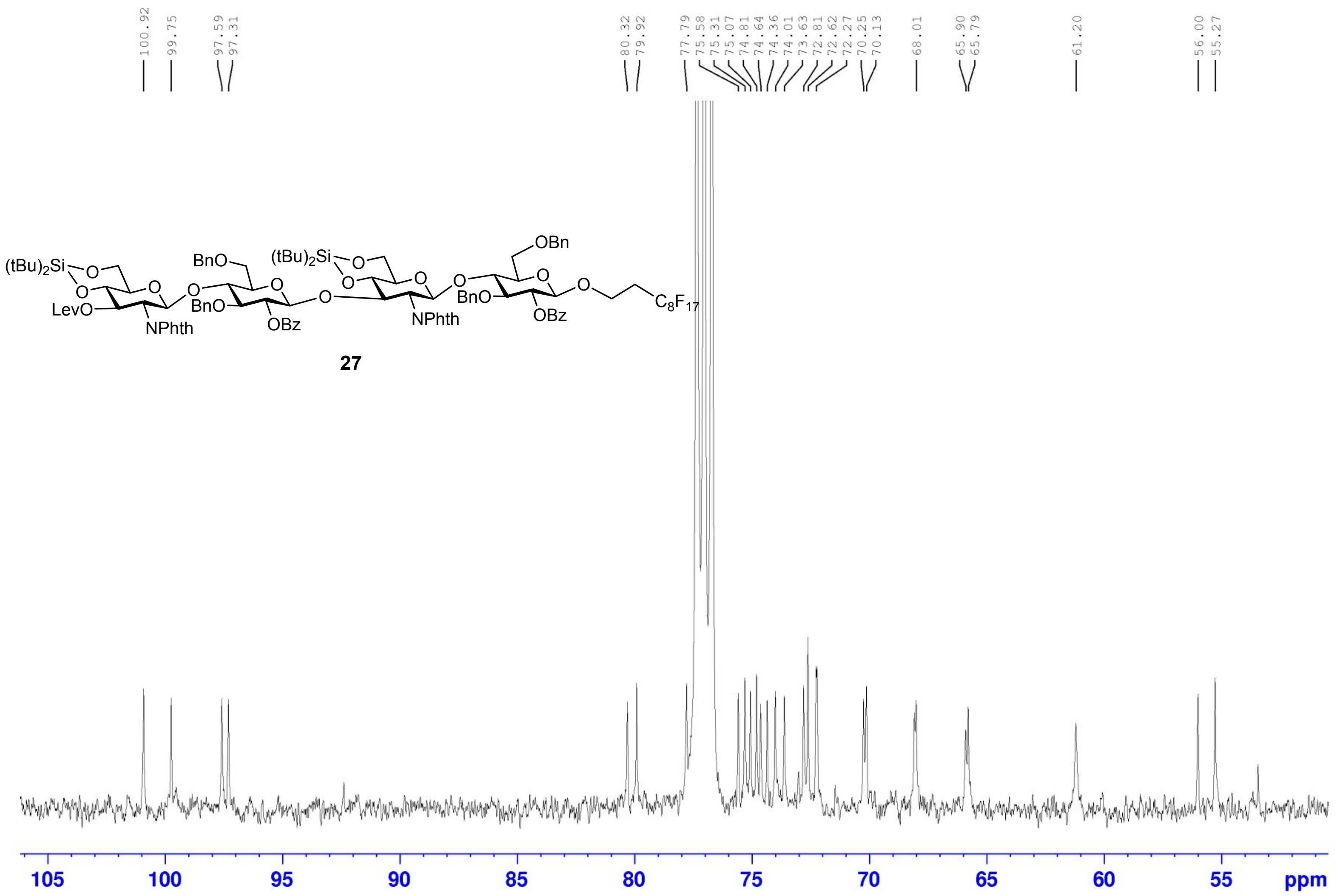

Compound 28 ^{1}H-NMR (400 MHz, CD_3OD)

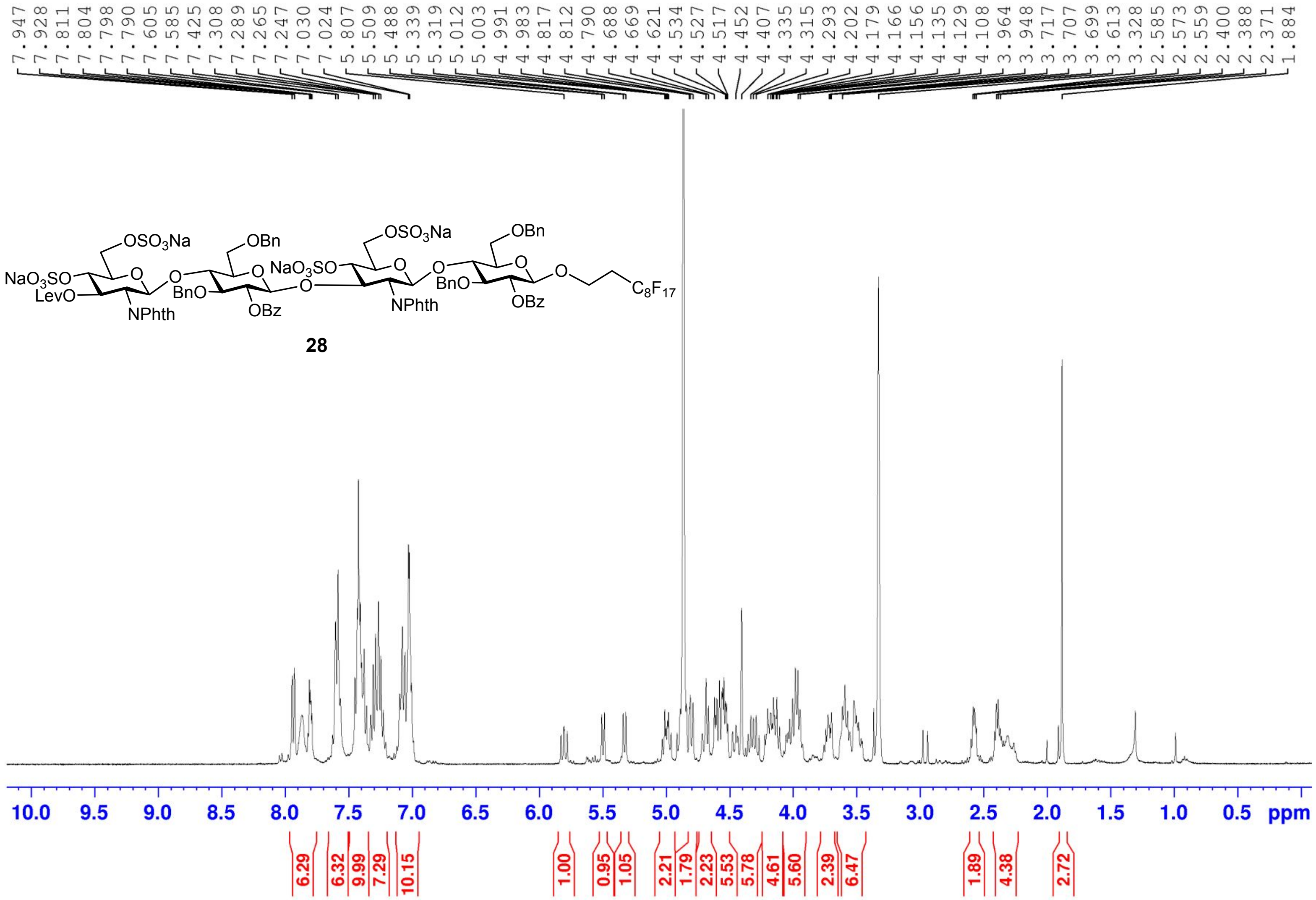

Compound 28 ^{1}H-NMR (400 MHz, CD_3OD)

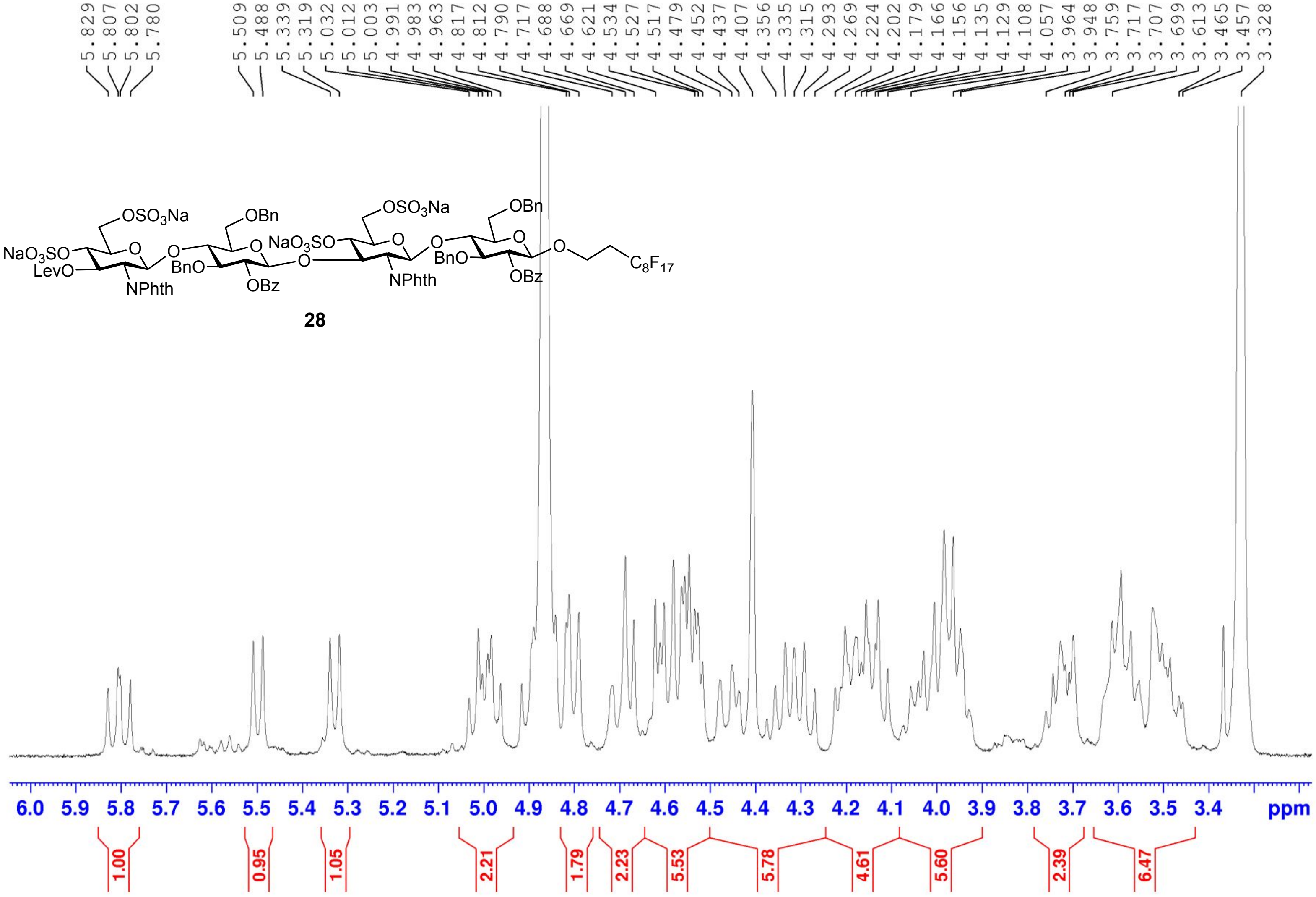

Compound 28 HSQC (400 MHz, CD_3OD)

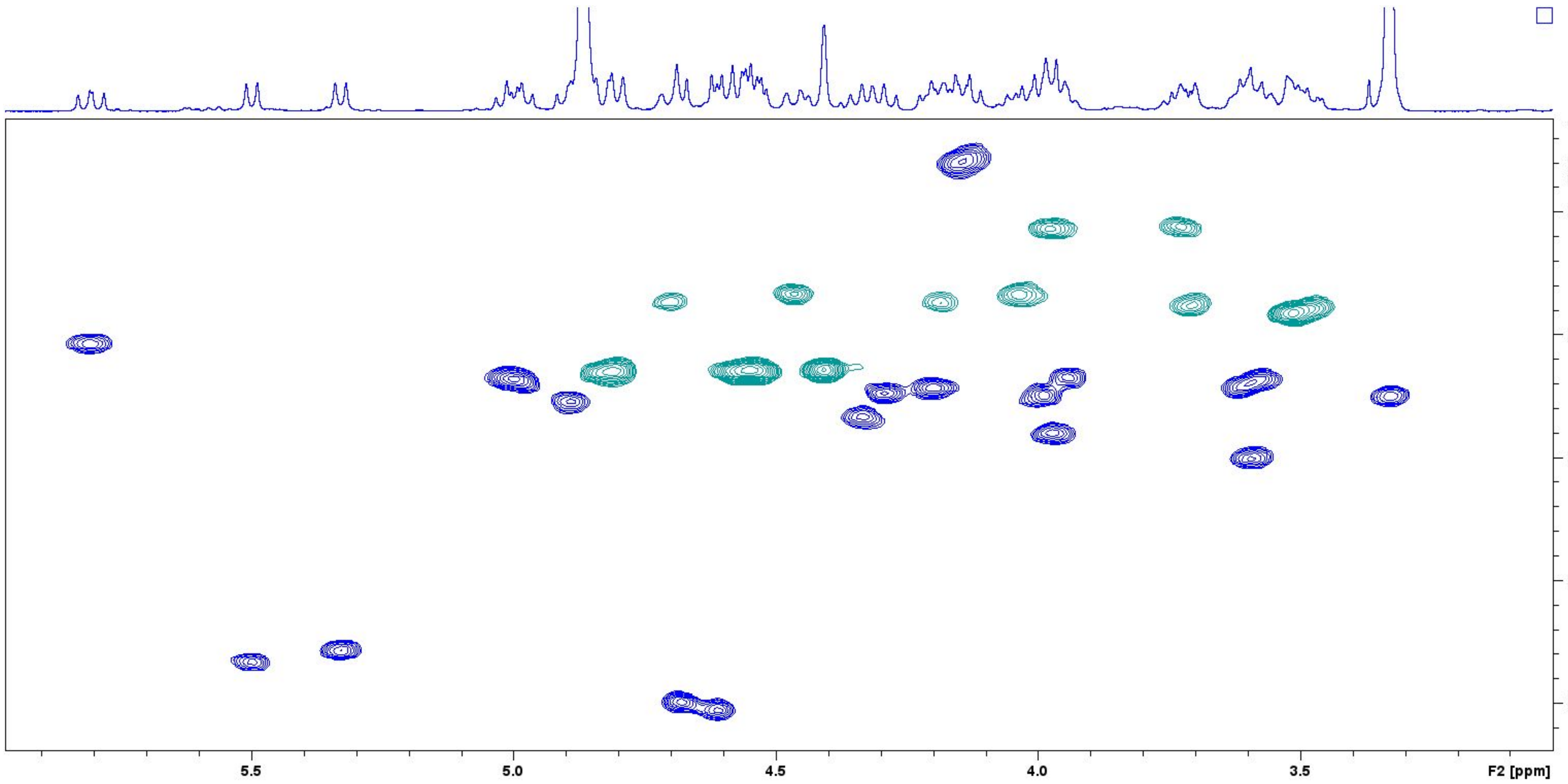

Compound 28 $^{13}C\{^{1}H\}$ NMR (100 MHz, CD_3OD)

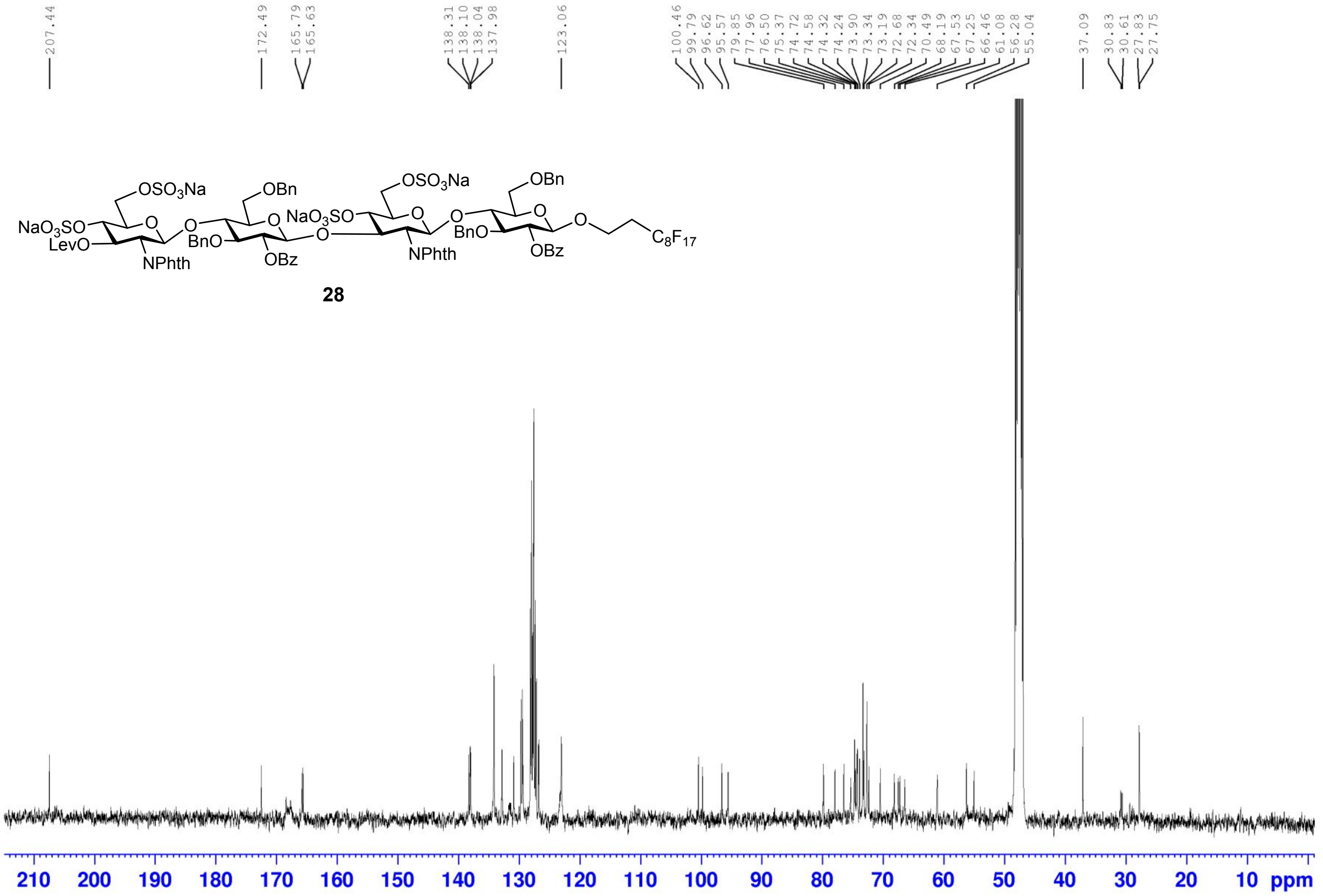

Compound 28 $^{13}C\{^{1}H\}$ NMR (100 MHz, CD_3OD)

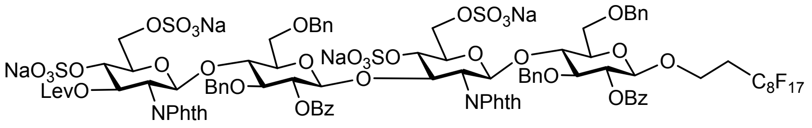

28

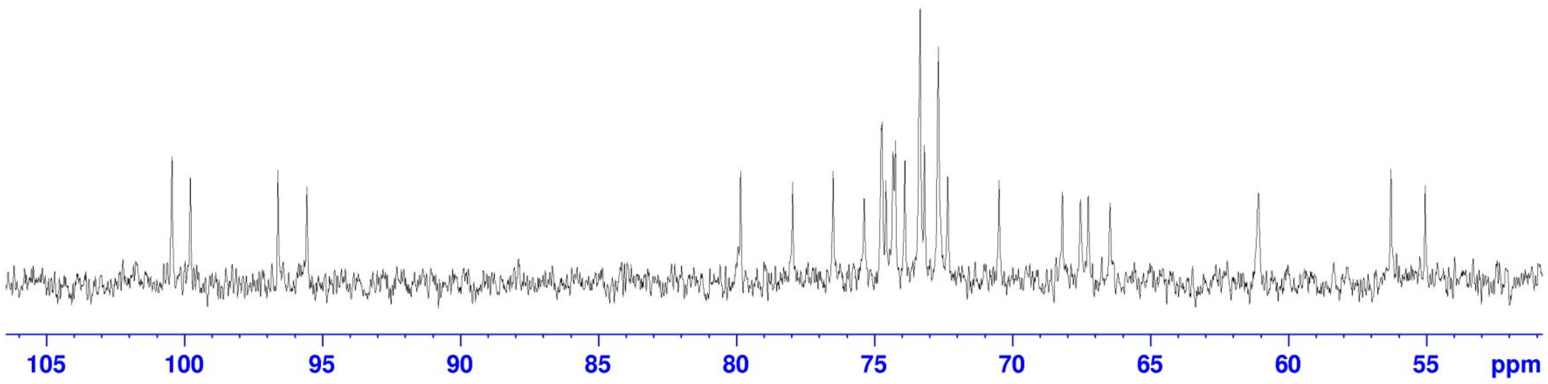

Compound 3 ^{1}H-NMR (400 MHz, CD_3OD)

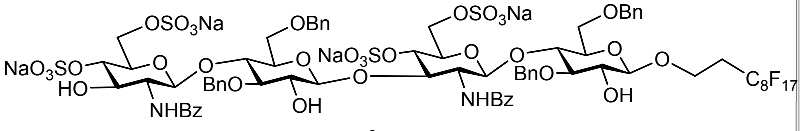

3

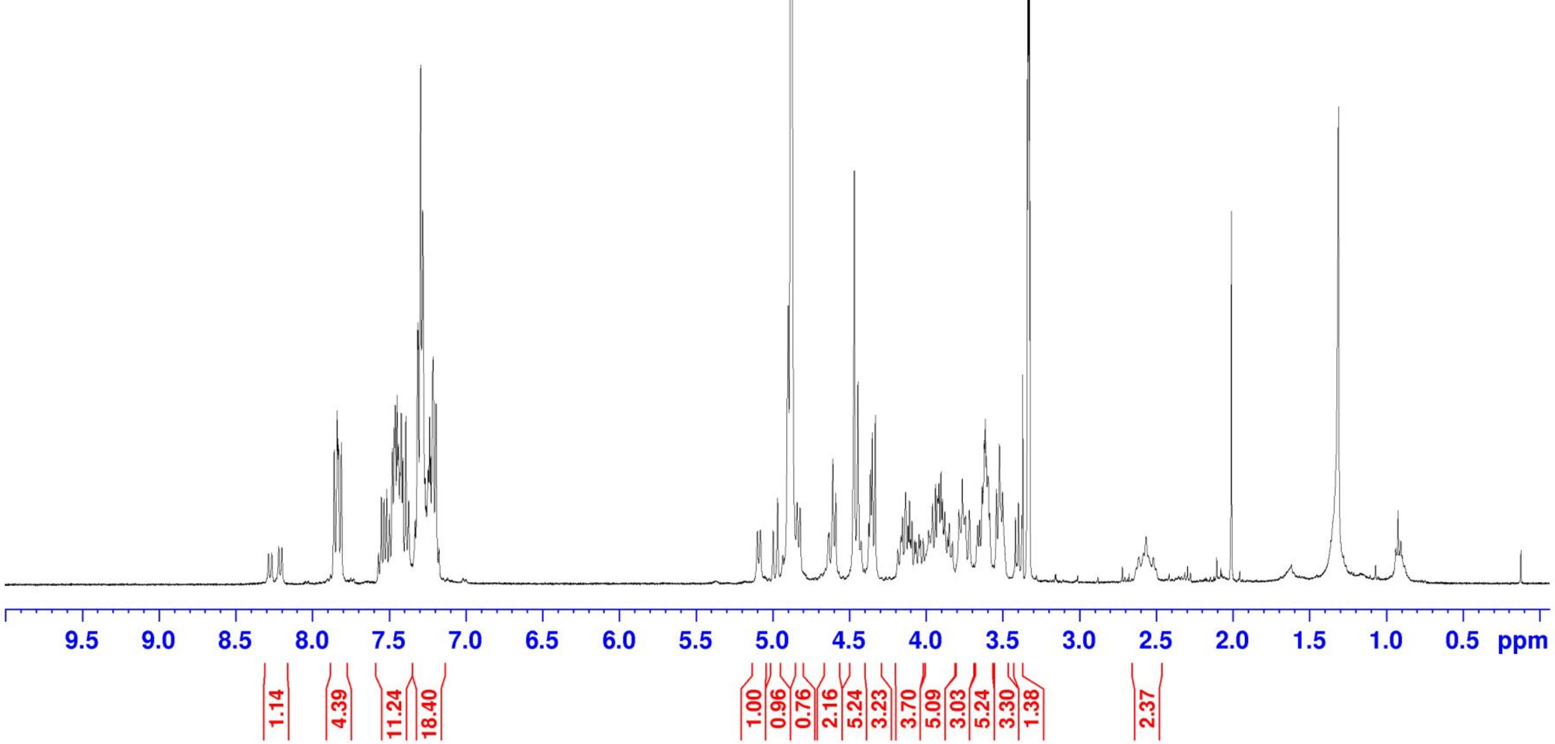

Compound 3 ^{1}H-NMR (400 MHz, CD_3OD)

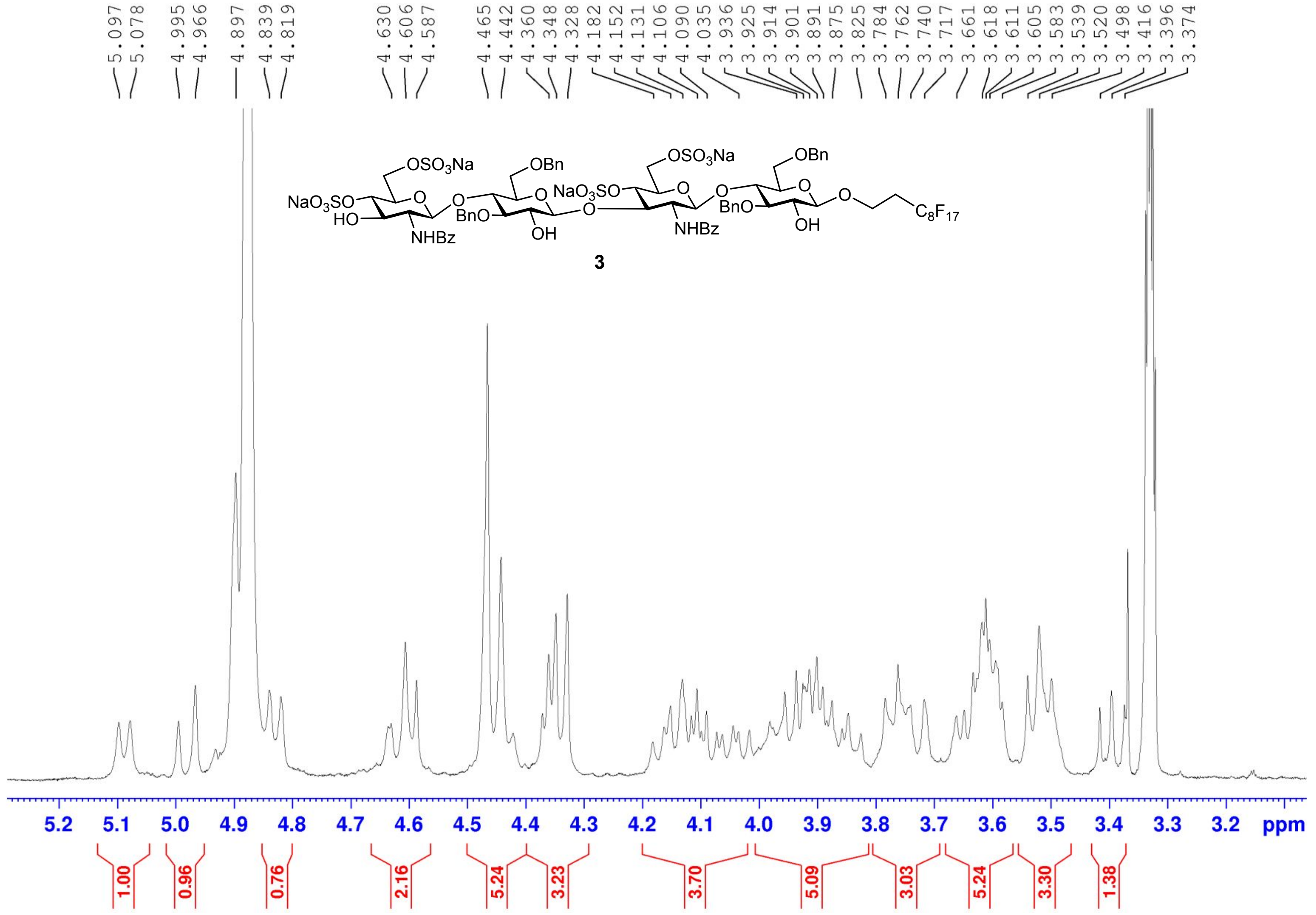

Compound 3 COSY (400 MHz, CD_3OD)

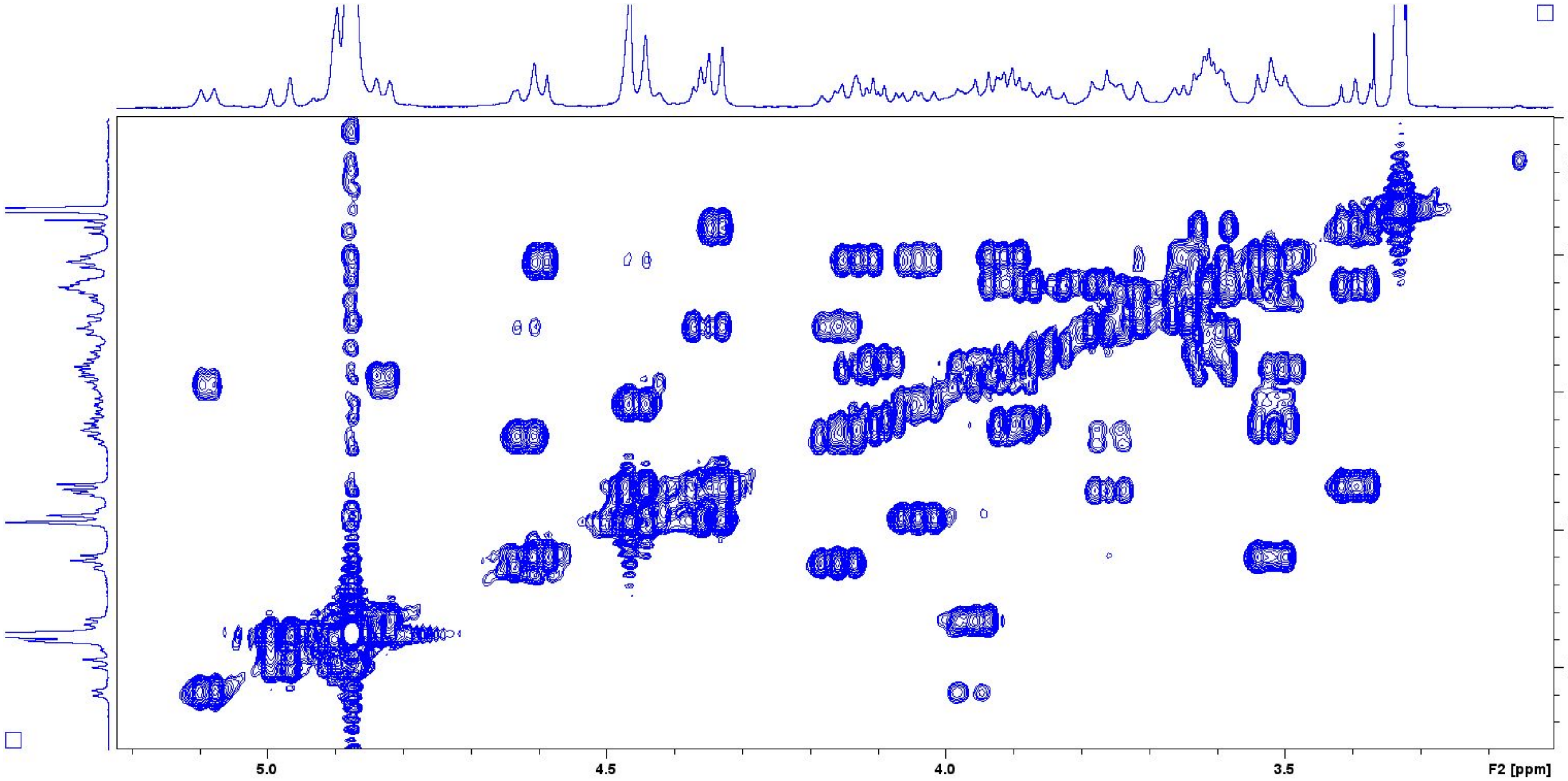

Compound 3 HSQC (400 MHz, CD_3OD)

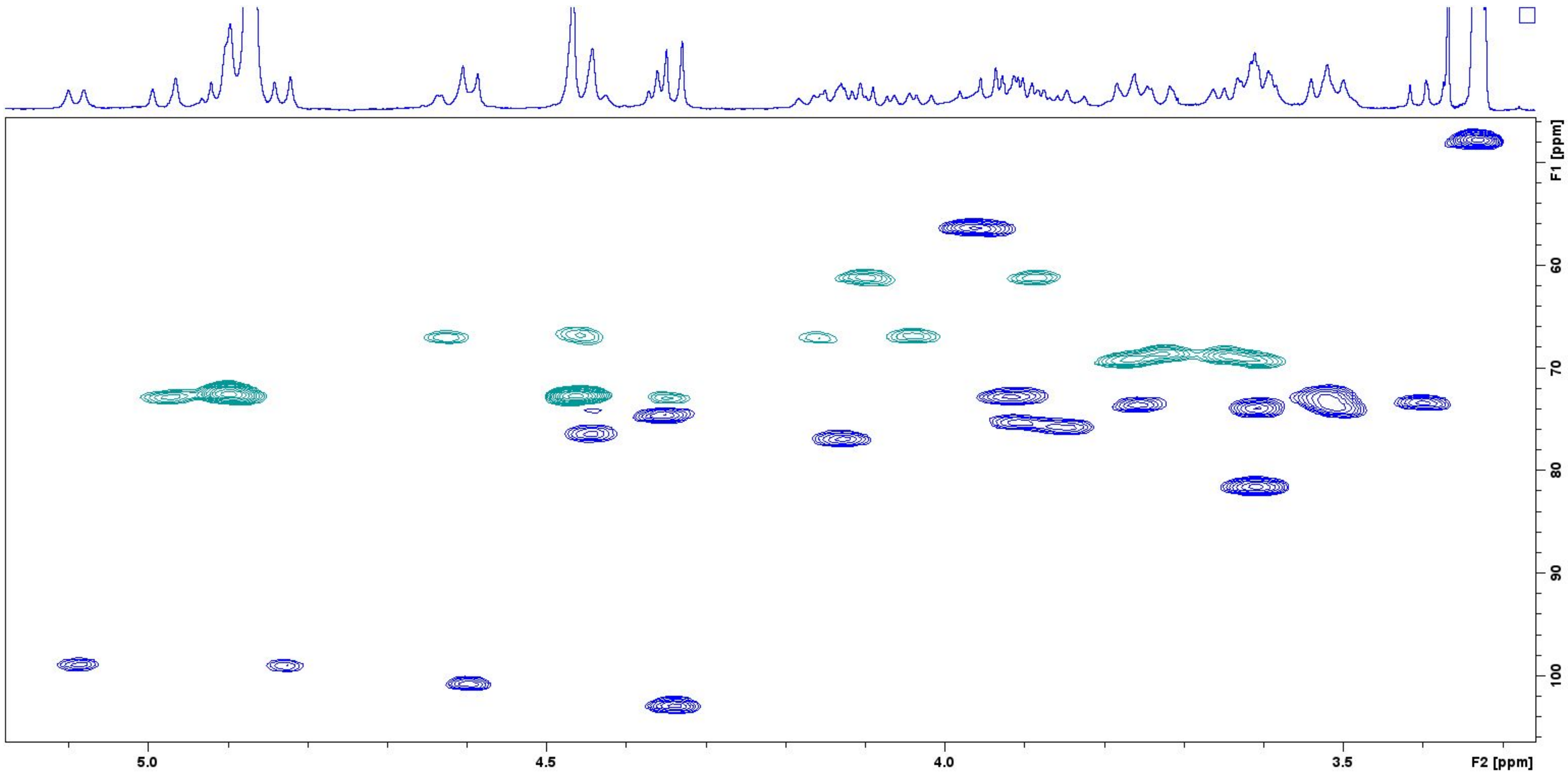

Compound 3 $^{13}C\{^{1}H\}$ NMR (100 MHz, CD_3OD)

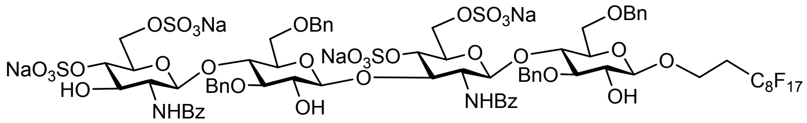

3

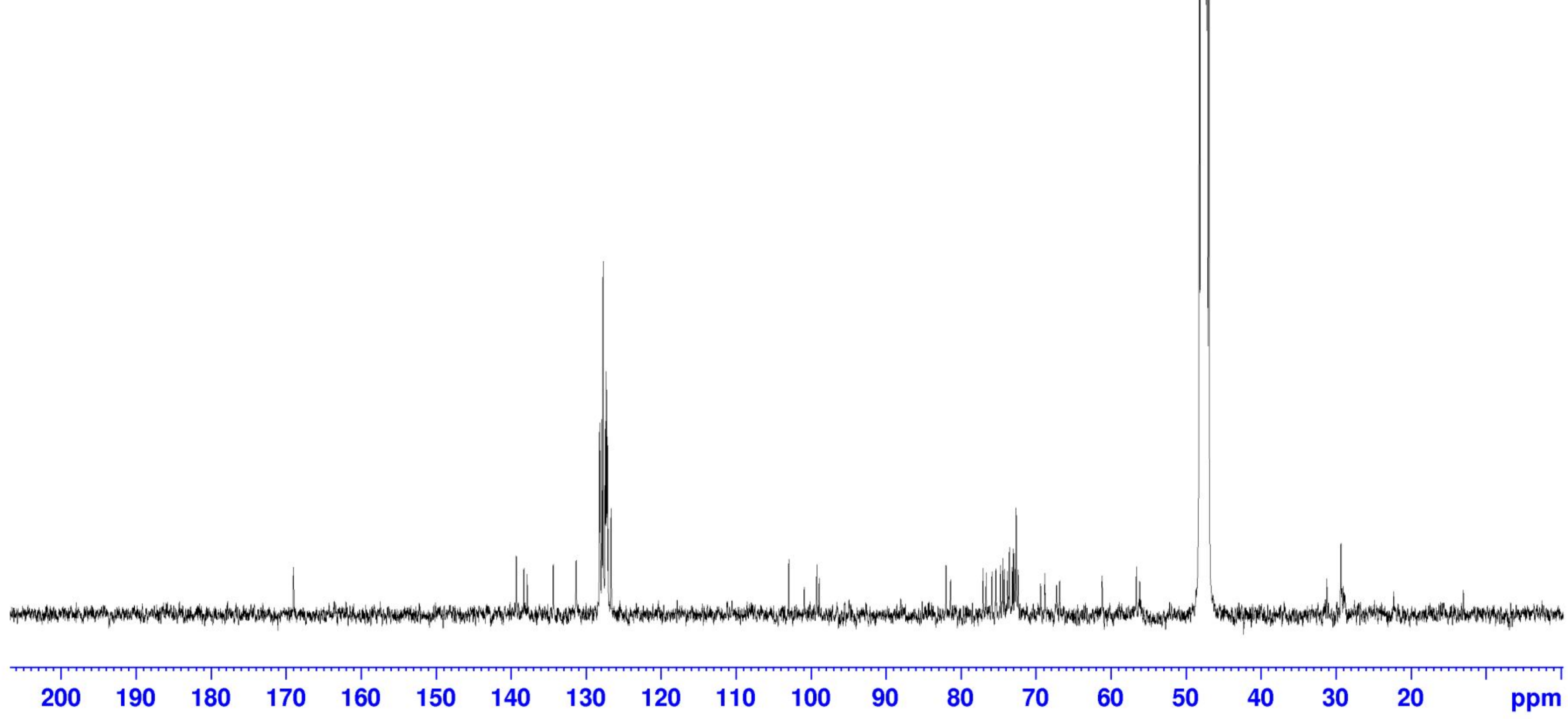

Compound 3 $^{13}C\{^{1}H\}$ NMR (100 MHz, CD_3OD)

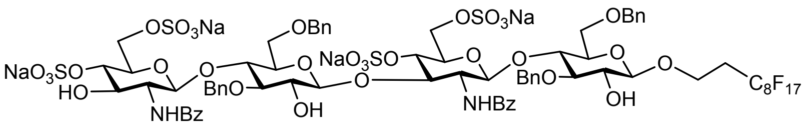

3

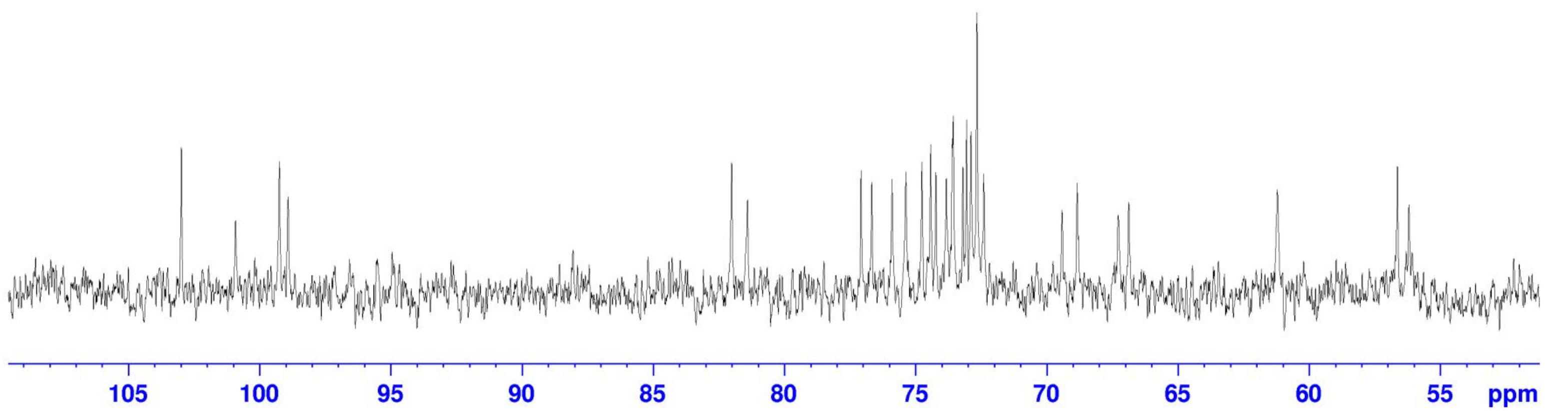

Compound 3 ^{19}F-NMR (376 MHz, CD_3OD)

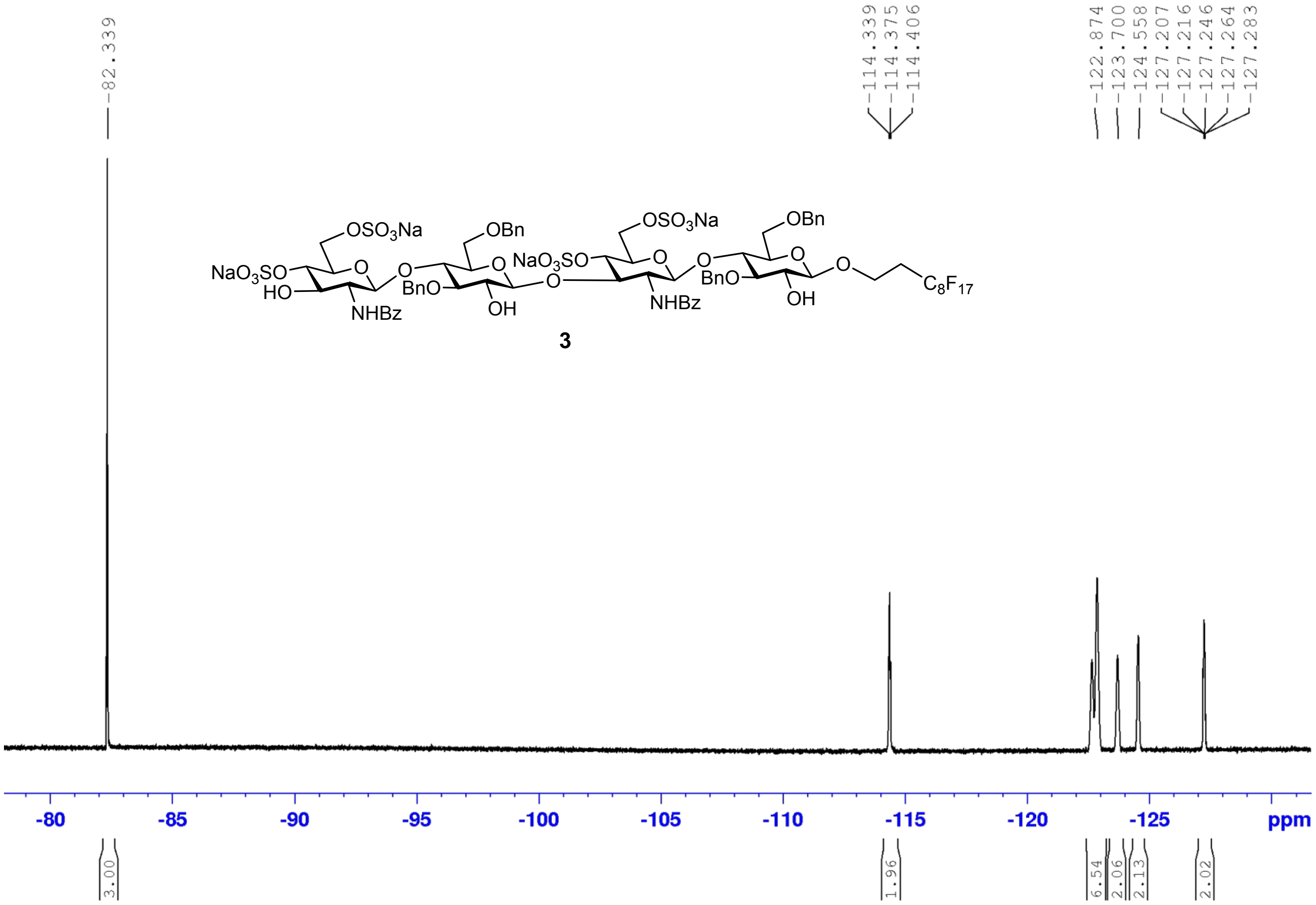

Compound 29 ^{1}H-NMR (400 MHz, CD_3OD)

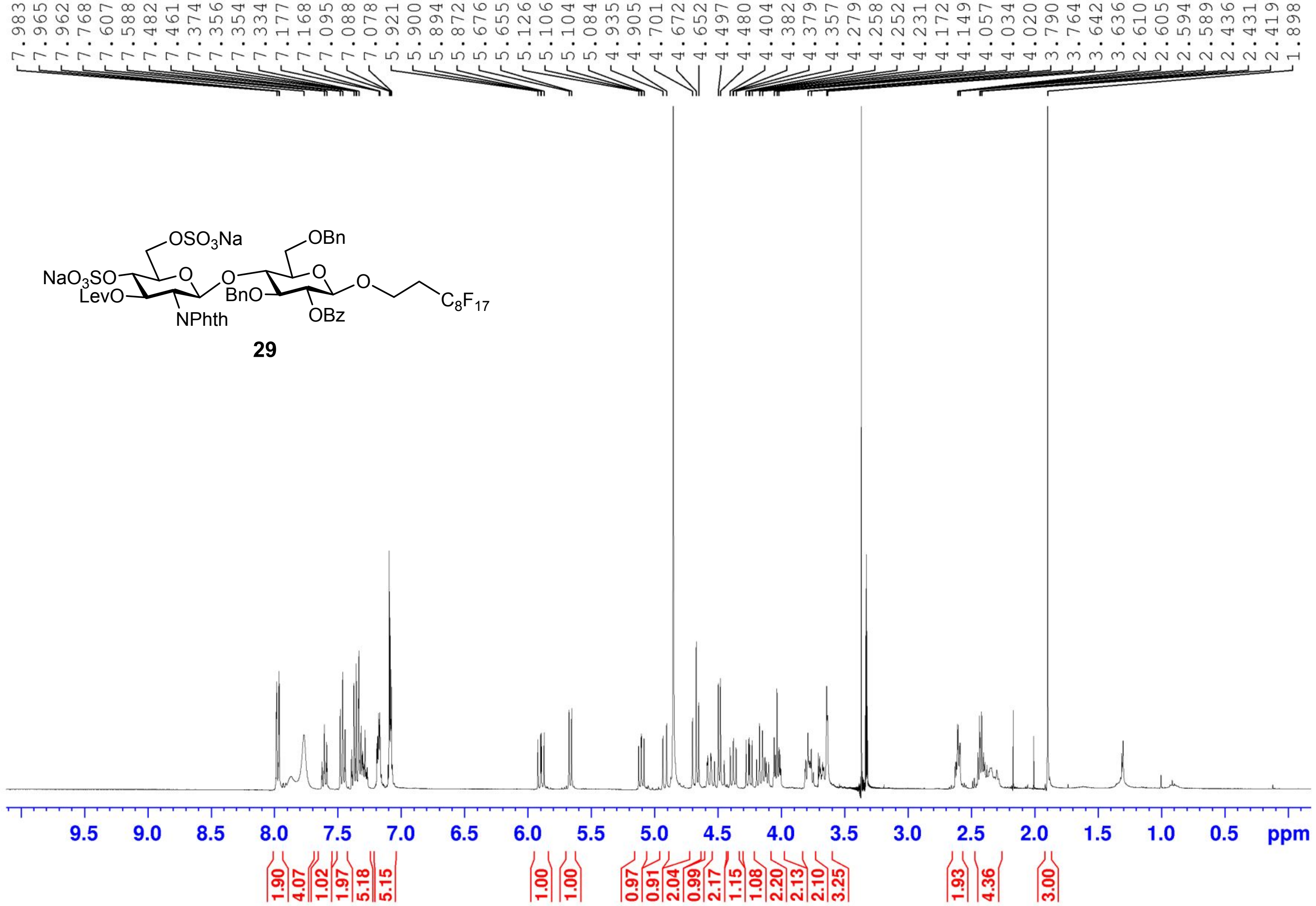

29

Compound 29 ^{1}H-NMR (400 MHz, CD_3OD)

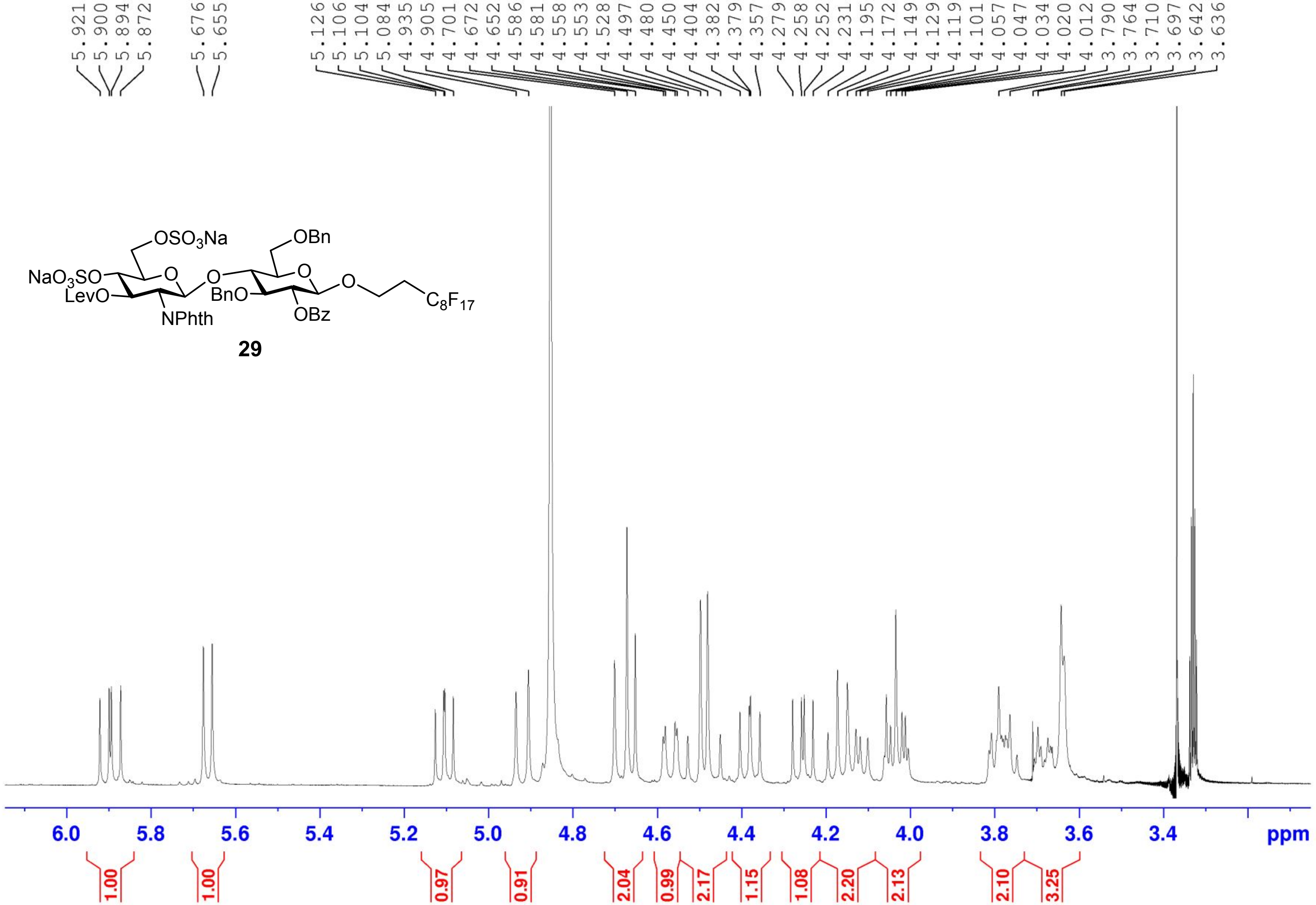

Compound 29 $^{13}C\{^{1}H\}$ NMR (100 MHz, CD_3OD)

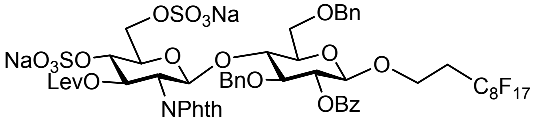

29

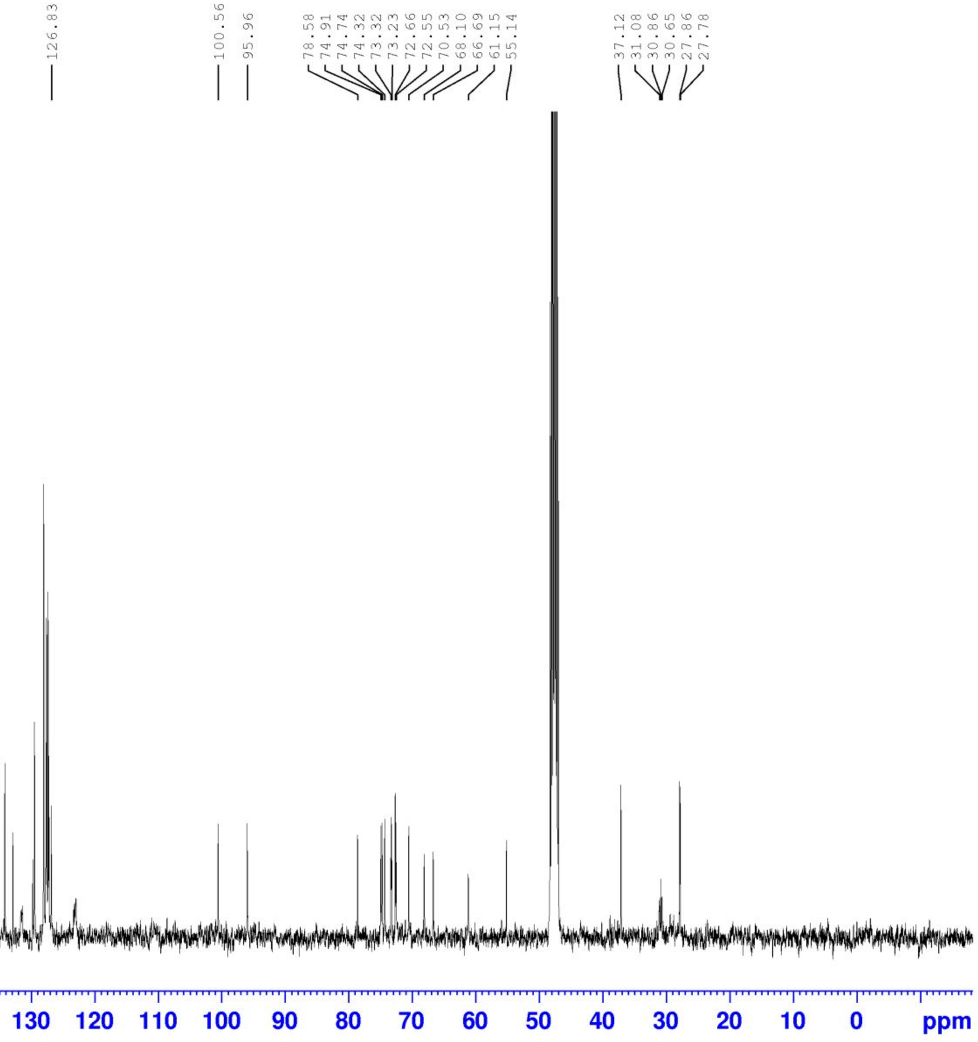

Compound 4 ^{1}H-NMR (400 MHz, CD_3OD)

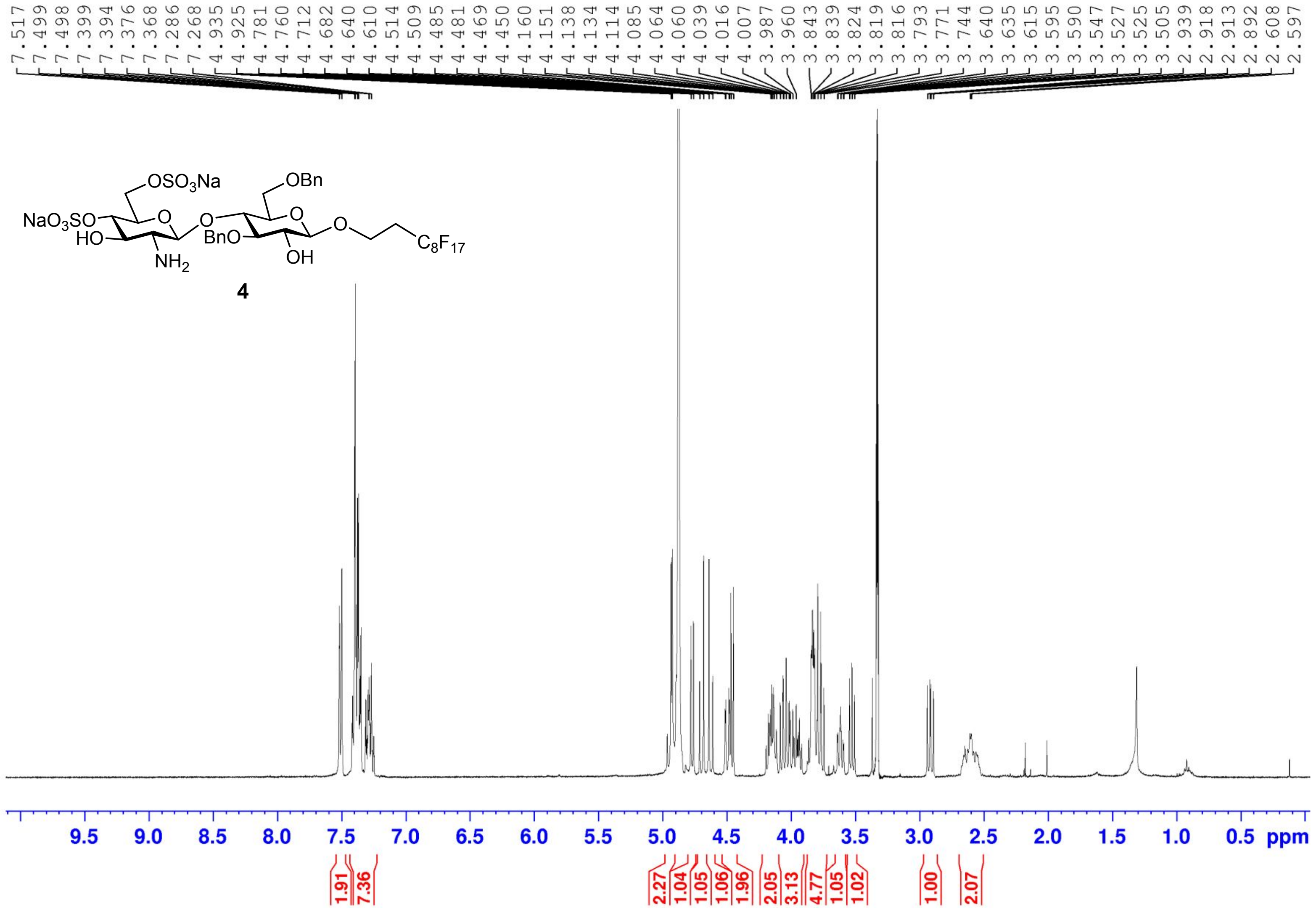

Compound 4 ^{1}H-NMR (400 MHz, CD_3OD)

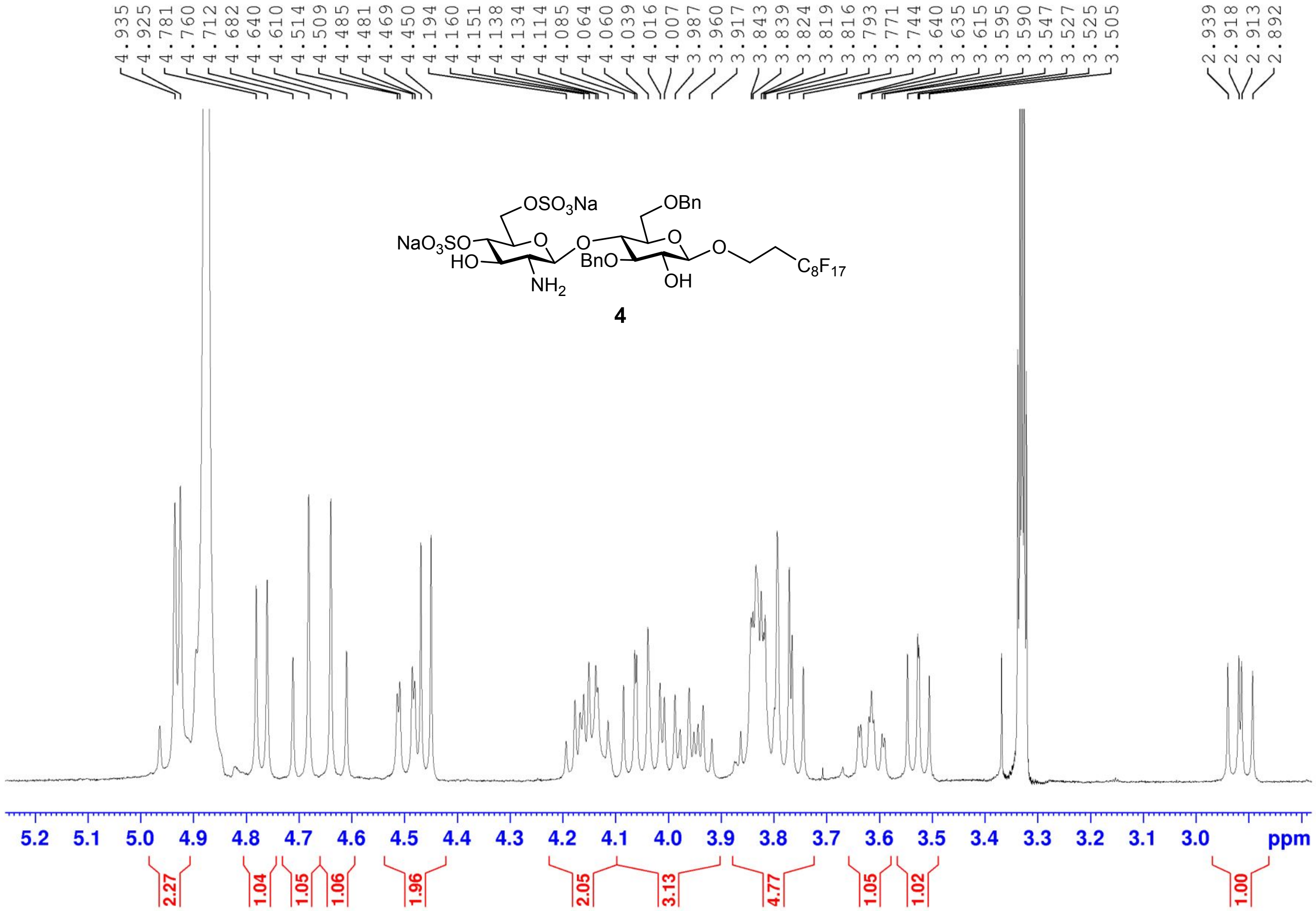

Compound 4 $^{13}C\{^1H\}$ NMR (100 MHz, CD_3OD)

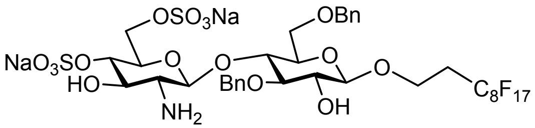

4

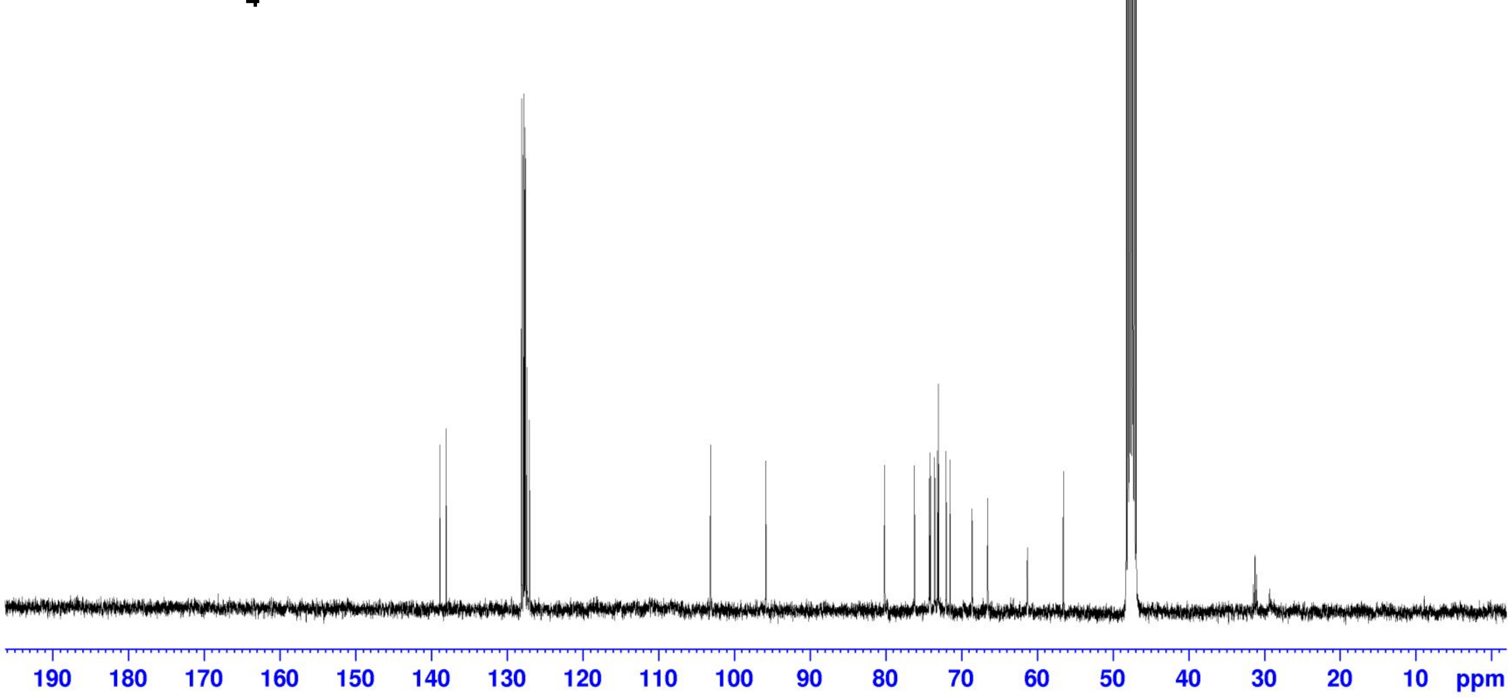

Compound 5 ^{1}H-NMR (400 MHz, CD_3OD)

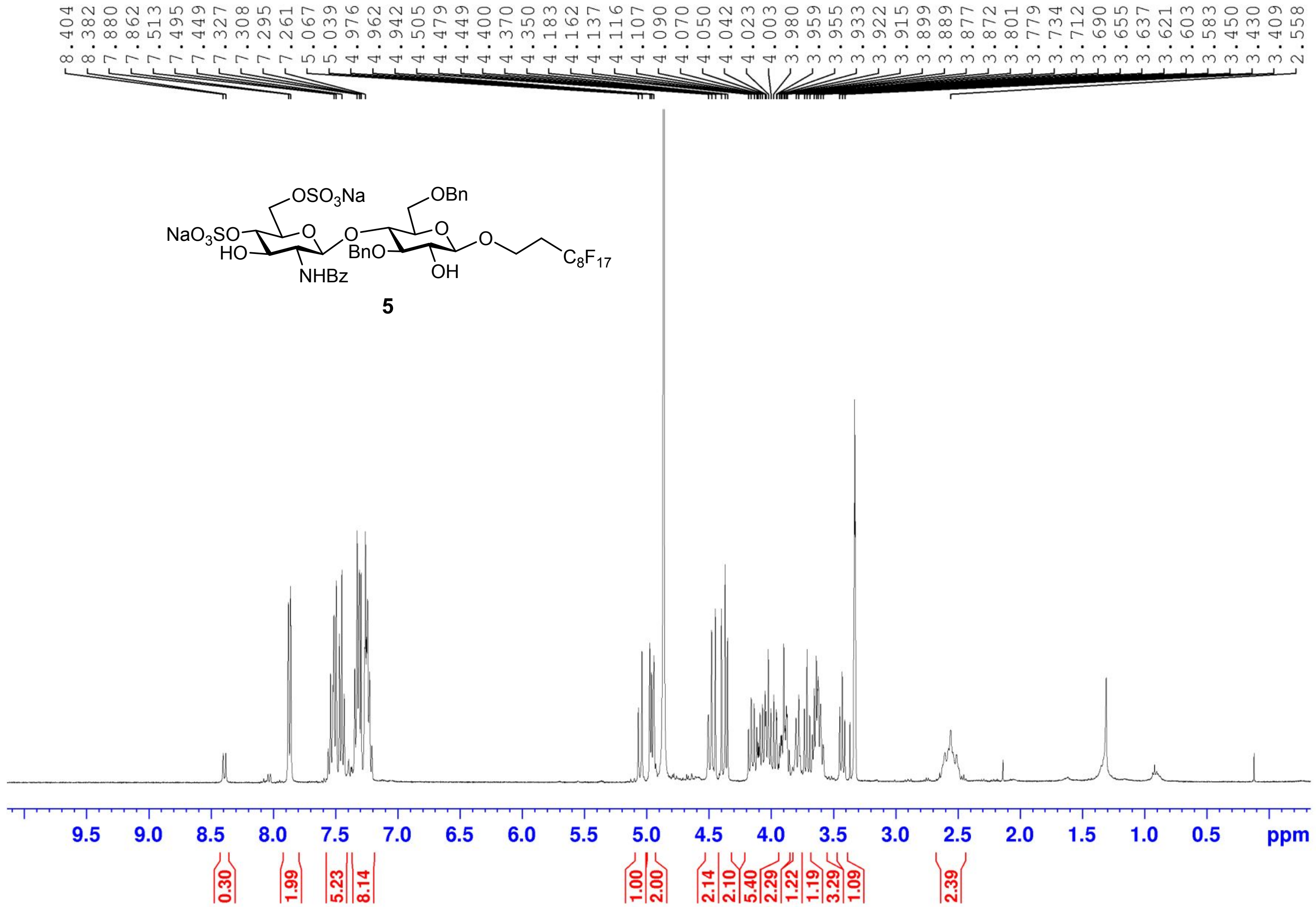

Compound 5 ^{1}H-NMR (400 MHz, CD_3OD)

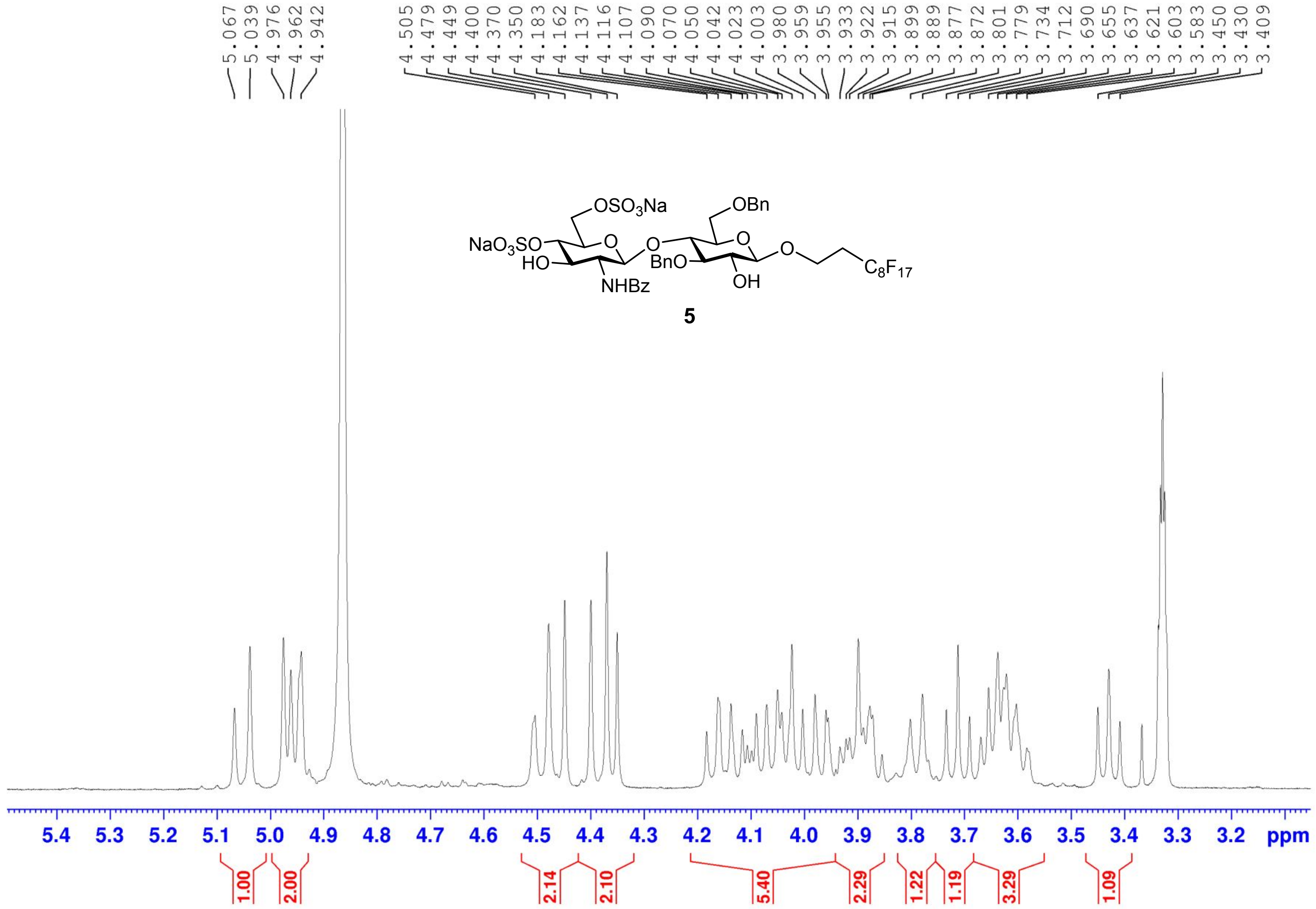

Compound 5 $^{13}C\{^1H\}$ NMR (100 MHz, CD_3OD)

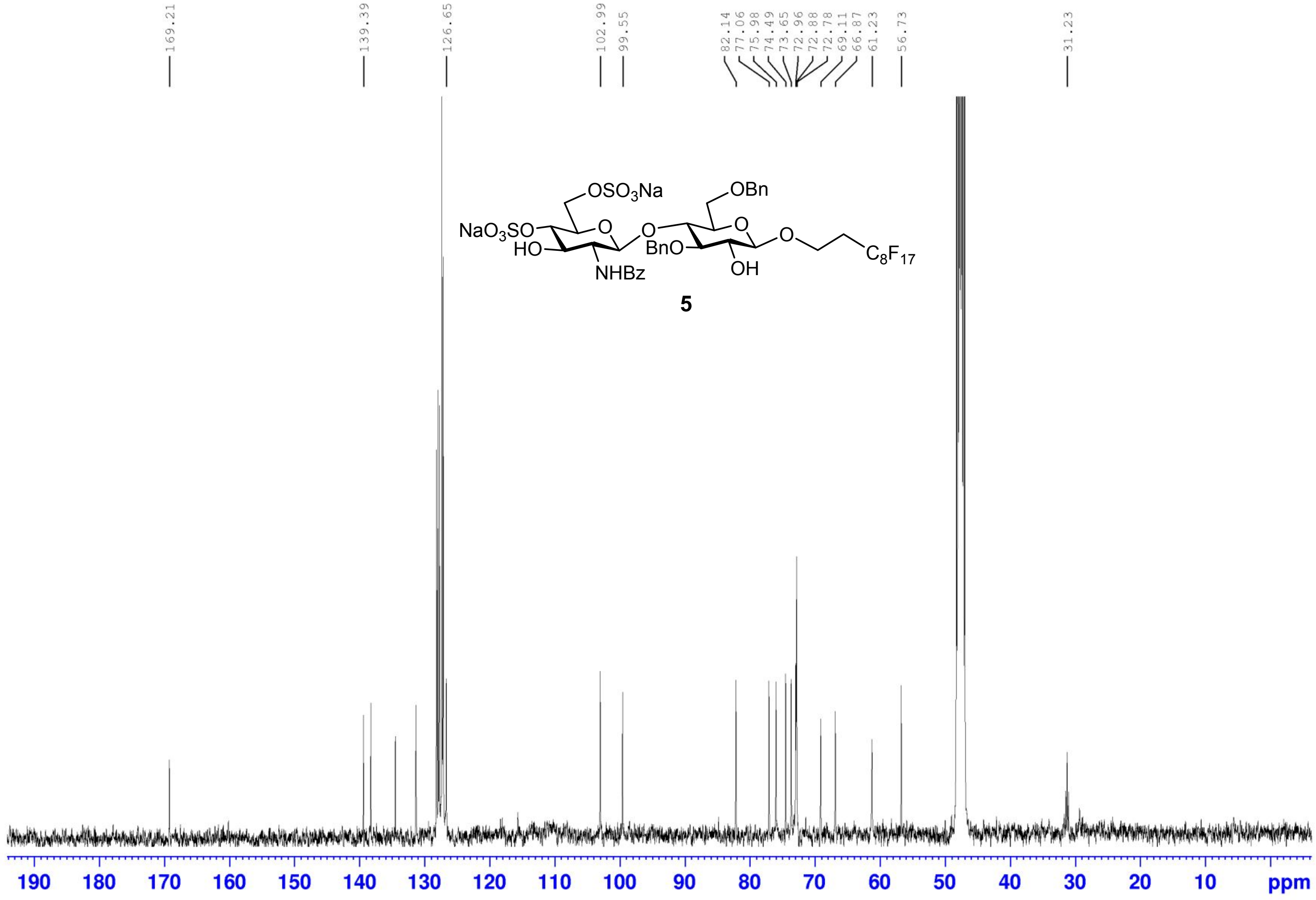

Compound 6 ^{1}H-NMR (400 MHz, CD_3OD)

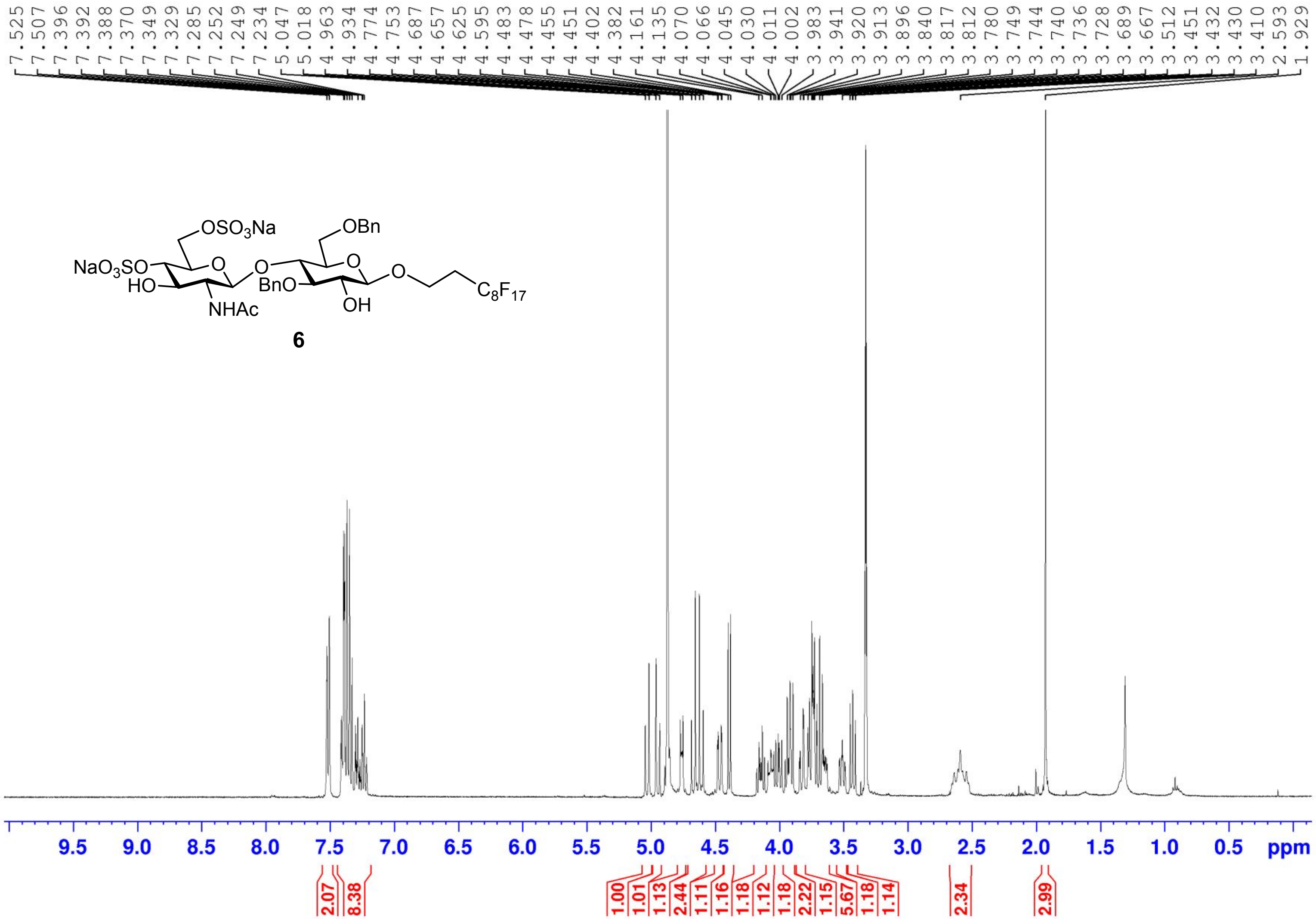

Compound 6 ^{1}H-NMR (400 MHz, CD_3OD)

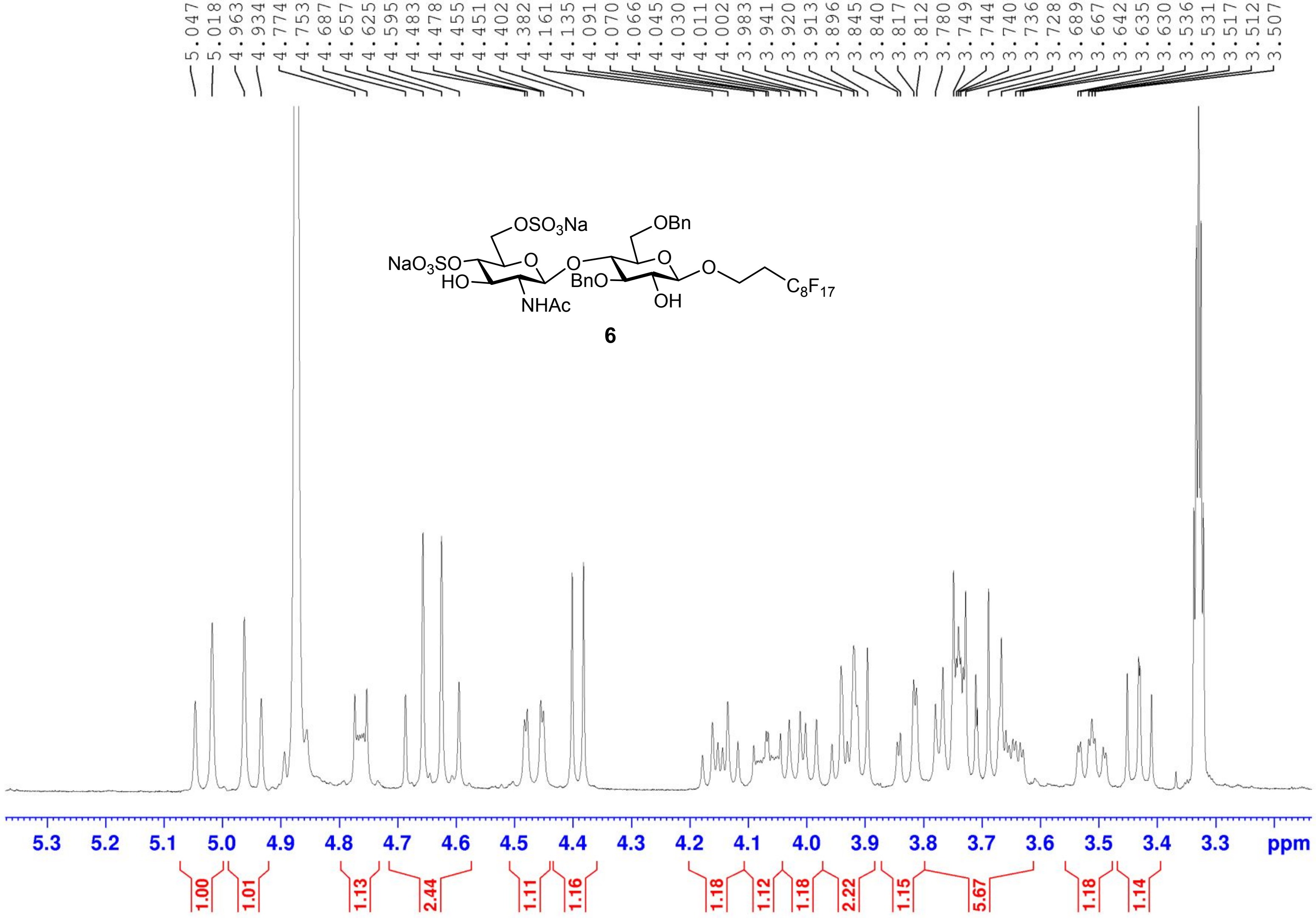

Compound 6 $^{13}C\{^{1}H\}$ NMR (100 MHz, CD_3OD)

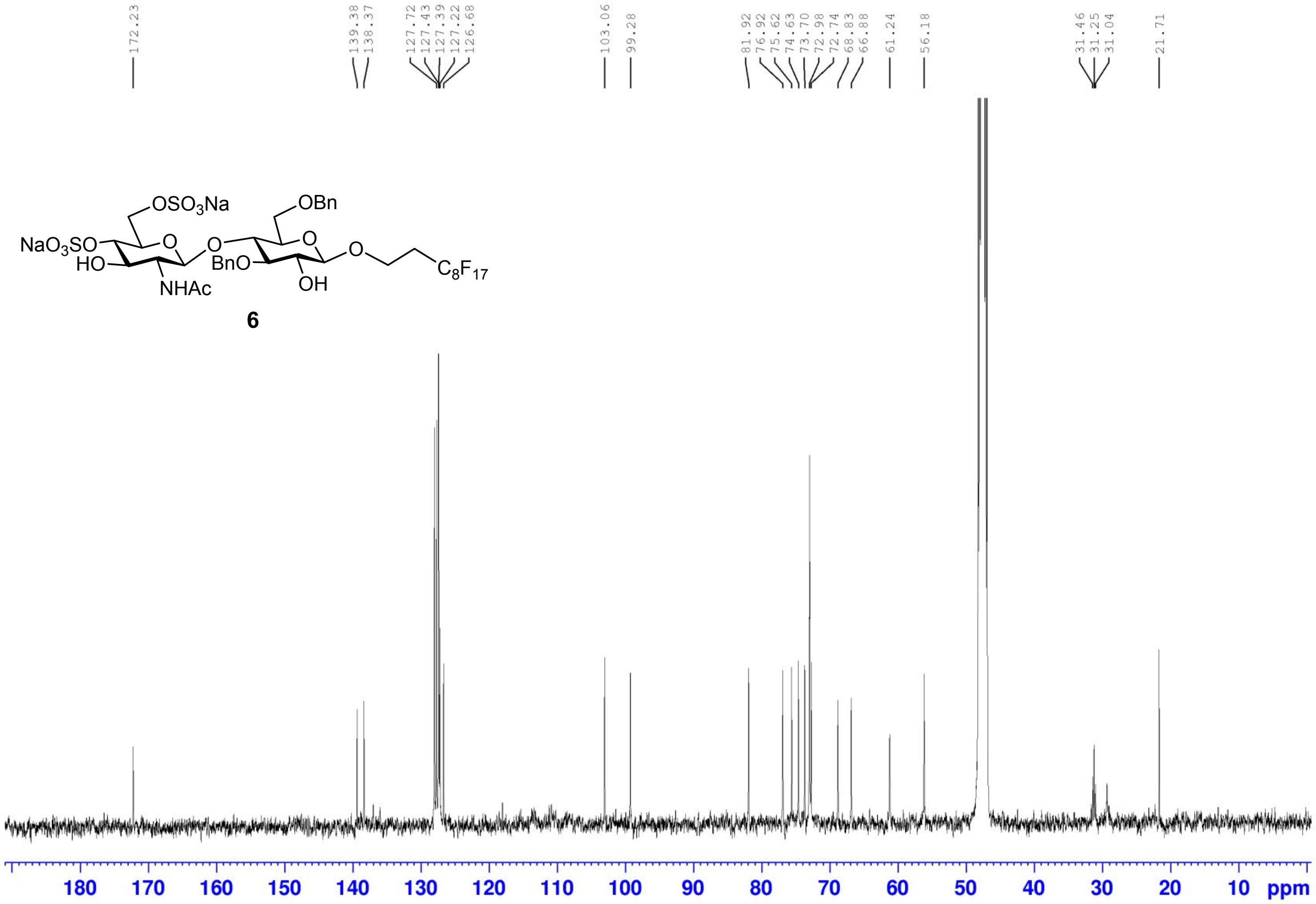

Compound 7 ^{1}H-NMR (400 MHz, CD_3OD)

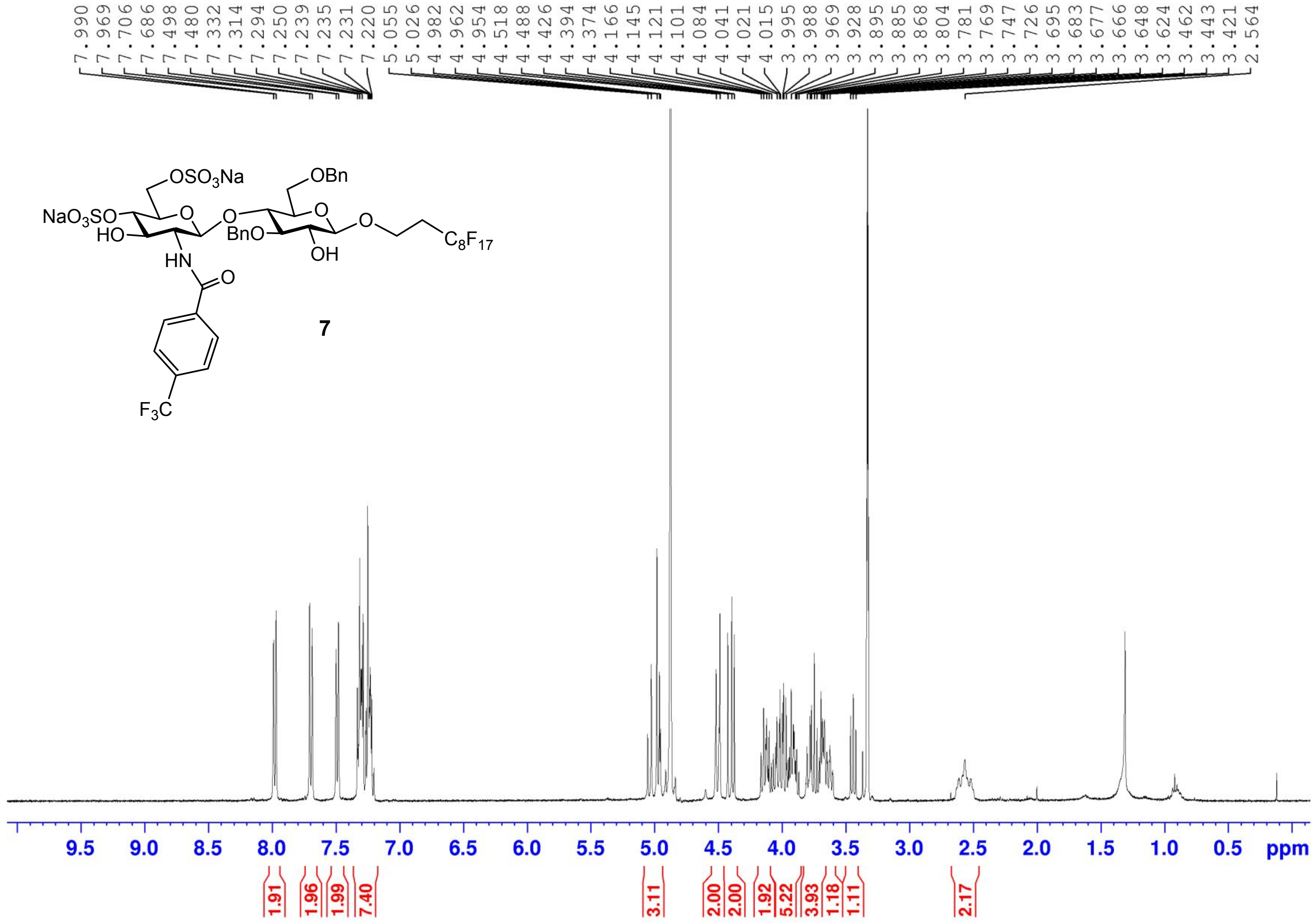

7

Compound 7 ^{1}H-NMR (400 MHz, CD_3OD)

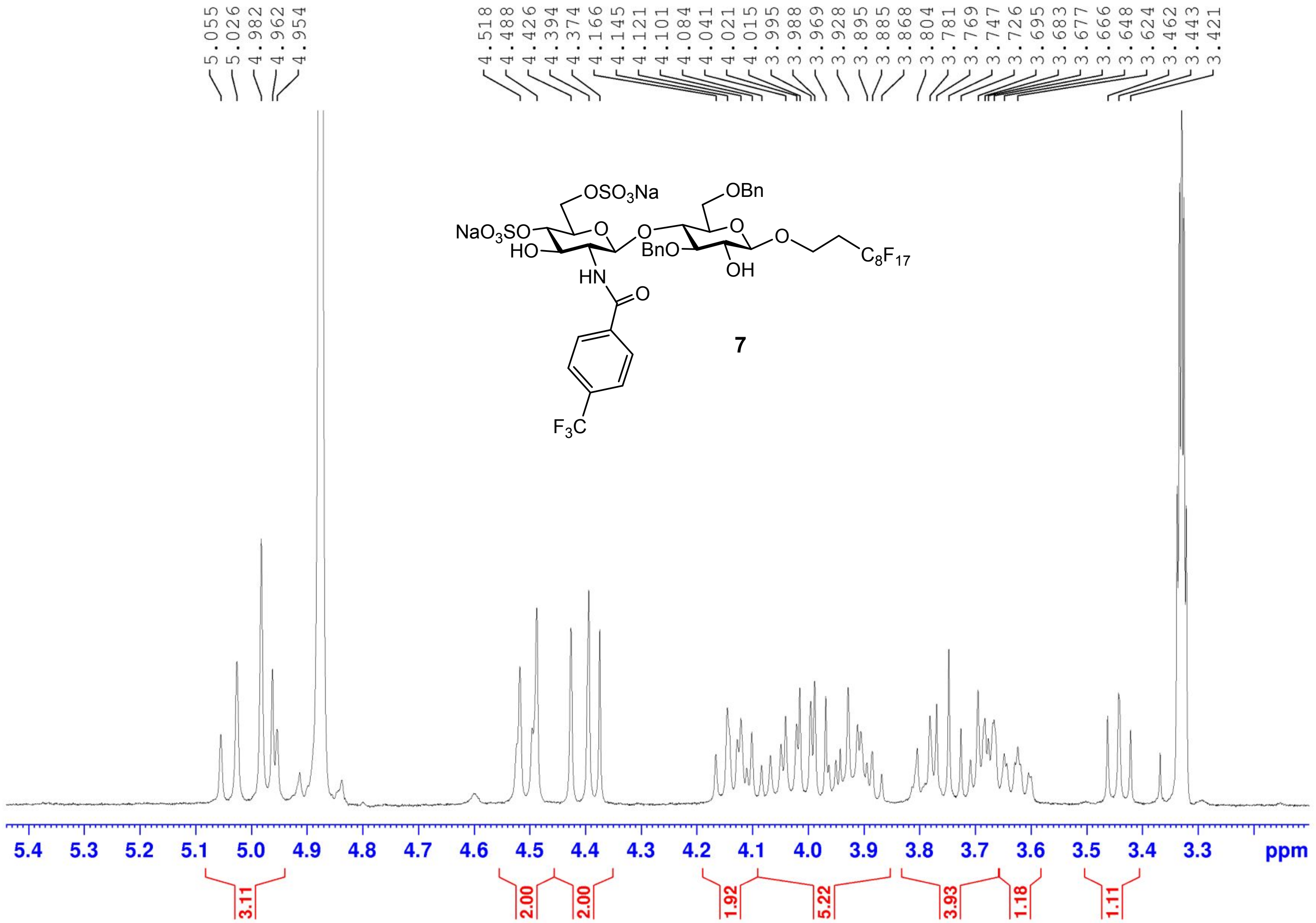

Compound 7 $^{13}C\{^{1}H\}$ NMR (100 MHz, CD_3OD)

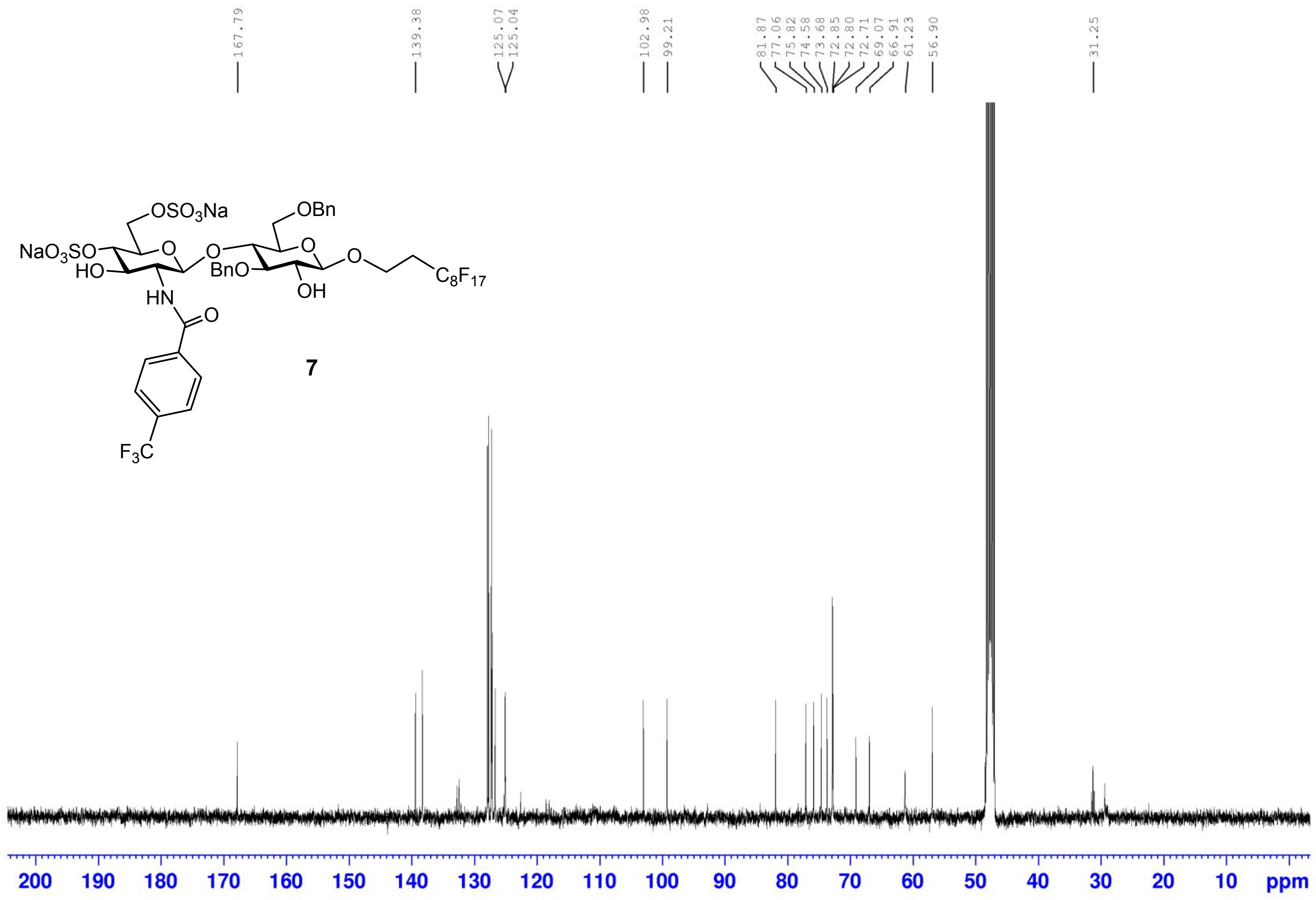

Compound 8 ^{1}H-NMR (400 MHz, CD_3OD)

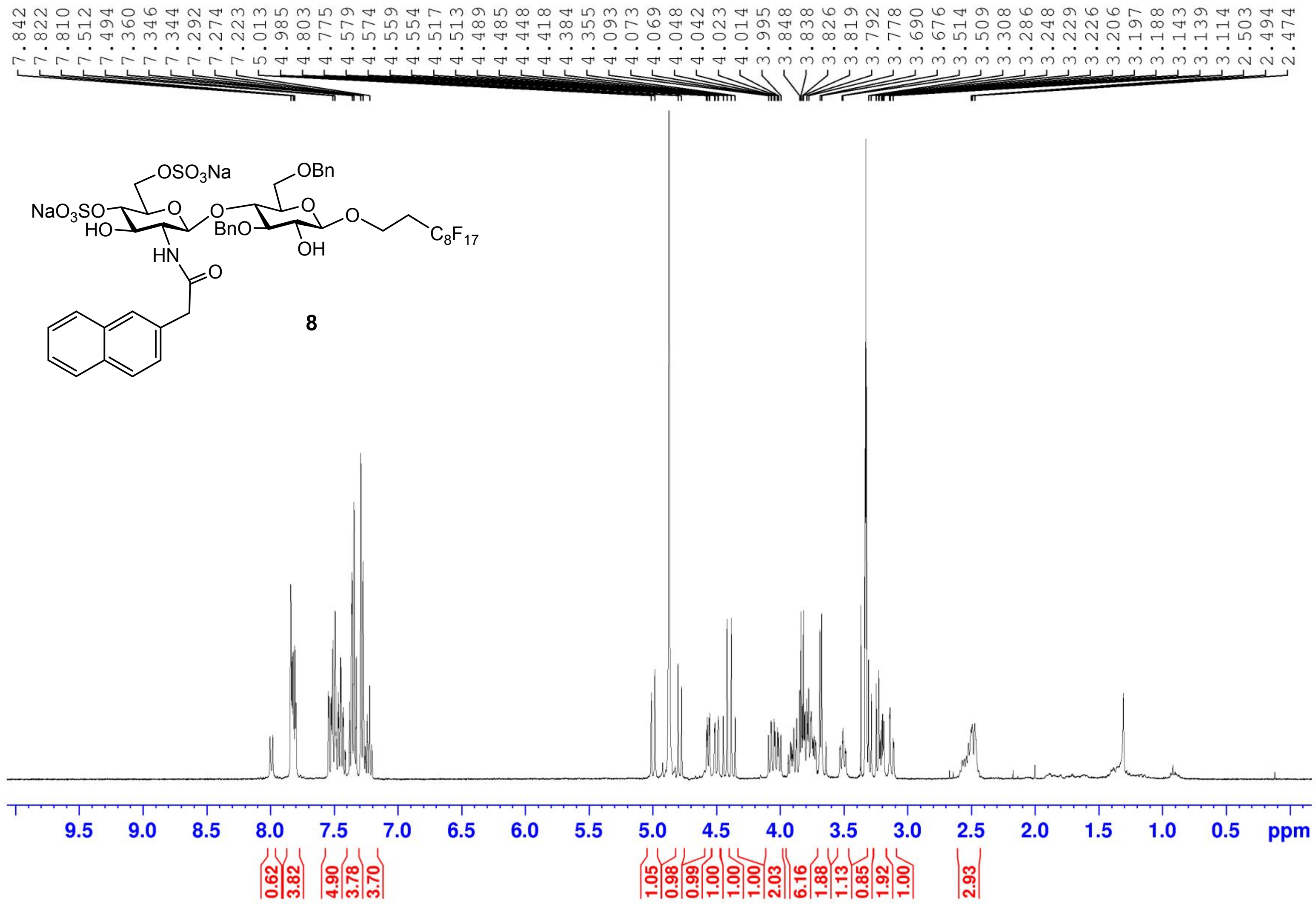

Compound 8 ^{1}H-NMR (400 MHz, CD_3OD)

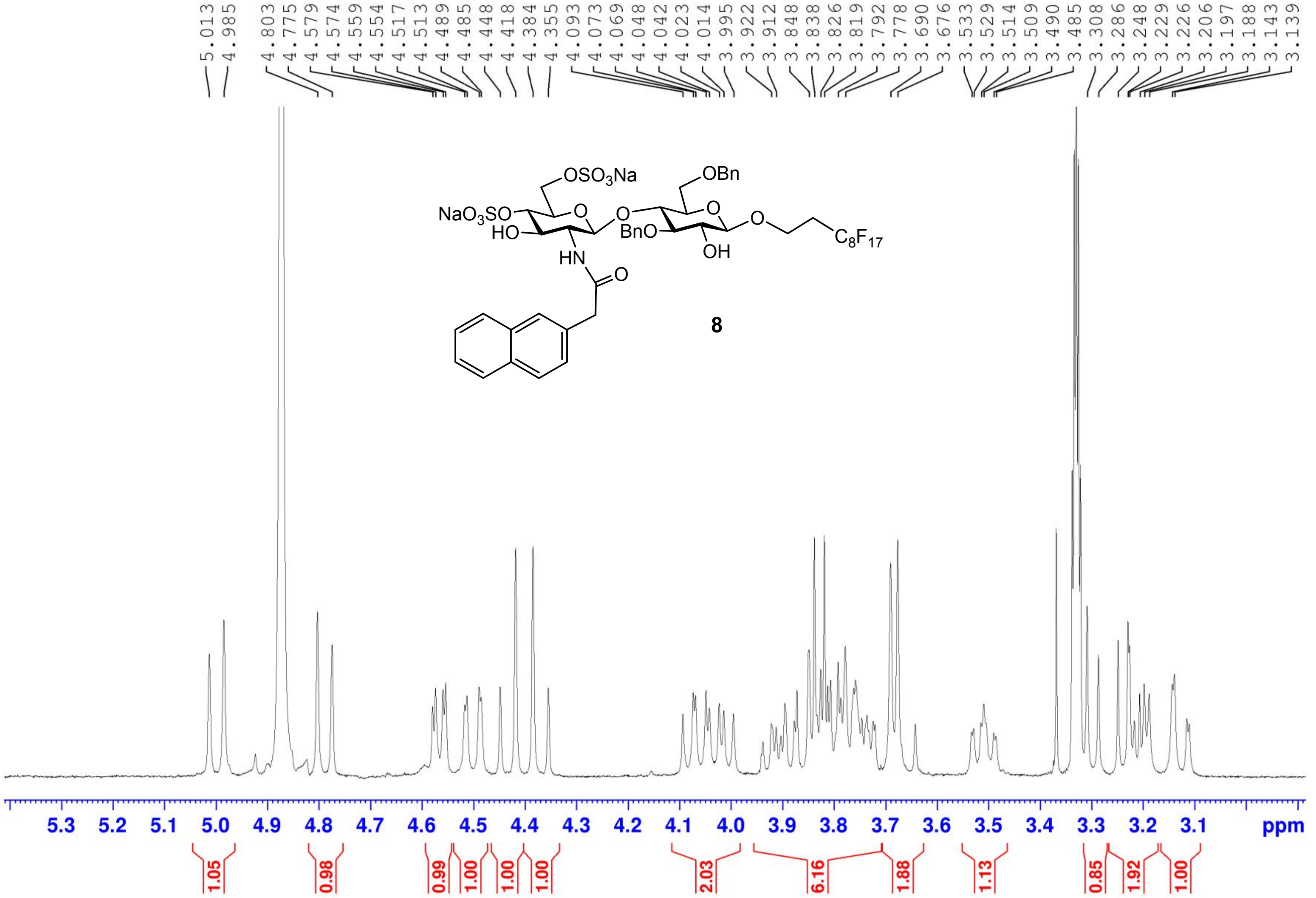

Compound 8 $^{13}C\{^{1}H\}$ NMR (100 MHz, CD_3OD)

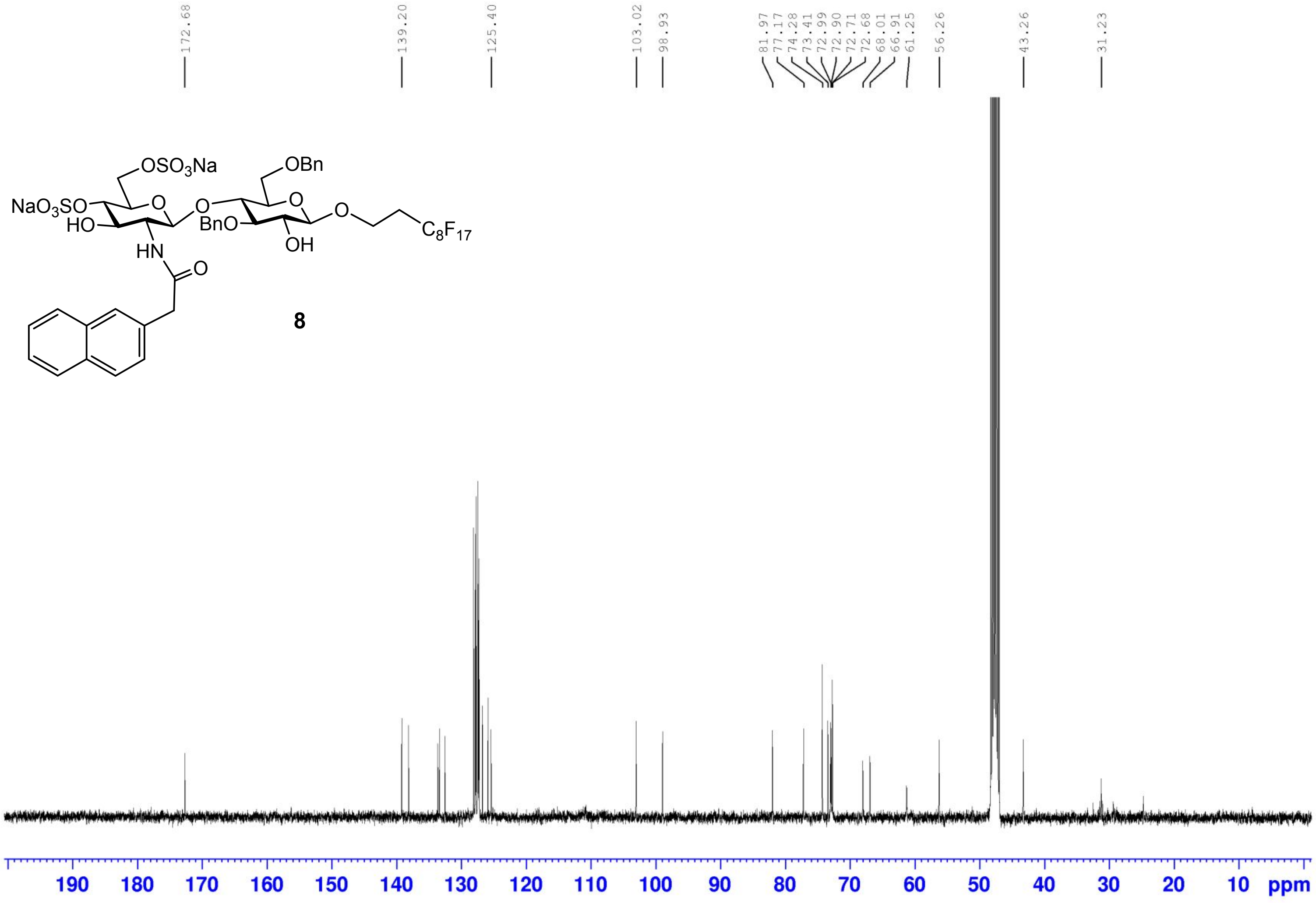

Compound 9 ^{1}H-NMR (400 MHz, CD_3OD)

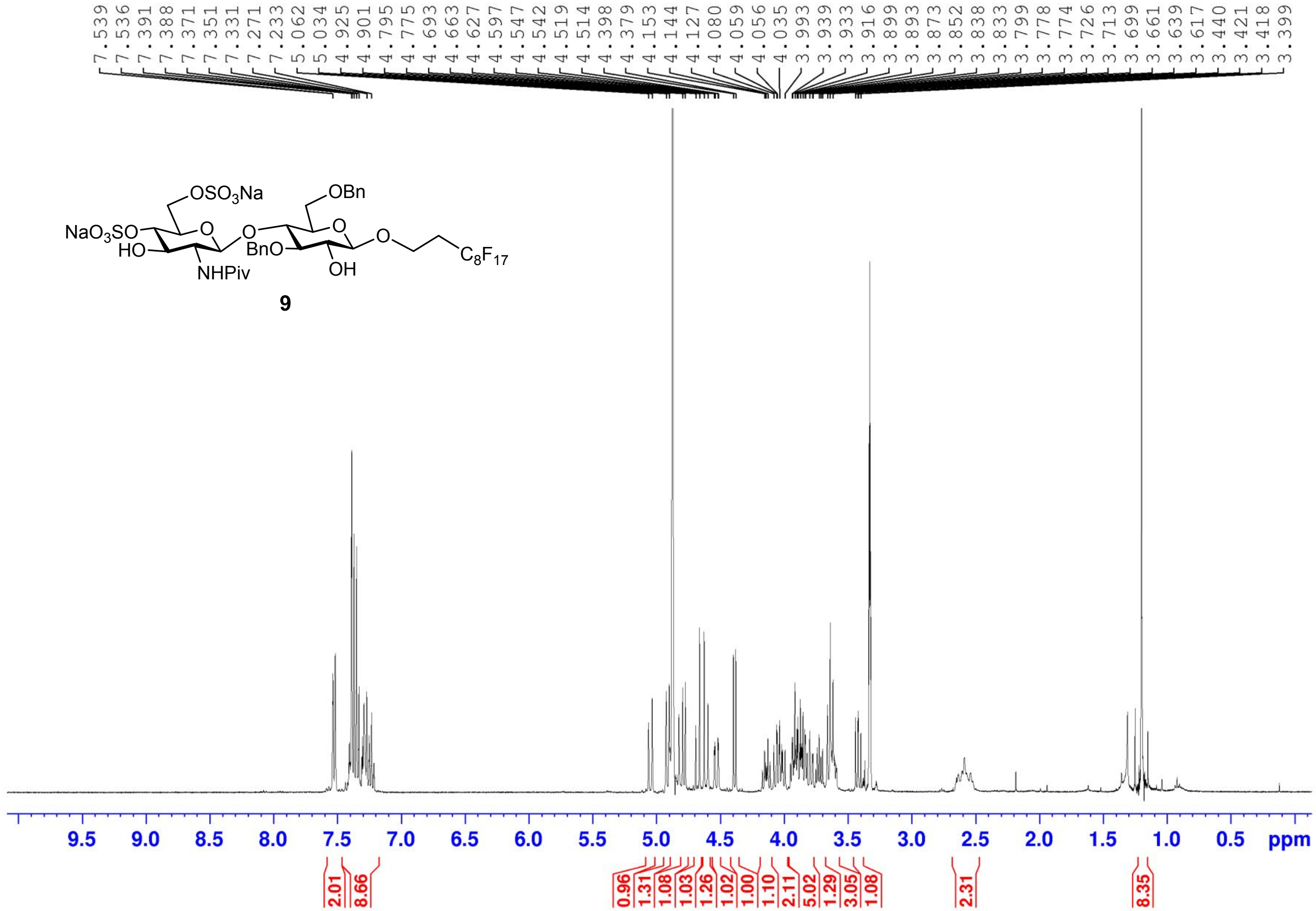

Compound 9 ^{1}H-NMR (400 MHz, CD_3OD)

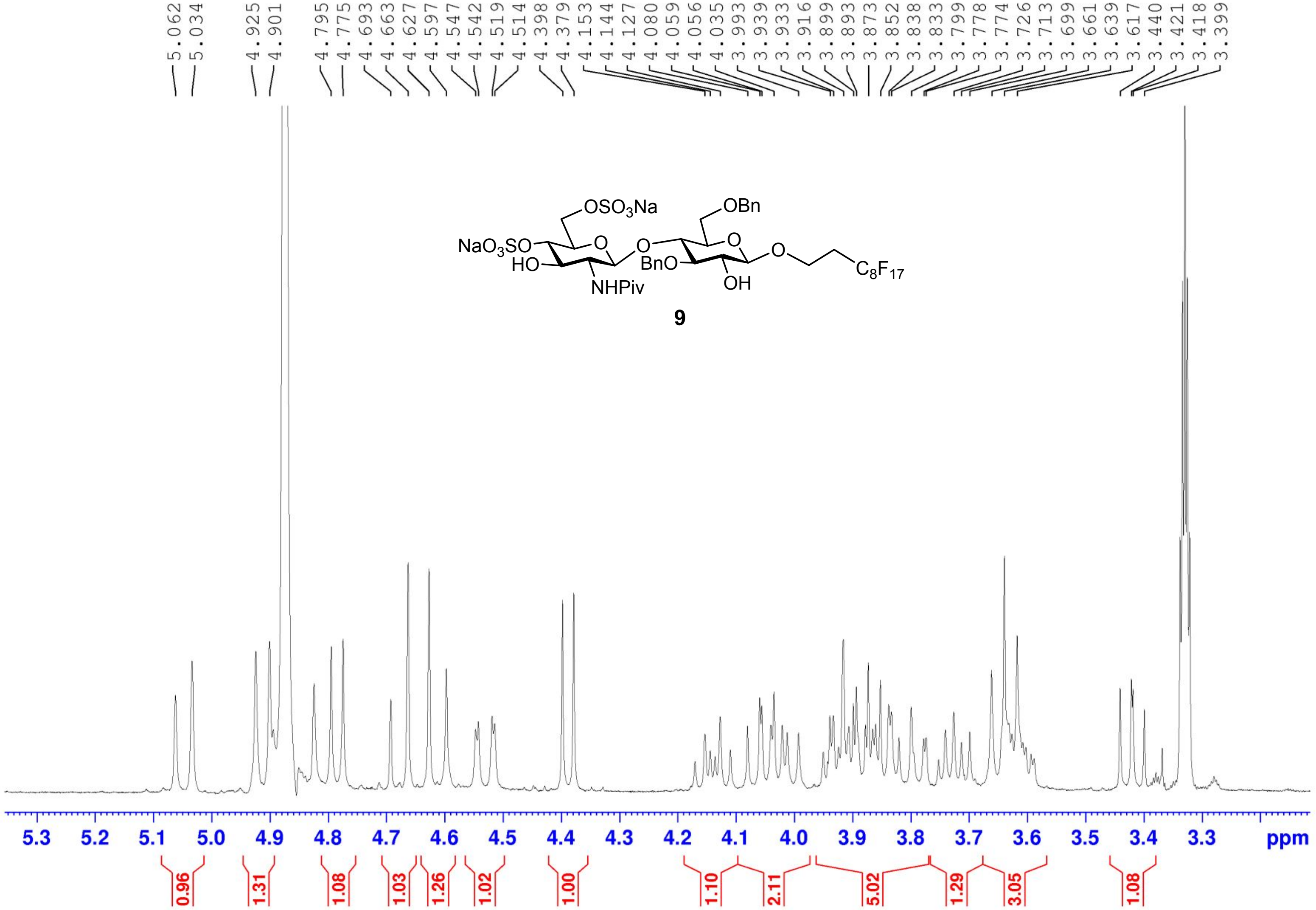

Compound 9 $^{13}C\{^{1}H\}$ NMR (100 MHz, CD_3OD)

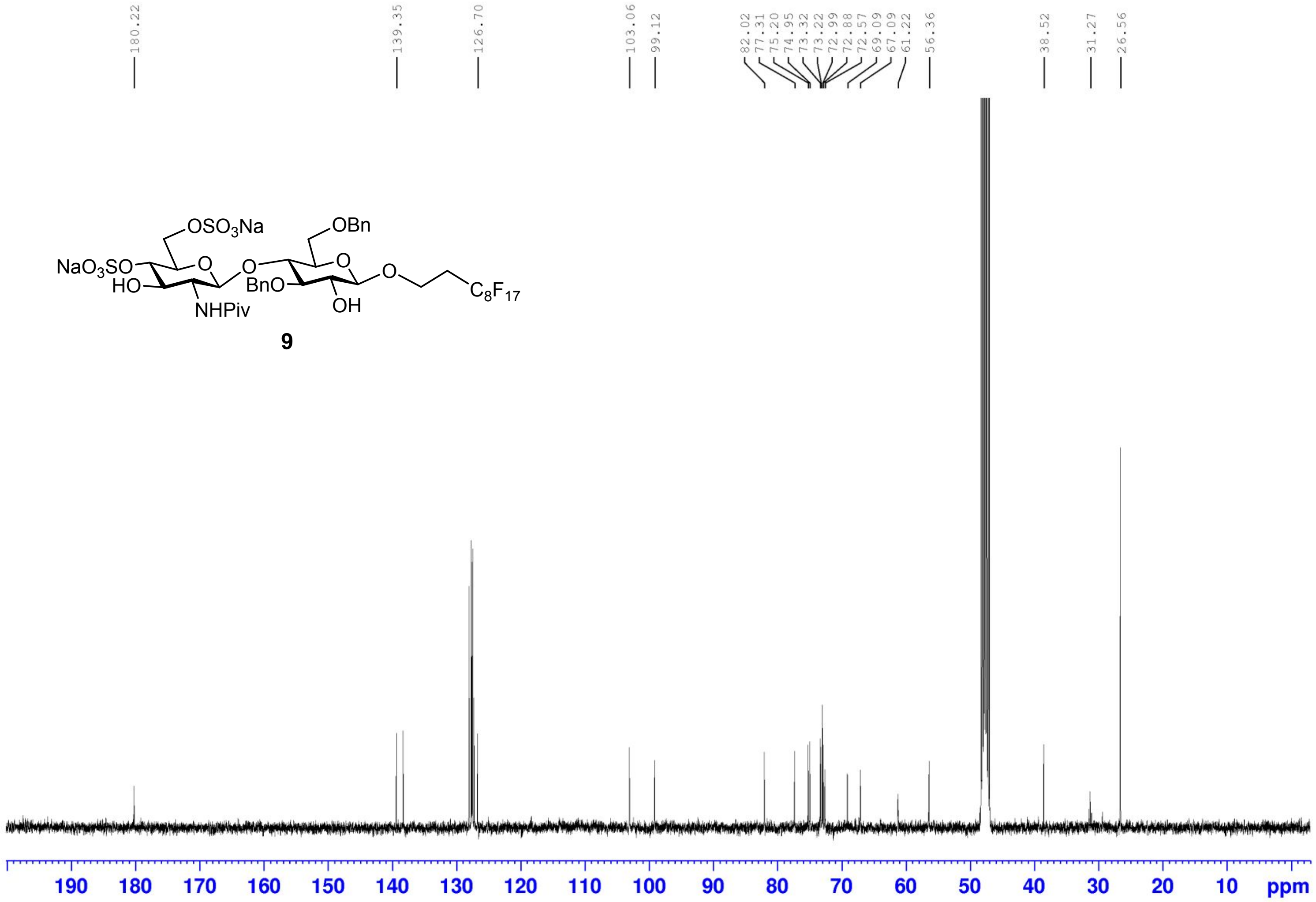

Compound 30 ^{1}H-NMR (400 MHz, CD_3OD)

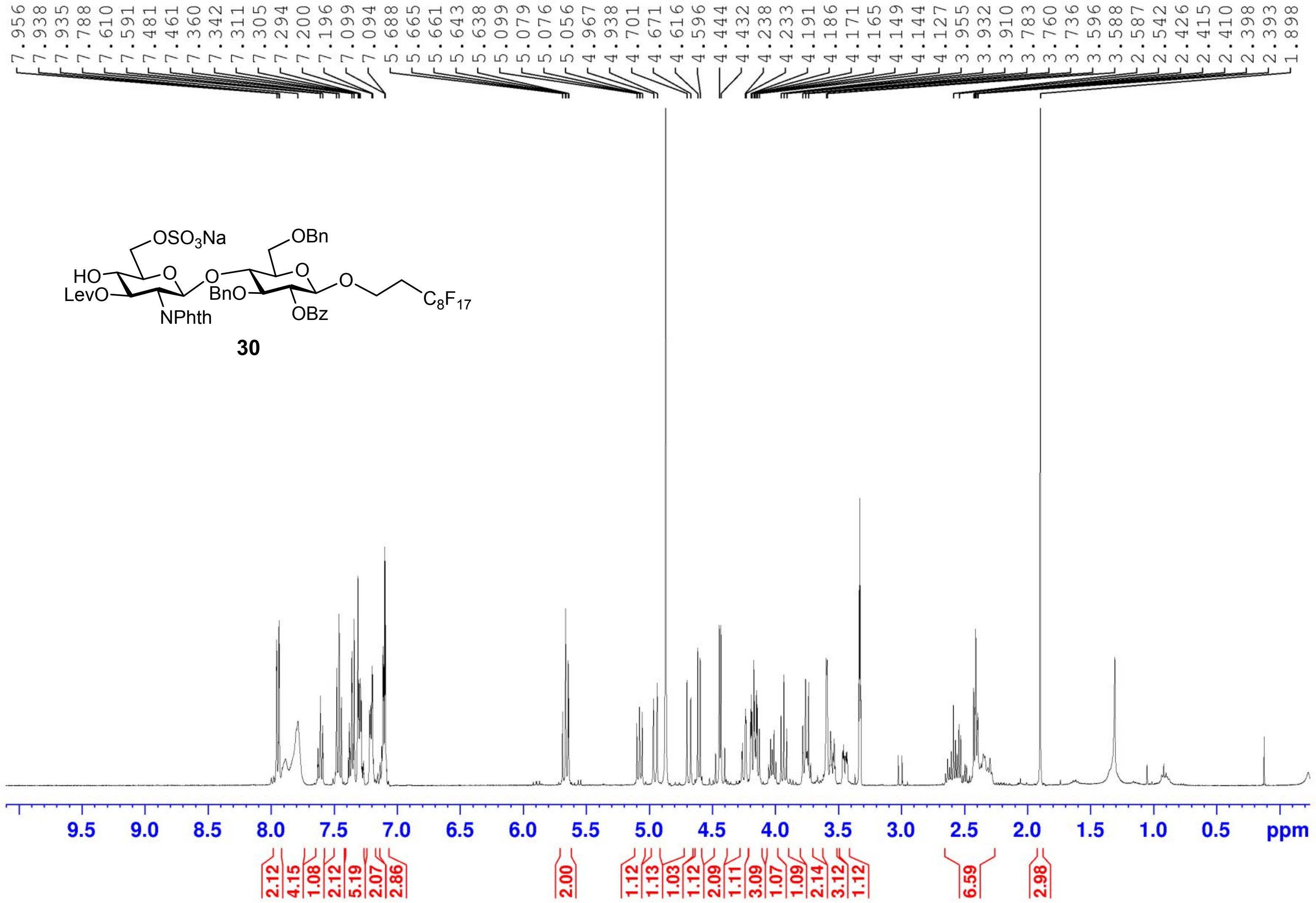

Compound 30 ^{1}H-NMR (400 MHz, CD_3OD)

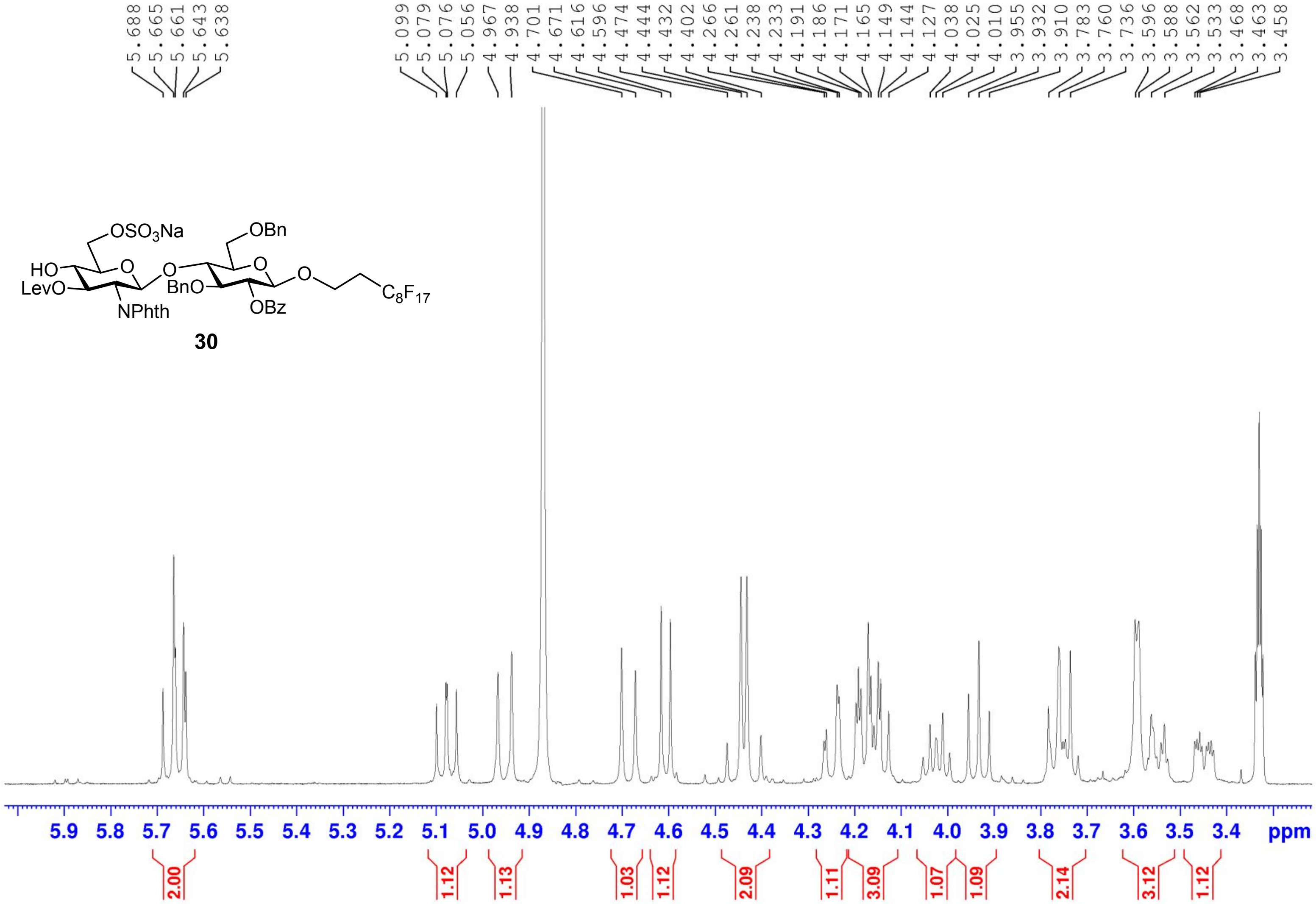

Compound 30 $^{13}C\{^{1}H\}$ NMR (100 MHz, CD_3OD)

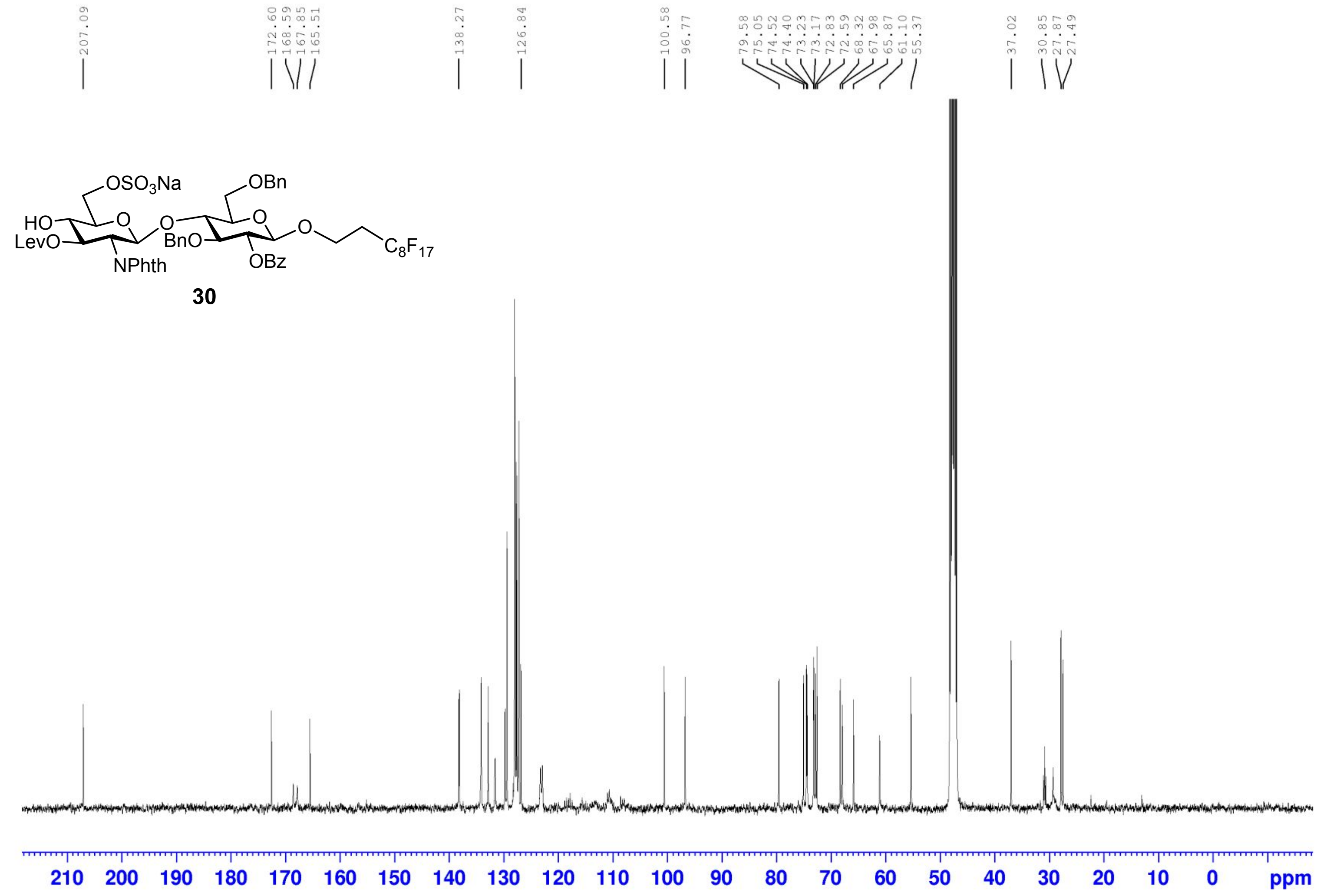

Compound 10 ^{1}H-NMR (400 MHz, CD_3OD)

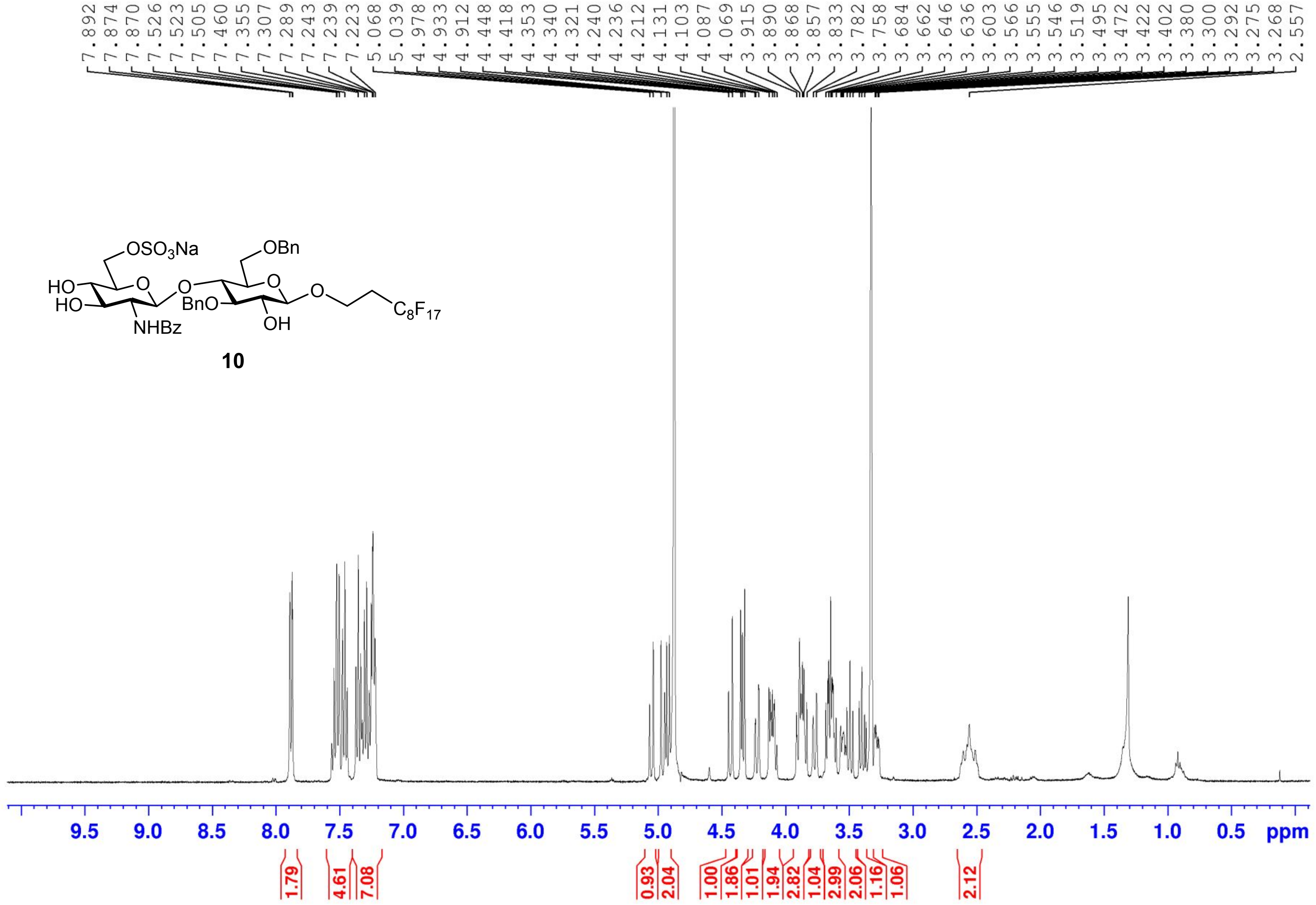

Compound 10 ^{1}H-NMR (400 MHz, CD_3OD)

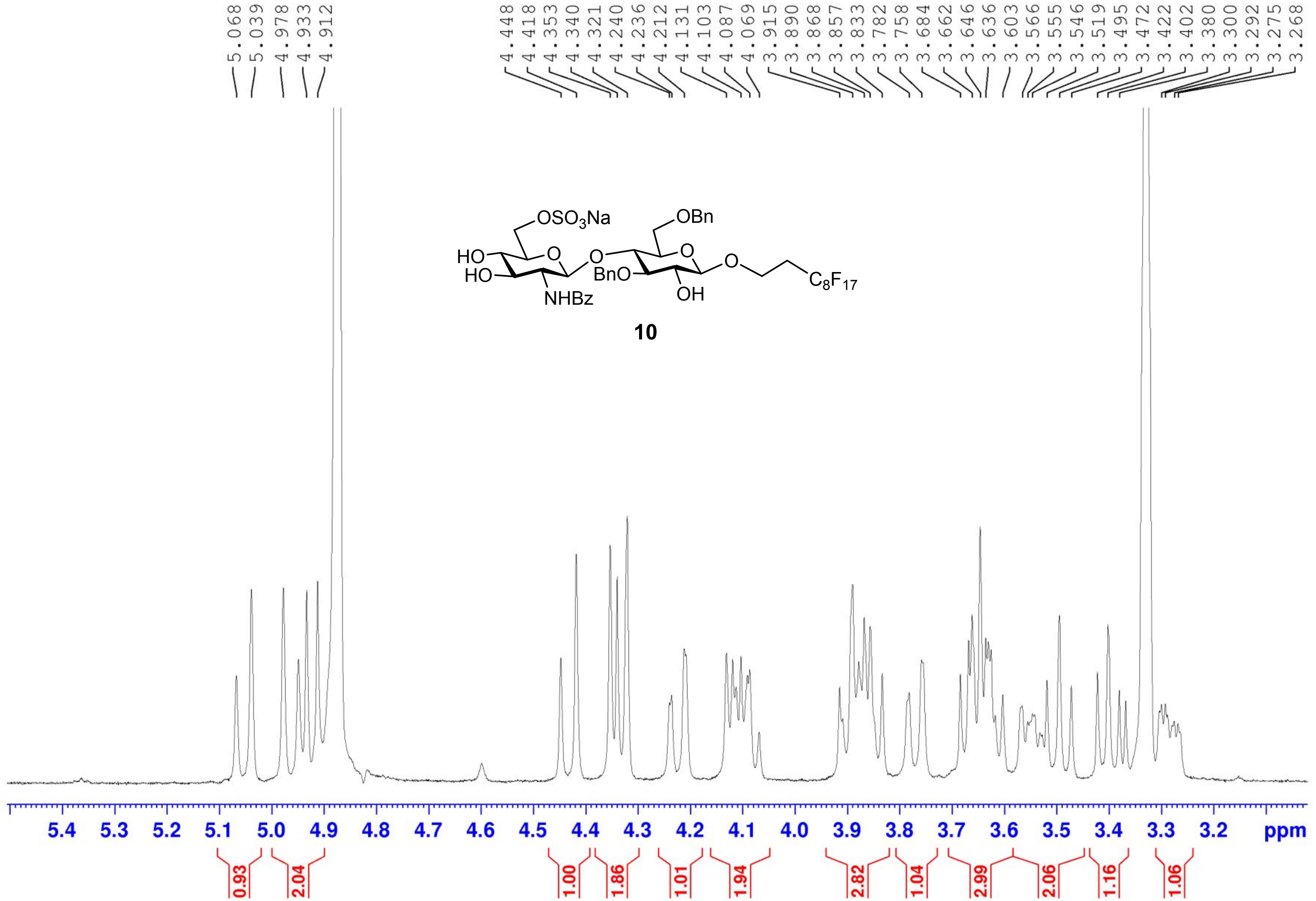

Compound 10 $^{13}C\{^{1}H\}$ NMR (100 MHz, CD_3OD)

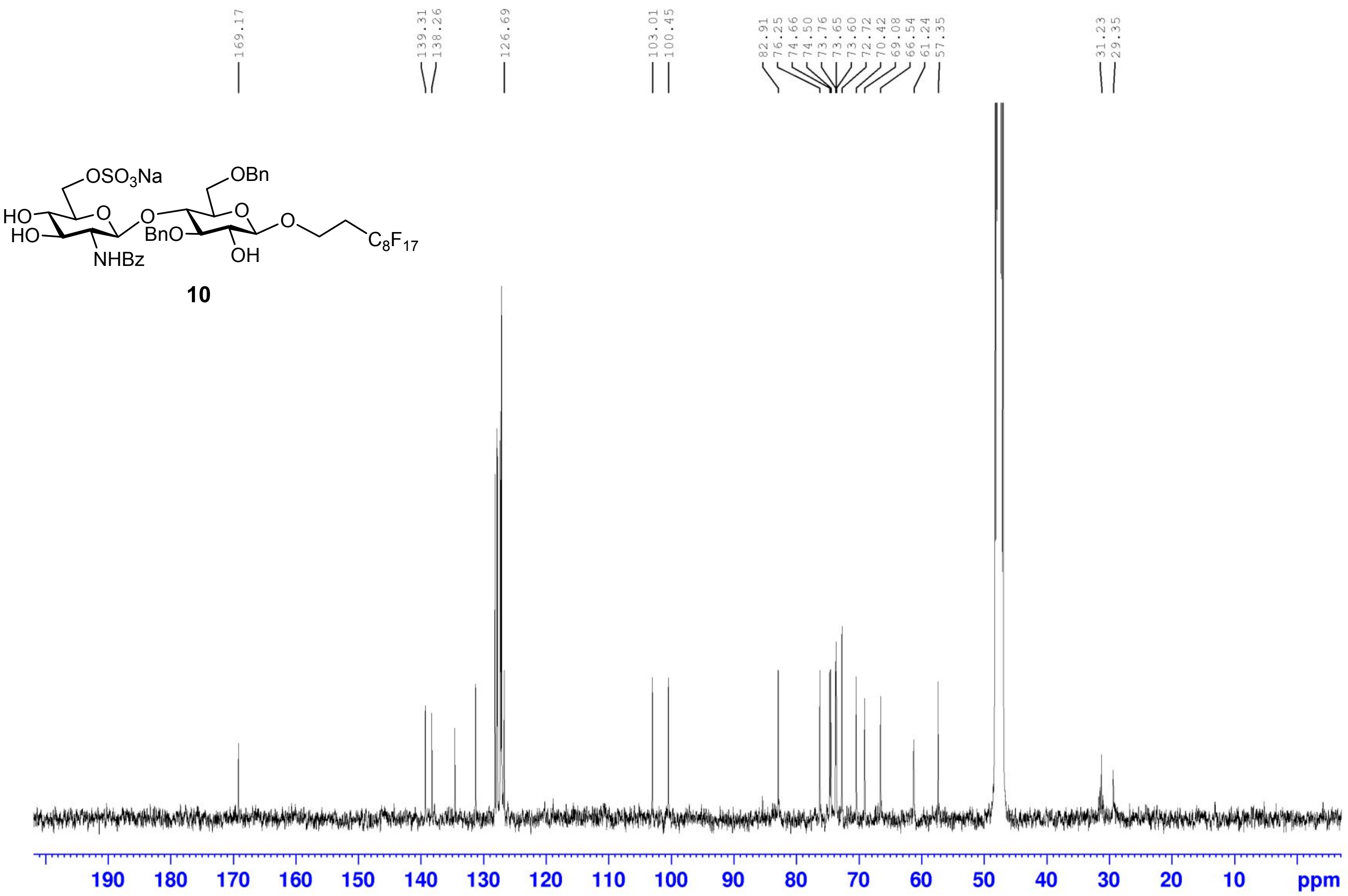

Compound 11 ^{1}H-NMR (400 MHz, CD_3OD)

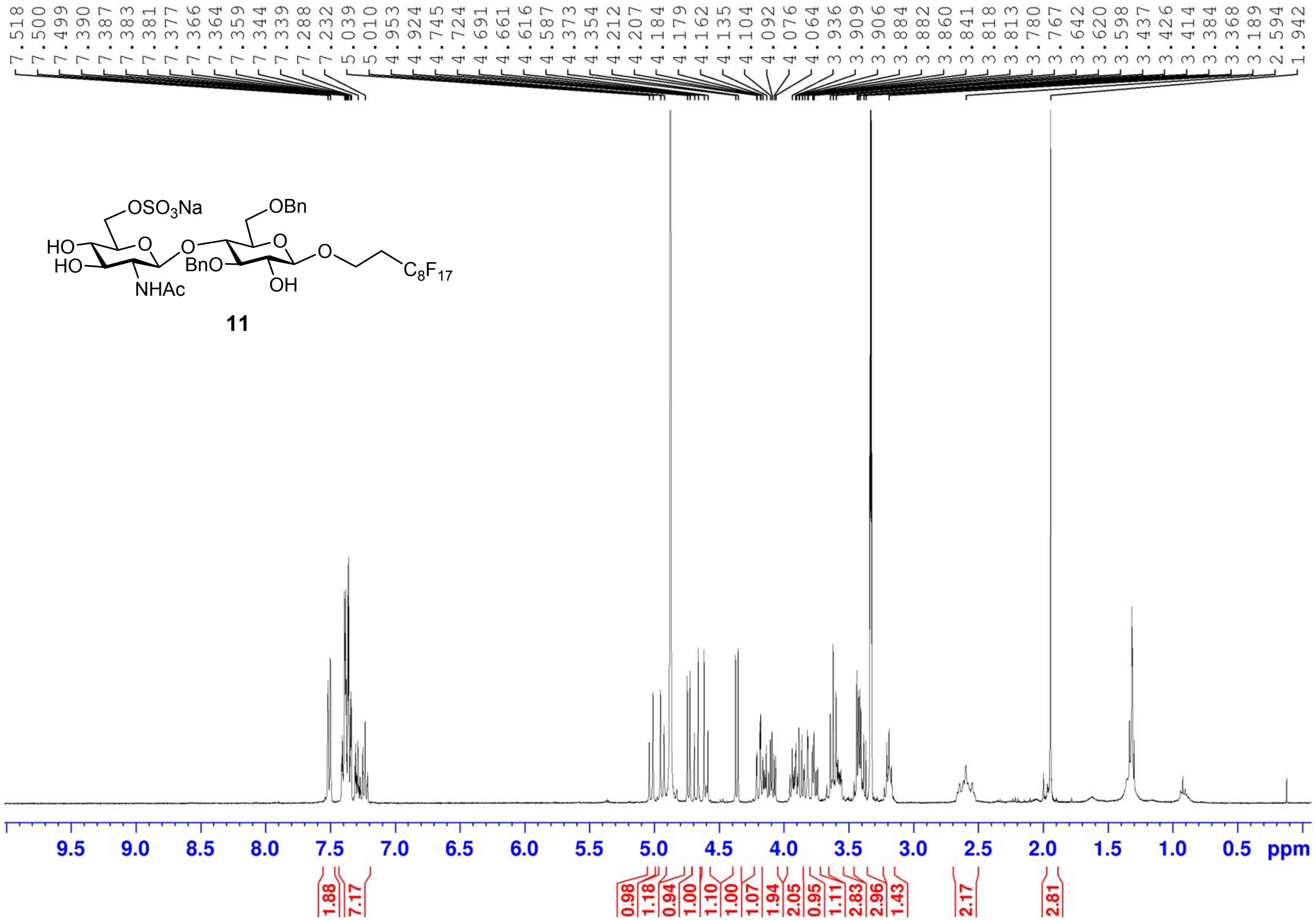

Compound 11 ^{1}H-NMR (400 MHz, CD_3OD)

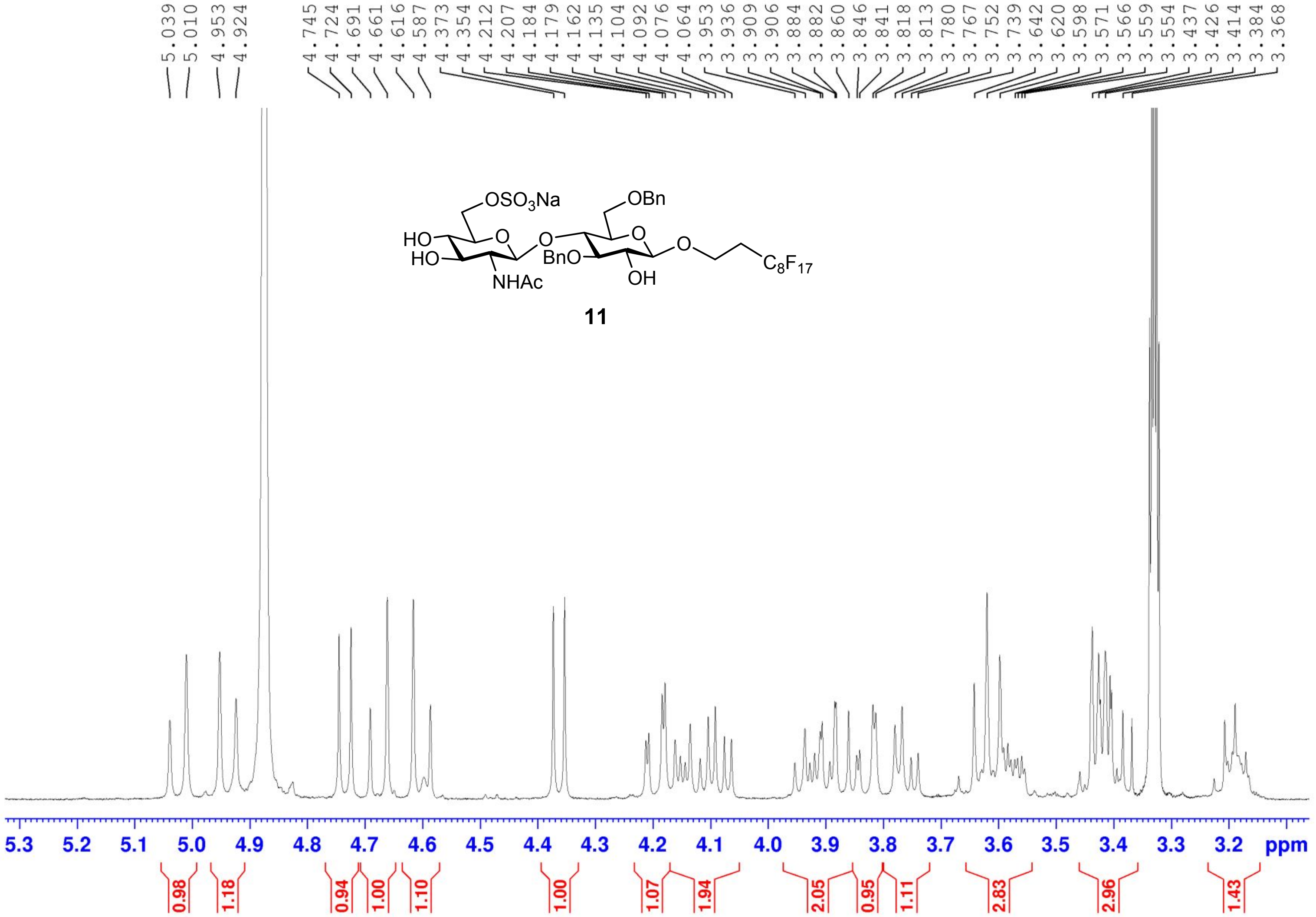

Compound 11 $^{13}C\{^{1}H\}$ NMR (100 MHz, CD_3OD)

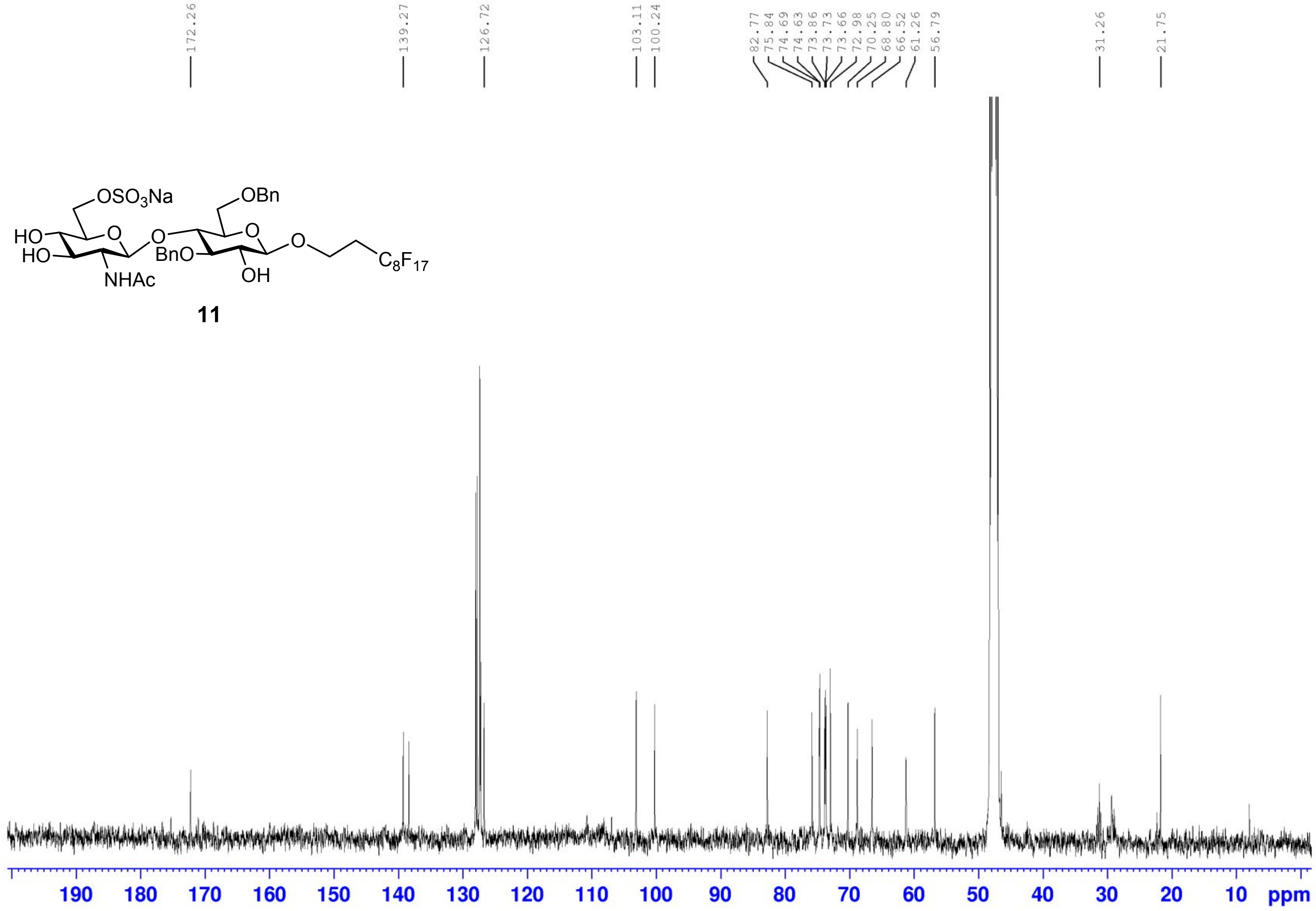

Compound 31 ^{1}H-NMR (400 MHz, CD_3OD)

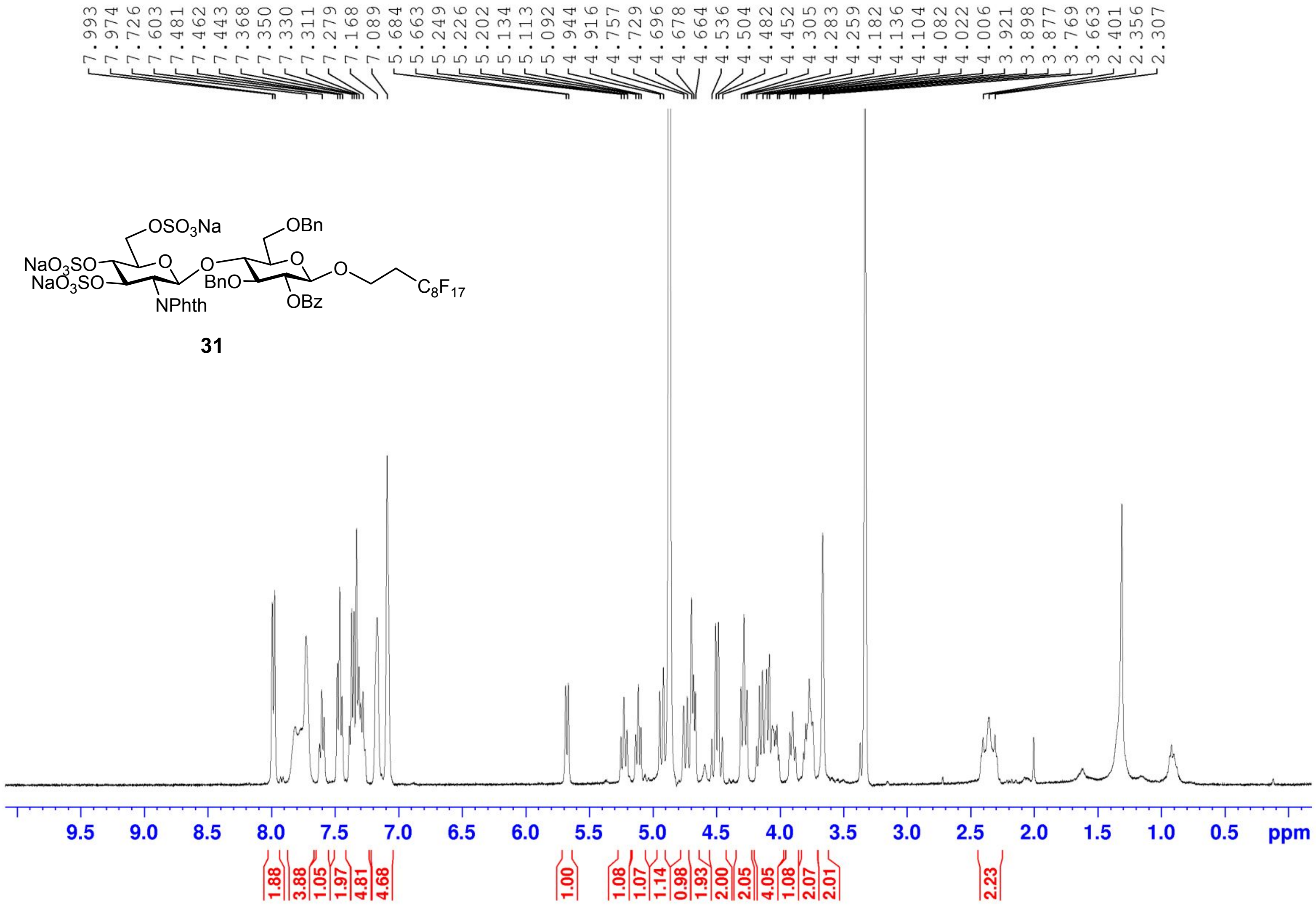

Compound 31 ^{1}H-NMR (400 MHz, CD_3OD)

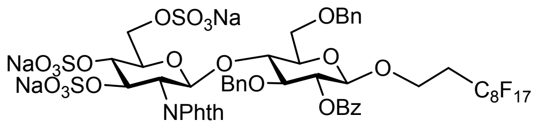

31

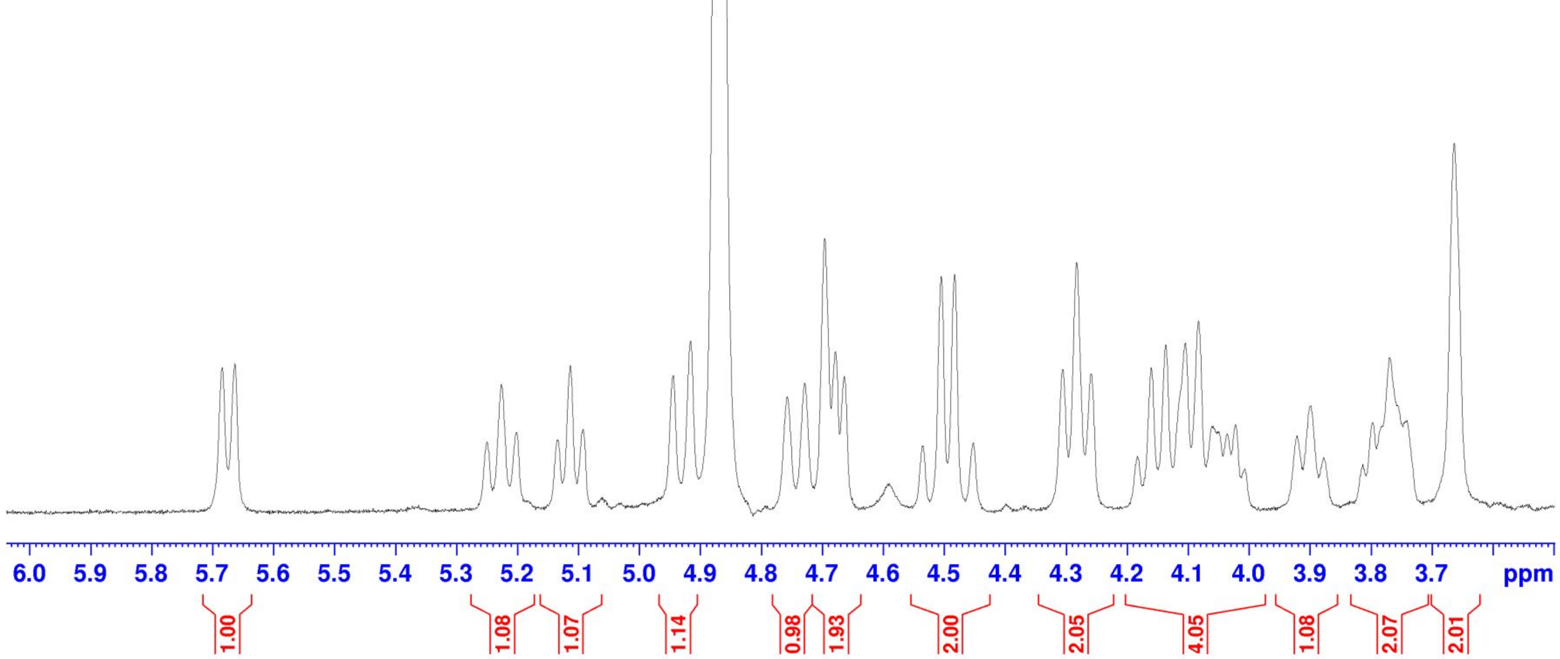

Compound 31 $^{13}C\{^{1}H\}$ NMR (100 MHz, CD_3OD)

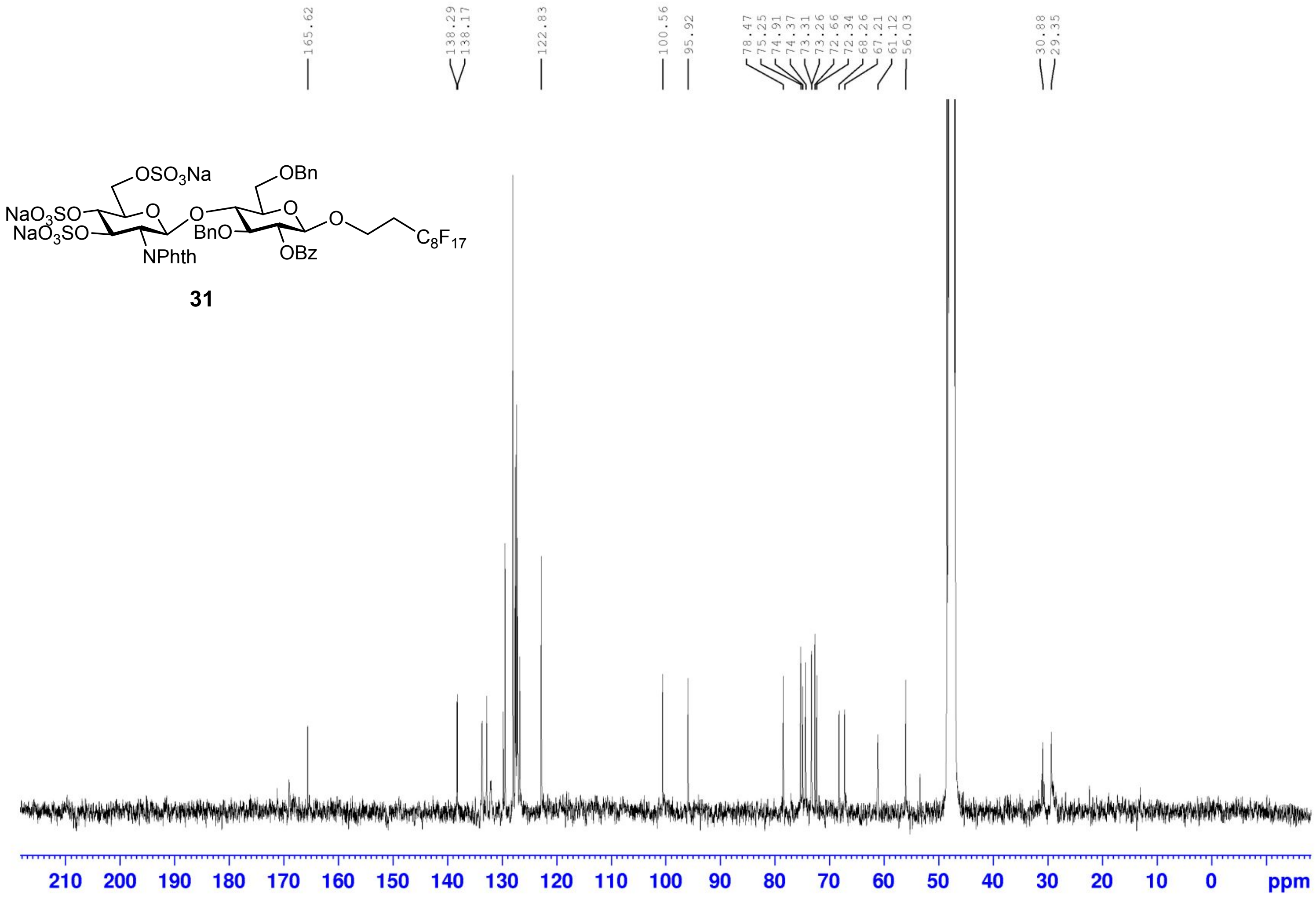

Compound 12 ^{1}H-NMR (400 MHz, CD_3OD)

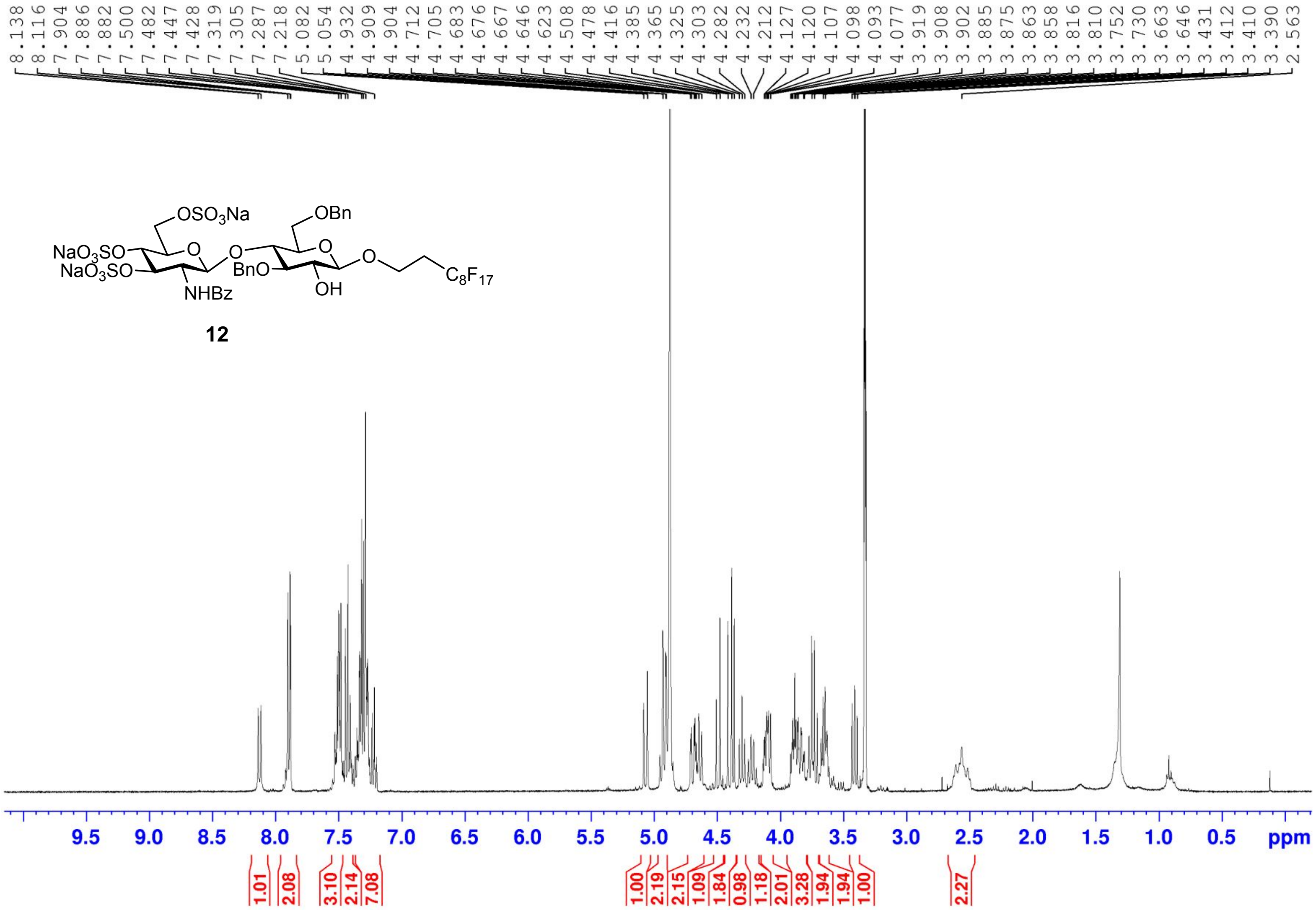

Compound 12 ^{1}H-NMR (400 MHz, CD_3OD)

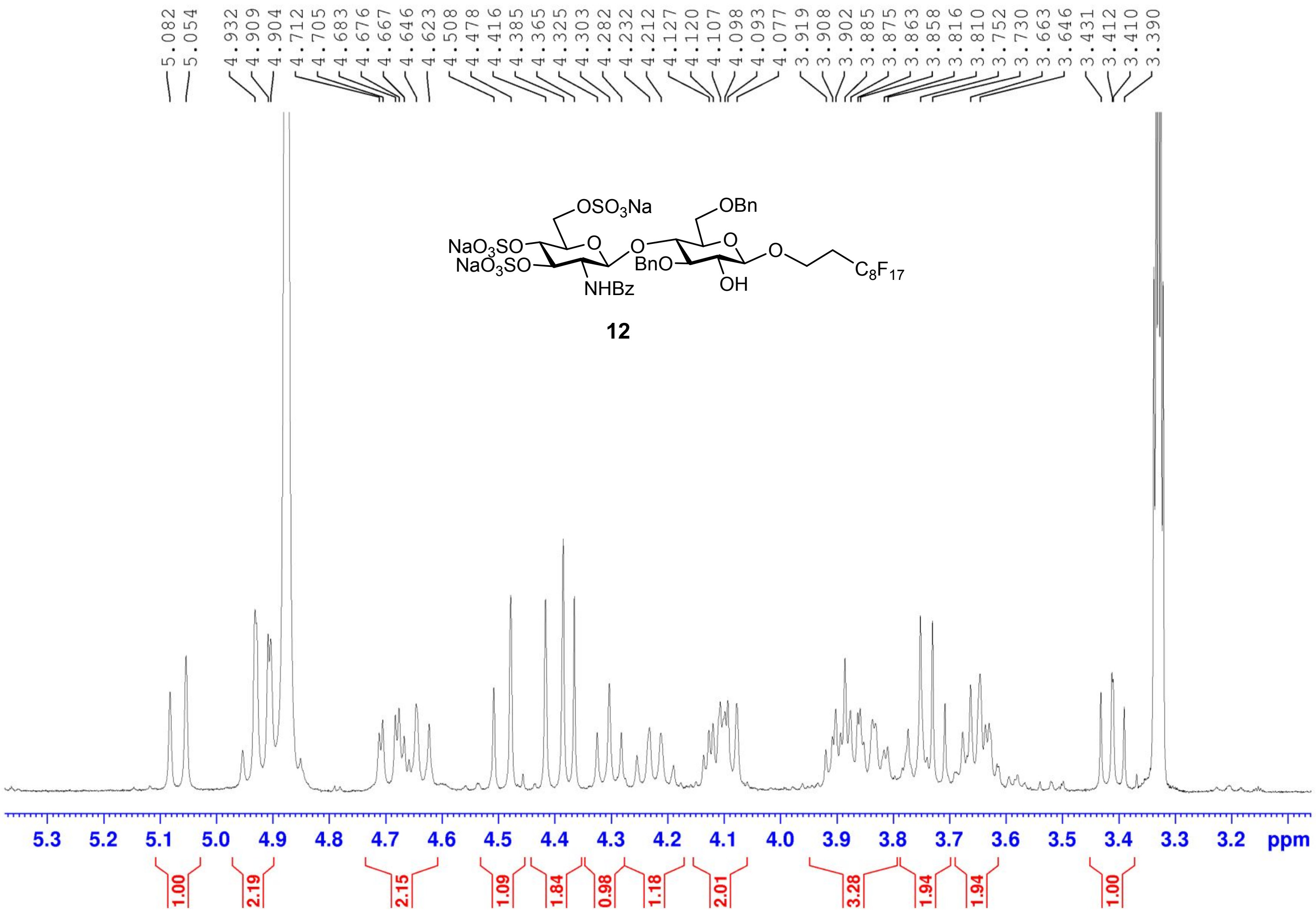

Compound 12 $^{13}C\{^{1}H\}$ NMR (100 MHz, CD_3OD)

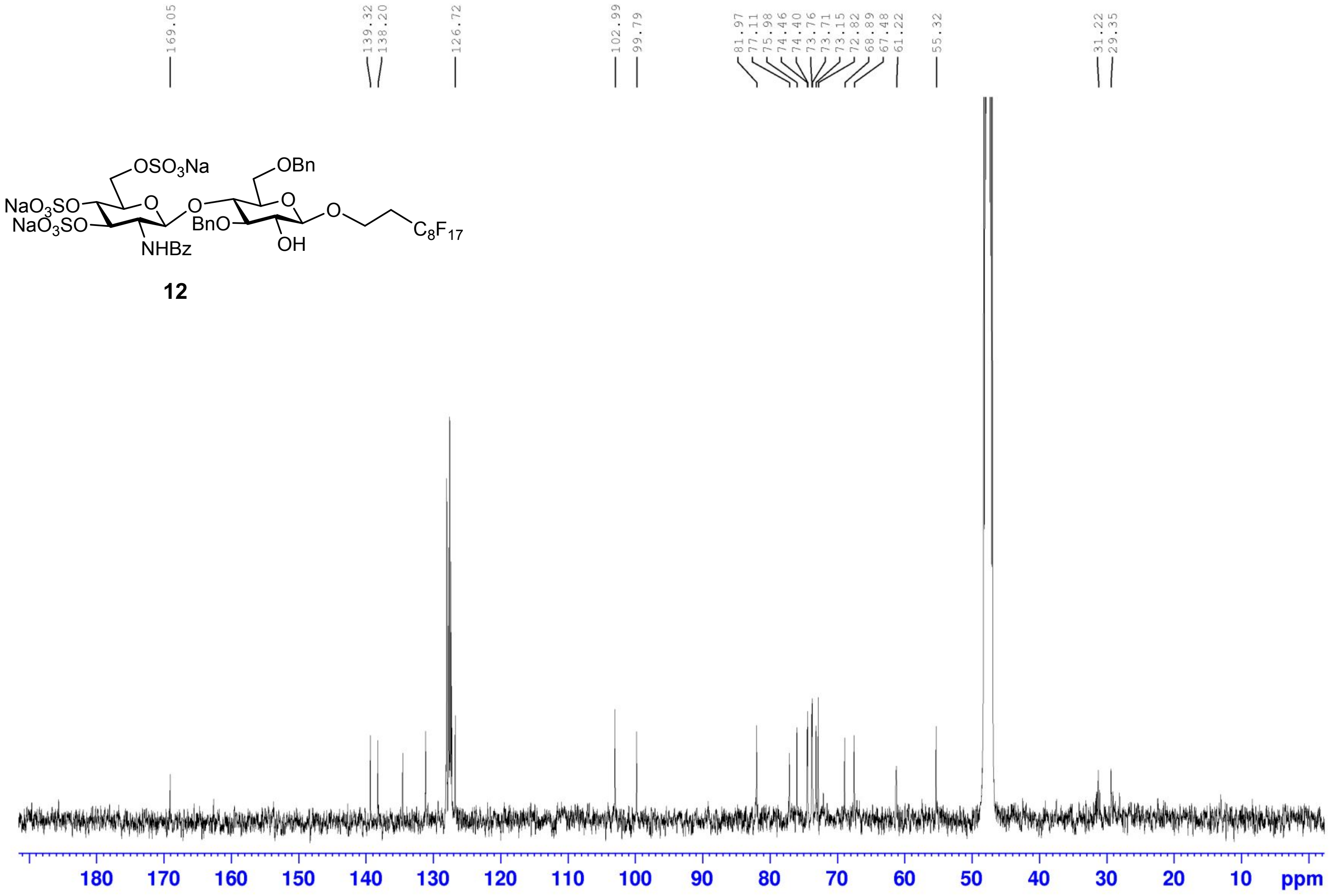

Compound 13 ^{1}H-NMR (400 MHz, CD_3OD)

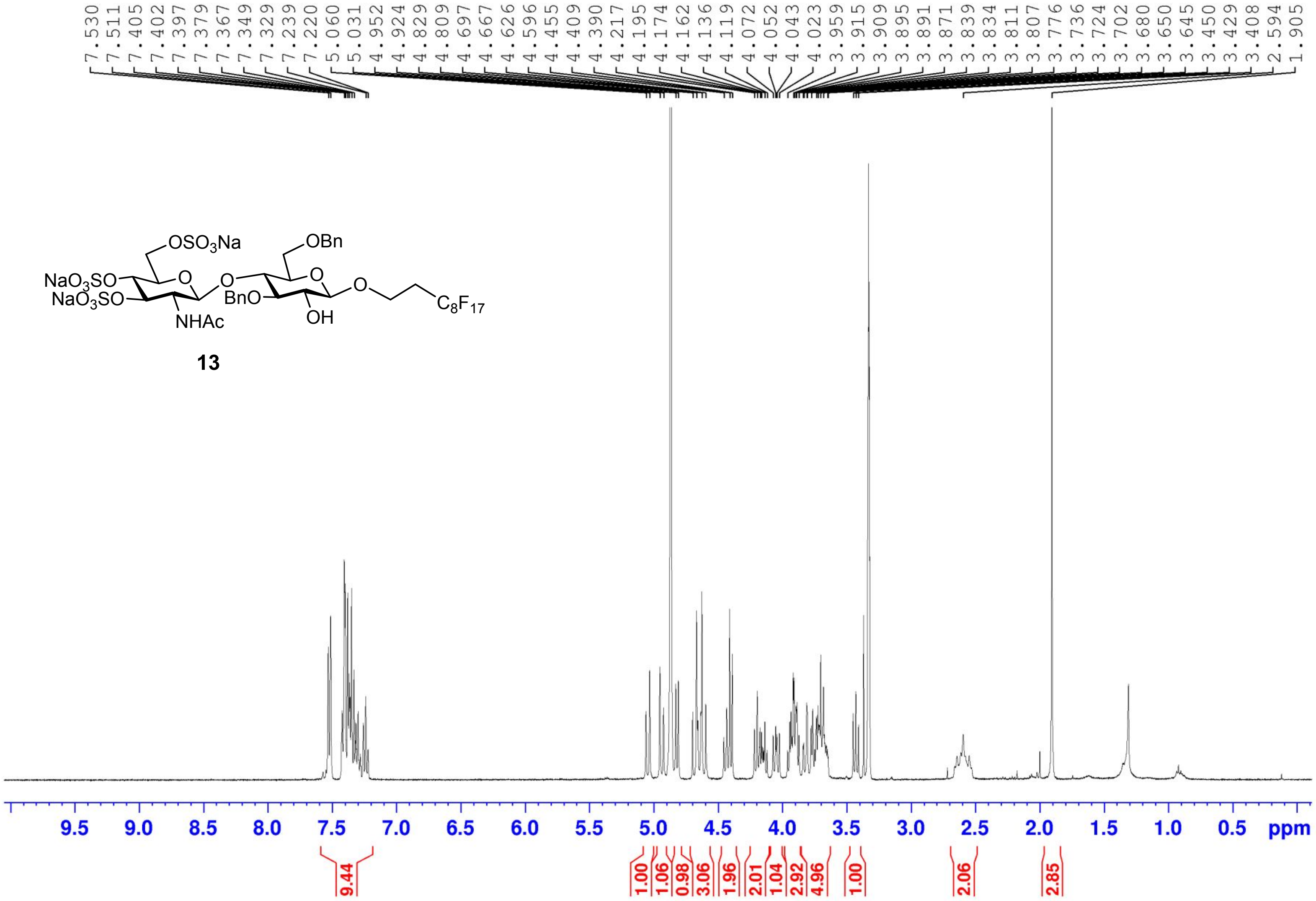

Compound 13 ^{1}H-NMR (400 MHz, CD_3OD)

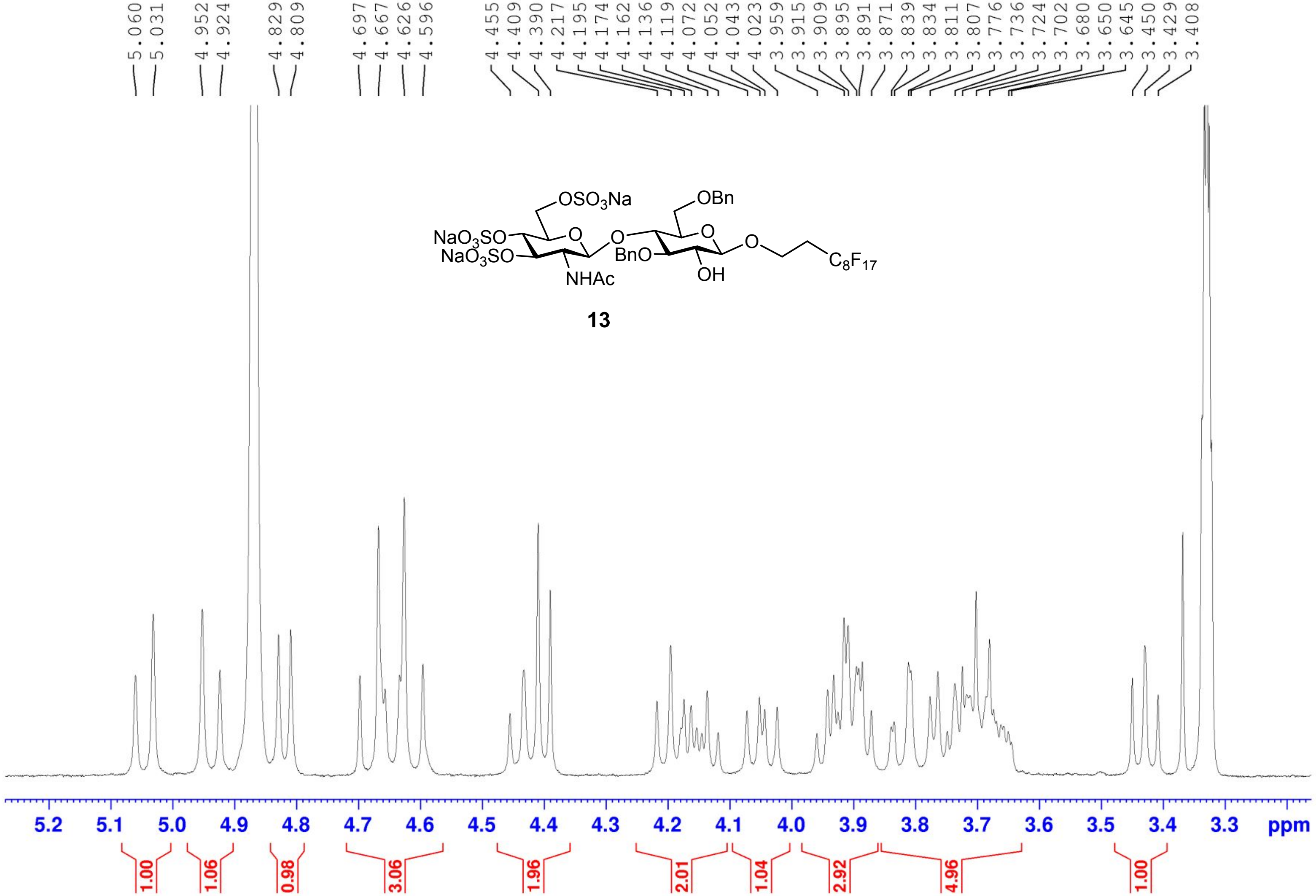

Compound 13 $^{13}C\{^{1}H\}$ NMR (100 MHz, CD_3OD)

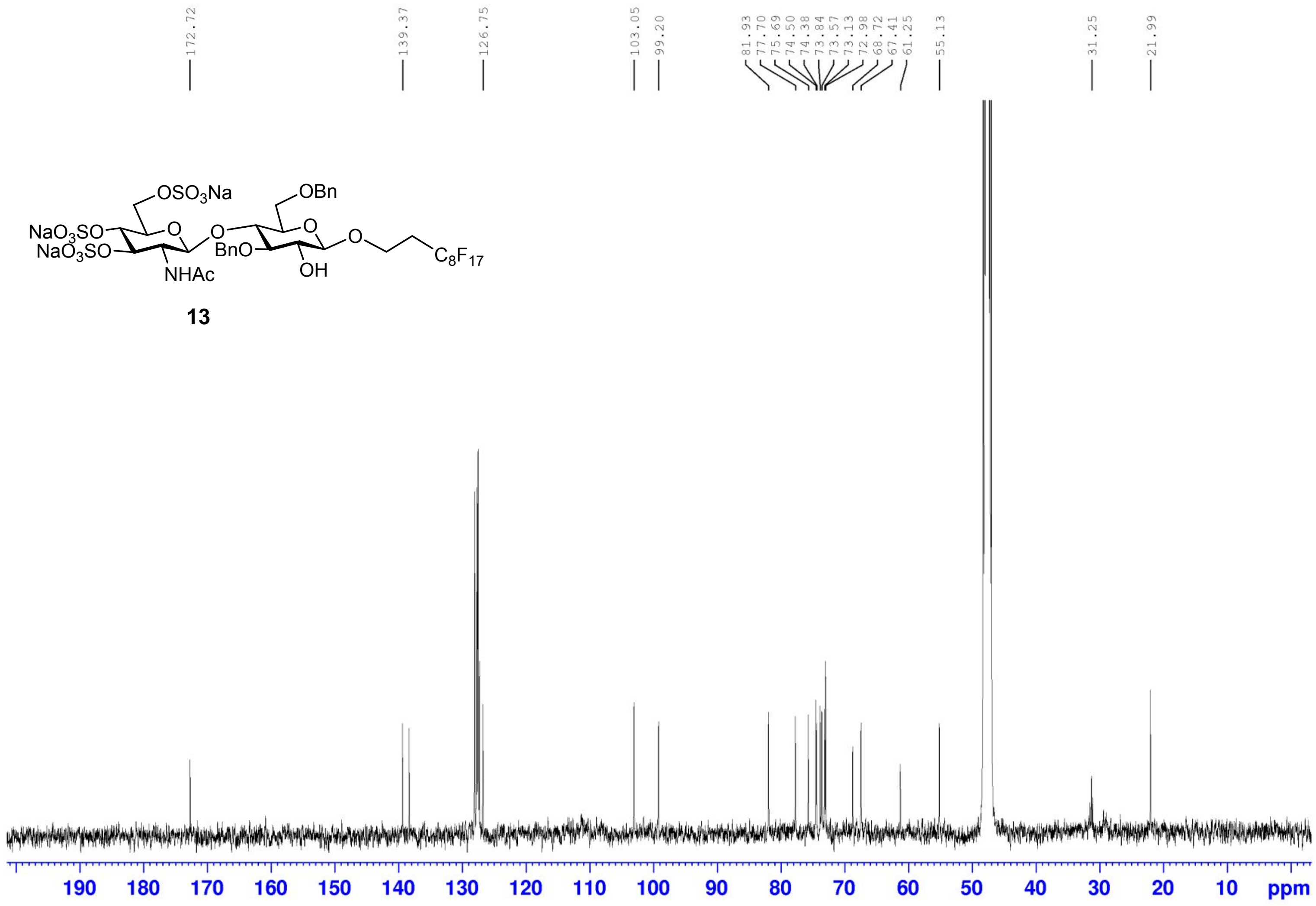

Supplement: Supplementary file 1 [file jo6c00064_si_001.pdf]
